# Homeobox oncogene activation by pan-cancer DNA hypermethylation

**DOI:** 10.1186/s13059-018-1492-3

**Published:** 2018-08-10

**Authors:** Jianzhong Su, Yung-Hsin Huang, Xiaodong Cui, Xinyu Wang, Xiaotian Zhang, Yong Lei, Jianfeng Xu, Xueqiu Lin, Kaifu Chen, Jie Lv, Margaret A. Goodell, Wei Li

**Affiliations:** 1 School of Biomedical Engineering, School of Ophthalmology and Optometry and Eye Hospital, Wenzhou Medical University, Wenzhou, 325011 Zhejiang China; 2 Division of Biostatistics, Dan L Duncan Cancer Center, Baylor College of Medicine, Houston, TX 77030 USA; 3 Stem Cells and Regenerative Medicine Center, Baylor College of Medicine, Houston, TX 77030 USA; 4 Program in Developmental Biology, Baylor College of Medicine, Houston, TX 77030 USA; 5 Wenzhou Institute of Biomaterials and Engineering, Chinese Academy of Sciences, Wenzhou, 325000 China; 6 Center for Epigenetics, Van Andel Research Institution, Grand Rapids, MI 49503 USA

**Keywords:** Pan-cancer analysis, DNA methylation, Whole-genome bisulfite sequencing, Gene-body, Hypermethylation, Transcription, Homeobox oncogene, Methylation editing

## Abstract

**Background:**

Cancers have long been recognized to be not only genetically but also epigenetically distinct from their tissues of origin. Although genetic alterations underlying oncogene upregulation have been well studied, to what extent epigenetic mechanisms, such as DNA methylation, can also induce oncogene expression remains unknown.

**Results:**

Here, through pan-cancer analysis of 4174 genome-wide profiles, including whole-genome bisulfite sequencing data from 30 normal tissues and 35 solid tumors, we discover a strong correlation between gene-body hypermethylation of DNA methylation canyons, defined as broad under-methylated regions, and overexpression of approximately 43% of homeobox genes, many of which are also oncogenes. To gain insights into the cause-and-effect relationship, we use a newly developed dCas9-SunTag-DNMT3A system to methylate genomic sites of interest. The locus-specific hypermethylation of gene-body canyon, but not promoter, of homeobox oncogene DLX1, can directly increase its gene expression.

**Conclusions:**

Our pan-cancer analysis followed by functional validation reveals DNA hypermethylation as a novel epigenetic mechanism for homeobox oncogene upregulation.

**Electronic supplementary material:**

The online version of this article (10.1186/s13059-018-1492-3) contains supplementary material, which is available to authorized users.

## Background

Upregulation of growth-promoting oncogenes is one of the key steps during tumorigenesis [1]. Genetic alterations underlying such oncogene upregulation have been extensively studied, including single nucleotide variation, chromosome translocation, focal amplification, and recently reported disruption of chromosome neighborhoods [2]. Meanwhile, cancers have also long been recognized to be not only genetically but also epigenetically distinct from their tissues of origin, yet little is known about the epigenetic alterations that can cause oncogene activation.

DNA methylation is the most extensively documented epigenetic modification that can influence cell fate and gene expression [3]. Previous DNA methylation analyses have been largely focused on long-range (> 100 kb) hypomethylation corresponding to lamina-associated domains (LAD) [4], variably methylated islands and shores [5], and highly methylated domains [6]. In most normal cells, DNA methylation patterns are stable [7], with 70–80% of all CpGs being methylated, and the remaining unmethylated CpGs tend to cluster together to form interspersed under-methylated regions (UMRs). These UMRs are generally associated with active regulatory regions, such as promoters and enhancers [8]. Aberrant DNA methylation has been repeatedly observed in many cancer types [9, 10], including colorectal [4, 11], lung [12], breast cancers [13], and hematological tumors. Furthermore, promoter hypermethylation-induced silencing of tumor suppressor genes [14, 15] is usually thought to be a key epigenetic event of tumorigenesis.

Besides promoter hypermethylation, several studies have established a positive correlation between gene expression and gene-body DNA methylation [16–19]. For example, the deoxycytidine-mediated gene-body hypomethylation has been shown to cause gene repression [11]. However, the causal claim in this study was based on the global hypomethylation agent 5-aza-2′-deoxycytidine, which lacks specificity and potentially suffers from significant off-target effects. For example, about 42% (188,631 out of 482,421) of the probes on Illumina Human Methylation 450 K BeadChip data were hypomethylated after deoxycytidine treatment in HCT116 cell [11]. Thus, whether the repression of a specific gene was caused by gene-body hypomethylation of the same gene remained unclear.

Recently, broad (i.e. > 3.5 kb) UMRs were reported as DNA methylation canyons [20] or DNA methylation valleys [21]. Canyons usually span promoters and gene-bodies and are very conserved across almost all normal cells. Canyon-associated genes are specifically enriched for developmental regulators, many of which have low or no expression in normal cells [20]. Alterations in canyon borders in hematopoietic stem cells are associated with dysregulated genes in acute myeloid leukemia [20] and promoters within canyons are hypermethylated in a colon cancer [21].

In this study, to gain insights into the functional role of DNA methylation canyon in tumorigenesis, we conducted an integrative analysis of 4174 genome-wide profiles, including whole-genome bisulfite sequencing (WGBS) data from 30 normal tissues and 35 solid tumors across seven major cancer types. Our pan-cancer analysis followed by functional validation using dCas9 mediated DNA methylation editing revealed an unexpected causal role of gene-body canyon hypermethylation for the activation of homeobox oncogenes.

## Results

### Identification of human reference under-methylated regions

**Fig. 1:**
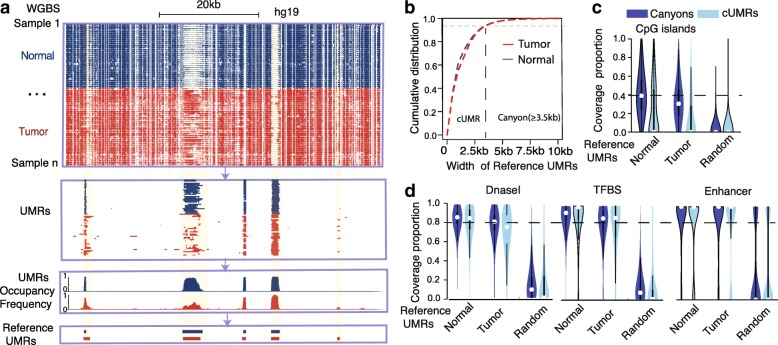
Human reference UMRs. **a** The statistical framework for the identification of conserved reference UMRs using WGBS data from 30 normal tissues and 35 solid tumors (Online Methods). **b** Cumulative distribution of UMR width for normal and tumor samples. Reference canyons (length > 3.5 kb) account for about 6% of all reference UMRs. The remaining short UMRs are regarded as control cUMRs. **c** Percentage of reference canyons/cUMRs covered by CpG islands (downloaded from UCSC). For each reference UMR, the percentage of UMR covered by the CpG islands is defined as the length of the UMR covered by CpG islands (either partially or entirely) divided by the total length of the UMR. Random represents 46,384 randomly selected regions for human genome that have the same length distribution, but without overlapping with reference UMRs. **d** Percentage of reference canyons/cUMRs covered by DNase I hypersensitivity clusters (DNaseI cluster) 125 cell types [23], transcription factor binding site clusters (TFBS cluster) of 161 TFs in 91 cell types [24], and enhancer clusters of H3K27ac peak regions in 88 human cell types [25]

To fully characterize DNA methylation canyons and their alterations across diverse tumor types, we designed a comprehensive pipeline (see “Methods”) to define the human reference UMRs using WGBS data from 30 normal tissues and 35 solid tumors across seven tumor types (Fig. 1a and Additional file 1: Table S1). A total of 46,384 recurrent (Poisson *p* value < 1.0e-8) human reference UMRs (tumor and normal UMRs combined) were identified that cover approximately 2.2% of the genome and also overlap with 71% (18,551) of 26,233 RefSeq genes (Additional file 2: Table S2). About 2935 (6.3%) of reference UMRs are > 3.5 kb and thus are defined as reference DNA methylation canyons. The remaining short UMRs are regarded as control (cUMRs, Fig. 1b).

Vertebrate CpG islands identified based on GC content and the observed to expected value (O/E value) of dinucleotide CpG have been shown to be associated with transcription start sites with low methylation [22]. Interestingly, CpG islands can only explain, on average, 40–50% of reference UMRs (Fig. 1c). On the other hand, most (> 80%) of the reference UMRs are covered by active cis-regulatory elements collected from hundreds of cell types, including DNase I hypersensitive sites [23], clusters of transcriptional factor binding sites [24], and enhancers [25] (Fig. 1d**)**. These results indicate that the reference UMRs and canyons are associated with active regulatory regions yet distinct from CpG islands.

### DNA methylation canyons are prone to hypermethylation in cancers

**Fig. 2:**
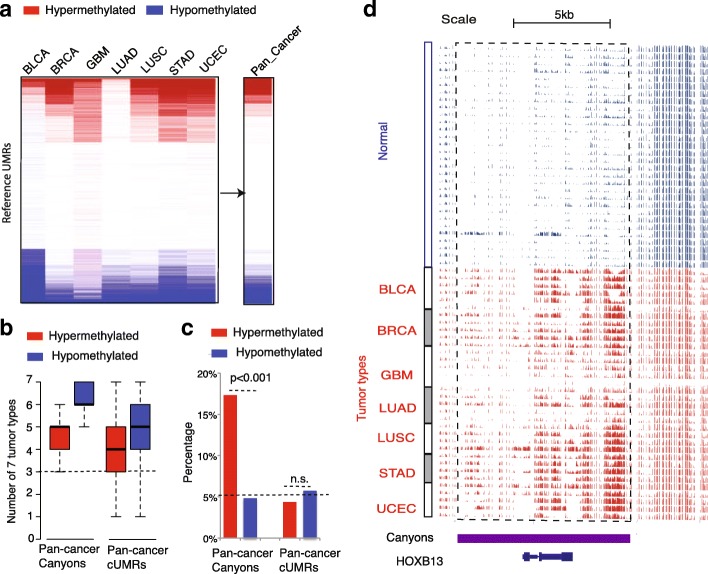
Pan-cancer differentially methylated UMRs. **a** The statistical framework for the identification of pan-cancer hypermethylated or hypomethylated UMRs. *Red* (*blue*) *lines* represent significant methylation increase (decrease) (*p* value < 0.001corrected by BH) in each tumor type and in pan-cancer. Tumors types include bladder urothelial carcinoma (BLCA), breast invasive carcinoma (BRCA), glioblastoma multiforme (GBM), lung adenocarcinoma (LUAD), lung squamous cell carcinoma (LUSC), stomach adenocarcinoma (STAD), and uterine corpus endometrial carcinoma (UCEC). **b** Pan-cancer differential UMRs found in individual tumor type. **c** Percentage of human reference canyons/cUMRs that are either hypermethylated or hypomethylated in pan-cancer. **d** DNA methylation genome browser tracks of a pan-cancer hyper-methylated canyon around homeobox gene *HOXB13*

To uncover aberrant UMRs in cancers, we first used a Shannon entropy-based method QDMR [26] to remove heterogeneous UMRs across normal tissues. This is inspired by recent advances in the analysis of GWAS data, in which high frequency mutations from a normal cohort will be removed since they are not likely to be associated with the disease phenotype. We then implemented a beta statistical framework to identify pan-cancer differentially methylated (BH corrected *p* value < 0.001) UMRs, which are significantly altered in most of 35 tumors but show almost no change within 30 normal tissues (see “Methods”). The resulting pan-cancer differential UMRs are thus unlikely to be artifacts due to the lack of matched normal tissues in our analysis. These pan-cancer differential UMRs can be further divided into four categories: (1) tumor-hypermethylated canyons; (2) tumor-hypermethylated cUMRs; (3) tumor-hypomethylated canyons; and (4) tumor-hypomethylated cUMRs (Fig. 2a and Additional file 3: Table S3). About 90% of pan-cancer differential UMRs can be recurrently identified in at least three individual tumor types (Fig. 2b). Notably, while cUMR has almost an equal number of hypermethylation and hypomethylation, canyons are surprisingly much more prone to hypermethylation (18%), but not hypomethylation (5%), in tumors (chi-square test *p* value < 0.001) (Fig. 2c). For example, a large (> 10 kb) pan-cancer hypermethylated canyon is located around *HOXB13* (Fig. 2d), an oncogene in ovarian [27] and breast [28] cancers.

### Hypermethylated canyons are enriched for homeobox genes and oncogenes

**Fig. 3:**
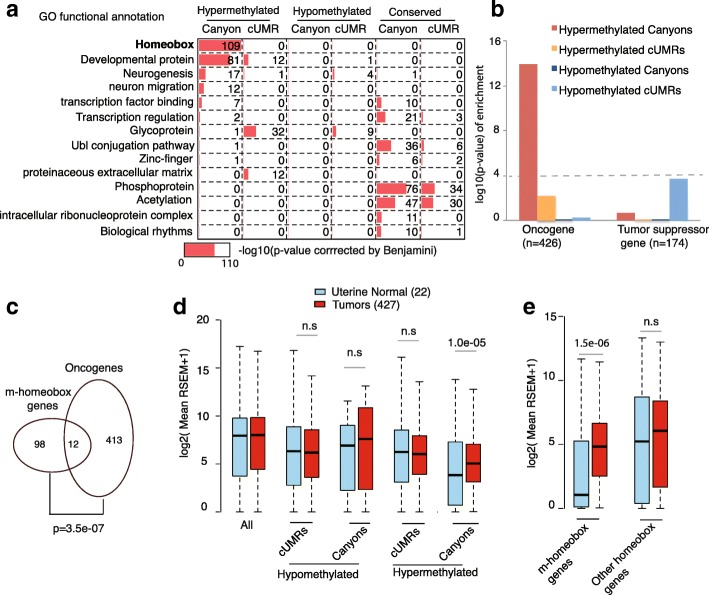
Canyon hypermethylation is associated with increased expression of homeobox oncogenes. **a** DAVID functional annotation of pan-cancer hypermethylated, hypomethylated, and conserved canyons/cUMRs. The *p* values were adjusted by the BH method. **b** Oncogene or tumor suppressor [31] gene enrichment levels in the pan-cancer hypermethylated/hypomethylated canyon/cUMRs. *P* values were computed by Fisher’s exact test. **c** *Venn diagram* showing the overlap between pan-cancer canyon hypermethylated homeobox genes (m-homeobox genes) and oncogenes. *P* value was computed by Fisher’s exact test. **d** *Boxplots* showing mean gene expression distribution of five categories gene sets of pan-cancer hypermethylated/hypomethylated canyons/cUMRs associated genes and all genes in the uterine tumor (427) and normal (22) samples. P values were calculated by Wilcoxon signed-rank test. *Boxplots* represent the interquartile range (25–75%), with the median; *whiskers* correspond to 1.5 times the interquartile range. **e** Mean gene expression distribution of the m-homeobox genes and the other homeobox genes between uterine tumors (427) and normal (22) samples. The average expression of each gene was first computed across 22 normal samples or 427 tumor samples. The *boxplot* shows the average expression of all genes

Pan-cancer differential UMRs exhibit distinct genomic features (Additional file 4: Figure S1). Hypomethylated canyon/cUMRs have low-CpG density, are mainly located in intergenic regions, and have no significant functional enrichment (Fig. 3a) for their associated genes, and thus are excluded from further analysis. In contrast, hypermethylated canyon/cUMRs have high CpG density and are mainly located in promoters and gene-bodies. Functional annotation of their associated genes (Fig. 3a and Additional file 5: Table S4) revealed that the 434 pan-cancer hypermethylated canyon (but not cUMR) genes include a significant (*p* value = 2.6e-109) amount (109, i.e. ~ 43% of 256 total) of homeobox genes [29] (named as m-homeobox hereafter), a superfamily of transcription factors (TFs) that are critical for cellular growth and differentiation [30]. To further investigate the role of hypermethylated canyons in tumorigenesis, we tested their enrichment in oncogenes or tumor suppressor genes defined in the COSMIC [31] Cancer Gene Census database. Unexpectedly, those pan-cancer hypermethylated canyons (but not cUMRs) are enriched in oncogenes but not in tumor suppressor genes (Fig. 3b). Notably, those m-homeobox genes are also significantly enriched in oncogenes (Fig. 3c binomial test *p* value = 3.5e-07).

### Hypermethylated canyons are strongly associated with increased expression of homeobox oncogenes

To evaluate the functional consequence of canyon hypermethylation, we compared gene expression between uterine corpus endometrial carcinoma (UCEC) tumors and matched normal tissues. Surprisingly, pan-cancer hypermethylated canyon genes have significantly higher gene expression in UCEC tumors than in normal samples (Wilcoxon signed-rank test *p* value = 1.0e-05, r-value = 0.49; Fig. 3d). Accordingly, the 110 m-homeobox genes (but not other homeobox genes) also have higher gene expression in UCEC tumors (Wilcoxon signed-rank test *p* value = 1.5e-06, r-value = 0.38; Fig. 3e). In contrast, no global expression difference was observed for hypomethylated canyons/cUMRs and hypermethylated cUMRs. Furthermore, the unique association between increased expression and canyon hypermethylation was also observed in four other tumor types including BLCA, BRCA, LUAD, and LUSC (Additional file 4: Figure S2). Together, our analysis suggested a previously unrecognized link between canyon hypermethylation and increased expression of homeobox oncogenes.

### Hypermethylation of gene-body but not promoter within canyon is associated with increased gene expression

**Fig. 4:**
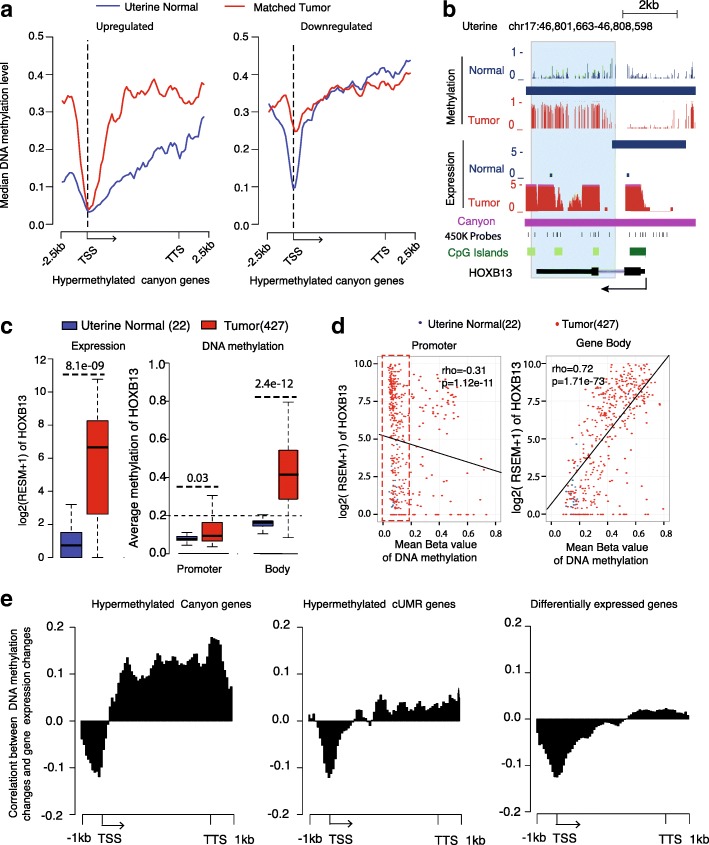
Hypermethylation of gene-body but not promoter within canyon is associated with increased gene expression. **a** DNA methylation of upregulated and downregulated pan-cancer hypermethylated canyon genes between a uterine tumor (Data ID: TCGA-AX-A1CI-01A) and its matched normal sample (TCGA-AX-A1CI-11A). **b** Increased methylation in gene-body and low methylation around TSS for *HOXB13*. **c** Gene expression and DNA methylation in *HOXB13* promoter (chr17: 46,806,000-46,807,000) and gene-body (chr17:46,802,200-46,805,999) between uterine normal (22) and tumor (427) samples. *P* values were calculated by Wilcoxon signed-rank test. **d** Correlation between *HOXB13* expression and methylation level at gene promoter (*left*) and gene-body (*right*) in uterine normal (22) and tumor (427) samples. The rho and *p* value were computed by Spearman’s rank test. **e** Locus-specific spearman’s rank correlation of gene expression change and DNA methylation change (see “Methods”) between a uterine tumor and its matched normal sample in Fig. 4a for pan-cancer hypermethylated canyon genes (*left*), pan-cancer hypermethylated cUMR genes (*middle*), and all differentially expressed genes (*right*)

To understand the relationship between hypermethylated canyon and increased gene expression, we plotted canyons/cUMRs around their associated genes. Although almost all the canyons/cUMRs are enriched in promoters, about 40% of hypermethylated canyons also cover the entire gene-bodies (Additional file 4: Figure S3). This observation suggested that gene-body (but not promoter) canyon hypermethylation might explain the gene overexpression, consistent with previously reported positive correlation between gene-body methylation and gene expression [11, 32]. As expected, in a comparison between a UCEC tumor and its matched normal tissue, we found that, for upregulated hypermethylated canyon genes in tumors, methylation level increased dramatically in gene-bodies (two-sided t-test *p* value = 3.8e-08) but not in promoters (Fig. 4a). In contrast, for downregulated hypermethylated canyon genes, DNA methylation increased only at gene promoters (two-sided t-test *p* value = 1.9e-06) but not in gene-bodies, consistent with the extensive studies of promoter hypermethylation associated with gene silencing. For example, the hypermethylated homeobox gene *HOXB13* overexpression and gene-body hypermethylation was observed in UCEC (Fig. 4b) and four other tumor types including BRCA, LUAD, LUSC, and STAD (Fig. 2d and Additional file 4: Figure S4a). In ovarian [27] and breast [28] cancer cell lines, *HOXB13* has been shown as an oncogene involved in upregulation of estrogen receptor (ER), increase of cancer cell proliferation, and invasiveness. Using Illumina 450 K methylation array and RNA-sequencing (RNA-seq) in a large cohort of UCEC tumors, we further validated that *HOXB13* had significantly increased gene expression and gene-body (but not promoter) methylation (Fig. 4c). Furthermore, hypermethylation of *HOXB13* gene-body (but not promoter) is strongly correlated with (Spearman’s rank correlation *p* value = 1.7e-73) their gene expression (Fig. 4d). Together, our data revealed that the canyon gene upregulation is mainly associated with hypermethylation of gene-bodies but not promoters within canyons.

### Canyon gene expression is more susceptible to gene-body DNA methylation change

To better understand the role of canyon in the relationship between hypermethylation and increased gene expression, we performed locus-specific correlation analysis for the same UCEC tumor and matched normal sample. Each gene (normalized into 5 kb) and the flanking 1-kb regions were split equally into non-overlapping 70 bins with 100 bp for each bin. The Spearman’s rank correlation between DNA methylation and gene expression was computed for each bin (Fig. 4e). As expected, for all differentially expressed genes, there is a strong negative correlation in promoter and relatively weak positive correlation in gene-body [18]. However, the canyon genes exhibit much stronger positive correlation in gene-body than hypermethylated cUMR genes and differentially expressed genes, although the negative correlation in promoter is similar across all three gene sets. Thus, the hypermethylated canyon genes represent a unique set of genes, whose expression might be more susceptible to gene-body DNA methylation change.

### Gene-body canyon hypermethylation by dCas9-SunTag-DNMT3A can directly increase oncogene *DLX1* expression

**Fig. 5:**
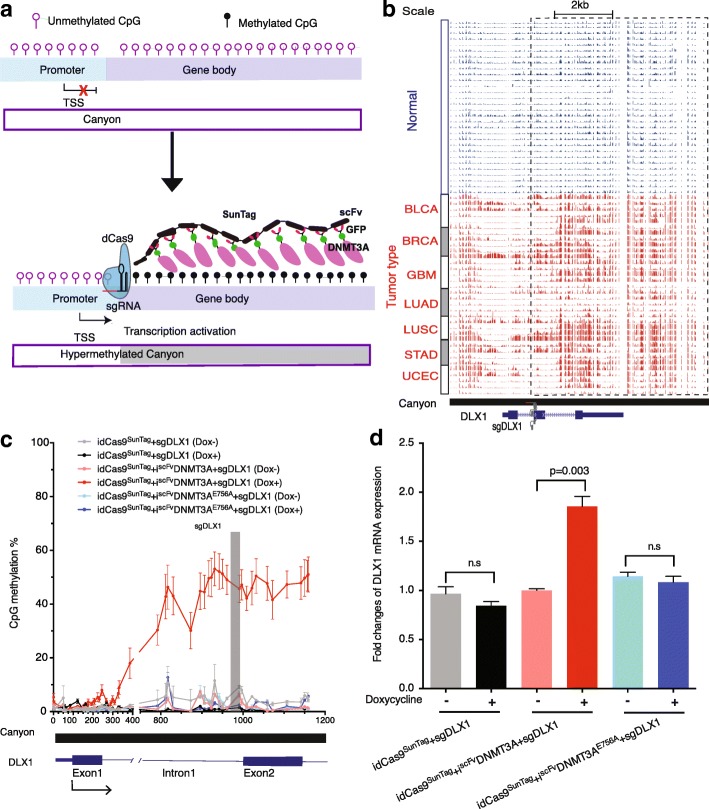
Gene-body canyon hypermethylation by dCas9-SunTag-DNMT3A can directly increase oncogene *DLX1* expression. **a** *Schematic graph* for dCas9-SunTag-DNMT3A DNA methylation editing system. De-activated Cas9 (dCas9) was fused to SunTag epitopes and single-chain variable fragment (scFv) was fused to GFP and DNMT3A to methylate the gene-body canyon and activate gene expression. **b** Genome browser tracks of gene-body hypermethylated canyons around homeobox gene *DLX1* across 30 normal and 35 tumor samples. **c** CpG DNA methylation level dramatically increased at the gene-body of *DLX1* after adding guide RNA *DLX1* (*gray bar*) with induction of dCas9^SunTag^ and ^scFv^DNMT3A, yet the methylation level in gene promoter was not affected. CpG DNA methylation level was calculated based on two biological replicates. *Error bars* represent mean ± s.e.m. of biological replicates. **d** qPCR shows significant increase of gene expression of *DLX1* with induction of dCas9^SunTag^ and ^scFv^DNMT3A compared to the same cells without induction. *P* value was computed by two-sided Student’s t-test

Recent advances in epigenetic editing allow the targeted modulation of DNA methylation of regions of interest (ROI) by the fusion of DNMT3A or TET1 with a nuclease-deactivated Cas9 (dCas9) [33, 34]. DNMT3A has been shown to occupy and methylate gene-bodies and intergenic regions involved in transcription upregulation of neurogenic genes in mouse postnatal neural stem cells [35]. To test whether gene-body canyon hypermethylation can directly lead to gene activation, we harnessed the newly developed dCas9-SunTag-DNMT3A system [36] to methylate gene-body ROIs (Fig. 5a) in the HEK293T human embryonic kidney cell line.

Due to the difference between cell lines and primary cells, the *HOX13B* gene-body is already highly methylated in HEK293T cells. We therefore decided to focus on two other m-homeobox genes, *DLX1* and *POU3F3*, in the dCas9-mediated methylation editing experiments. Gene-body canyon hypermethylation of *DLX1* and its aberrant overexpression were widely observed in four tumor types including BLCA, LUAD, LUSC, and UCEC (Fig. 5b and Additional file 4: Figure S4b). *DLX1* has also been found to promote ovarian cancer cell growth, cell migration, and invasion [37]. To specifically methylate the gene-body canyon of *DLX1* without affecting the promoter, single guide RNAs (sgRNA; sgDLX1) were designed in intron 1 of *DLX1* using the CRISPR analysis tool CHOPCHOP [38] (Fig. 5b). Induction of dCas9-SunTag-DNMT3A expression can methylate gene-body portion of the canyon (average methylation of 25 CpGs increases from 5.1% to 46.2%), while keeping the remaining part (mostly promoter) of the canyon largely unchanged (average methylation of 27 CpGs changes from 1.0% to 1.1%) (Fig. 5c). Quantitative polymerase chain reaction (qPCR) showed that the dCas9-SunTag-DNMT3A together with sgRNAs can directly increase *DLX1* gene expression by about twofold, comparing to the same cells without induction of dCas9^SunTag^ and ^scFv^DNMT3A. Furthermore, induction of dCas9^SunTag^ and ^scFv^DNMT3A^E756A^ with sgRNAs did not change *DLX1* expression, suggesting DNA methylation, rather than dCas9^SunTag^-DNMT3A complex, is responsible for increased *DLX1* expression (Fig. 5d). Similar to *DLX1*, another m-homeobox gene *POU3F3* has hypermethylation canyon in gene-body and aberrant overexpression in multiple tumor types including BLCA, LUSC, and UCEC (Additional file 4: Figure S5a and S5b). We used the dCas9-SunTag-DNMT3A methylation editing tool to specifically methylate the gene-body canyon of *POU3F3* (average methylation of 23 CpGs increases from 4.9% to 28.0%), while keeping the promoter at a low methylation level (average methylation of 71 CpGs changes only from 1.3% to 1.9%) (Additional file 4: Figure S5c). Consistent with the result of *DLX1*, the expression of *POU3F3* also exhibits significant increase (approximately twofold) due to gene-body canyon hypermethylation (Additional file 4: Figure S5d). Together, our methylation editing experiments on two independent homeobox genes (*DLX1* and *POU3F3*) provide robust experimental evidence that gene-body canyon hypermethylation can directly increase oncogene expression.

## Discussion

Our pan-cancer analysis of 35 solid tumors across seven cancer types revealed that DNA hypermethylation preferentially occurs in broad (i.e. canyons) but not in short UMRs. To overcome concerns regarding the lack of matched normal tissues, we removed tissue-specific UMRs in our pan-cancer analysis and then focused on UCEC tumors and their matched normal tissues in the downstream functional analysis. The hypermethylated canyon genes are strongly associated with increased expression of homeobox oncogenes and represent a unique set of genes whose expression might be more susceptible to gene-body methylation change. Our locus-specific dCas9-mediated DNA methylation editing experiment reveals an unexpected causal role of gene-body canyon hypermethylation for gene activation. This is fundamentally different from the well-known promoter hypermethylation [10, 39–41] leading to the silence of tumor suppressor genes.

In the human genome, most gene-bodies have low CpG density and are heavily methylated [17, 32]. Gene-body methylation is involved in preventing alternative promoters, spurious transcription initiation, and retrotransposon elements to maintain gene transcription efficiency [42, 43]. Our study is the first to use a locus-specific DNA methylation editing system to prove gene-body canyon hypermethylation can directly increase expression of a unique set of homeobox oncogenes.

Homeobox genes comprise a superfamily of TFs that are critical for cellular growth and differentiation. The homeodomain (the evolutionary conserved helix–loop–helix DNA-binding motif) is usually present in the second exon. Homeobox genes in general have high CpG density, which might facilitate the establishment of DNA methylation canyons. In fact, about 67% (157 out of 234) of homeobox genes are associated with reference canyons (Additional file 3: Table S3). Growing evidence has demonstrated that homeobox genes are frequently dysregulated in cancers [44]. However, very few of them are associated with pan-cancer oncogenic genetic signatures [45], such as copy number variation (CNV) and somatic mutation (Additional file 4: Figure S6). In contrast, the pan-cancer hypermethylated canyons are associated with ~ 43% of homeobox genes that are overexpressed in multiple tumors (Fig. 3e and Additional file 4: Figure S2b). This observation suggests that the gene-body canyon hypermethylation might be a dominant epigenetic mechanism for homeobox oncogene activation in tumors.

## Conclusions

Our data suggest that the pan-cancer gene-body canyon hypermethylation is a novel epigenetic mechanism for homeobox oncogene activation. Our finding might provide new insights into tumorigenesis, especially for those tumors that harbor low genetic alterations yet are largely epigenetically deregulated.

## Methods

### Public datasets

In this study, we used a total of 4174 genome wide datasets (Additional file 1: Table S1), including 65 WGBS profiles, 449 Infinium 450 K arrays, 3660 RNA-seq data from Roadmap Epigenomics [46], ENCODE [47], and the TCGA consortium [48], respectively. The TFs were downloaded from Human TF repertoire [49]. The homeobox genes were downloaded from HomeoDB2 database [29]. A total of 426 cancer dominant genes (oncogenes) and 128 cancer recessive genes (tumor suppressor gene) were defined by the COSMIC [31] database. The pan-cancer oncogenic signatures including CNV deletion (116) and amplification (151), somatic mutation (199), and DNA methylation (13) were identified by a hierarchical classification method of 3299 TCGA tumors from 12 cancer types [45]. Also, a human genome-wide enhancer cluster was obtained from the ChIP-seq datasets of H3K27ac peaks in 88 human cell types [25]. Gene expression values of normalized read counts by expectation-maximization (RSEM) from RNA-seq data of primary tumor and normal samples were obtained from the TCGA data portal (https://tcga-data.nci.nih.gov/docs/publications/tcga/) including 19 bladder normal and 408 urothelial carcinomas (BLCA), 113 breast normal and 1102 invasive carcinomas (BRCA), 59 lung normal and 515 adenocarcinomas (LUAD), 102 lung squamous normal and 502 carcinomas (LUSC), 35 stomach normal and 415 adenocarcinoma (STAD), 22 uterine normal and 437 corpus endometrial carcinoma (UCEC). Promoters were defined from 1 kb upstream to 500 bp downstream of RefSeq transcription start sites (TSS) and gene-bodies were defined from 500 bp downstream of RefSeq TSS to RefSeq transcription termination sites (TTS).

### Identification of reference under-methylated regions

We developed a comprehensive statistical framework to identify human reference UMRs from 65 high-quality WGBS profiles (genome-wide CpG coverage percentage > 90%; Additional file 1: Table S1), comprising 30 normal tissues and 35 primary solid tumors:

**Step 1:** For each WGBS profile, we used BSMAP [50] to trim adaptor, low-quality, and duplicated sequence with default threshold, aligned bisulfite-treated reads to the human genome (hg19). We used the coverage threshold of 4 reads to ensure the accuracy of CpG methylation detection [51]. The methylation ratio of each CpG covered with at least 4 reads was calculated by the module bsratio in BSMAP.

**Step 2:** The UMRs were identified that include at least four consecutive hypomethylated CpGs with the mean methylation ratio < 10% as described previously [20]. To reduce the effect of sparse CpG density in our UMR detection based on HMM model, we removed UMRs with Obs/Exp value of CpGs < 0.1.

**Step 3:** A total 3,521,985 redundant UMRs from multiple tissue and tumor WGBS profiles were reduced to 369,852 non-redundant ones through merging the overlapping UMRs among multiple samples. To describe genome-wide UMR enrichment distribution across tissue and tumor samples, the UMR frequency (UOF) of the intersect segment (*s*) among *N* samples was defined as$UOF(s)=\frac{\sum_{i=1}^N{s}_i}{N},$

$$ where\ {s}_i=\left\{\begin{array}{c}1,\kern0.5em if\kern0.5em the\  ith\  sample\  UMR\  covering\ segment\ s\\ {}0,\kern0.5em otherwise\kern17.25em \end{array}\right.. $$

UMR occupancy scores represent the UMR co-occupancy level of population-scale samples of normal tissues and tumors. The higher the UOF, the more conserved are the UMR in the population-scale samples. Conversely, a UOF decrease represents the UMR shortening or loss, suggesting that hypermethylation occurs in these regions at a population scale.

**Step 4:** Inspired by ChIP-seq peak calling for detection of significantly enriched regions, we detected reference UMRs from UOF profile within population of samples based on a Poisson test (*p* values < 1.0e-8), *p* values adjusted by the Benjamini and Hochberg (BH) method. These reference UMRs were identified for normal tissue (32,864) and tumors (45,081), respectively. A total of 46,384 recurrent UMRs were identified through combining the normal and tumor reference UMRs (Additional file 2**:** Table S2).

### Identification of pan-cancer differentially methylated UMRs

We sought to uncover common patterns of aberrant DNA methylation across diverse tumor types with low heterogeneity among normal tissues. A statistical framework was devised to identify pan-cancer differentially methylated UMRs.

**Step 1:** Normal tissue-specific UMRs were removed using a quantitative method QDMR [26] based on Shannon entropy with default threshold. The lower the entropy value, the bigger the difference of DNA methylation across sample. In total, 24,098 reference UMRs with low heterogeneity across normal samples were retained.

**Step 2:** The differential methylation (DM) analysis was performed by employing a likelihood ratio test method to dissect aberrant methylation between tumor and normal samples*.* The mean methylation level of the *i*_*th*_ UMR in the *j*_*th*_ normal sample is denoted as ${x}_{ij}^0$, while the methylation level in the *k*_*th*_ tumor sample is represented as ${x}_{ik}^1.$ Here ${x}_{ij}^0\sim Beta\left({\alpha}_i^0,{\beta}_i^0\right),\kern0.5em i\in \left[1,2,..,N\right],\kern0.5em j\in \left[1,2,..,{M}^0\right]$, *N* is the total number of UMRs and *M*^0^ is the number of the normal samples. In addition, ${x}_{ik}^1\sim Beta\left({\alpha}_i^1,{\beta}_i^1\right),k\in \left[1,2,..,{M}^1\right]\kern0.5em$and *M*^1^ is the number of the tumor samples. Then, the goal of testing if the *i*_*th*_ UMR is differential across tumor and normal samples is to determine if they have the same distribution parameters. This is equivalent to test the following hypothesis

$$ {H}_o:{\alpha}_i^0={\alpha}_i^1={\alpha}^s\  and\ {\beta}_i^0={\beta}_i^1={\beta}^s\kern0.5em vs $$

$$ {H}_1:{\alpha}_i^0\ne {\alpha}_i^1\  or\ {\beta}_i^0\ne {\beta}_i^1 $$

To this end, a likelihood ratio test for is adopted, whose test statistics are expressed as:

$$ {D}_i=-2\mathit{\ln}\prod \limits_{j=1}^{M^0+{M}^1}P\left({x}_{ij}|{\alpha}^s,{\beta}^s\right)+\mathit{\ln}\prod \limits_{k=1}^{M^0}P\left({x}_{ik}|{\alpha}^0,{\beta}^0\right)+\mathit{\ln}\prod \limits_{l=1}^{M^1}P\left({x}_{il}|{\alpha}^1,{\beta}^1\right) $$

Here, *D*_*i*_ approximately follows a *χ*^2^ distribution with degree of freedom *df*2 − *df*1 under *H*_*o*_, from which the *p* value can be computed as

$$ {p}_{value}=1-{\chi}^2\left({D}_i, df2- df1\right) $$

where df2 and df1 represent the degrees of freedom for the model under H_1_ and H_o_, which are 4 and 2, respectively. In the end, the *p* values for all the UMRs are adjusted to false discovery rate (FDR) using the BH method. The absolute DM values of UMRs were defined as the difference of mean methylation levels between tumor and normal samples. Both *p* value adjusted by BH method < 0.001 and DM value > 0.1 were used to identify the differentially methylated UMRs relative to all normal samples (Additional file 3**:** Table S3). To compare these differentially methylated UMRs with conserved UMRs across tumor types, we established two control groups: (1) 1398 conserved canyons; and (2) 9596 conserved cUMRs, which are not differentially methylated in all of the seven tumor types.

### Gene expression analyses

Differentially expressed m-homeobox genes were identified using the software edgR [52] with FDR-adjusted *P* value < 0.01 and relative fold changes of mean expression level > 2 (tumor vs norm).

### HumanMethylation450 BeadChip analysis

We selected a large cohort of Infinium Human Methylation 450 K BeadChip data for Uterine Corpus Endometrial Carcinoma (UCEC), including 22 normal and 427 primary tumor samples from TCGA (Additional file 1: Table S1). The probes with one or more single nucleotide polymorphisms (SNPs) were removed and the ComBat normalization was used to reduce the batch effect. DNA methylation levels of 482,421 CpG sites were measured as *β* values in the range of 0–1 that cover about 1.7% of total CpGs in the human genome. 450 K BeadChip probes are enriched in the pan-cancer hypermethylated canyons, > 90% of which include at least 10 CpGs (Additional file 4: Figure S7a). The mean beta value of 450 K BeadChip probes exhibited an almost perfect accordance (Pearson correlation coefficient ~ 0.90) with the mean methylation level using WGBS (Additional file 4: Figure S7b). Thus, the 450 K BeadChip can be used to reliably measure the methylation level of pan-cancer hypermethylated canyons.

### Correlation between gene expression and locus-specific DNA methylation

Each gene (normalized into 5 kb) and the flanking 1-kb regions were split into 70 bins with a 100-bp window. Spearman’s rank correlation was computed in each bin. For a single sample, Spearman’s rank correlation coefficient was computed between gene expression and the DNA methylation level of the selected gene set at each bin. For pairwise samples (tumor vs normal), Spearman’s rank correlation coefficient was computed between gene expression changes (fold change of RSEM) and the DNA methylation level changes (absolute difference) of the selected gene set at each bin.

### Gene enrichment analyses

We used DAVID [53] version 6.8 for the gene ontology analysis of pan-cancer hypermethylated canyon/cUMRs and we only plotted the GO terms with *p* values < 1.0e-10 with Benjamini correction. Gene enrichment significant levels for homeobox genes, tumor suppressors and oncogenes were calculated by Fisher’s exact test.

### Vector construction

In order to control expression of dCas9-SunTag and scFv-DNMT3A, we acquired doxycycline-inducible open-reading frame expressing vector Pinducer 20 (P20) (Addgene 44,012) from the Thomas F. Westbrook lab and we further exchanged the selection marker of the original P20 vector from neomycin to blasticidin (P20-BSD). The sequence of dCas9-SunTag-2A-BFP and scFv-sfGFP-DNMT3A was then gateway cloned to P20 and P20-BSD, respectively. Catalytic inactive mutation (E756A) of DNMT3A was generated using agilent QuickChange II XL kit based on manufacturer’s instructions in PDONR223-scFv-sgGFP-DNMT3A and then gateway cloned to the P20-BSD vector.

### DNA methylation editing using the dCas9-SunTag-DNMT3A system

Locus-specific DNA methylation of *DLX1* and *POU3F3* gene-bodies was conducted using our dCas9-SunTag-DNMT3A system [36]. In brief, doxycycline-inducible lentiviral particles of dCas9-SunTag-p2A-BFP and scFv-sfGFP-DNMT3A were transduced in a human embryonic kidney cell line (HEK293T). The single clones of idCas9^SunTag^, idCas9^SunTag^ + i^scFv^DNMT3A, and idCas9^SunTag^ + i^scFv^DNMT3A^E756A^ were purified. Lentiviral particles of sgDLX1-puromycin and sgPOU3F3-puromycin were also generated and transduced in previously generated inducible dCas9-SunTag-DNMT3A cells. Transduced cells were treated with 2 μg/mL puromycin for seven consecutive days and cultured in 2 μg/mL doxycycline for another 30 days. SgRNA primers were listed as follows: DLX1-F 5’-CACCGGGCGGACTCGGAGAAGAGCA-3′, DLX1-R 5’-AAACTGCTCTTCTC CGAGTCCGCCC-3′, POU3F3-F: 5’-CACCGCGGCGGCGGGGGCGGCGCAG.

-3′, POU3F3-R: 5’-AAACCTGCGCCGCCCCCGCCGCCGC-3′.

### DNA methylation analysis of targeted regions

Genomic DNA of dCas9-SunTag-DNMT3A-treated cells was extracted by Purelink Mini Kit (Invitrogen) and bisulfite converted by Epitect Bisulfite Kit (Qiagen). Promoter (chr2:172,950,395-172,950,785) and gene-body (chr2:172,951,180-172,951,400) regions of *DLX1*, and promoter (chr2:105,470,350-10,470,850) and gene-body (105,471,850-105,472,350) regions of *POU3F3* were amplified from bisulfite-treated DNA by PCR using the following program. First, samples were heat activated at 95 °C for 5 min, then kept at 95 °C for 30 s, then at 60 °C for 2 min and 30 s and decreased by 0.2 °C every cycle, at 72 °C for 2 min and 30 s and repeated from second step for 40 cycles. Finally, the samples were elongated at 72 °C for 10 min. Bisulfite PCR primers used for promoter (P) and gene-body (E) of *DLX1*: DLX1-P-F 5′- GGGAAGTAGAGGAGAGAAAGTTTTA -3′, DLX1-P-R 5′- CTCTCCTCTCTTCTCTTTCTCTC -3′, DLX1-E-F: 5′- ATTTTTTTT GTAAAGGTAGGAGTTGAG -3′, DLX1-E-R 5′- AACACATACACACAATAACA CCC -3′. Bisulfite PCR products were run in 2% agarose gel electrophoresis, excised, and extracted using a gel extraction kit (Qiagen). DNA concentration of gel-extracted products was measured using qubit dsDNA HS assay kit (Life Technologies) and adjusted to 0.2 ng/μL for Nextera libraries preparation. Nextera libraries preparation was based on the manufacturer’s instructions (Illumina). We used the software of BSMAP [50] to align the paired-end reads to the human genome (hg19) and low-quality sequences were trimmed as the default threshold. High average coverage of each sample was obtained (> 2000×) and their methylation ratios of CpGs with coverage depth > 1000× were computed using the bs-ratio module in software BSMAP.

### Quantitative PCR

Complementary DNA was reverse-transcripted from 1 μg RNA following the manufacturer’s instructions. Primer for qPCR: 18S-F: GTAACCCGTTGAACCCCATT, 18S-R: CCATCCAATCGGTAGTAGCG, qPCR-DLX1-F: ATGCACTGTTTACACTCGGC, qPCR-DLX1-R: GACTGCACCGAACTGATGTAG. qPCR-POU3F3-F: GCGGCTTCTAACCCCTACC, qPCR-POU3F3-R: CCCCTGCATGAAGTCGCTC. qPCR cycle conditions: 3 min at 95 °C; 40 cycles of 10 s for 95 °C,10 s for 55 °C, and 30 s for 72 °C .

## Additional files

Additional file 1: **Table S1.** Data resource of WGBS, RNA-seq, and Infinium HumanMethylation 450 BeadChip data. (XLS 720 kb)

Additional file 2: **Table S2.** Human reference UMRs and annotation. (XLS 8950 kb)

Additional file 3: **Table S3.** Pan-cancer differentially methylated UMRs across 7 tumor types. (XLS 15579 kb)

Additional file 4: **Figures S1–S7.** Supplementary figures. (DOCX 2450 kb)

Additional file 5: **Table S4.** GO functional annotation of pan-cancer hypermethylated, hypomethylated, and conserved Canyon/cUMRs genes. (XLS 698 kb)

Additional file 6: Review history. (DOCX 2465 kb)
