# Supplementary material for: Homeobox oncogene activation by pan-cancer DNA hypermethylation
Source: Genome Biol. 2018 Aug 10;19:108. doi: 10.1186/s13059-018-1492-3 (PMC6085761; doi:10.1186/s13059-018-1492-3)
Supplement: Supplementary file 1 — Table S1. Data resource of WGBS, RNA-seq, and Infinium HumanMethylation 450 BeadChip data. (XLS 720 kb) [file 13059_2018_1492_MOESM1_ESM.xls]

**Supplementary Figures**

**Homeobox oncogene activation by pan-cancer DNA hypermethylation**

**Figure S1. Sequence features of pan-cancer hypermethylated or hypomethylated canyon/cUMRs.** (**a**) CpG observed/Excepted value and GC content of pan-cancer hypermethylated, hypomethylated conserved canyon/cUMRs. Dash lines represent the average CpG Obs/Exp value and GC content of the pan-cancer hypermethylated canyons. (**b**) Genomic distribution of pan-cancer hypermethylated, hypomethylated or stable canyon/cUMRs. The human genome was divided into five categories, i.e. promoter (from upstream 1kb to downstream 500bp of RefSeq gene TSS), gene body (from downstream 500bp of TSS to TTS) and the remaining regions as intergenic region.

**Figure S2. Canyon hypermethylation is associated with increased expression of homeobox oncogenes in multiple tumor types. (a)** Gene expression distributions of pan-cancer hypermethylated or hypomethylated Canyon/cUMRs genes and **(b)** m-homeobox genes in Bladder Urothelial Carcinoma (BLCA), Breast invasive carcinoma (BRCA), Lung adenocarcinoma (LUAD) and Lung squamous cell carcinoma(LUSC), Box plots represent the interquartile range (25–75%), with the median whiskers correspond to 1.5 times the interquartile range.* p-values were calculated by Wilcoxon signed-rank test. n.s represents no significant.

**Figure S3. (a)** Pan-cancer hypermethylated canyons cover from gene promoters to gene bodies. The gene lengths are normalized into 10kb. **(b)** Occupancy of pan-cancer hypermethylated canyons related to the associated genes. Each row represents a pan-cancer hypermethylated canyon related to its associated gene.

**Figure S4. Gene expression of HOXB13 (a) and DLX1 (b) between tumor and normal samples for BLCA, BRCA, LUAD, LUSC, STAD and UCEC**. * p-values were calculated by Wilcoxon signed-rank test. Box plots represent the interquartile range (25–75%), with the median; whiskers correspond to 1.5 times the interquartile range.

**Figure S5. Gene-body canyon hypermethylation by dCas9-SunTag-DNMT3A can directly increase gene** ***POU3F3* expression.** (**a**) Genome browser tracks of gene-body hypermethylated canyons around homeobox gene *POU3F3* across 30 normal and 6 BLCA, 4 LUSC and 5 UCEC tumor samples. (**b**) Gene expression of *POU3F3* between tumor and normal samples for BLCA, LUSC and UCEC. * p-values were calculated by Wilcoxon signed-rank test. Box plots represent the interquartile range (25–75%), with the median; whiskers correspond to 1.5 times the interquartile range. (**c**) CpG DNA methylation level increased at the gene-body of *POU3F3* after adding guide RNA POU3F3 (gray bar) with induction of dCas9^SunTag^ and ^scFv^DNMT3A, yet the methylation level in gene promoter was not affected. CpG DNA methylation level was calculated based on two biological replicates. Error bars represent mean ± s.e.m. of biological replicates. **(d**) Quantitative PCR shows significant increase of gene expression of *POU3F3* with induction of dCas9^SunTag^ and ^scFv^DNMT3A compared to the same cells without induction. P-value was computed by two-sided student’s t test.

**Figure S6.** Percentage of homeobox genes covered by pan-cancer copy number variation (CNV), somatic mutation and hypermethylated canyons. N in the parentheses indicates the number of pan-cancer oncogenic signatures identified from 12 cancer types or the number of pan-cancer hypermethylated canyon genes identified by current manuscript.

**Figure S7. Quantitative comparison between Infinium HumanMethylation 450K assay and whole genome bisulfite sequencing (WGBS). (a)** Boxplot shows the distribution of 450K BeadChip probes in pan-cancer hypermethylated or hypomethylated Canyons/cUMRs. Dash line represents ten 450K BeadChip probes in Canyons/cUMRs. (b) Correlation between mean beta values of 450K BeadChip and mean methylation level of CpGs measured by WGBS between a uterine tumor (Data ID: TCGA-AX-A1CI-01A) and its adjacent normal sample (TCGA-AX-A1CI-11A) as in Fig. 4a. The Pearson correlation coefficient was calculated with p-value <1.0e-16.
