# Supplementary material for: Homeobox oncogene activation by pan-cancer DNA hypermethylation
Source: Genome Biol. 2018 Aug 10;19:108. doi: 10.1186/s13059-018-1492-3 (PMC6085761; doi:10.1186/s13059-018-1492-3)
Supplement: Supplementary file 2 [file 13059_2018_1492_MOESM4_ESM.docx]

## Sheet: Overview

Table S3. Differentially methylated UMRs across seven tumor
Column	Description
chr	chromosome of d-UMR (hg19)
start	start site of d-UMR
end	end site of d-UMR
length 	length of d-UMR
d-UMR category	Category of d-UMR
Differetial_methylation_level(Tumor-Normal) 	Differentail methylation level (Mean_methylation_level_Tumor-Mean_methylation_level_Tumor)
p_value	P vlaue of signficant difference 
FDR	p vlaue justed by FDR
Pattern_d-UMR	The patterns of d-UMR( Hypermethylated : Hypermethylated d-UMR; Hypomethylated : Hypomethylated d-UMR; Non: Others)
##versions of human genome references (hg19)

## Sheet: Normal_conserved_UMR-pattern.tx

chr	start	end	length (bp)	type	Bladder Urothelial Carcinoma(BLCA)				Breast invasive carcinoma(BRCA)				Glioblastoma multiforme(GBM)				Lung adenocarcinoma(LUAD)				Lung squamous cell carcinoma(LUSC)				Stomach adenocarcinoma(STAD)				Uterine Corpus Endometrial Carcinoma(UCEC)				Pan-cancer				Closest genes
					Differetial_methylation_level(Tumor-Normal) 	p_value	FDR	Pattern_DMU	Differetial_methylation_level(Tumor-Normal) 	p_value	FDR	Pattern_DMU	Differetial_methylation_level(Tumor-Normal) 	p_value	FDR	Pattern_DMU	Differetial_methylation_level(Tumor-Normal) 	p_value	FDR	Pattern_DMU	Differetial_methylation_level(Tumor-Normal) 	p_value	FDR	Pattern_DMU	Differetial_methylation_level(Tumor-Normal) 	p_value	FDR	Pattern_DMU	Differetial_methylation_level(Tumor-Normal) 	p_value	FDR	Pattern_DMU	Differetial_methylation_level(Tumor-Normal) 	p_value	FDR	Pattern_DMU	gene_name2	Distance to DM(bp)
chr1	521380	521683	303	cUMR	-0.045288839	0.036227675	0.042721532	Non	-0.037063454	0.280363833	0.353121853	Non	-0.050601336	0.00599612	0.011353382	Non	-0.031060974	0.381955179	0.465699187	Non	-0.003392345	0.377558755	0.427112256	Non	-0.020434727	0.796985194	0.809877055	Non	0.088517943	0.024322481	0.034555463	Non	-0.016246124	0.015590464	0.017127051	Non	MIR6723	46022
chr1	713405	715504	2099	cUMR	-0.119133333	0.000000000000452	0.00000000000656	Hypomethylated	-0.041433333	0.006246162	0.015163587	Non	0.0327	0.049570588	0.0726445	Non	-0.061833333	0.0000307	0.000230732	Non	-0.068883333	0.00000452	0.0000214	Non	-0.097383333	0.0000000274	0.000000151	Non	-0.120433333	0.000000000000538	0.0000000000142	Hypomethylated	-0.06577619	0.000000000000583	0.00000000000479	Non	LOC100288069	0
chr1	761448	763814	2366	cUMR	-0.016966667	0.0353916	0.041801048	Non	-0.0101	0.14301098	0.203539991	Non	0.012366667	0.444078691	0.494291376	Non	-0.0027	0.129570898	0.196364978	Non	0.0722	0.000155332	0.000464012	Non	0.00445	0.077311802	0.095627244	Non	0.0029	0.93258328	0.938974039	Non	0.006557143	0.020009274	0.021789665	Non	LINC00115	0
chr1	804763	805662	899	cUMR	-0.053966667	0.0000332	0.000068	Non	0.0203	0.102315021	0.15511073	Non	0.2092	0.000000000019	0.000000000362	Hypermethylated	0.0023	0.771208952	0.812976943	Non	0.0267	0.173136632	0.215263985	Non	0.0092	0.460845408	0.489179006	Non	0.0393	0.093166184	0.116935742	Non	0.039557143	0.0000000741	0.000000171	Non	FAM41C	0
chr1	839395	841144	1749	cUMR	-0.045566667	0.00000401	0.0000101	Non	-0.028933333	0.190475991	0.257801615	Non	-0.046733333	0.007527716	0.013843323	Non	-0.052933333	0.01427798	0.033160896	Non	-0.056483333	0.006065219	0.011481512	Non	-0.077233333	0.00000699	0.0000221	Non	0.010666667	0.014987625	0.02232077	Non	-0.04127619	0.00000481	0.00000803	Non	LOC100130417	11054
chr1	845125	846924	1799	cUMR	-0.148566667	0.000011	0.0000252	Hypomethylated	0.132766667	0.0000459	0.000220025	Hypermethylated	-0.038566667	0.0000953	0.00029984	Non	0.014766667	0.490060684	0.567385755	Non	-0.058233333	0.008709669	0.015704122	Non	0.031766667	0.282592366	0.312567624	Non	0.074966667	0.021754165	0.031223991	Non	-0.003319048	0.00000401	0.00000678	Non	LOC100130417	5274
chr1	858375	861924	3549	Canyon	-0.025466667	0.003734753	0.005157006	Non	0.022966667	0.127535345	0.18555846	Non	0.0002	0.013835245	0.023663975	Non	-0.013833333	0.295869358	0.380725709	Non	0.004366667	0.322508078	0.371856443	Non	0.025116667	0.043935868	0.057239906	Non	0.011566667	0.334392074	0.371587872	Non	0.001995238	0.004215562	0.004851782	Non	SAMD11	0
chr1	874805	878916	4111	Canyon	-0.017166667	0.088522116	0.099228112	Non	0.0307	0.030117023	0.056648966	Non	0.006166667	0.730710628	0.761884074	Non	-0.0113	0.312762067	0.397916704	Non	0.01525	0.071168844	0.098934341	Non	-0.00925	0.640283339	0.661928267	Non	0.0511	0.001344184	0.002492646	Non	0.008871429	0.024187081	0.026155999	Non	SAMD11	0
chr1	893679	896828	3149	cUMR	-0.0145	0.355133038	0.370187557	Non	0.007266667	0.832020097	0.860812686	Non	-0.012	0.002484795	0.00524285	Non	-0.010333333	0.015466061	0.035387376	Non	-0.007333333	0.822459233	0.844195257	Non	-0.017333333	0.000407345	0.000840069	Non	0.026266667	0.003891272	0.006567032	Non	-0.004047619	0.000298862	0.000388226	Non	KLHL17	0
chr1	901379	902928	1549	cUMR	-0.051166667	0.000537655	0.000862209	Non	-0.041133333	0.000990155	0.003157305	Non	0.053833333	0.02301021	0.037110019	Non	-0.038733333	0.031077291	0.062392783	Non	-0.047833333	0.010916333	0.019184786	Non	-0.051833333	0.001717691	0.003067505	Non	-0.011733333	0.744159139	0.765409938	Non	-0.024019048	0.002113069	0.002501141	Non	PLEKHN1	0
chr1	905786	906535	749	cUMR	0.2025	0.0000075	0.0000178	Hypermethylated	0.531933333	0.00000000000287	0.000000000218	Hypermethylated	0.071666667	0.022417595	0.036270928	Non	0.300333333	0.000000405	0.00000667	Hypermethylated	0.149583333	0.0000437	0.000153848	Hypermethylated	0.520833333	0.0000000000326	0.000000000368	Hypermethylated	0.391933333	0.00000000553	0.0000000374	Hypermethylated	0.298504762	0.00000000000157	0.0000000000114	Hypermethylated	PLEKHN1	0
chr1	910855	913966	3111	cUMR	-0.3759	0.00000000000049	0.00000000000702	Hypomethylated	-0.200733333	0.0000745	0.000336193	Hypomethylated	-0.015233333	0.0000124	0.0000493	Non	-0.202733333	0.000104535	0.00062077	Hypomethylated	-0.297733333	0.0000000315	0.000000306	Hypomethylated	-0.292733333	0.000000337	0.00000146	Hypomethylated	-0.300133333	0.0000000241	0.000000138	Hypomethylated	-0.235047619	0.000000000000926	0.00000000000717	Hypomethylated	PERM1	0
chr1	931027	932326	1299	cUMR	-0.159366667	0.0000766	0.000145184	Hypomethylated	-0.078166667	0.082077916	0.129634367	Non	0.1768	0.000156136	0.000462801	Hypermethylated	-0.150166667	0.000945996	0.003753145	Non	-0.218116667	0.00000347	0.0000169	Hypomethylated	-0.182616667	0.000853072	0.001631923	Non	-0.008166667	0.526465735	0.559285704	Non	-0.076595238	0.00000307	0.00000528	Non	HES4	2016
chr1	933521	937820	4299	Canyon	-0.0185	0.00441008	0.006019149	Non	0.007466667	0.632072228	0.685558107	Non	0.062	0.000512352	0.001306248	Non	0.021266667	0.16605197	0.239662839	Non	-0.005583333	0.095455017	0.127807256	Non	0.030916667	0.135232777	0.159270782	Non	0.058266667	0.002634698	0.004601412	Non	0.022780952	0.000594911	0.000745549	Non	HES4	0
chr1	948228	949927	1699	cUMR	-0.223033333	0.00000000702	0.0000000348	Hypomethylated	-0.1329	0.000197348	0.000783441	Hypomethylated	0.004633333	0.037189756	0.056510419	Non	-0.0979	0.000124734	0.000719101	Non	-0.05295	0.001250841	0.002866653	Non	-0.15795	0.0000746	0.000183685	Hypomethylated	-0.1159	0.0000578	0.000144807	Hypomethylated	-0.111071429	0.00000000691	0.0000000199	Hypomethylated	ISG15	0
chr1	954442	957191	2749	cUMR	-0.055233333	0.0002581	0.000439182	Non	-0.040233333	0.006120644	0.01490413	Non	-0.0179	0.524297016	0.570914873	Non	-0.046833333	0.011875868	0.028490452	Non	-0.026983333	0.362611007	0.412315389	Non	-0.048233333	0.008096496	0.01236748	Non	-0.031233333	0.070981179	0.091442796	Non	-0.038033333	0.0000294	0.0000437	Non	AGRN	0
chr1	975111	976610	1499	cUMR	0.216633333	0.00000335	0.00000858	Hypermethylated	0.100233333	0.00921347	0.021094251	Non	0.319133333	0.00000000594	0.0000000596	Hypermethylated	0.227433333	0.0000071	0.0000684	Hypermethylated	0.066633333	0.177295467	0.219856233	Non	0.091633333	0.021637112	0.030085461	Non	0.255633333	0.0000000536	0.000000279	Hypermethylated	0.193261905	0.00000000042	0.0000000016	Hypermethylated	AGRN	0
chr1	993219	996318	3099	cUMR	-0.1115	0.0000000339	0.00000014	Hypomethylated	-0.014466667	0.580121285	0.639223678	Non	-0.005	0.156755537	0.199561252	Non	-0.059666667	0.011162847	0.027156938	Non	-0.070916667	0.000377927	0.001009677	Non	-0.076166667	0.000938961	0.001779139	Non	-0.080466667	0.000141018	0.000323986	Non	-0.058866667	0.000000195	0.000000415	Non	AGRN	-1721
chr1	999006	1000555	1549	cUMR	-0.148066667	0.00000229	0.00000612	Hypomethylated	-0.1035	0.002207163	0.006280082	Non	0.111766667	0.001869683	0.004085937	Non	-0.1055	0.004259469	0.012545575	Non	-0.08515	0.044500948	0.065465102	Non	-0.1279	0.0008423	0.001612981	Non	-0.0729	0.060662333	0.079264658	Non	-0.070842857	0.0000277	0.0000413	Non	RNF223	6571
chr1	1002673	1005522	2849	cUMR	-0.1293	0.00000000233	0.000000013	Hypomethylated	-0.079033333	0.00000355	0.0000232	Non	0.048366667	0.00000018	0.00000119	Non	-0.153033333	0.00000000027	0.0000000224	Hypomethylated	-0.128883333	0.00000000202	0.0000000315	Hypomethylated	-0.188883333	0.0000000000361	0.000000000403	Hypomethylated	-0.172033333	0.0000000000159	0.000000000245	Hypomethylated	-0.107919048	0.0000000000000161	0.000000000000214	Hypomethylated	RNF223	1604
chr1	1014026	1016425	2399	cUMR	-0.1028	0.0000892	0.000166873	Hypomethylated	-0.066366667	0.095505028	0.146417144	Non	-0.062133333	0.001850905	0.004051514	Non	-0.117166667	0.000173899	0.00093918	Hypomethylated	-0.046716667	0.341582788	0.391154815	Non	-0.08196666699999999	0.040567882	0.053220361	Non	-0.127966667	0.0000544	0.000137147	Hypomethylated	-0.087480952	0.00000117	0.00000216	Non	C1orf159	773
chr1	1050304	1052703	2399	cUMR	-0.018033333	0.00000000238	0.0000000133	Non	-0.018433333	0.000133036	0.000556653	Non	-0.0072	0.019577336	0.032172302	Non	-0.000633333	0.180641448	0.256300052	Non	-0.011033333	0.03867368	0.057968423	Non	-0.016033333	0.00249232	0.00429122	Non	-0.007633333	0.004760928	0.007870966	Non	-0.011233333	0.000000128	0.000000283	Non	C1orf159	0
chr1	1068757	1069056	299	cUMR	-0.1398382	0.397651698	0.412297161	Non	-0.049939267	0.313738918	0.386847807	Non	0.24099552	0.051448163	0.075034669	Non	-0.075137234	0.925378971	0.940641263	Non	-0.049088009	0.743334274	0.773339781	Non	-0.359087307	0.000480346	0.00097444	Hypomethylated	-0.226938038	0.315218416	0.352181711	Non	-0.079595337	0.810619136	0.812642481	Non	LINC01342	3341
chr1	1092435	1094184	1749	cUMR	-0.071833333	0.00234578	0.003353609	Non	0.004766667	0.989539395	0.991349553	Non	0.082666667	0.001402154	0.003174475	Non	-0.056833333	0.036024406	0.070151898	Non	-0.023833333	0.430130681	0.479425663	Non	-0.093583333	0.002572205	0.004414572	Non	0.008766667	0.001629916	0.00295875	Non	-0.017747619	0.004055962	0.004675241	Non	MIR200B	8300
chr1	1136147	1137211	1064	cUMR	-0.019166667	0.041418462	0.048477444	Non	0.018766667	0.227358279	0.298315669	Non	0.018	0.159595205	0.202865558	Non	-0.0000333	0.091172225	0.148300255	Non	0.058916667	0.0000407	0.000144576	Non	0.133916667	0.000000000307	0.00000000265	Hypermethylated	0.038966667	0.002433544	0.00427489	Non	0.030080952	0.000000164	0.000000354	Non	TNFRSF18	1677
chr1	1141326	1144629	3303	cUMR	-0.136833333	0.0000078	0.0000184	Hypomethylated	-0.041133333	0.329749508	0.402307422	Non	0.203833333	0.000000821	0.00000449	Hypermethylated	-0.077533333	0.035809174	0.069835931	Non	-0.086583333	0.008139603	0.014794701	Non	-0.095333333	0.009893715	0.014839033	Non	-0.036533333	0.010911731	0.01669497	Non	-0.03147619	0.00000387	0.00000656	Non	TNFRSF18	0
chr1	1166086	1168139	2053	cUMR	-0.006133333	0.085782594	0.096300613	Non	-0.001633333	0.31588168	0.388875283	Non	0.008533333	0.027703246	0.043610716	Non	0.022766667	0.008502463	0.021866846	Non	0.002366667	0.642072882	0.680805751	Non	0.007616667	0.472902893	0.500989753	Non	0.013366667	0.120531014	0.147712101	Non	0.006480952	0.011836017	0.013122209	Non	B3GALT6	0
chr1	1181565	1182914	1349	cUMR	0.075566667	0.00000335	0.00000859	Non	0.3669	0.0000000000433	0.0000000017	Hypermethylated	0.301566667	0.0000000000166	0.000000000326	Hypermethylated	0.1287	0.0000615	0.000405069	Hypermethylated	0.0044	0.144998679	0.184272659	Non	0.3924	0.00000000014	0.00000000131	Hypermethylated	0.1161	0.00000045	0.00000185	Hypermethylated	0.197385714	0.0000000000015	0.0000000000109	Hypermethylated	FAM132A	0
chr1	1207640	1210021	2381	cUMR	-0.0544	0.0000869	0.0001629	Non	-0.030433333	0.111151658	0.16595957	Non	-0.0314	0.053394146	0.077446258	Non	-0.027633333	0.170429609	0.244642346	Non	-0.026983333	0.253655671	0.30108336	Non	-0.037233333	0.064508283	0.080998364	Non	-0.037233333	0.037761784	0.051529262	Non	-0.035661905	0.0000438	0.0000635	Non	UBE2J2	0
chr1	1239470	1245382	5912	Canyon	-0.05	0.008161457	0.010638546	Non	-0.0042	0.308649938	0.381642561	Non	-0.079833333	0.0000285	0.000103492	Non	0.0054	0.964263943	0.971764491	Non	-0.08175	0.0002574	0.000722494	Non	-0.04675	0.061413568	0.077463709	Non	0.0624	0.006657572	0.010695437	Non	-0.027857143	0.001232423	0.001493835	Non	ACAP3	0
chr1	1259170	1261519	2349	cUMR	-0.046466667	0.000231662	0.00039887	Non	-0.038833333	0.002257678	0.006405659	Non	-0.001466667	0.970881445	0.974959495	Non	-0.025033333	0.087298582	0.143291994	Non	-0.037633333	0.008754291	0.015776317	Non	-0.038133333	0.010805601	0.016058796	Non	-0.025833333	0.104580012	0.129827922	Non	-0.029690476	0.0000707	0.0000994	Non	CPSF3L	0
chr1	1282928	1285529	2601	cUMR	-0.0293	0.0000741	0.000140919	Non	-0.0042	0.815122461	0.846448509	Non	-0.003133333	0.548515414	0.59399292	Non	-0.0122	0.367873267	0.452355123	Non	-0.0293	0.004995358	0.009665774	Non	-0.02405	0.041232639	0.054039492	Non	-0.0186	0.139931594	0.169046009	Non	-0.016657143	0.003114609	0.003628691	Non	DVL1	0
chr1	1290356	1292520	2164	cUMR	-0.098966667	0.000133287	0.000240433	Non	0.116866667	0.0000115	0.0000652	Hypermethylated	0.130533333	0.001655627	0.003672509	Non	0.140266667	0.000131766	0.000751196	Hypermethylated	-0.086133333	0.022290349	0.035838859	Non	0.063866667	0.036872027	0.048861265	Non	0.029066667	0.00474975	0.007853563	Non	0.043752381	0.00000165	0.00000298	Non	MXRA8	0
chr1	1293117	1294366	1249	cUMR	0.086833333	0.012118135	0.01536477	Non	0.1116	0.041658617	0.073801477	Non	0.118	0.009529204	0.017056551	Non	-0.0102	0.231755777	0.313333186	Non	0.081	0.004146082	0.008189531	Non	0.034	0.559926179	0.584851158	Non	0.1446	0.007429591	0.01180049	Non	0.0834	0.000498754	0.000631149	Non	MXRA8	0
chr1	1297415	1297741	326	cUMR	-0.016866667	0.618978332	0.629957761	Non	0.168433333	0.005234261	0.013104413	Non	0.102966667	0.000672838	0.001659235	Non	0.201433333	0.000150015	0.000831431	Hypermethylated	0.083383333	0.001343052	0.00305473	Non	0.065633333	0.019710562	0.027626657	Non	0.258633333	0.0000465	0.000119004	Hypermethylated	0.12157619	0.0000526	0.0000753	Hypermethylated	MXRA8	0
chr1	1307237	1307936	699	cUMR	0.049066667	0.28544572	0.300667495	Non	0.1057	0.020826654	0.041655037	Non	0.0014	0.9095674	0.920994798	Non	-0.0599	0.077535523	0.130460204	Non	0.0574	0.277368728	0.325903916	Non	-0.04885	0.035477375	0.04716876	Non	0.3581	0.0000000168	0.000000101	Hypermethylated	0.067328571	0.000223384	0.000294787	Non	AURKAIP1	1174
chr1	1309506	1311255	1749	cUMR	-0.030133333	0.00000232	0.00000618	Non	0.0081	0.306470646	0.379497593	Non	-0.006633333	0.000361315	0.000962097	Non	0.0101	0.02167088	0.046667284	Non	-0.0358	0.0000405	0.00014387	Non	-0.04055	0.000000799	0.00000317	Non	-0.0021	0.013479082	0.020267829	Non	-0.012728571	0.000000214	0.000000452	Non	AURKAIP1	0
chr1	1333636	1335985	2349	cUMR	-0.023233333	0.00000308	0.00000796	Non	-0.013633333	0.031270917	0.058397272	Non	-0.011066667	0.044940377	0.066644505	Non	-0.014033333	0.023720093	0.050198191	Non	-0.015733333	0.016807785	0.028021143	Non	-0.021233333	0.000394635	0.000816231	Non	-0.016033333	0.005081563	0.008359755	Non	-0.016347619	0.000000146	0.000000318	Non	CCNL2	0
chr1	1341723	1343570	1847	cUMR	-0.016133333	0.0000000912	0.00000034	Non	-0.007366667	0.043038187	0.075893977	Non	-0.011966667	0.012368124	0.021426819	Non	0.006033333	0.305257071	0.390388203	Non	-0.024466667	0.0000165	0.000066	Non	-0.034216667	0.0000000151	0.0000000876	Non	-0.014166667	0.000207557	0.000457742	Non	-0.013738095	0.00000000859	0.0000000242	Non	MRPL20	0
chr1	1355055	1357304	2249	cUMR	-0.187633333	0.000000387	0.00000124	Hypomethylated	0.067133333	0.027720801	0.052889006	Non	0.033866667	0.004876325	0.009454475	Non	-0.082666667	0.010223663	0.025362346	Non	-0.209466667	0.0000002	0.00000148	Hypomethylated	0.125283333	0.013213358	0.019237283	Non	0.174133333	0.0000731	0.000179094	Hypermethylated	-0.01332381	0.00000197	0.0000035	Non	ANKRD65	0
chr1	1361395	1363644	2249	cUMR	-0.060266667	0.059463785	0.068090202	Non	0.120266667	0.001347614	0.004098631	Non	-0.000933333	0.01662259	0.027813579	Non	-0.092133333	0.017871742	0.039904385	Non	-0.000933333	0.098967957	0.131895246	Non	0.073816667	0.01417089	0.020508685	Non	0.134466667	0.001812118	0.003259054	Non	0.021066667	0.000603819	0.000756045	Non	TMEM88B	0
chr1	1405734	1408533	2799	cUMR	-0.071666667	0.0000000717	0.000000274	Non	-0.057566667	0.00000482	0.0000303	Non	-0.020166667	0.008203639	0.014934751	Non	-0.037766667	0.020035813	0.043769652	Non	-0.052916667	0.001214399	0.002794822	Non	-0.063666667	0.0000595	0.000150002	Non	-0.056766667	0.000094	0.000224846	Non	-0.050795238	0.00000000287	0.00000000901	Non	ATAD3B	0
chr1	1446119	1449768	3649	Canyon	-0.070333333	0.00000000441	0.0000000229	Non	-0.035933333	0.006278598	0.015228535	Non	-0.006333333	0.000117547	0.000359701	Non	-0.034333333	0.008946556	0.022751594	Non	-0.079583333	0.0000000617	0.000000537	Non	-0.100333333	0.000000000101	0.000000000993	Hypomethylated	-0.051533333	0.00000163	0.00000583	Non	-0.051104762	0.0000000000248	0.000000000127	Non	ATAD3A	0
chr1	1474870	1476469	1599	cUMR	0.446633333	0.000000000135	0.00000000105	Hypermethylated	0.499566667	0.0000000000969	0.0000000033	Hypermethylated	-0.025533333	0.369335422	0.421632716	Non	0.055966667	0.265868733	0.349664614	Non	0.215716667	0.0000117	0.0000488	Hypermethylated	0.573966667	0.00000000000824	0.000000000113	Hypermethylated	0.291166667	0.000000187	0.000000845	Hypermethylated	0.283395238	0.00000000000934	0.0000000000542	Hypermethylated	TMEM240	0
chr1	1508937	1511139	2202	cUMR	-0.0156	0.000000249	0.000000835	Non	-0.0138	0.013050183	0.028130446	Non	0.002733333	0.016558178	0.027719276	Non	0.0104	0.033242487	0.065791513	Non	-0.02185	0.0000198	0.0000774	Non	-0.0221	0.0000534	0.000136243	Non	-0.018	0.000573224	0.001151226	Non	-0.010285714	0.000000301	0.000000619	Non	SSU72	0
chr1	1521444	1521893	449	cUMR	-0.168066667	0.0000000137	0.0000000631	Hypomethylated	-0.074133333	0.0000159	0.0000868	Non	0.080433333	0.000000000163	0.00000000244	Non	-0.067133333	0.0000182	0.000149812	Non	0.004516667	0.000530615	0.001364504	Non	-0.053483333	0.036459857	0.048354961	Non	-0.092533333	0.0000000597	0.000000308	Non	-0.054019048	0.000000000028	0.000000000142	Non	SSU72	-11183
chr1	1534483	1536282	1799	cUMR	0.004266667	0.100075047	0.111555578	Non	0.067433333	0.001979412	0.005713068	Non	0.1051	0.000143307	0.000428623	Hypermethylated	0.002833333	0.935309873	0.948335815	Non	0.031433333	0.323687302	0.373019779	Non	0.147933333	0.0000282	0.000077	Hypermethylated	0.094433333	0.00071377	0.001403085	Non	0.06277619	0.0000199	0.0000304	Non	C1orf233	0
chr1	1549288	1552287	2999	cUMR	-0.051866667	0.00000967	0.0000224	Non	-0.025233333	0.076675605	0.122563984	Non	-0.007866667	0.698568356	0.732905231	Non	-0.035033333	0.017182384	0.038643127	Non	-0.025283333	0.172767093	0.214837784	Non	-0.033533333	0.005829394	0.009210985	Non	-0.031433333	0.031567391	0.043780814	Non	-0.030061905	0.00006	0.0000853	Non	MIB2	0
chr1	1565486	1567937	2451	cUMR	0.262833333	0.00000234	0.00000624	Hypermethylated	0.321	0.00000356	0.0000233	Hypermethylated	0.335166667	0.00000172	0.00000865	Hypermethylated	0.2422	0.000396882	0.001854743	Non	0.21575	0.000181516	0.000532333	Hypermethylated	0.27175	0.0000163	0.000047	Hypermethylated	0.4404	0.0000000198	0.000000116	Hypermethylated	0.3016	0.0000000000407	0.000000000198	Hypermethylated	MMP23B	0
chr1	1589395	1591294	1899	cUMR	-0.088133333	0.0000113	0.0000257	Non	-0.064966667	0.001906333	0.005527907	Non	-0.036466667	0.025402395	0.040400404	Non	-0.036966667	0.051873531	0.09472216	Non	-0.083966667	0.000246362	0.000695423	Non	-0.080966667	0.000668998	0.001313683	Non	-0.081766667	0.0000754	0.000184198	Non	-0.066452381	0.0000000614	0.000000145	Non	CDK11B	0
chr1	1676241	1678890	2649	cUMR	-0.012233333	0.313537343	0.328633939	Non	0.007066667	0.530845189	0.594330554	Non	0.0481	0.029080075	0.045516474	Non	-0.016733333	0.166523306	0.240076496	Non	0.038516667	0.127356888	0.164480749	Non	-0.016233333	0.004417106	0.007170321	Non	-0.002533333	0.66977629	0.695973499	Non	0.006952381	0.53162153	0.536025758	Non	SLC35E2	0
chr1	1708576	1712034	3458	cUMR	-0.0332	0.011662107	0.014831826	Non	-0.0245	0.037286613	0.067383795	Non	0.016633333	0.181174333	0.226355198	Non	-0.0361	0.010013434	0.02494611	Non	-0.02745	0.086856123	0.117660288	Non	-0.0562	0.000061	0.000153617	Non	0.0135	0.247884466	0.283363731	Non	-0.019128571	0.000918692	0.001127624	Non	NADK	0
chr1	1820369	1824268	3899	Canyon	-0.016633333	0.095720826	0.106904596	Non	0.001966667	0.974181792	0.978690013	Non	0.010866667	0.04358839	0.064875118	Non	-0.007233333	0.754268952	0.798961459	Non	-0.022133333	0.098794222	0.131700125	Non	-0.011633333	0.4943384	0.52190873	Non	0.023366667	0.123918723	0.151463201	Non	-0.002261905	0.52008794	0.524550252	Non	GNB1	0
chr1	1837483	1838082	599	cUMR	-0.0317	0.003350704	0.004665468	Non	0.038666667	0.115950829	0.171720676	Non	0.0443	0.046002954	0.067973462	Non	0.013066667	0.214274935	0.294347532	Non	0.070466667	0.000273791	0.000762224	Non	-0.046033333	0.000075	0.000184728	Non	0.204666667	0.00000000856	0.000000055	Hypermethylated	0.041580952	0.0000011	0.00000205	Non	CALML6	8184
chr1	1839815	1841314	1499	cUMR	-0.020966667	0.156668724	0.170247245	Non	0.0002	0.018191818	0.037225564	Non	0.054366667	0.001795656	0.003942754	Non	0.0076	0.705136108	0.756292057	Non	0.05945	0.004934674	0.009562989	Non	-0.0168	0.010065606	0.015066834	Non	0.0114	0.357775763	0.394787049	Non	0.013342857	0.023943378	0.025902919	Non	CALML6	4952
chr1	1850006	1851407	1401	cUMR	-0.042733333	0.00000268	0.00000705	Non	0.030833333	0.018156523	0.03717289	Non	0.1671	0.000000000197	0.00000000288	Hypermethylated	-0.010166667	0.565379817	0.634613761	Non	-0.005066667	0.39308622	0.442685845	Non	-0.030566667	0.012448124	0.018239991	Non	-0.016566667	0.072636276	0.093366249	Non	0.017833333	0.000000283	0.000000584	Non	TMEM52	0
chr1	1855020	1855319	299	cUMR	-0.348505506	0.038041578	0.044751083	Non	0.04922962	0.074464899	0.119729144	Non	-0.027670869	0.972664745	0.976473714	Non	-0.153971581	0.521775303	0.595968398	Non	-0.348670853	0.118063174	0.153963221	Non	-0.318173082	0.112131413	0.134197503	Non	-0.193571182	0.282861728	0.319287777	Non	-0.183314739	0.14358401	0.148278872	Non	TMEM52	-4281
chr1	1875079	1876028	949	cUMR	-0.126433333	0.0000565	0.000110333	Hypomethylated	-0.075466667	0.0000557	0.00026067	Non	0.008066667	0.037652235	0.057112329	Non	-0.098066667	0.003759325	0.0113382	Non	-0.067766667	0.119632041	0.155764692	Non	-0.063766667	0.117368287	0.139975303	Non	-0.101866667	0.003438402	0.00586229	Non	-0.074666667	0.000058	0.0000826	Non	KIAA1751	8724
chr1	1891042	1892641	1599	cUMR	-0.1625	0.0000021	0.00000566	Hypomethylated	-0.012566667	0.226426052	0.297323774	Non	-0.0085	0.019768636	0.032444636	Non	-0.161766667	0.0000252	0.000195961	Hypomethylated	-0.038666667	0.183822187	0.226991907	Non	-0.072916667	0.155972619	0.181269745	Non	-0.139966667	0.0000892	0.000214333	Hypomethylated	-0.086966667	0.00000134	0.00000246	Non	KIAA1751	0
chr1	1934815	1935664	849	cUMR	-0.070866667	0.0000101	0.0000232	Non	0.299066667	0.000000000093	0.00000000319	Hypermethylated	-0.029866667	0.07419503	0.103324505	Non	0.127866667	0.000000432	0.00000703	Hypermethylated	0.189466667	0.0000000179	0.00000019	Hypermethylated	0.120716667	0.00000567	0.0000184	Hypermethylated	0.062466667	0.000100664	0.000239281	Non	0.088095238	0.000000000104	0.000000000457	Non	KIAA1751	0
chr1	1949883	1951632	1749	cUMR	-0.030533333	0.024737269	0.029910623	Non	0.119033333	0.0000152	0.0000834	Hypermethylated	0.095466667	0.0000191	0.0000723	Non	-0.013966667	0.145683665	0.21558553	Non	0.071883333	0.008071297	0.014689382	Non	0.282883333	0.0000000000344	0.000000000386	Hypermethylated	0.034233333	0.002092571	0.003718754	Non	0.07157619	0.00000000969	0.000000027	Non	GABRD	0
chr1	1976073	1976822	749	cUMR	-0.0238	0.000333031	0.000554967	Non	0.012666667	0.438765239	0.509047952	Non	-0.009633333000000001	0.009020875	0.016227608	Non	-0.006733333	0.475273113	0.554174843	Non	-0.010633333	0.599053979	0.640717358	Non	0.007866667	0.760523914	0.77538946	Non	0.035066667	0.002373774	0.004176899	Non	-0.000190476	0.00079959	0.000987622	Non	PRKCZ	5087
chr1	1980738	1983187	2449	cUMR	-0.045333333	0.00000984	0.0000227	Non	-0.0168	0.325535012	0.398173554	Non	0.183666667	0.0000000000687	0.00000000113	Hypermethylated	-0.0492	0.0000413	0.00029356	Non	-0.02675	0.024090282	0.03830854	Non	-0.03225	0.014312114	0.020679538	Non	0.0046	0.284112892	0.32050488	Non	0.0082	0.0000000262	0.0000000667	Non	PRKCZ	0
chr1	2124986	2127171	2185	cUMR	-0.104066667	0.000110415	0.000202603	Hypomethylated	-0.0156	0.723357406	0.767096925	Non	0.0406	0.517574368	0.564571208	Non	-0.0758	0.08009166	0.133891697	Non	-0.10515	0.010725455	0.01888238	Non	-0.1419	0.000214767	0.000473127	Hypomethylated	-0.0826	0.002363493	0.004160311	Non	-0.063971429	0.000166925	0.000224098	Non	C1orf86	0
chr1	2135693	2137311	1618	cUMR	-0.0191	0.001045122	0.001591266	Non	0.100666667	0.0000943	0.000412414	Hypermethylated	0.234566667	0.000000000273	0.00000000381	Hypermethylated	-0.005933333	0.706397254	0.757340547	Non	0.091816667	0.000270876	0.000754807	Non	-0.036933333	0.085414841	0.104680203	Non	0.092666667	0.000539419	0.001088961	Non	0.069980952	0.0000000931	0.000000211	Non	C1orf86	0
chr1	2158213	2164662	6449	Canyon	-0.029833333	0.199943633	0.214372739	Non	-0.020466667	0.553976558	0.61593564	Non	-0.025	0.34779055	0.400413582	Non	-0.001866667	0.991056159	0.993172112	Non	-0.015166667	0.004637638	0.009055081	Non	-0.013166667	0.679252002	0.698647606	Non	0.002333333	0.898326443	0.907865844	Non	-0.015495238	0.289038719	0.294364595	Non	SKI	0
chr1	2245721	2247020	1299	cUMR	-0.0301	0.001256612	0.001885426	Non	0.007633333	0.400461198	0.472617959	Non	0.1369	0.000000000555	0.0000000071	Hypermethylated	-0.018166667	0.163570236	0.236768113	Non	0.050233333	0.003936115	0.007824822	Non	0.034483333	0.000752808	0.001460053	Non	0.041633333	0.0000108	0.0000318	Non	0.032433333	0.0000000285	0.000000072	Non	SKI	-4070
chr1	2313170	2313619	449	cUMR	-0.0503	0.005162139	0.006962012	Non	0.035066667	0.352057992	0.424228704	Non	-0.071633333	0.011772801	0.020509765	Non	0.021666667	0.011210966	0.027222466	Non	-0.036633333	0.126825751	0.163882618	Non	-0.092383333	0.005043207	0.008074627	Non	0.068266667	0.011850063	0.017994651	Non	-0.017790476	0.000546444	0.000687815	Non	MORN1	0
chr1	2314120	2314419	299	cUMR	-0.1857	0.00000364	0.00000925	Hypomethylated	-0.063366667	0.199919664	0.268771711	Non	-0.079033333	0.0000053	0.0000235	Non	-0.031566667	0.561525223	0.631286906	Non	-0.088116667	0.077340907	0.106336576	Non	-0.237866667	0.000000075	0.000000377	Hypomethylated	-0.118366667	0.000101767	0.000241737	Hypomethylated	-0.113109524	0.0000000486	0.000000117	Hypomethylated	MORN1	0
chr1	2321848	2324247	2399	cUMR	-0.044633333	0.000000294	0.000000967	Non	-0.0357	0.0000633	0.000291261	Non	-0.0048	0.864812859	0.882500964	Non	-0.0159	0.224621833	0.305453243	Non	-0.0323	0.001995322	0.004311628	Non	-0.03555	0.001119835	0.002085133	Non	-0.0353	0.000366767	0.000765625	Non	-0.028642857	0.00000024	0.000000502	Non	MORN1	0
chr1	2343165	2348614	5449	Canyon	-0.1014	0.000391751	0.000644573	Hypomethylated	-0.0841	0.010474736	0.023458152	Non	0.059266667	0.005923588	0.011235041	Non	-0.0965	0.001256343	0.004729784	Non	-0.0844	0.017763478	0.02940204	Non	-0.12265	0.000139594	0.000322526	Hypomethylated	-0.0389	0.422432884	0.458263226	Non	-0.062242857	0.000157703	0.000212249	Non	PEX10	0
chr1	2374341	2375761	1420	cUMR	0.418266667	0.000000000000251	0.00000000000392	Hypermethylated	0.037433333	0.173336606	0.23858412	Non	-0.015566667	0.610145315	0.65197241	Non	0.003833333	0.951843546	0.962023477	Non	0.453933333	0.00000000000102	0.000000000079	Hypermethylated	0.247433333	0.000000033	0.000000179	Hypermethylated	0.137233333	0.0000387	0.000100965	Hypermethylated	0.174690476	0.00000000000219	0.0000000000152	Hypermethylated	PEX10	-30332
chr1	2398268	2399817	1549	cUMR	0.0668	0.02969946	0.035481513	Non	0.035133333	0.238289211	0.309593602	Non	0.1398	0.000273549	0.000754578	Hypermethylated	0.010533333	0.267557128	0.351309958	Non	0.193883333	0.0000871	0.000280143	Hypermethylated	0.011133333	0.685241416	0.704477288	Non	-0.047066667	0.040076171	0.054404249	Non	0.058647619	0.000461528	0.000586228	Non	PLCH2	7937
chr1	2456577	2458976	2399	cUMR	-0.036933333	0.00014919	0.000266804	Non	0.007833333	0.452589502	0.522045341	Non	-0.040933333	0.00000864	0.0000359	Non	0.014833333	0.265698782	0.349498321	Non	-0.029016667	0.014072567	0.023974601	Non	-0.028766667	0.00964097	0.014504189	Non	-0.007366667	0.20405115	0.23764217	Non	-0.017766667	0.000272566	0.000355986	Non	PANK4	0
chr1	2460127	2462794	2667	cUMR	0.064033333	0.000000274	0.00000091	Non	0.097833333	0.0000347	0.000171684	Non	0.0942	0.00000147	0.00000752	Non	-0.008166667	0.212411414	0.292279464	Non	0.136533333	0.00000218	0.0000114	Hypermethylated	0.036283333	0.124426792	0.147618985	Non	0.092633333	0.00000246	0.00000842	Non	0.072919048	0.00000000527	0.0000000155	Non	HES5	0
chr1	2471996	2472738	742	cUMR	0.0416	0.000104759	0.000193047	Non	0.197166667	0.0000000272	0.000000346	Hypermethylated	-0.0034	0.184427266	0.229883012	Non	0.032166667	0.029411042	0.059689008	Non	0.140516667	0.000000209	0.00000153	Hypermethylated	0.261266667	0.00000000247	0.0000000172	Hypermethylated	0.195366667	0.0000000000744	0.000000000915	Hypermethylated	0.113138095	0.0000000000362	0.000000000178	Hypermethylated	LOC115110	8621
chr1	2476386	2480587	4201	Canyon	-0.033166667	0.070587438	0.080028986	Non	-0.037233333	0.002637633	0.007300603	Non	0.192333333	0.000000012	0.00000011	Hypermethylated	-0.041833333	0.044492955	0.083400065	Non	0.036416667	0.001677153	0.003703136	Non	-0.052083333	0.004807731	0.007737192	Non	-0.015233333	0.451701934	0.48705463	Non	0.01202381	0.0000142	0.0000222	Non	LOC115110	772
chr1	2514903	2519144	4241	Canyon	-0.031533333	0.000000736	0.00000221	Non	-0.025033333	0.005329778	0.01329658	Non	-0.001366667	0.000112834	0.000347306	Non	-0.044233333	0.000000523	0.00000822	Non	-0.019533333	0.002889446	0.005975786	Non	-0.047033333	0.000000454	0.00000191	Non	0.029366667	0.00000538	0.000017	Non	-0.018947619	0.00000000402	0.0000000122	Non	FAM213B	0
chr1	2559236	2561085	1849	cUMR	-0.0213	0.001102695	0.001671674	Non	0.061333333	0.003258824	0.008733083	Non	0.028866667	0.011378467	0.019904057	Non	0.018733333	0.012792031	0.030341624	Non	-0.012466667	0.011377607	0.019892445	Non	0.224033333	0.000000000136	0.00000000128	Hypermethylated	0.151133333	0.0000000985	0.000000481	Hypermethylated	0.058504762	0.00000000326	0.0000000101	Non	MMEL1	0
chr1	2573629	2575378	1749	cUMR	-0.008133333	0.00000708	0.0000169	Non	0.0508	0.00000244	0.0000168	Non	-0.0043	0.000190526	0.000549362	Non	0.0106	0.177143417	0.252248541	Non	0.01245	0.147606837	0.187241647	Non	0.10195	0.00000000000184	0.0000000000312	Hypermethylated	0.1138	0.000000000000186	0.00000000000594	Hypermethylated	0.035971429	0.00000000000135	0.00000000000999	Non	TTC34	0
chr1	2581964	2588063	6099	Canyon	-0.3443	0.00000000000000022	0.00000000000000775	Hypomethylated	-0.122033333	0.000425534	0.001530919	Non	-0.178466667	0.00000000745	0.0000000731	Hypomethylated	-0.245633333	0.000000000433	0.0000000304	Hypomethylated	-0.333633333	0.00000000000000355	0.0000000000011	Hypomethylated	-0.284883333	0.0000000000265	0.000000000308	Hypomethylated	-0.318233333	0.0000000000000204	0.000000000000988	Hypomethylated	-0.258290476	0.00000000000000022	0.00000000000000546	Hypomethylated	TTC34	0
chr1	2591140	2592289	1149	cUMR	-0.344066667	0.0000000000492	0.000000000428	Hypomethylated	-0.1155	0.036874783	0.066792864	Non	-0.216733333	0.00000431	0.0000195	Hypomethylated	-0.2929	0.0000000541	0.0000013	Hypomethylated	-0.2684	0.00000312	0.0000155	Hypomethylated	-0.22515	0.0000622	0.00015621	Hypomethylated	-0.3103	0.00000000583	0.0000000392	Hypomethylated	-0.255214286	0.000000000000123	0.00000000000124	Hypomethylated	TTC34	0
chr1	2604420	2607119	2699	cUMR	-0.3985	0.0000000000000167	0.000000000000359	Hypomethylated	-0.1492	0.003014692	0.008178377	Non	-0.2595	0.0000000938	0.000000667	Hypomethylated	-0.3046	0.0000000136	0.000000431	Hypomethylated	-0.301	0.000000069	0.000000592	Hypomethylated	-0.25275	0.00000498	0.0000164	Hypomethylated	-0.331	0.000000000618	0.00000000559	Hypomethylated	-0.2882	0.000000000000000444	0.00000000000001	Hypomethylated	TTC34	0
chr1	2612472	2633171	20699	Canyon	-0.324833333	0.000000000000000111	0.00000000000000775	Hypomethylated	-0.078633333	0.005791937	0.014243118	Non	-0.192333333	0.000000000935	0.0000000114	Hypomethylated	-0.243633333	0.000000000101	0.0000000102	Hypomethylated	-0.302333333	0.000000000000507	0.0000000000463	Hypomethylated	-0.241333333	0.00000000188	0.0000000134	Hypomethylated	-0.286233333	0.000000000000484	0.000000000013	Hypomethylated	-0.237719048	0.00000000000000022	0.00000000000000546	Hypomethylated	TTC34	0
chr1	2685890	2686633	743	cUMR	0.053693581	0.217662368	0.232418811	Non	0.052428672	0.005630484	0.013901411	Non	0.100862852	0.140524244	0.181069043	Non	-0.007372215	0.905399296	0.924583111	Non	-0.062441855	0.063139987	0.08894817	Non	-0.084180545	0.066953193	0.083788848	Non	0.010027061	0.737736811	0.759290373	Non	0.017608895	0.28196426	0.287281191	Non	TTC34	0
chr1	2687650	2690649	2999	cUMR	-0.2162	0.00000000000000999	0.000000000000225	Hypomethylated	-0.0463	0.102035643	0.154765084	Non	-0.111366667	0.00000124	0.00000645	Hypomethylated	-0.1447	0.0000000499	0.00000121	Hypomethylated	-0.2067	0.0000000000011	0.0000000000827	Hypomethylated	-0.17995	0.00000000165	0.0000000119	Hypomethylated	-0.2101	0.000000000000244	0.00000000000738	Hypomethylated	-0.157642857	0.000000000000000111	0.00000000000000546	Hypomethylated	TTC34	0
chr1	2703087	2703736	649	cUMR	-0.090366667	0.0000425	0.000085	Non	0.068066667	0.000422627	0.00152137	Non	-0.014866667	0.131402059	0.170683852	Non	0.010866667	0.000000364	0.00000611	Non	0.134966667	0.00036578	0.000981577	Hypermethylated	0.007716667	0.000601613	0.001192831	Non	0.045666667	0.234888737	0.269980873	Non	0.016066667	0.000000308	0.000000632	Non	TTC34	0
chr1	2717835	2719034	1199	cUMR	-0.234566667	0.00000000405	0.0000000213	Hypomethylated	-0.058933333	0.077697711	0.123926511	Non	0.0126	0.000539275	0.001366648	Non	-0.120933333	0.00000179	0.0000224	Hypomethylated	-0.153733333	0.00000695	0.0000311	Hypomethylated	-0.085733333	0.003906205	0.006427567	Non	-0.191733333	0.00000191	0.00000673	Hypomethylated	-0.118504762	0.0000000019	0.00000000622	Hypomethylated	TTC34	-11606
chr1	2738231	2739051	820	cUMR	-0.138933333	0.00000159	0.00000441	Hypomethylated	-0.004566667	0.017221377	0.035532453	Non	-0.034266667	0.070152378	0.098343921	Non	-0.088566667	0.004551224	0.013252222	Non	-0.061016667	0.12945534	0.166877864	Non	0.021483333	0.000530743	0.00106536	Non	-0.054366667	0.028063356	0.03934912	Non	-0.055280952	0.0000106	0.0000169	Non	TTC34	-32002
chr1	2928362	2929811	1449	cUMR	-0.042233333	0.007454961	0.009780045	Non	0.020766667	0.201857549	0.270804885	Non	-0.150733333	0.001143592	0.002647797	Non	-0.058033333	0.126376733	0.192419694	Non	0.054516667	0.416442014	0.466091666	Non	0.069266667	0.345835398	0.376012517	Non	0.118366667	0.053354083	0.070591916	Non	-0.007347619	0.004562506	0.005233342	Non	ACTRT2	8235
chr1	2984092	2988041	3949	Canyon	0.292933333	0.00000000342	0.0000000183	Hypermethylated	0.222366667	0.000000743	0.00000599	Hypermethylated	0.1401	0.000000759	0.0000042	Hypermethylated	-0.032033333	0.14094784	0.210027273	Non	0.102766667	0.00244008	0.005150757	Non	0.062016667	0.034464707	0.045941351	Non	0.353566667	0.000000000416	0.00000000397	Hypermethylated	0.170766667	0.000000000497	0.00000000187	Hypermethylated	LINC00982	0
chr1	3086669	3088218	1549	cUMR	-0.0049	0.542441225	0.554828041	Non	0.176866667	0.000000386	0.00000338	Hypermethylated	-0.0354	0.069319775	0.097301255	Non	0.046466667	0.000215379	0.001122448	Non	0.007016667	0.012717646	0.021920451	Non	0.195266667	0.000000753	0.00000301	Hypermethylated	0.036666667	0.00350619	0.005969839	Non	0.053352381	0.00000037	0.000000749	Non	PRDM16	0
chr1	3309504	3311103	1599	cUMR	-0.0563	0.023881285	0.028959904	Non	0.168666667	0.001410889	0.004265234	Non	0.453866667	0.000000000154	0.00000000232	Hypermethylated	0.185466667	0.000317378	0.001541348	Non	0.088366667	0.008292544	0.015035923	Non	0.099116667	0.029516718	0.039875202	Non	0.294466667	0.000000795	0.00000308	Hypermethylated	0.182238095	0.0000000224	0.0000000578	Hypermethylated	PRDM16	0
chr1	3340949	3341698	749	cUMR	-0.1284	0.0000027	0.00000708	Hypomethylated	-0.0954	0.000950039	0.00304819	Non	0.021933333	0.464948472	0.514573725	Non	-0.016	0.147667449	0.217993252	Non	-0.07315	0.001085068	0.002531511	Non	-0.00715	0.591473995	0.615004329	Non	-0.0206	0.632129331	0.660643145	Non	-0.046285714	0.00024743	0.000324847	Non	PRDM16	0
chr1	3369514	3372047	2533	cUMR	-0.0365	0.026230403	0.031598692	Non	-0.044266667	0.002460609	0.006874597	Non	0.052333333	0.011246317	0.019702925	Non	-0.032466667	0.085447646	0.140932442	Non	-0.034666667	0.044324491	0.065251664	Non	-0.052166667	0.001741994	0.003106992	Non	-0.001866667	0.913633749	0.921897773	Non	-0.018438095	0.009378357	0.010478956	Non	ARHGEF16	0
chr1	3400462	3400711	249	cUMR	0.032033333	0.051528165	0.059546622	Non	-0.095766667	0.02471608	0.048050293	Non	0.182533333	0.003692585	0.007409152	Non	0.061233333	0.255881297	0.339269738	Non	-0.122966667	0.001901732	0.004135349	Non	-0.105716667	0.015475968	0.022209379	Non	0.008233333	0.001271488	0.002370794	Non	0.006890476	0.001882672	0.002237773	Non	ARHGEF16	-2786
chr1	3407416	3408565	1149	cUMR	-0.154266667	0.000000188	0.000000648	Hypomethylated	-0.018033333	0.043819584	0.076984581	Non	-0.098933333	0.000901196	0.002147436	Non	-0.081433333	0.005500286	0.015441041	Non	-0.098683333	0.000276135	0.000767953	Non	-0.083683333	0.018120277	0.025594187	Non	-0.073433333	0.016044908	0.023727636	Non	-0.088947619	0.000000389	0.000000784	Non	MEGF6	0
chr1	3446617	3448566	1949	cUMR	-0.070766667	0.002550091	0.003623996	Non	-0.0576	0.027927608	0.053169911	Non	0.221566667	0.000000856	0.00000466	Hypermethylated	-0.0432	0.15920397	0.231574652	Non	-0.0731	0.007617323	0.013956984	Non	-0.03785	0.253880921	0.2829405	Non	-0.033	0.283593114	0.319993429	Non	-0.005942857	0.000467201	0.000593183	Non	MEGF6	0
chr1	3526787	3528786	1999	cUMR	-0.0101	0.076205919	0.086099219	Non	0.0589	0.001729687	0.005089786	Non	0.0194	0.281570348	0.332970961	Non	-0.0371	0.007307959	0.01933147	Non	0.0089	0.876780288	0.892483373	Non	0.07115	0.002082581	0.003650955	Non	0.0393	0.01638026	0.024180528	Non	0.019471429	0.002942077	0.00343682	Non	MEGF6	0
chr1	3535275	3536274	999	cUMR	-0.0608	0.006955449	0.009174188	Non	-0.0262	0.204823815	0.274202192	Non	0.040533333	0.033449602	0.05142711	Non	0.0158	0.790205989	0.829299883	Non	-0.0338	0.118011294	0.153903894	Non	-0.0653	0.03937464	0.051821414	Non	-0.0214	0.271681288	0.307748887	Non	-0.019342857	0.139517208	0.144177952	Non	TPRG1L	5282
chr1	3537499	3538198	699	cUMR	-0.198566667	0.00000000643	0.0000000321	Hypomethylated	-0.145433333	0.00000874	0.0000514	Hypomethylated	-0.038066667	0.348231967	0.400845185	Non	-0.048633333	0.145279951	0.215125739	Non	-0.189233333	0.000000558	0.0000035	Hypomethylated	-0.125233333	0.000661939	0.00130067	Non	-0.078833333	0.026005879	0.03670988	Non	-0.115490476	0.00000000721	0.0000000207	Hypomethylated	TPRG1L	3358
chr1	3540190	3542639	2449	cUMR	-0.010633333	0.199713484	0.214154564	Non	0.010333333	0.02671901	0.051290368	Non	-0.029966667	0.00000317	0.0000149	Non	0.001533333	0.951716704	0.961935623	Non	-0.003716667	0.340106144	0.389741684	Non	-0.011716667	0.060954297	0.076924674	Non	-0.002866667	0.347360744	0.384259712	Non	-0.007438095	0.007506735	0.008456701	Non	TPRG1L	0
chr1	3565468	3570067	4599	Canyon	0.1401	0.00000000637	0.0000000319	Hypermethylated	0.1407	0.00000000424	0.000000073	Hypermethylated	0.058266667	0.0000595	0.000198514	Non	-0.0023	0.863419921	0.890354455	Non	0.1326	0.000000378	0.00000253	Hypermethylated	0.14785	0.0000000279	0.000000154	Hypermethylated	0.1863	0.000000000155	0.00000000169	Hypermethylated	0.112442857	0.00000000000265	0.000000000018	Hypermethylated	WRAP73	0
chr1	3612045	3612644	599	cUMR	-0.102233333	0.00000221	0.00000593	Hypomethylated	0.106033333	0.017929029	0.036762748	Non	0.049933333	0.001227814	0.002821115	Non	-0.130366667	0.00010386	0.000617826	Hypomethylated	0.027933333	0.022164063	0.035654846	Non	0.029683333	0.000304105	0.000646122	Non	0.074233333	0.00000000162	0.0000000129	Non	0.004747619	0.00000000517	0.0000000153	Non	TP73	0
chr1	3640991	3641340	349	cUMR	-0.0216	0.030737508	0.036648977	Non	0.035266667	0.045591415	0.079627189	Non	0.0034	0.26346289	0.314459075	Non	-0.005933333	0.712802483	0.763189863	Non	0.015566667	0.086092709	0.116763963	Non	0.037316667	0.00930855	0.01404266	Non	0.085917874	0.000000000916	0.0000000078	Non	0.019390317	0.00000277	0.0000048	Non	TP73	0
chr1	3662914	3664613	1699	cUMR	0.1754	0.00000619	0.000015	Hypermethylated	0.1356	0.0000387	0.000189495	Hypermethylated	0.247233333	0.000000000238	0.00000000338	Hypermethylated	-0.0516	0.145560392	0.215448334	Non	0.06165	0.004988334	0.00965378	Non	0.2639	0.00000138	0.00000519	Hypermethylated	-0.0078	0.710570324	0.73386921	Non	0.120542857	0.00000000745	0.0000000213	Hypermethylated	TP73-AS1	0
chr1	3688159	3690108	1949	cUMR	-0.056366667	0.001883695	0.002737173	Non	0.008	0.124966383	0.182404441	Non	0.291966667	0.000000000102	0.0000000016	Hypermethylated	-0.055	0.00439712	0.012870375	Non	-0.02595	0.070729229	0.098419734	Non	-0.0092	0.80391479	0.816416141	Non	0.0154	0.299933385	0.336759463	Non	0.031857143	0.00000147	0.00000267	Non	SMIM1	0
chr1	3711746	3714045	2299	cUMR	-0.050033333	0.00000000116	0.00000000695	Non	-0.023233333	0.024824844	0.048218948	Non	-0.0337	0.0000708	0.000230809	Non	-0.003833333	0.920045871	0.936167943	Non	-0.062533333	0.0000000319	0.000000309	Non	-0.030533333	0.003256707	0.005445472	Non	-0.039433333	0.000158195	0.000359067	Non	-0.034490476	0.00000000136	0.0000000046	Non	LRRC47	0
chr1	3718024	3718573	549	cUMR	-0.105366667	0.0000000951	0.000000352	Hypomethylated	-0.086866667	0.00000753	0.0000452	Non	-0.043033333	0.058240146	0.083624562	Non	-0.071866667	0.000799799	0.003266149	Non	-0.055366667	0.005762735	0.010981368	Non	-0.002116667	0.723353823	0.740311749	Non	-0.003066667	0.415078347	0.451219338	Non	-0.05512381	0.00000206	0.00000365	Non	LRRC47	-4957
chr1	3772678	3775027	2349	cUMR	-0.062933333	0.0000055	0.0000134	Non	-0.0412	0.008351559	0.019395482	Non	-0.0016	0.009378039	0.016821138	Non	-0.0288	0.117711013	0.182055067	Non	-0.0301	0.095704919	0.128058076	Non	-0.0576	0.000447496	0.000913259	Non	-0.0516	0.000322542	0.00068235	Non	-0.038457143	0.00000336	0.00000574	Non	CEP104	0
chr1	3815975	3818177	2202	cUMR	-0.012333333	0.000215537	0.000373159	Non	0.004066667	0.119776954	0.176325082	Non	0.004333333	0.004321839	0.008504629	Non	-0.016333333	0.0000512	0.000350294	Non	0.002416667	0.203416259	0.247960191	Non	-0.009083333	0.070772269	0.088179005	Non	0.009466667	0.114726944	0.141303437	Non	-0.002533333	0.000112949	0.000154914	Non	C1orf174	0
chr1	3826990	3828506	1516	cUMR	-0.231266667	0.0000000000671	0.000000000565	Hypomethylated	-0.138133333	0.000164238	0.000668636	Hypomethylated	0.019066667	0.022070944	0.035760479	Non	-0.142333333	0.000161126	0.000882257	Hypomethylated	-0.192683333	0.000000922	0.00000542	Hypomethylated	-0.146933333	0.00027728	0.000595057	Hypomethylated	-0.153933333	0.0000188	0.0000527	Hypomethylated	-0.137247619	0.000000000569	0.00000000211	Hypomethylated	LINC01134	0
chr1	4087045	4087994	949	cUMR	-0.3165	0.000000000679	0.00000000436	Hypomethylated	-0.2115	0.000287994	0.00108519	Non	-0.098666667	0.0000205	0.0000772	Non	-0.2237	0.00000391	0.0000423	Hypomethylated	-0.021	0.006400677	0.012023034	Non	0.00825	0.045390239	0.058914897	Non	0.0113	0.000246794	0.00053502	Non	-0.133185714	0.00000000486	0.0000000145	Hypomethylated	LINC01346	-74403
chr1	4160652	4162551	1899	cUMR	-0.390966667	0.00000000000105	0.000000000014	Hypomethylated	-0.237733333	0.00000398	0.0000256	Hypomethylated	-0.025133333	0.0000356	0.000126173	Non	-0.182733333	0.000128554	0.000737406	Hypomethylated	-0.119633333	0.051418605	0.074307979	Non	-0.206133333	0.0000119	0.0000355	Hypomethylated	-0.216533333	0.000000426	0.00000176	Hypomethylated	-0.199561905	0.0000000000303	0.000000000152	Hypomethylated	LINC01346	-148010
chr1	4178202	4181801	3599	Canyon	-0.501633333	0.00000000000000022	0.00000000000000775	Hypomethylated	-0.348033333	0.00000000000268	0.00000000021	Hypomethylated	-0.2573	0.00000000000464	0.000000000107	Hypomethylated	-0.267633333	0.0000000000738	0.00000000801	Hypomethylated	-0.213633333	0.00000232	0.000012	Hypomethylated	-0.313883333	0.000000000183	0.00000000166	Hypomethylated	-0.201833333	0.00000000064	0.00000000575	Hypomethylated	-0.30717619	0.00000000000000022	0.00000000000000546	Hypomethylated	LINC01346	-165560
chr1	4201087	4201986	899	cUMR	-0.538766667	0.00000000000333	0.0000000000392	Hypomethylated	-0.3825	0.00000000314	0.0000000569	Hypomethylated	-0.295933333	0.0000019	0.00000949	Hypomethylated	-0.3887	0.000000155	0.00000306	Hypomethylated	-0.24035	0.001260519	0.002885806	Non	-0.3531	0.00000501	0.0000164	Hypomethylated	-0.2497	0.000000868	0.00000332	Hypomethylated	-0.356757143	0.000000000000185	0.00000000000176	Hypomethylated	LINC01346	-188445
chr1	4231521	4234320	2799	cUMR	-0.3913	0.00000000000000022	0.00000000000000775	Hypomethylated	-0.2409	0.000000000637	0.0000000147	Hypomethylated	-0.218466667	0.000000000124	0.00000000191	Hypomethylated	-0.2299	0.000000000522	0.0000000342	Hypomethylated	-0.17705	0.00000859	0.0000374	Hypomethylated	-0.14455	0.00000614	0.0000197	Hypomethylated	-0.2331	0.00000000192	0.0000000149	Hypomethylated	-0.241842857	0.00000000000000022	0.00000000000000546	Hypomethylated	LINC01346	-218879
chr1	4314012	4315837	1825	cUMR	-0.567366667	0.00000000000000022	0.00000000000000775	Hypomethylated	-0.430666667	0.000000000000126	0.0000000000206	Hypomethylated	-0.333366667	0.000000000000393	0.0000000000125	Hypomethylated	-0.359266667	0.000000000121	0.0000000118	Hypomethylated	-0.238866667	0.00000927	0.0000401	Hypomethylated	-0.342116667	0.00000000671	0.0000000423	Hypomethylated	-0.278066667	0.000000000345	0.00000000337	Hypomethylated	-0.373380952	0.00000000000000022	0.00000000000000546	Hypomethylated	LOC284661	156274
chr1	4497755	4500804	3049	cUMR	-0.634766667	0.00000000000000022	0.00000000000000775	Hypomethylated	-0.496933333	0.000000000000128	0.0000000000209	Hypomethylated	-0.3566	0.000000000211	0.00000000304	Hypomethylated	-0.461733333	0.000000000059	0.00000000699	Hypomethylated	-0.438933333	0.0000000011	0.0000000194	Hypomethylated	-0.500933333	0.0000000000544	0.000000000579	Hypomethylated	-0.464133333	0.0000000000347	0.000000000472	Hypomethylated	-0.480619048	0.00000000000000022	0.00000000000000546	Hypomethylated	LOC284661	-13012
chr1	4516232	4517031	799	cUMR	-0.689833333	0.00000000000000022	0.00000000000000775	Hypomethylated	-0.461266667	0.000000000000144	0.0000000000229	Hypomethylated	-0.426833333	0.00000000000194	0.0000000000491	Hypomethylated	-0.450666667	0.00000000000711	0.00000000154	Hypomethylated	-0.342666667	0.00000000531	0.0000000699	Hypomethylated	-0.469916667	0.0000000000687	0.000000000709	Hypomethylated	-0.413066667	0.000000000129	0.00000000145	Hypomethylated	-0.473580952	0.00000000000000022	0.00000000000000546	Hypomethylated	LOC284661	-31489
chr1	4542986	4546235	3249	cUMR	-0.5202	0.00000000000000022	0.00000000000000775	Hypomethylated	-0.3406	0.0000000000000191	0.00000000000517	Hypomethylated	-0.260533333	0.000000000000406	0.0000000000128	Hypomethylated	-0.3562	0.0000000000000153	0.0000000000194	Hypomethylated	-0.2387	0.0000000123	0.00000014	Hypomethylated	-0.33445	0.000000000000705	0.0000000000138	Hypomethylated	-0.3128	0.0000000000000694	0.00000000000265	Hypomethylated	-0.343571429	0.00000000000000022	0.00000000000000546	Hypomethylated	LOC284661	-58243
chr1	4553713	4555762	2049	cUMR	-0.429466667	0.00000000000468	0.000000000053	Hypomethylated	-0.287133333	0.000000397	0.00000346	Hypomethylated	-0.164133333	0.00000751	0.0000318	Hypomethylated	-0.306133333	0.000000262	0.00000472	Hypomethylated	-0.226383333	0.000337834	0.000917723	Hypomethylated	-0.238883333	0.000215882	0.000475378	Hypomethylated	-0.241333333	0.00000471	0.0000151	Hypomethylated	-0.274161905	0.00000000000179	0.0000000000127	Hypomethylated	LOC284661	-68970
chr1	4644821	4648270	3449	cUMR	-0.354033333	0.000000000000000333	0.0000000000000111	Hypomethylated	-0.224733333	0.000000000784	0.0000000174	Hypomethylated	0.006633333	0.002169834	0.004647055	Non	-0.215533333	0.00000000427	0.000000182	Hypomethylated	-0.141783333	0.000288375	0.000798398	Hypomethylated	-0.154283333	0.000000124	0.000000592	Hypomethylated	-0.167133333	0.0000000432	0.000000231	Hypomethylated	-0.180161905	0.0000000000000299	0.000000000000364	Hypomethylated	AJAP1	66835
chr1	4661653	4664152	2499	cUMR	-0.514566667	0.00000000000000022	0.00000000000000775	Hypomethylated	-0.3738	0.000000000000494	0.0000000000576	Hypomethylated	-0.1489	0.00000313	0.0000147	Hypomethylated	-0.3124	0.0000000000667	0.00000000751	Hypomethylated	-0.24565	0.000000822	0.00000493	Hypomethylated	-0.2999	0.00000000904	0.0000000553	Hypomethylated	-0.2746	0.00000000151	0.0000000121	Hypomethylated	-0.313342857	0.00000000000000022	0.00000000000000546	Hypomethylated	AJAP1	50953
chr1	4712131	4713180	1049	cUMR	-0.118066667	0.0000000000999	0.000000000805	Hypomethylated	0.106333333	0.000000403	0.00000351	Hypermethylated	0.053433333	0.000000287	0.00000178	Non	-0.067666667	0.00000192	0.0000236	Non	-0.040566667	0.049570107	0.071937409	Non	0.074683333	0.00000000000555	0.0000000000804	Non	0.076333333	0.00000000072	0.00000000636	Non	0.009247619	0.000000000000352	0.0000000000031	Non	AJAP1	1925
chr1	4713741	4720030	6289	Canyon	0.1988	0.0000000408	0.000000165	Hypermethylated	0.330533333	0.0000000000302	0.0000000013	Hypermethylated	0.025133333	0.406128282	0.457822863	Non	0.057533333	0.001169916	0.004462271	Non	0.113133333	0.000812776	0.001977812	Non	0.541633333	0.00000000000000455	0.000000000000192	Hypermethylated	0.516933333	0.000000000000002	0.000000000000146	Hypermethylated	0.242504762	0.00000000000000555	0.0000000000000863	Hypermethylated	AJAP1	0
chr1	4814803	4818002	3199	cUMR	-0.503233333	0.00000000000000022	0.00000000000000775	Hypomethylated	-0.285533333	0.00000000154	0.0000000306	Hypomethylated	0.039266667	0.000023	0.0000857	Non	-0.253333333	0.00000000817	0.000000294	Hypomethylated	-0.130483333	0.004174919	0.008238389	Non	-0.223233333	0.00000116	0.00000443	Hypomethylated	-0.230133333	0.000000000137	0.00000000153	Hypomethylated	-0.229819048	0.00000000000000466	0.0000000000000745	Hypomethylated	AJAP1	0
chr1	4838144	4838443	299	cUMR	-0.212266667	0.0000000163	0.0000000736	Hypomethylated	-0.096	0.039701462	0.070933945	Non	0.084066667	0.022492309	0.036374717	Non	-0.1292	0.001989035	0.006817204	Non	-0.10535	0.0000784	0.000255311	Hypomethylated	-0.13885	0.002118655	0.003705033	Non	-0.0544	0.003072789	0.00529249	Non	-0.089828571	0.000000189	0.000000403	Non	AJAP1	0
chr1	5025335	5026584	1249	cUMR	-0.363114433	0.000000000000000111	0.00000000000000775	Hypomethylated	-0.2556	0.00000000317	0.0000000572	Hypomethylated	-0.118633333	0.0000361	0.000127506	Hypomethylated	-0.2674	0.00000000158	0.0000000824	Hypomethylated	-0.18305	0.00013271	0.000404355	Hypomethylated	-0.24205	0.000000753	0.00000302	Hypomethylated	-0.189	0.000000343	0.00000146	Hypomethylated	-0.232881102	0.00000000000000278	0.000000000000048	Hypomethylated	AJAP1	-181485
chr1	5326242	5329091	2849	cUMR	-0.489366667	0.00000000000000022	0.00000000000000775	Hypomethylated	-0.303066667	0.0000000000965	0.00000000329	Hypomethylated	-0.0472	0.002505852	0.005282655	Non	-0.317866667	0.000000000124	0.000000012	Hypomethylated	-0.187866667	0.0000596	0.000201162	Hypomethylated	-0.328366667	0.000000000662	0.00000000525	Hypomethylated	-0.297466667	0.0000000000936	0.00000000111	Hypomethylated	-0.282180952	0.000000000000000111	0.00000000000000546	Hypomethylated	MIR4417	295040
chr1	5352753	5355352	2599	cUMR	-0.5211	0.00000000000000022	0.00000000000000775	Hypomethylated	-0.360433333	0.000000000000204	0.0000000000295	Hypomethylated	-0.103266667	0.000723839	0.00176746	Non	-0.301233333	0.000000000469	0.000000032	Hypomethylated	-0.339183333	0.0000000014	0.0000000235	Hypomethylated	-0.362683333	0.000000000102	0.000000001	Hypomethylated	-0.356033333	0.0000000000037	0.0000000000718	Hypomethylated	-0.332633333	0.00000000000000022	0.00000000000000546	Hypomethylated	MIR4417	268779
chr1	5388156	5391455	3299	cUMR	-0.5684	0.000000000000000222	0.00000000000000775	Hypomethylated	-0.419366667	0.0000000000109	0.000000000596	Hypomethylated	-0.219066667	0.000000276	0.00000172	Hypomethylated	-0.338366667	0.0000000127	0.000000409	Hypomethylated	-0.433566667	0.00000000116	0.0000000201	Hypomethylated	-0.436316667	0.000000000841	0.0000000065	Hypomethylated	-0.459966667	0.00000000000181	0.0000000000391	Hypomethylated	-0.408366667	0.00000000000000022	0.00000000000000546	Hypomethylated	MIR4417	232676
chr1	5419330	5422279	2949	cUMR	-0.555166667	0.00000000000000022	0.00000000000000775	Hypomethylated	-0.405333333	0.00000000000573	0.000000000368	Hypomethylated	-0.248666667	0.0000000000482	0.000000000825	Hypomethylated	-0.350533333	0.000000000669	0.0000000419	Hypomethylated	-0.326083333	0.000000000252	0.00000000594	Hypomethylated	-0.354583333	0.00000000645	0.0000000408	Hypomethylated	-0.376533333	0.0000000000228	0.000000000335	Hypomethylated	-0.377361905	0.00000000000000022	0.00000000000000546	Hypomethylated	MIR4417	201852
chr1	5515170	5516859	1689	cUMR	-0.3117	0.000000000318	0.00000000222	Hypomethylated	-0.074533333	0.070585844	0.114719717	Non	0.052966667	0.004445702	0.00871563	Non	-0.119133333	0.002766441	0.008889944	Non	-0.114283333	0.000523541	0.001348758	Non	-0.187533333	0.000104697	0.000249184	Hypomethylated	-0.172533333	0.00000203	0.00000709	Hypomethylated	-0.131161905	0.00000000583	0.0000000171	Hypomethylated	MIR4417	107272
chr1	5569751	5570500	749	cUMR	-0.209133333	0.0000000015	0.00000000879	Hypomethylated	-0.031833333	0.178832621	0.244890019	Non	0.008866667	0.960975207	0.966469115	Non	-0.108233333	0.003122793	0.009767573	Non	-0.107133333	0.007713178	0.014115444	Non	-0.151133333	0.000133155	0.000309219	Hypomethylated	-0.017833333	0.002720878	0.004737189	Non	-0.086404762	0.000000582	0.00000114	Non	MIR4417	53631
chr1	5711840	5713439	1599	cUMR	-0.255933333	0.00000000000015	0.00000000000249	Hypomethylated	0.090033333	0.001771786	0.005192112	Non	-0.0361	0.029722072	0.046373745	Non	-0.095166667	0.0000238	0.000186229	Non	-0.096016667	0.002970675	0.006121715	Non	-0.042266667	0.062347315	0.078509985	Non	0.055433333	0.00000178	0.00000632	Non	-0.058680952	0.000000000265	0.00000000106	Non	MIR4417	-87638
chr1	5727371	5728920	1549	cUMR	-0.423633333	0.000000000000922	0.0000000000125	Hypomethylated	-0.260066667	0.00000442	0.0000281	Hypomethylated	-0.142633333	0.000088	0.000279424	Hypomethylated	-0.238266667	0.0000189	0.000153871	Hypomethylated	-0.358216667	0.00000000677	0.0000000857	Hypomethylated	-0.277716667	0.00000751	0.0000236	Hypomethylated	-0.307866667	0.0000000889	0.000000439	Hypomethylated	-0.28492381	0.000000000000143	0.00000000000141	Hypomethylated	MIR4417	-103169
chr1	5804856	5808105	3249	cUMR	-0.3103	0.0000000000174	0.00000000017	Hypomethylated	-0.200666667	0.0000045	0.0000286	Hypomethylated	-0.1768	0.0000276	0.000100868	Hypomethylated	-0.125466667	0.00230022	0.007677382	Non	-0.127716667	0.001654131	0.003659681	Non	-0.122216667	0.017545456	0.024866812	Non	-0.058466667	0.000119568	0.000279394	Non	-0.167009524	0.000000000946	0.00000000333	Hypomethylated	MIR4689	114627
chr1	5826730	5827629	899	cUMR	-0.548933333	0.0000000000294	0.000000000271	Hypomethylated	-0.329366667	0.0000156	0.0000852	Hypomethylated	0.029233333	0.688180582	0.723992651	Non	-0.304766667	0.000178098	0.000957563	Hypomethylated	-0.583016667	0.000000000203	0.00000000502	Hypomethylated	-0.398016667	0.0000019	0.00000689	Hypomethylated	-0.442366667	0.000000164	0.000000755	Hypomethylated	-0.354995238	0.0000000000137	0.0000000000757	Hypomethylated	MIR4689	95103
chr1	5830803	5831952	1149	cUMR	-0.112233333	0.00000488	0.0000121	Hypomethylated	0.1303	0.000318245	0.001188034	Non	-0.039733333	0.183953072	0.229422478	Non	-0.0733	0.010286165	0.02547282	Non	0.11735	0.000000795	0.00000479	Hypermethylated	0.06885	0.007745683	0.011885098	Non	0.0331	0.0000332	0.0000879	Non	0.0081	0.000000138	0.000000302	Non	MIR4689	90780
chr1	5890868	5891263	395	cUMR	0.02	0.466366561	0.480401017	Non	0.1578	0.00026169	0.000996355	Hypermethylated	0.200333333	0.00000164	0.00000832	Hypermethylated	0.0504	0.006006291	0.016539779	Non	0.193	0.0000642	0.000214653	Hypermethylated	0.4155	0.00000000057	0.0000000046	Hypermethylated	0.0596	0.097005928	0.121256152	Non	0.145571429	0.0000000146	0.0000000393	Hypermethylated	MIR4689	31469
chr1	5891764	5893113	1349	cUMR	-0.2365	0.000000000141	0.00000000109	Hypomethylated	-0.0287	0.669645733	0.71918943	Non	0.0605	0.000000438	0.00000258	Non	-0.0359	0.000675952	0.002850235	Non	-0.05225	0.0000966	0.000306625	Non	-0.1865	0.00000871	0.0000269	Hypomethylated	-0.1655	0.00000191	0.00000673	Hypomethylated	-0.090328571	0.000000000506	0.0000000019	Non	MIR4689	29619
chr1	5907324	5907750	426	cUMR	0.0052	0.002734043	0.003864096	Non	0.164366667	0.000986295	0.003147915	Non	0.2937	0.0000000129	0.000000117	Hypermethylated	0.026766667	0.009580886	0.024047516	Non	-0.025133333	0.08398791	0.11427591	Non	0.286116667	0.00000499	0.0000164	Hypermethylated	-0.035009271	0.00000549	0.0000174	Non	0.103370004	0.000000014	0.0000000378	Hypermethylated	MIR4689	14982
chr1	6051076	6054238	3162	cUMR	-0.0092	0.000988359	0.001510495	Non	0.019	0.020143502	0.040497077	Non	-0.011866667	0.009575415	0.017127995	Non	0.0032	0.810254289	0.846285882	Non	-0.0112	0.014169425	0.024120853	Non	0.00655	0.647108256	0.668381756	Non	0.0214	0.03305403	0.0456299	Non	0.002085714	0.007755264	0.008723631	Non	NPHP4	0
chr1	6168426	6169625	1199	cUMR	-0.374133333	0.00000000000000266	0.000000000000071	Hypomethylated	-0.241933333	0.0000000147	0.000000206	Hypomethylated	-0.0613	0.008119687	0.014798686	Non	-0.277733333	0.000000000401	0.0000000289	Hypomethylated	-0.266633333	0.0000000227	0.000000232	Hypomethylated	-0.271383333	0.0000000142	0.000000083	Hypomethylated	-0.233933333	0.0000000065	0.000000043	Hypomethylated	-0.243790476	0.000000000000000555	0.0000000000000122	Hypomethylated	CHD5	0
chr1	6187575	6188274	699	cUMR	-0.1242	0.00000411	0.0000103	Hypomethylated	-0.003033333	0.103882651	0.157052527	Non	0.277466667	0.0000000106	0.0000000993	Hypermethylated	-0.093233333	0.014729304	0.034011765	Non	-0.108283333	0.000938261	0.002234211	Non	0.092466667	0.039255678	0.051681781	Non	-0.150433333	0.00000197	0.00000691	Hypomethylated	-0.01077619	0.0000000268	0.000000068	Non	CHD5	0
chr1	6208150	6209381	1231	cUMR	-0.079866667	0.0000000208	0.000000091	Non	0.120233333	0.00000000417	0.0000000721	Hypermethylated	0.2783	0.00000000000000022	0.0000000000000218	Hypermethylated	-0.012566667	0.213019194	0.292915066	Non	-0.073866667	0.000000448	0.00000291	Non	0.086133333	0.00000305	0.0000106	Non	0.000433333	0.527358651	0.559987399	Non	0.050861905	0.000000000000034	0.000000000000408	Non	CHD5	0
chr1	6221378	6222177	799	cUMR	-0.073366667	0.000000608	0.00000187	Non	-0.060833333	0.064632841	0.106565691	Non	-0.036366667	0.002010027	0.004350425	Non	-0.028033333	0.004318663	0.012688508	Non	0.089466667	0.019664666	0.032127399	Non	-0.110283333	0.002057424	0.003611846	Non	-0.172033333	0.000000512	0.00000208	Hypomethylated	-0.058461905	0.00000016	0.000000346	Non	CHD5	0
chr1	6230540	6232083	1543	cUMR	-0.3609	0.000000000124	0.00000000097	Hypomethylated	-0.0313	0.619449978	0.67398018	Non	-0.109566667	0.003676724	0.007384705	Non	-0.1537	0.006806342	0.018279196	Non	-0.25715	0.00000101	0.00000591	Hypomethylated	-0.27115	0.00000705	0.0000223	Hypomethylated	-0.3199	0.000000118	0.000000564	Hypomethylated	-0.213157143	0.000000000356	0.00000000138	Hypomethylated	CHD5	0
chr1	6238609	6242158	3549	Canyon	-0.0402	0.0000146	0.0000325	Non	0.019433333	0.04760472	0.082509598	Non	0.044466667	0.000258053	0.00071692	Non	-0.007166667	0.25506013	0.338478936	Non	0.000633333	0.249608767	0.296791438	Non	0.185883333	0.0000000000699	0.000000000719	Hypermethylated	0.113633333	0.000000015	0.0000000908	Hypermethylated	0.040033333	0.0000000015	0.00000000502	Non	CHD5	0
chr1	6258291	6260190	1899	cUMR	-0.002766667	0.009002436	0.011652828	Non	0.007066667	0.242168661	0.313654298	Non	0.0024	0.531507595	0.577521419	Non	0.002466667	0.07347204	0.125116897	Non	-0.004183333	0.002894071	0.005984326	Non	-0.014433333	0.046938301	0.060731231	Non	0.004466667	0.772310704	0.791628327	Non	-0.000190476	0.018834669	0.02056165	Non	RPL22	0
chr1	6264746	6267095	2349	cUMR	-0.079233333	0.00000381	0.00000962	Non	-0.0074	0.707042273	0.752344681	Non	0.193433333	0.00000000109	0.0000000131	Hypermethylated	-0.0016	0.827721828	0.860724977	Non	0.15485	0.0000000171	0.000000183	Hypermethylated	-0.0579	0.00698002	0.010832337	Non	-0.0188	0.10689487	0.132449158	Non	0.026685714	0.0000000127	0.0000000345	Non	RNF207	0
chr1	6294608	6296683	2075	cUMR	-0.0774	0.000000475	0.00000149	Non	-0.043933333	0.008022533	0.018739724	Non	0.016266667	0.211959577	0.259978719	Non	-0.038533333	0.040575628	0.077381408	Non	-0.035983333	0.060021367	0.085032034	Non	-0.086983333	0.000000581	0.00000239	Non	-0.069133333	0.00000843	0.0000255	Non	-0.046190476	0.000000433	0.000000866	Non	LINC00337	0
chr1	6301400	6307664	6264	Canyon	0.009633333000000001	0.545541186	0.557856722	Non	0.077033333	0.00000288	0.0000194	Non	0.110466667	0.00000000453	0.0000000468	Hypermethylated	0.003233333	0.090608699	0.147572886	Non	0.123883333	0.0000000102	0.00000012	Hypermethylated	0.102133333	0.000000399	0.0000017	Hypermethylated	0.105433333	0.0000000395	0.000000213	Hypermethylated	0.072947619	0.0000000000585	0.000000000272	Non	HES3	0
chr1	6309033	6310382	1349	cUMR	-0.047033333	0.0000388	0.0000782	Non	0.2389	0.000000000101	0.0000000034	Hypermethylated	0.476633333	0.000000000000000111	0.0000000000000218	Hypermethylated	0.0041	0.304145544	0.389238284	Non	0.0993	0.00000000807	0.0000000989	Non	0.13155	0.000000128	0.000000611	Hypermethylated	0.1063	0.000000945	0.00000359	Hypermethylated	0.149928571	0.0000000000000178	0.000000000000233	Hypermethylated	GPR153	0
chr1	6319336	6322235	2899	cUMR	-0.031966667	0.000214239	0.000370993	Non	0.005733333	0.033785527	0.062190042	Non	0.016366667	0.018490016	0.030598297	Non	-0.022066667	0.00808453	0.020980079	Non	0.017283333	0.211305879	0.256461802	Non	0.015533333	0.016550483	0.023597215	Non	0.019933333	0.00962033	0.014898867	Non	0.001590476	0.003068555	0.003576938	Non	GPR153	0
chr1	6452039	6454538	2499	cUMR	-0.0191	0.003082667	0.004318959	Non	-0.023766667	0.000458406	0.001628993	Non	-0.008433333	0.023050105	0.037159581	Non	-0.014166667	0.057478396	0.102690964	Non	-0.011766667	0.034111222	0.051950466	Non	-0.033016667	0.00000217	0.00000777	Non	-0.010566667	0.210713554	0.244719805	Non	-0.016766667	0.0000049	0.00000816	Non	ACOT7	0
chr1	6464754	6465403	649	cUMR	-0.112066667	0.00000214	0.00000574	Hypomethylated	0.006766667	0.140094102	0.200251962	Non	0.3221	0.000000000014	0.000000000279	Hypermethylated	-0.053433333	0.067798717	0.117169642	Non	-0.052483333	0.019335134	0.031670613	Non	-0.120733333	0.0000388	0.000102218	Hypomethylated	-0.096233333	0.000142682	0.000327247	Non	-0.004204762	0.00000000457	0.0000000137	Non	HES2	9891
chr1	6478526	6481375	2849	cUMR	-0.003533333	0.000617484	0.000980117	Non	0.2122	0.000000000261	0.00000000733	Hypermethylated	0.096633333	0.000002	0.00000994	Non	0.0126	0.441423513	0.521774848	Non	0.0578	0.0000102	0.0000435	Non	0.00805	0.484286652	0.512036668	Non	0.1224	0.000000308	0.00000132	Hypermethylated	0.073085714	0.00000000284	0.00000000891	Non	HES2	0
chr1	6482801	6486100	3299	cUMR	-0.069866667	0.00000204	0.00000551	Non	-0.0172	0.465724217	0.534789443	Non	0.0963	0.000000747	0.00000414	Non	-0.026	0.117821947	0.182191561	Non	-0.0357	0.006887754	0.012805207	Non	0.0018	0.907773661	0.914299494	Non	0.0148	0.000504251	0.001023712	Non	-0.0034	0.00000311	0.00000534	Non	ESPN	0
chr1	6498057	6498606	549	cUMR	-0.0701	0.0000129	0.0000291	Non	0.021833333	0.184485946	0.251118898	Non	-0.043570938	0.000000000018	0.000000000347	Non	-0.044566667	0.000227349	0.001173664	Non	0.009733333	0.585795129	0.628125434	Non	-0.062766667	0.058227219	0.073800007	Non	0.046833333	0.002037354	0.003630535	Non	-0.022106044	0.00000000374	0.0000000114	Non	ESPN	0
chr1	6499957	6501080	1123	cUMR	-0.018833333	0.255552535	0.270718524	Non	0.2836	0.0000000548	0.00000063	Hypermethylated	0.107	0.002606709	0.00547316	Non	-0.0252	0.447006506	0.526875166	Non	0.02775	0.249925232	0.297123742	Non	0.025	0.684162817	0.703469407	Non	0.0866	0.023044109	0.03290104	Non	0.070428571	0.000054	0.0000772	Non	ESPN	0
chr1	6506924	6509666	2742	cUMR	-0.024833333	0.08177853	0.092037129	Non	0.4193	0.000000000000121	0.0000000000202	Hypermethylated	0.060666667	0.00341768	0.006938438	Non	-0.0085	0.237897382	0.31982433	Non	0.28325	0.000000000023	0.000000000864	Hypermethylated	0.02325	0.387093169	0.416752499	Non	0.1699	0.000000085	0.000000421	Hypermethylated	0.124128571	0.0000000000127	0.0000000000709	Hypermethylated	ESPN	0
chr1	6535335	6536084	749	cUMR	-0.099466667	0.053717934	0.061925697	Non	-0.093666667	0.117552524	0.173687116	Non	-0.116633333	0.002927341	0.006054679	Non	-0.128266667	0.011502944	0.027817155	Non	-0.128716667	0.037305599	0.056116749	Non	-0.244966667	0.00000127	0.00000479	Hypomethylated	0.023333333	0.868502821	0.880599367	Non	-0.10812381	0.0000753	0.000105517	Hypomethylated	PLEKHG5	0
chr1	6544799	6547254	2455	cUMR	-0.001066667	0.000292458	0.000492121	Non	0.141766667	0.00000733	0.0000441	Hypermethylated	0.000433333	0.883831532	0.899346067	Non	-0.043433333	0.025784858	0.05372972	Non	0.125016667	0.0000117	0.000049	Hypermethylated	-0.046233333	0.028675653	0.038852237	Non	0.048566667	0.003454436	0.005887544	Non	0.029880952	0.0000023	0.00000404	Non	PLEKHG5	0
chr1	6548883	6551582	2699	cUMR	-0.1333	0.0000000000438	0.000000000386	Hypomethylated	-0.0096	0.01061776	0.023718935	Non	0.0197	0.511845031	0.559080843	Non	-0.0516	0.019187465	0.042315322	Non	-0.1378	0.000000000664	0.0000000128	Hypomethylated	-0.08755	0.000105662	0.000251258	Non	-0.0222	0.0000806	0.000195608	Non	-0.057142857	0.00000000192	0.00000000628	Non	PLEKHG5	0
chr1	6556327	6558226	1899	cUMR	-0.043433333	0.006888727	0.009097137	Non	0.0313	0.021715949	0.0431081	Non	0.072733333	0.001808488	0.003969121	Non	-0.0045	0.793842572	0.832246511	Non	-0.01785	0.184471457	0.227676987	Non	0.01165	0.050776453	0.065189716	Non	-0.0033	0.170322751	0.201844145	Non	0.007671429	0.005438301	0.006201598	Non	PLEKHG5	0
chr1	6613234	6615533	2299	cUMR	-0.020566667	0.512700418	0.525545734	Non	-0.014666667	0.00286437	0.00784171	Non	-0.0554	0.011514916	0.02011646	Non	-0.030666667	0.083000888	0.137723017	Non	-0.005316667	0.256769187	0.304194675	Non	-0.027816667	0.095234356	0.115626638	Non	-0.025066667	0.4257616	0.461521054	Non	-0.026866667	0.009818218	0.010954184	Non	NOL9	0
chr1	6639399	6641148	1749	cUMR	-0.043533333	0.001330971	0.001987221	Non	-0.034266667	0.037448897	0.067621015	Non	-0.012033333	0.350566749	0.403037979	Non	-0.044466667	0.001796621	0.006296535	Non	-0.056616667	0.000370503	0.000992263	Non	-0.022116667	0.113348915	0.135543973	Non	-0.022066667	0.336984736	0.374141095	Non	-0.03292381	0.000542845	0.000683606	Non	ZBTB48	0
chr1	6659386	6664599	5213	Canyon	-0.021166667	0.015621382	0.0194907	Non	0.005033333	0.596913438	0.654107463	Non	0.02	0.043138122	0.064292316	Non	-0.015966667	0.497177787	0.573678039	Non	0.004083333	0.972255385	0.975534424	Non	-0.001916667	0.985844282	0.987319238	Non	0.038633333	0.009639765	0.014925129	Non	0.004004762	0.283042024	0.288354895	Non	KLHL21	0
chr1	6672305	6675904	3599	Canyon	-0.025	0.000174973	0.000308493	Non	-0.0216	0.008101064	0.018908499	Non	0.011166667	0.179906164	0.225014351	Non	-0.0206	0.013057407	0.030876008	Non	-0.0045	0.21928541	0.264798824	Non	-0.01125	0.146541952	0.171309206	Non	-0.0092	0.428319226	0.464105588	Non	-0.011514286	0.00090763	0.001114898	Non	PHF13	0
chr1	6684682	6686181	1499	cUMR	-0.0238	0.006036991	0.008048691	Non	0.000933333	0.152943848	0.215160717	Non	-0.002633333	0.921499707	0.931863196	Non	-0.012466667	0.055351347	0.099630772	Non	-0.009216667	0.033873797	0.051631294	Non	-0.025466667	0.00306138	0.005153915	Non	-0.021266667	0.032246236	0.044619576	Non	-0.013180952	0.013785989	0.015206425	Non	THAP3	0
chr1	6760822	6762327	1505	cUMR	-0.0244	0.00000105	0.00000303	Non	-0.0165	0.004234753	0.010956071	Non	-0.001566667	0.056706986	0.081646947	Non	-0.0001	0.194041307	0.271513612	Non	-0.0169	0.004376648	0.008596338	Non	-0.0149	0.015967236	0.022851733	Non	-0.0133	0.029279938	0.040912113	Non	-0.012357143	0.000011	0.0000175	Non	DNAJC11	0
chr1	6780022	6780321	299	cUMR	-0.2975	0.000000000013	0.000000000132	Hypomethylated	-0.184533333	0.0000106	0.0000606	Hypomethylated	0.024166667	0.000157339	0.000465735	Non	-0.250133333	0.0000000408	0.00000103	Hypomethylated	-0.101833333	0.0000123	0.0000512	Hypomethylated	-0.093833333	0.047472487	0.061353212	Non	-0.106533333	0.014485253	0.021647026	Non	-0.146533333	0.000000000365	0.00000000141	Hypomethylated	LOC100505887	4484
chr1	6843734	6846583	2849	cUMR	-0.0088	0.002571094	0.003650458	Non	-0.004066667	0.290383388	0.363523157	Non	-0.0048	0.27426434	0.325641608	Non	-0.005466667	0.075984018	0.128432551	Non	-0.000966667	0.555826731	0.600286863	Non	-0.008966667	0.05884338	0.07449476	Non	-0.003066667	0.543405855	0.575505779	Non	-0.005266667	0.018513686	0.020224062	Non	CAMTA1	0
chr1	7129988	7131940	1952	cUMR	-0.091066667	0.000286382	0.00048257	Non	0.050333333	0.162661141	0.226360219	Non	0.1466	0.00000000935	0.0000000889	Hypermethylated	-0.060866667	0.010799847	0.026462097	Non	0.167683333	0.000000525	0.00000333	Hypermethylated	0.093683333	0.005497224	0.008734809	Non	0.025933333	0.100370413	0.125117717	Non	0.041590476	0.000000112	0.000000251	Non	CAMTA1	0
chr1	7137431	7137980	549	cUMR	-0.4934	0.00000000000000466	0.000000000000116	Hypomethylated	-0.3273	0.00000000383	0.0000000671	Hypomethylated	-0.4134	0.00000000000304	0.0000000000732	Hypomethylated	-0.3251	0.00000000094	0.0000000549	Hypomethylated	-0.2974	0.0000000994	0.000000807	Hypomethylated	-0.32165	0.000000131	0.000000624	Hypomethylated	-0.3913	0.0000000000293	0.000000000411	Hypomethylated	-0.3753	0.00000000000000022	0.00000000000000546	Hypomethylated	CAMTA1	0
chr1	7267837	7268686	849	cUMR	-0.18	0.00000000872	0.0000000421	Hypomethylated	-0.0864	0.013073252	0.028165057	Non	0.144333333	0.0000049100000000000004	0.0000219	Hypermethylated	-0.1394	0.0000125	0.00010933	Hypomethylated	0.067	0.000000292	0.00000202	Non	-0.06825	0.087714297	0.107231084	Non	-0.0538	0.022170727	0.031759457	Non	-0.0462	0.0000000189	0.0000000496	Non	CAMTA1	0
chr1	7278263	7279012	749	cUMR	-0.229933333	0.00000000000193	0.0000000000241	Hypomethylated	-0.1075	0.000524143	0.001828885	Non	-0.041766667	0.000261902	0.000726775	Non	-0.0899	0.0000264	0.000203793	Non	-0.14785	0.00000804	0.0000352	Hypomethylated	-0.18435	0.000000765	0.00000305	Hypomethylated	-0.0993	0.001437393	0.002649588	Non	-0.126928571	0.0000000000563	0.000000000263	Hypomethylated	CAMTA1	0
chr1	7675742	7677391	1649	cUMR	-0.188833333	0.00000244	0.00000648	Hypomethylated	-0.089	0.001110401	0.003478593	Non	0.055166667	0.004043604	0.008024605	Non	-0.169	0.0000369	0.000268774	Hypomethylated	-0.14275	0.003275977	0.006663669	Non	-0.02025	0.378468462	0.408214708	Non	-0.2082	0.00000212	0.00000737	Hypomethylated	-0.108142857	0.00000149	0.00000271	Hypomethylated	CAMTA1	0
chr1	7729068	7730365	1297	cUMR	-0.156766667	0.000000000627	0.00000000407	Hypomethylated	-0.042	0.132883508	0.191845539	Non	-0.040266667	0.0000184	0.0000702	Non	-0.1244	0.00000076	0.0000111	Hypomethylated	-0.07085	0.000149619	0.000448894	Non	-0.07985	0.003910237	0.006431568	Non	0.0092	0.0000102	0.0000303	Non	-0.073457143	0.0000000028	0.00000000882	Non	CAMTA1	0
chr1	7739887	7741443	1556	cUMR	-0.015	0.006685802	0.00884904	Non	0.013433333	0.132169739	0.190952347	Non	-0.016333333	0.002391148	0.00506566	Non	-0.001766667	0.633686062	0.69481148	Non	-0.011666667	0.095207586	0.127518476	Non	0.118083333	0.0000000000647	0.000000000675	Hypermethylated	0.104033333	0.000000000504	0.00000000468	Hypermethylated	0.023319048	0.00000000466	0.000000014	Non	CAMTA1	0
chr1	7762114	7766547	4433	Canyon	-0.021566667	0.000389687	0.000641396	Non	0.003433333	0.583272417	0.642255321	Non	-0.020566667	0.130292083	0.169442991	Non	-0.037966667	0.035749327	0.069767353	Non	-0.003066667	0.494015406	0.541200311	Non	-0.019066667	0.619781891	0.642055885	Non	0.133033333	0.000014	0.0000405	Hypermethylated	0.004319048	0.000434646	0.000553863	Non	CAMTA1	0
chr1	7812404	7813416	1012	cUMR	-0.187033333	0.0000000388	0.000000158	Hypomethylated	-0.015566667	0.824515556	0.854589503	Non	-0.0432	0.13979696	0.18027651	Non	-0.069566667	0.014320978	0.033253581	Non	-0.135116667	0.000697135	0.001728433	Non	-0.025616667	0.713353231	0.730790552	Non	-0.116166667	0.00000221	0.00000768	Hypomethylated	-0.086595238	0.000000622	0.00000121	Non	CAMTA1	0
chr1	7830648	7832357	1709	cUMR	-0.017966667	0.000185529	0.000325557	Non	-0.014633333	0.0000177	0.0000949	Non	-0.0013	0.003487388	0.00705677	Non	-0.010633333	0.140150918	0.209007787	Non	-0.011633333	0.067634674	0.094528972	Non	-0.019883333	0.001560557	0.002812058	Non	0.010366667	0.000386141	0.000802411	Non	-0.009033333	0.0000374	0.0000547	Non	VAMP3	0
chr1	7842992	7844991	1999	cUMR	-0.021066667	0.099811785	0.111272409	Non	0.004866667	0.031110026	0.058167156	Non	0.038933333	0.419479202	0.470824863	Non	-0.039133333	0.263021869	0.346829057	Non	0.060266667	0.188799773	0.23231286	Non	0.044016667	0.107237394	0.128894545	Non	0.065266667	0.018497998	0.026963146	Non	0.019409524	0.388341122	0.393451518	Non	PER3	0
chr1	7962665	7963514	849	cUMR	-0.1265	0.000000529	0.00000164	Hypomethylated	-0.0729	0.006552538	0.015786494	Non	0.174166667	0.000000113	0.000000785	Hypermethylated	-0.0995	0.000228253	0.001177323	Non	-0.04625	0.001250845	0.002866653	Non	-0.11175	0.0000791	0.000193715	Hypomethylated	-0.1379	0.0000000263	0.000000148	Hypomethylated	-0.054214286	0.0000000034	0.0000000105	Non	TNFRSF9	12417
chr1	8001563	8002962	1399	cUMR	-0.111	0.000000858	0.00000254	Hypomethylated	-0.0109	0.199400768	0.26819381	Non	0.119333333	0.000000109	0.000000762	Hypermethylated	0.0001	0.00269365	0.008707119	Non	0.11375	0.0000000795	0.000000668	Hypermethylated	0.09225	0.0000119	0.0000355	Non	0.0215	0.347948811	0.384849962	Non	0.0265	0.00000000677	0.0000000196	Non	TNFRSF9	0
chr1	8013680	8014983	1303	cUMR	0.127433333	0.00000000000761	0.000000000082	Hypermethylated	0.2867	0.00000000000000022	0.000000000000223	Hypermethylated	0.164933333	0.0000000000000123	0.00000000000064	Hypermethylated	-0.0343	0.00351381	0.010747023	Non	0.0466	0.0000000000396	0.00000000135	Non	-0.04715	0.0000269	0.0000737	Non	0.2967	0.00000000000000022	0.000000000000026	Hypermethylated	0.1285	0.00000000000000022	0.00000000000000546	Hypermethylated	PARK7	6731
chr1	8020606	8022708	2102	cUMR	-0.023966667	0.000865555	0.001335946	Non	-0.0057	0.469791747	0.53858755	Non	-0.0218	0.006206078	0.011708609	Non	-0.0191	0.012655457	0.030067161	Non	-0.01805	0.065945094	0.092435137	Non	-0.0388	0.00000706	0.0000223	Non	-0.0245	0.003058901	0.005271583	Non	-0.021385714	0.00000252	0.00000439	Non	PARK7	0
chr1	8042423	8042772	349	cUMR	-0.060133333	0.021939825	0.026748249	Non	-0.048866667	0.19225931	0.259952452	Non	-0.004966667	0.053131813	0.077126102	Non	-0.037666667	0.267513121	0.3512962	Non	-0.05993951	0.0000000856	0.000000711	Non	-0.046931707	0.000000853	0.00000336	Non	0.023733333	0.001986097	0.003544962	Non	-0.032344244	0.000000581	0.00000114	Non	PARK7	0
chr1	8075035	8075634	599	cUMR	-0.109233333	0.009979398	0.012826472	Non	-0.130566667	0.007142593	0.016973872	Non	0.047766667	0.458685676	0.508600167	Non	-0.074966667	0.21425037	0.294339286	Non	-0.101316667	0.019505318	0.031901666	Non	-0.130566667	0.017366892	0.02463547	Non	0.012033333	0.312148681	0.34927024	Non	-0.064680952	0.00336521	0.003907244	Non	ERRFI1	0
chr1	8084192	8089230	5038	Canyon	-0.073833333	0.000029	0.0000603	Non	0.0351	0.026517638	0.050988619	Non	0.042833333	0.106218389	0.141440611	Non	-0.0555	0.005707783	0.015910487	Non	0.0205	0.160295207	0.200883764	Non	-0.08025	0.0000825	0.000201277	Non	-0.0157	0.197040608	0.230245404	Non	-0.0173	0.000436287	0.000555807	Non	ERRFI1	0
chr1	8277048	8277856	808	cUMR	0.0925	0.000000112	0.000000407	Non	0.224766667	0.0000000099	0.000000148	Hypermethylated	0.1925	0.0000000029	0.0000000315	Hypermethylated	0.013166667	0.20983282	0.28930149	Non	0.411916667	0.0000000000000576	0.00000000000948	Hypermethylated	0.247666667	0.0000000011	0.00000000825	Hypermethylated	0.268566667	0.0000000000607	0.000000000765	Hypermethylated	0.196595238	0.0000000000000456	0.000000000000526	Hypermethylated	SLC45A1	100289
chr1	8374191	8375140	949	cUMR	-0.109	0.0000000921	0.000000342	Hypomethylated	0.005833333	0.496788199	0.56337264	Non	0.0885	0.020676382	0.033755128	Non	-0.067366667	0.0252702	0.052824539	Non	-0.118916667	0.000000201	0.00000148	Hypomethylated	-0.039916667	0.008374118	0.012751138	Non	0.076633333	0.0000103	0.0000306	Non	-0.019509524	0.0000000557	0.000000132	Non	SLC45A1	3005
chr1	8377774	8378973	1199	cUMR	-0.002733333	0.017609799	0.02179787	Non	0.089166667	0.004012183	0.010469344	Non	-0.014733333	0.063388363	0.090019021	Non	0.030966667	0.446526053	0.52648913	Non	0.052266667	0.21277334	0.258034922	Non	0.093266667	0.021467982	0.029877876	Non	0.070966667	0.014681664	0.021907937	Non	0.040938095	0.002290709	0.002700132	Non	SLC45A1	0
chr1	8408924	8409530	606	cUMR	-0.1083	0.000000221	0.00000075	Hypomethylated	-0.0636	0.008824809	0.020349418	Non	0.0317	0.045359278	0.0671789	Non	-0.1056	0.00000296	0.0000339	Hypomethylated	-0.0733	0.000640549	0.001606572	Non	-0.03905	0.0466826	0.060436085	Non	-0.0248	0.00000834	0.0000253	Non	-0.053685714	0.000000112	0.000000249	Non	RERE	2934
chr1	8480825	8485059	4234	Canyon	0.010733333	0.221020258	0.235837149	Non	0.033366667	0.025529393	0.049360651	Non	0.044733333	0.00287223	0.005951419	Non	0.025766667	0.041651199	0.079113312	Non	0.062316667	0.001055679	0.002472038	Non	-0.014683333	0.386549983	0.416204884	Non	-0.006233333	0.756385644	0.776725697	Non	0.022509524	0.002659707	0.003116939	Non	RERE	0
chr1	8731414	8731621	207	cUMR	-0.044009369	0.000126005	0.000228324	Non	-0.008466667	0.427462518	0.498526973	Non	-0.046040662	0.013651411	0.023379412	Non	0.021733333	0.634432761	0.695408719	Non	0.200683333	0.002573277	0.005400698	Non	0.041933333	0.474208676	0.502262687	Non	0.114533333	0.064085472	0.083330826	Non	0.030544849	0.000436361	0.000555872	Non	RERE	0
chr1	8760608	8763927	3319	cUMR	-0.028933333	0.000969963	0.001484545	Non	-0.018466667	0.012694594	0.027519038	Non	-0.025766667	0.003608291	0.007267245	Non	-0.018266667	0.005424269	0.015268546	Non	-0.020766667	0.013161218	0.022617059	Non	-0.023266667	0.049313871	0.063484463	Non	-0.028666667	0.0000526	0.000133138	Non	-0.023752381	0.00000294	0.00000507	Non	RERE	0
chr1	8771992	8772984	992	cUMR	-0.0917	0.011448405	0.01458313	Non	-0.045033333	0.06781402	0.110945375	Non	0.0823	0.130626149	0.169785811	Non	-0.067833333	0.101944997	0.162251146	Non	-0.077783333	0.107395123	0.141785856	Non	-0.111533333	0.002722823	0.004645281	Non	-0.054033333	0.144818468	0.174377392	Non	-0.047090476	0.019470845	0.021224427	Non	RERE	0
chr1	8874631	8878880	4249	Canyon	-0.0065	0.317224682	0.33231092	Non	0.0188	0.019477444	0.039371526	Non	-0.029333333	0.00000442	0.00002	Non	-0.0054	0.224812924	0.305678601	Non	-0.0045	0.474766528	0.52253857	Non	-0.00975	0.418225858	0.447372458	Non	-0.009	0.00178422	0.003212235	Non	-0.007142857	0.001574375	0.001886776	Non	RERE	0
chr1	8935193	8940892	5699	Canyon	-0.1122	0.000000000067	0.000000000564	Hypomethylated	-0.021433333	0.138105432	0.197937702	Non	0.053633333	0.002268074	0.004829552	Non	-0.076433333	0.000022	0.000174449	Non	-0.042033333	0.000496229	0.001284715	Non	-0.091533333	0.00000192	0.00000695	Non	-0.104433333	0.00000000957	0.0000000607	Hypomethylated	-0.054204762	0.00000000102	0.00000000355	Non	ENO1-AS1	0
chr1	9170672	9171173	501	cUMR	-0.022133333	0.018167572	0.022445636	Non	0.037033333	0.02118505	0.042256117	Non	0.013866667	0.404424846	0.45611597	Non	-0.07013508	0.00000287	0.0000331	Non	0.058033333	0.00505369	0.00976536	Non	-0.020216667	0.073975106	0.091785197	Non	-0.008766667	0.005074535	0.008349335	Non	-0.003078022	0.0000162	0.000025	Non	GPR157	0
chr1	9187456	9190412	2956	cUMR	-0.082633333	0.000100032	0.000185329	Non	-0.0656	0.000649166	0.002193859	Non	0.208366667	0.000000282	0.00000175	Hypermethylated	-0.0576	0.029740777	0.060234149	Non	-0.0523	0.068056808	0.095063353	Non	-0.03205	0.414172193	0.44339056	Non	-0.0508	0.003151145	0.005416993	Non	-0.012942857	0.000112833	0.000154778	Non	GPR157	0
chr1	9202402	9202601	199	cUMR	-0.013286769	0.006227592	0.008283976	Non	-0.01370246	0.267537535	0.340221224	Non	0.192129602	0.000000133	0.000000909	Hypermethylated	-0.020483845	0.010344039	0.025571467	Non	0.123747338	0.0000122	0.0000509	Hypermethylated	-0.014972674	0.012645912	0.018501681	Non	0.007092199	0.315496006	0.352442826	Non	0.039219567	0.000000359	0.000000729	Non	MIR34A	9126
chr1	9241377	9243826	2449	cUMR	0.041633333	0.140838263	0.153891379	Non	0.077233333	0.006250099	0.015170089	Non	0.0328	0.159640039	0.202903334	Non	0.058033333	0.017483178	0.03919186	Non	0.044383333	0.204399586	0.248944771	Non	0.108133333	0.000433749	0.000887493	Hypermethylated	0.145033333	0.00000911	0.0000273	Hypermethylated	0.070233333	0.000018	0.0000276	Non	MIR34A	-29542
chr1	9256324	9257028	704	cUMR	-0.0758	0.001326647	0.001981484	Non	-0.011033333	0.354126112	0.426263895	Non	0.0077	0.793724376	0.81897538	Non	-0.030433333	0.25151122	0.334672411	Non	-0.015133333	0.71324157	0.746311863	Non	-0.019133333	0.604180705	0.627215209	Non	0.029566667	0.54522769	0.577282996	Non	-0.017290476	0.103598514	0.107812964	Non	H6PD	37835
chr1	9258496	9259145	649	cUMR	-0.005233333	0.0000000252	0.000000107	Non	0.1618	0.000000375	0.00000329	Hypermethylated	-0.030066667	0.16102595	0.204317783	Non	-0.0196	0.075431794	0.127687228	Non	0.19685	0.00000000109	0.0000000192	Hypermethylated	0.2286	0.0000000665	0.000000339	Hypermethylated	0.0346	0.000851896	0.001646001	Non	0.067828571	0.000000000238	0.000000000963	Non	H6PD	35718
chr1	9261076	9264108	3032	cUMR	-0.0964	0.0000117	0.0000267	Non	0.031033333	0.198943505	0.267638547	Non	-0.0594	0.003366529	0.006847861	Non	-0.084166667	0.00219968	0.007405406	Non	-0.008566667	0.00035072	0.000946963	Non	0.021433333	0.668483993	0.688689122	Non	-0.050966667	0.000300018	0.000638513	Non	-0.040109524	0.00000357	0.00000607	Non	H6PD	30755
chr1	9294161	9295760	1599	cUMR	-0.0173	0.002857709	0.004025263	Non	-0.0062	0.424724118	0.49600548	Non	0.014533333	0.256015589	0.306841283	Non	-0.0152	0.049151514	0.090485346	Non	0.0532	0.000797139	0.001943855	Non	-0.0193	0.00522761	0.008343264	Non	-0.0016	0.332112243	0.369207687	Non	0.000114286	0.039370613	0.042015546	Non	H6PD	0
chr1	9327122	9327476	354	cUMR	0.01432441	0.001211237	0.001822224	Non	0.231433333	0.000776417	0.002560467	Non	0.0468	0.009608302	0.01718172	Non	0.004633333	0.885419094	0.908684866	Non	0.233133333	0.003438764	0.00694846	Non	-0.034616667	0.804501477	0.816977521	Non	0.245833333	0.000158998	0.000360722	Hypermethylated	0.102009139	0.0000307	0.0000455	Hypermethylated	H6PD	0
chr1	9340328	9340777	449	cUMR	-0.079133333	0.00000185	0.00000504	Non	-0.016733333	0.698084589	0.74481932	Non	0.1127	0.0000469	0.00016058	Hypermethylated	-0.022533333	0.012652606	0.03006335	Non	0.052901399	0.00000179	0.00000964	Non	-0.026633333	0.094114215	0.114387671	Non	-0.016533333	0.025957163	0.036649701	Non	0.000784799	0.000000926	0.00000175	Non	H6PD	-8935
chr1	9351866	9355494	3628	Canyon	-0.037033333	0.022799197	0.027724432	Non	0.014833333	0.657251054	0.708181839	Non	-0.0122	0.426860931	0.477774952	Non	-0.033166667	0.049959863	0.091791154	Non	-0.012116667	0.505897452	0.552711859	Non	-0.009116667	0.772564231	0.786866138	Non	0.001833333	0.92664623	0.933620487	Non	-0.01322381	0.144874276	0.149585685	Non	SPSB1	0
chr1	9488470	9489869	1399	cUMR	-0.0813	0.00000105	0.00000303	Non	-0.048666667	0.023222685	0.045666479	Non	-0.0433	0.002251246	0.004797959	Non	-0.049066667	0.031889705	0.06371067	Non	-0.036966667	0.073028428	0.101175063	Non	-0.028716667	0.359745174	0.389659259	Non	0.033133333	0.062995847	0.08205124	Non	-0.038095238	0.0000225	0.0000341	Non	LOC100506022	0
chr1	9551130	9551629	499	cUMR	-0.119766667	0.00000000141	0.00000000834	Hypomethylated	-0.0676	0.00000521	0.0000325	Non	-0.043933333	0.004549484	0.008894488	Non	-0.066	0.001767144	0.006215588	Non	-0.0946	0.0000105	0.0000445	Non	-0.05835	0.00462436	0.00747274	Non	0.0664	0.0000000000257	0.000000000369	Non	-0.055142857	0.000000000258	0.00000000104	Non	SLC25A33	47899
chr1	9554915	9555814	899	cUMR	-0.0749	0.000000293	0.000000965	Non	-0.017966667	0.486994449	0.554379499	Non	0.0691	0.00000145	0.00000741	Non	-0.047166667	0.003242671	0.01006205	Non	0.106433333	0.000000307	0.00000211	Hypermethylated	-0.051316667	0.000831706	0.001595229	Non	-0.009166667	0.009456949	0.01466658	Non	-0.005309524	0.0000000359	0.0000000888	Non	SLC25A33	43714
chr1	9598985	9600684	1699	cUMR	-0.0473	0.000000765	0.00000229	Non	-0.020933333	0.065957704	0.108335	Non	0.0212	0.003127931	0.00642654	Non	-0.016933333	0.128759003	0.195392598	Non	-0.030383333	0.014933522	0.025278732	Non	-0.057883333	0.0000000875	0.000000434	Non	-0.024333333	0.00856493	0.013409171	Non	-0.023447619	0.00000125	0.00000231	Non	SLC25A33	0
chr1	9648259	9650308	2049	cUMR	-0.0233	0.000313091	0.000524095	Non	-0.0053	0.027063034	0.051802203	Non	-0.021633333	0.000766219	0.001859135	Non	-0.0043	0.01503342	0.034601125	Non	0.00345	0.651252955	0.68902374	Non	-0.01455	0.0819096	0.100763568	Non	-0.0143	0.052365522	0.069405919	Non	-0.012385714	0.002547679	0.002990014	Non	TMEM201	0
chr1	9675610	9675892	282	cUMR	-0.064085075	0.00000000000129	0.0000000000168	Non	0.0106	0.416724098	0.488271934	Non	0.084533333	0.000000929	0.00000501	Non	-0.0656	0.00000975	0.0000898	Non	-0.09255	0.0000465	0.000162682	Non	-0.09255	0.000193112	0.000429776	Non	0.4074	0.000000000000164	0.00000000000539	Hypermethylated	0.032694999	0.000000000000529	0.0000000000044	Non	TMEM201	-676
chr1	9686394	9687594	1200	cUMR	-0.196666667	0.00000000404	0.0000000213	Hypomethylated	-0.134033333	0.000551399	0.001909335	Non	0.175333333	0.000038	0.000133231	Hypermethylated	-0.121433333	0.001355352	0.005030228	Non	-0.152083333	0.000242633	0.000686022	Hypomethylated	-0.161083333	0.00003	0.0000814	Hypomethylated	-0.150633333	0.0000718	0.0001763	Hypomethylated	-0.097461905	0.000000148	0.000000322	Non	TMEM201	-11460
chr1	9711126	9713025	1899	cUMR	-0.013466667	0.027014514	0.032481577	Non	0.150266667	0.00000000000594	0.000000000377	Hypermethylated	0.031866667	0.003757418	0.007518581	Non	0.009066667	0.522978976	0.596975385	Non	0.037116667	0.003524614	0.007102872	Non	0.293366667	0.000000000000000222	0.0000000000000155	Hypermethylated	0.131666667	0.0000000000358	0.000000000484	Hypermethylated	0.082495238	0.0000000000000396	0.000000000000464	Non	C1orf200	0
chr1	9747036	9750535	3499	cUMR	-0.0692	0.000747395	0.0011668	Non	0.033466667	0.217442069	0.287879871	Non	0.0528	0.045624281	0.067508202	Non	-0.043133333	0.029317047	0.059523312	Non	0.034716667	0.016050915	0.026937457	Non	0.021966667	0.763104341	0.777888681	Non	-0.018533333	0.28826874	0.324782511	Non	-0.000361905	0.013456368	0.014856331	Non	PIK3CD	0
chr1	9881856	9886155	4299	Canyon	-0.0263	0.000107415	0.000197504	Non	-0.0295	0.000828803	0.00271133	Non	0.063366667	0.0000113	0.0000454	Non	-0.0211	0.033188812	0.065723066	Non	-0.01605	0.009747121	0.017340351	Non	-0.0313	0.004916393	0.007892036	Non	-0.0277	0.005768164	0.009394368	Non	-0.010242857	0.000018	0.0000276	Non	CLSTN1	0
chr1	9968841	9971440	2599	cUMR	-0.025933333	0.00000774	0.0000183	Non	-0.0159	0.025383073	0.049125042	Non	-0.002266667	0.096599199	0.130112766	Non	-0.0147	0.002720533	0.008775294	Non	-0.01885	0.00057047	0.001453653	Non	-0.02335	0.000474774	0.000964518	Non	-0.0169	0.005535188	0.0090455	Non	-0.016442857	0.00000554	0.00000917	Non	CTNNBIP1	0
chr1	10002304	10004053	1749	cUMR	-0.007733333	0.034659902	0.040999132	Non	-0.003	0.561694448	0.623165336	Non	0.0071	0.418920833	0.470307674	Non	-0.0002	0.501735182	0.578037692	Non	0.0016	0.876171446	0.89205203	Non	-0.0129	0.007031538	0.010901757	Non	-0.0016	0.925462674	0.932623002	Non	-0.002085714	0.736778411	0.73907864	Non	LZIC	0
chr1	10010329	10010917	588	cUMR	-0.091966667	0.000373609	0.000616956	Non	-0.0254	0.269259596	0.342014047	Non	0.000366667	0.003167933	0.00649796	Non	-0.0286	0.550146758	0.621293217	Non	-0.01105	0.028239261	0.044028837	Non	-0.04055	0.339792413	0.369826005	Non	-0.0326	0.370699876	0.407555058	Non	-0.033971429	0.001320468	0.001595019	Non	NMNAT1	0
chr1	10056812	10058211	1399	cUMR	-0.0442	0.00000947	0.0000219	Non	0.0479	0.011341922	0.025046398	Non	0.3573	0.00000000000000244	0.000000000000165	Hypermethylated	0.0021	0.251736698	0.334898473	Non	0.13655	0.00000000162	0.0000000266	Hypermethylated	0.1868	0.000000000303	0.00000000261	Hypermethylated	0.1143	0.0000000086	0.0000000552	Hypermethylated	0.1141	0.000000000000147	0.00000000000145	Hypermethylated	RBP7	0
chr1	10092238	10095037	2799	cUMR	-0.041366667	0.00000000037	0.00000000254	Non	-0.021466667	0.003336964	0.008921649	Non	0.001966667	0.962056917	0.967315375	Non	-0.018666667	0.02212957	0.047479289	Non	-0.033366667	0.00000432	0.0000205	Non	-0.026866667	0.000264626	0.000570441	Non	-0.040066667	0.00000000337	0.0000000242	Non	-0.025095238	0.00000000149	0.00000000501	Non	UBE4B	0
chr1	10269261	10272185	2924	cUMR	-0.0294	0.002460785	0.003507215	Non	-0.009866667	0.142996099	0.203539991	Non	-0.0274	0.0000312	0.000112414	Non	-0.011866667	0.01387267	0.032384347	Non	-0.016566667	0.317741651	0.367274478	Non	-0.009066667	0.40571877	0.435133336	Non	-0.024066667	0.041008613	0.055566752	Non	-0.019209524	0.000432798	0.000551566	Non	KIF1B	0
chr1	10458034	10460283	2249	cUMR	-0.043733333	0.0000000000922	0.000000000751	Non	-0.0133	0.05050114	0.086725056	Non	-0.023733333	0.0000924	0.000291646	Non	-0.0179	0.005620533	0.015716361	Non	-0.0309	0.00000044	0.00000287	Non	-0.02515	0.000609517	0.001206815	Non	-0.0073	0.000181197	0.000405359	Non	-0.023471429	0.000000000704	0.00000000256	Non	PGD	0
chr1	10487914	10488663	749	cUMR	-0.238066667	0.00000000238	0.0000000133	Hypomethylated	-0.1671	0.00000351	0.0000231	Hypomethylated	-0.186733333	0.00000269	0.0000129	Hypomethylated	-0.1205	0.001826163	0.006376404	Non	-0.26665	0.000000019	0.0000002	Hypomethylated	-0.21865	0.000000355	0.00000153	Hypomethylated	-0.1367	0.000763367	0.001491091	Non	-0.1889	0.00000000000222	0.0000000000154	Hypomethylated	APITD1	1496
chr1	10489962	10491619	1657	cUMR	-0.024933333	0.0000169	0.0000371	Non	-0.017766667	0.016905388	0.034985327	Non	-0.0051	0.279027504	0.330352992	Non	-0.015966667	0.034611753	0.067943469	Non	-0.027516667	0.0000956	0.000303888	Non	-0.016516667	0.046135896	0.05979899	Non	-0.014166667	0.001637229	0.002970907	Non	-0.01702381	0.00000248	0.00000433	Non	APITD1	0
chr1	10531703	10533258	1555	cUMR	-0.023333333	0.00000095	0.00000277	Non	0.018833333	0.04367481	0.076798332	Non	0.003	0.02746384	0.043281906	Non	-0.001966667	0.368075712	0.452503388	Non	0.009833333	0.006113855	0.011561774	Non	-0.019416667	0.002913151	0.004927432	Non	-0.009166667	0.05762326	0.07569591	Non	-0.003480952	0.0000262	0.0000392	Non	DFFA	0
chr1	10534590	10536014	1424	cUMR	-0.0757	0.0000000000000862	0.00000000000153	Non	-0.0383	0.0000478	0.000228571	Non	-0.0192	0.00000000681	0.0000000673	Non	-0.0417	0.000000692	0.0000103	Non	-0.0512	0.00000000187	0.0000000297	Non	-0.0307	0.000142387	0.000328254	Non	-0.0529	0.00000000315	0.0000000228	Non	-0.044614286	0.0000000000000273	0.000000000000339	Non	PEX14	0
chr1	10554661	10556410	1749	cUMR	-0.2785	0.000000012	0.0000000557	Hypomethylated	-0.021166667	0.864968045	0.888984687	Non	0.022833333	0.006258401	0.011789787	Non	-0.130766667	0.006280835	0.01712942	Non	-0.362916667	0.0000000000578	0.0000000018	Hypomethylated	-0.019666667	0.099465149	0.120417541	Non	-0.152766667	0.001144459	0.002153267	Non	-0.131080952	0.000000124	0.000000274	Hypomethylated	PEX14	0
chr1	10752284	10755833	3549	Canyon	-0.0359	0.01165653	0.014825516	Non	-0.030233333	0.017354392	0.035757911	Non	0.0521	0.013635696	0.023357478	Non	-0.034233333	0.033083318	0.065562647	Non	-0.026983333	0.058455555	0.083062796	Non	-0.043733333	0.008017449	0.012259945	Non	0.010966667	0.725654343	0.747877119	Non	-0.012947619	0.0128506	0.014206522	Non	CASZ1	0
chr1	10853368	10857117	3749	Canyon	-0.002333333	0.00854751	0.01110932	Non	-0.0007	0.263922665	0.336352045	Non	-0.004333333	0.502057767	0.54986084	Non	0.0025	0.005855938	0.016205373	Non	0.007	0.128684545	0.165981918	Non	-0.003204696	0.017387659	0.024662025	Non	-0.0001	0.032075018	0.044403059	Non	-0.000466614	0.012395175	0.013720667	Non	CASZ1	0
chr1	10926121	10928020	1899	cUMR	-0.026866667	0.03486786	0.041222867	Non	0.017666667	0.112177327	0.167211249	Non	-0.016366667	0.246257863	0.296404874	Non	0.019466667	0.462980256	0.542307793	Non	0.029466667	0.282575828	0.331427641	Non	0.004966667	0.789611674	0.803109024	Non	0.039266667	0.048472788	0.064741439	Non	0.007438095	0.20423233	0.20934031	Non	CASZ1	-69389
chr1	10948208	10949257	1049	cUMR	0.039666667	0.00000689	0.0000165	Non	0.4106	0.000000000109	0.00000000362	Hypermethylated	0.216666667	0.0000000386	0.000000308	Hypermethylated	0.1306	0.001793061	0.006285886	Non	0.177	0.000013	0.0000538	Hypermethylated	0.0565	0.005859736	0.009252861	Non	0.4416	0.0000000000481	0.000000000622	Hypermethylated	0.211028571	0.0000000000171	0.0000000000925	Hypermethylated	C1orf127	57273
chr1	10969946	10970295	349	cUMR	-0.087433333	0.030363691	0.036226568	Non	-0.091466667	0.075984924	0.121685965	Non	0.053566667	0.002788	0.005805341	Non	-0.049666667	0.404104163	0.486661775	Non	0.065983333	0.067477549	0.094325307	Non	-0.167016667	0.000967961	0.00182891	Non	0.091533333	0.017158105	0.025197568	Non	-0.024438095	0.000808509	0.00099777	Non	C1orf127	36235
chr1	11001715	11003164	1449	cUMR	-0.050733333	0.284767762	0.299992722	Non	0.038533333	0.397485026	0.469542461	Non	-0.0974	0.00951586	0.017036642	Non	-0.060666667	0.046789898	0.086907889	Non	-0.017066667	0.633773192	0.673101207	Non	-0.049316667	0.002702757	0.004614412	Non	-0.064866667	0.060236802	0.078759889	Non	-0.045409524	0.041569358	0.044289433	Non	C1orf127	3366
chr1	11003850	11004842	992	cUMR	-0.006333333	0.25084916	0.266028388	Non	0.085533333	0.032874441	0.06079627	Non	-0.055333333	0.016440214	0.027555331	Non	-0.050666667	0.059911663	0.106134768	Non	-0.023666667	0.681288332	0.717086098	Non	-0.048666667	0.108844203	0.130682457	Non	-0.084435798	0.0000116	0.0000339	Non	-0.025918131	0.001065178	0.001299482	Non	C1orf127	1688
chr1	11020717	11021766	1049	cUMR	-0.1622	0.00000000862	0.0000000417	Hypomethylated	-0.1689	0.000000171	0.00000167	Hypomethylated	-0.049866667	0.005036894	0.009728225	Non	-0.0585	0.072743302	0.124112722	Non	-0.08945	0.00654608	0.01225794	Non	-0.1772	0.0000046	0.0000152	Hypomethylated	-0.1645	0.000000000881	0.00000000756	Hypomethylated	-0.122814286	0.0000000000738	0.000000000336	Hypomethylated	C1orf127	0
chr1	11071606	11073855	2249	cUMR	-0.079033333	0.000000000464	0.00000000311	Non	-0.038266667	0.004846079	0.012274119	Non	-0.0232	0.0000373	0.000131268	Non	-0.027266667	0.018857036	0.041731733	Non	-0.001116667	0.029071127	0.045165109	Non	-0.050866667	0.000502705	0.001015864	Non	-0.036466667	0.000073	0.00017902	Non	-0.038038095	0.0000000101	0.0000000282	Non	TARDBP	0
chr1	11097342	11098341	999	cUMR	-0.124866667	0.00000000000281	0.0000000000336	Hypomethylated	-0.080533333	0.0000000207	0.000000274	Non	-0.054033333	0.00000057	0.00000327	Non	-0.063733333	0.000123242	0.000712033	Non	-0.099783333	0.000000109	0.00000087	Non	-0.080533333	0.00000671	0.0000213	Non	-0.139133333	0.000000000000502	0.0000000000134	Hypomethylated	-0.091761905	0.00000000000000344	0.0000000000000574	Non	MASP2	0
chr1	11118864	11121363	2499	cUMR	-0.028166667	0.000997017	0.001522664	Non	-0.0153	0.415439714	0.48713242	Non	0.0165	0.422628297	0.473873846	Non	0.0109	0.51395174	0.588988005	Non	-0.03175	0.026327693	0.041427708	Non	-0.047	0.000111708	0.000263942	Non	0.0007	0.699584018	0.723918943	Non	-0.011528571	0.004880001	0.005583168	Non	SRM	0
chr1	11158434	11160483	2049	cUMR	-0.0429	0.002521075	0.003586355	Non	-0.038966667	0.007833249	0.018383113	Non	-0.027733333	0.025350463	0.04032789	Non	-0.033566667	0.075340822	0.127558714	Non	-0.050316667	0.007222735	0.013339884	Non	-0.060816667	0.000508505	0.001026036	Non	-0.032166667	0.061219925	0.079930885	Non	-0.039766667	0.0000122	0.0000191	Non	EXOSC10	0
chr1	11321497	11323696	2199	cUMR	-0.137366667	0.000000000000000444	0.0000000000000144	Hypomethylated	-0.0733	0.0000013	0.00000969	Non	-0.024866667	0.0000000022	0.0000000244	Non	-0.0731	0.000000198	0.00000376	Non	-0.0787	0.0000000699	0.000000598	Non	-0.1222	0.00000000000546	0.0000000000795	Hypomethylated	-0.1421	0.000000000000000888	0.0000000000000773	Hypomethylated	-0.091985714	0.00000000000000022	0.00000000000000546	Non	MTOR	0
chr1	11332576	11334325	1749	cUMR	-0.0325	0.000642753	0.001016677	Non	-0.008066667	0.673664714	0.722764854	Non	0.0095	0.067208221	0.094734658	Non	-0.006066667	0.640823521	0.701106202	Non	-0.004916667	0.57668923	0.619660511	Non	-0.026916667	0.028970084	0.039213677	Non	-0.013066667	0.284002746	0.320410627	Non	-0.011466667	0.021255644	0.023112467	Non	UBIAD1	0
chr1	11379745	11380744	999	cUMR	-0.083233333	0.0000668	0.00012825	Non	0.0383	0.312652297	0.385784342	Non	0.138433333	0.000000652	0.00000368	Hypermethylated	-0.0797	0.0000554	0.000373547	Non	0.17835	0.00000084	0.00000501	Hypermethylated	-0.0424	0.000863201	0.001649054	Non	0.0787	0.0000000245	0.00000014	Non	0.030328571	0.00000000195	0.00000000635	Non	UBIAD1	-31255
chr1	11535898	11541747	5849	Canyon	-0.0249	0.001641484	0.00241007	Non	0.0349	0.088183599	0.137457803	Non	-0.0859	0.00000000342	0.0000000365	Non	0.0085	0.003426227	0.010519203	Non	-0.02915	0.128130089	0.165355266	Non	0.1701	0.00000000574	0.0000000368	Hypermethylated	0.0113	0.015334709	0.022773815	Non	0.004928571	0.000000129	0.000000284	Non	PTCHD2	0
chr1	11610553	11613052	2499	cUMR	-0.208633333	0.00000000000000389	0.0000000000000987	Hypomethylated	-0.183666667	0.0000000000000817	0.0000000000149	Hypomethylated	-0.0428	0.0000000000000218	0.00000000000105	Non	-0.143466667	0.00000000000401	0.00000000102	Hypomethylated	-0.196966667	0.00000000000263	0.000000000159	Hypomethylated	-0.176216667	0.0000000000642	0.000000000671	Hypomethylated	-0.229266667	0.000000000000000444	0.0000000000000453	Hypomethylated	-0.165238095	0.00000000000000022	0.00000000000000546	Hypomethylated	PTCHD2	-12914
chr1	11653927	11654656	729	cUMR	-0.131633333	0.000297657	0.00050017	Hypomethylated	0.065633333	0.080055166	0.126980604	Non	0.184033333	0.000016	0.000062	Hypermethylated	-0.069166667	0.113399034	0.176770161	Non	0.028283333	0.010835082	0.019060063	Non	0.001283333	0.000321152	0.000678597	Non	0.048433333	0.273145343	0.309276359	Non	0.01877619	0.0000107	0.0000169	Non	LOC101929181	14932
chr1	11695986	11696135	149	cUMR	-0.037066667	0.317276604	0.332350863	Non	-0.068633333	0.016018535	0.033456804	Non	0.236933333	0.00000276	0.0000132	Hypermethylated	-0.012033333	0.645167397	0.70493058	Non	0.005016667	0.975604686	0.978691271	Non	0.141516667	0.006854538	0.010657504	Non	-0.063833333	0.186422599	0.219059737	Non	0.030366667	0.000649971	0.000811092	Non	FBXO2	12283
chr1	11701813	11702862	1049	cUMR	-0.206233333	0.000000002	0.0000000113	Hypomethylated	-0.090366667	0.015664706	0.032820836	Non	-0.063233333	0.000310205	0.000842955	Non	-0.008966667	0.010048021	0.025006424	Non	-0.148316667	0.0000235	0.0000899	Hypomethylated	-0.181816667	0.00000118	0.00000449	Hypomethylated	-0.166766667	0.00000000717	0.000000047	Hypomethylated	-0.121938095	0.00000000017	0.000000000714	Hypomethylated	FBXO2	5556
chr1	11705764	11706113	349	cUMR	-0.043328694	0.004433274	0.006047539	Non	-0.001508582	0.844253455	0.87144894	Non	0.004274356	0.697026454	0.731753937	Non	0.042915241	0.109623477	0.171920309	Non	-0.043611112	0.051821115	0.074786515	Non	-0.034369787	0.18859852	0.215589732	Non	0.036562401	0.010265966	0.015791172	Non	-0.004477861	0.005618304	0.006399597	Non	FBXO2	2305
chr1	11714161	11715623	1462	cUMR	-0.0163	0.074474831	0.084250046	Non	0.011866667	0.457020181	0.526475538	Non	0.1027	0.0000305	0.000110165	Hypermethylated	-0.014133333	0.139984777	0.20883024	Non	0.055366667	0.014339242	0.024370341	Non	0.063866667	0.007422793	0.011445768	Non	0.091266667	0.000251755	0.000544795	Non	0.041152381	0.0000756	0.000105887	Non	FBXO44	0
chr1	11723869	11725186	1317	cUMR	-0.034833333	0.000251486	0.00042917	Non	-0.0194	0.103246423	0.156234368	Non	0.231	0.00000000000000133	0.000000000000102	Hypermethylated	-0.02	0.081976085	0.136332622	Non	-0.0165	0.162021112	0.202846257	Non	-0.024	0.050510462	0.064896626	Non	-0.008	0.699491948	0.723854755	Non	0.022228571	0.0000000000703	0.000000000322	Non	FBXO6	0
chr1	11740272	11742471	2199	cUMR	-0.045066667	0.000000233	0.000000787	Non	0.000833333	0.278306448	0.351025935	Non	0.037433333	0.000000517	0.00000299	Non	-0.013966667	0.276035238	0.360200481	Non	-0.008066667	0.628567529	0.668131988	Non	-0.022816667	0.051363885	0.06588773	Non	-0.006566667	0.055836986	0.073569993	Non	-0.007652381	0.00000493	0.00000821	Non	MAD2L2	0
chr1	11750016	11753757	3741	Canyon	-0.015766667	0.014235535	0.017862428	Non	0.08196666699999999	0.000000659	0.00000538	Non	0.072233333	0.0000000997	0.000000703	Non	0.002366667	0.001670808	0.005935889	Non	0.044566667	0.0000674	0.000223666	Non	0.219566667	0.000000000000147	0.00000000000357	Hypermethylated	0.068366667	0.00000022	0.000000978	Non	0.061680952	0.00000000000841	0.0000000000493	Non	MAD2L2	0
chr1	11779299	11780211	912	cUMR	-0.072533333	0.00000523	0.0000128	Non	-0.014633333	0.27605784	0.348935595	Non	0.030966667	0.125286358	0.163763867	Non	-0.042233333	0.000258836	0.001304903	Non	-0.049033333	0.000833495	0.002017434	Non	-0.012783333	0.058722118	0.074352963	Non	-0.043633333	0.001331345	0.002471691	Non	-0.028547619	0.00000671	0.000011	Non	DRAXIN	0
chr1	11795636	11797685	2049	cUMR	-0.027366667	0.000399241	0.000655738	Non	-0.0094	0.433884697	0.504454286	Non	-0.004533333	0.648891569	0.687854178	Non	-0.0012	0.43829938	0.51863347	Non	-0.01745	0.018631882	0.030656226	Non	-0.00545	0.752036371	0.767450346	Non	-0.0108	0.070991178	0.091446778	Non	-0.011142857	0.016548648	0.018144933	Non	AGTRAP	0
chr1	11821940	11822494	554	cUMR	-0.072033333	0.0000005	0.00000156	Non	0.030433333	0.140846992	0.201149171	Non	0.322633333	0.00000000000000022	0.0000000000000218	Hypermethylated	-0.005966667	0.489409392	0.566927248	Non	0.023883333	0.028227509	0.044013362	Non	0.006633333	0.892031341	0.899760213	Non	0.000833333	0.000000554	0.00000223	Non	0.050061905	0.000000000000903	0.00000000000702	Non	C1orf167	0
chr1	11844915	11845914	999	cUMR	-0.002433333	0.000180139	0.000316675	Non	0.067433333	0.008954559	0.020593435	Non	0.0639	0.00000751	0.0000318	Non	0.004433333	0.012100858	0.028937826	Non	0.183483333	0.0000000172	0.000000184	Hypermethylated	0.053233333	0.001340507	0.002450579	Non	0.071633333	0.00663563	0.01066303	Non	0.058090476	0.0000000419	0.000000102	Non	C1orf167	0
chr1	11862397	11867453	5056	Canyon	-0.001666667	0.97822259	0.979059341	Non	0.029333333	0.360916248	0.433376461	Non	0.072833333	0.00473484	0.009214259	Non	-0.035266667	0.133127751	0.200657527	Non	0.009583333	0.743539207	0.773519591	Non	0.003083333	0.647826576	0.669008992	Non	0.072733333	0.021468838	0.030854893	Non	0.023190476	0.066194081	0.06966307	Non	MTHFR	0
chr1	11898723	11899072	349	cUMR	-0.063033333	0.011504949	0.014649736	Non	-0.026333333	0.208343364	0.277956485	Non	-0.084366667	0.000188112	0.000543539	Non	-0.011333333	0.146074669	0.216090078	Non	-0.061783333	0.017134966	0.028500719	Non	-0.021533333	0.064484633	0.080972888	Non	0.121866667	0.0000175	0.0000492	Hypermethylated	-0.022761905	0.000182946	0.000244273	Non	CLCN6	0
chr1	11918770	11920019	1249	cUMR	-0.112133333	0.0000000246	0.000000105	Hypomethylated	0.020566667	0.562738224	0.624122197	Non	0.308866667	0.00000000000698	0.000000000152	Hypermethylated	-0.037433333	0.086833657	0.142707322	Non	-0.052633333	0.102834862	0.136407469	Non	0.110616667	0.000154126	0.000351352	Hypermethylated	0.010166667	0.109453687	0.135313532	Non	0.039395238	0.00000000644	0.0000000187	Non	NPPB	0
chr1	11958333	11959991	1658	cUMR	-0.066166667	0.050385952	0.058293757	Non	0.0184	0.838975998	0.86696705	Non	0.287	0.00000000295	0.0000000319	Hypermethylated	-0.048	0.081191221	0.135251351	Non	-0.015	0.897795763	0.911324777	Non	0.12775	0.002839081	0.004815138	Non	0.0682	0.000341022	0.000717466	Non	0.056257143	0.00000164	0.00000296	Non	KIAA2013	19654
chr1	11967216	11970166	2950	cUMR	-0.010166667	0.14295947	0.15606058	Non	0.018533333	0.019020362	0.03858892	Non	0.027333333	0.001106103	0.002571126	Non	0.009933333	0.197225717	0.275043133	Non	0.019333333	0.04298453	0.063591454	Non	0.000583333	0.601066269	0.62414336	Non	0.011333333	0.23799941	0.273357697	Non	0.010904762	0.012600374	0.013936926	Non	KIAA2013	9479
chr1	11984745	11986994	2249	cUMR	-0.0467	0.080455264	0.090628287	Non	-0.0153	0.216371355	0.286793209	Non	-0.016533333	0.677087959	0.713631283	Non	-0.0147	0.368951652	0.45336785	Non	0.00305	0.989787866	0.991186337	Non	-0.0212	0.696232219	0.714922619	Non	-0.0359	0.156419075	0.186883877	Non	-0.022328571	0.167014748	0.171857099	Non	KIAA2013	0
chr1	11989699	11990098	399	cUMR	-0.028133333	0.209053715	0.223603037	Non	0.048566667	0.401320529	0.473516173	Non	-0.046633333	0.170049871	0.214222478	Non	0.033766667	0.427061284	0.508464566	Non	0.021616667	0.849185902	0.867914237	Non	0.025116667	0.421138351	0.450088344	Non	0.029601486	0.0000258	0.0000699	Non	0.008513385	0.007370125	0.008309407	Non	KIAA2013	-3215
chr1	11994054	11995303	1249	cUMR	-0.018370866	0.009943252	0.012783424	Non	0.018566667	0.270862283	0.343669357	Non	0.0116	0.610353485	0.652079194	Non	-0.011633333	0.233305766	0.314898754	Non	0.001766667	0.992195889	0.993143781	Non	-0.012733333	0.106501218	0.128080975	Non	-0.008578155	0.018764166999999998	0.027306521	Non	-0.002649988	0.17380219	0.178673486	Non	PLOD1	0
chr1	12039244	12042343	3099	cUMR	-0.172333333	0.0000000000214	0.000000000205	Hypomethylated	-0.0759	0.00042022	0.001514968	Non	0.007666667	0.917947084	0.928543375	Non	-0.0755	0.000447665	0.00205092	Non	-0.16575	0.0000000035	0.0000000496	Hypomethylated	-0.1015	0.00039083	0.000809196	Hypomethylated	-0.1077	0.0000315	0.0000839	Hypomethylated	-0.095785714	0.000000000572	0.00000000212	Non	MFN2	0
chr1	12078915	12080493	1578	cUMR	-0.042133333	0.0000311	0.0000641	Non	-0.022166667	0.002406498	0.006750015	Non	-0.0163	0.21182599	0.259854547	Non	-0.017566667	0.131843713	0.199095739	Non	-0.014966667	0.251979999	0.299286017	Non	-0.030466667	0.015461808	0.022193022	Non	-0.028566667	0.015493644	0.022988599	Non	-0.024966667	0.0000193	0.0000295	Non	MIIP	0
chr1	12123105	12124304	1199	cUMR	0.107466667	0.00000193	0.00000524	Hypermethylated	0.2156	0.000000000342	0.00000000909	Hypermethylated	0.146466667	0.0000000514	0.000000396	Hypermethylated	0.0178	0.291947345	0.376564102	Non	0.21055	0.00000000188	0.0000000298	Hypermethylated	0.40105	0.0000000000000141	0.000000000000491	Hypermethylated	0.4306	0.00000000000000178	0.000000000000133	Hypermethylated	0.208285714	0.000000000000000777	0.0000000000000163	Hypermethylated	TNFRSF8	0
chr1	12184835	12185371	536	cUMR	-0.112566667	0.00000698	0.0000167	Hypomethylated	0.010333333	0.171432695	0.23628746	Non	-0.036233333	0.178020811	0.222877468	Non	-0.094666667	0.004276382	0.012582693	Non	-0.027816667	0.145439369	0.184774247	Non	-0.060316667	0.136709871	0.16075117	Non	-0.035666667	0.241036232	0.276506025	Non	-0.05272381	0.000381592	0.000489518	Non	TNFRSF8	0
chr1	12226757	12228001	1244	cUMR	0.013266667	0.286298518	0.301499877	Non	0.140533333	0.000107785	0.000464716	Hypermethylated	0.1021	0.000378743	0.001002411	Non	-0.026666667	0.311691841	0.396848417	Non	0.098933333	0.006331488	0.011917222	Non	0.033933333	0.120349926	0.14320524	Non	0.222933333	0.000000324	0.00000138	Hypermethylated	0.08307619	0.00000303	0.00000521	Non	MIR7846	0
chr1	12268870	12269569	699	cUMR	-0.058333333	0.014406264	0.018064427	Non	-0.0311	0.059360461	0.099244364	Non	-0.168833333	0.0000000297	0.000000244	Hypomethylated	-0.1041	0.0012178	0.004612784	Non	-0.0555	0.152173296	0.192140882	Non	-0.05775	0.167439836	0.193365849	Non	0.0301	0.358370357	0.395370731	Non	-0.0669	0.000019	0.0000291	Non	TNFRSF1B	0
chr1	12289711	12291810	2099	cUMR	-0.02	0.072074445	0.081630398	Non	-0.0085	0.006952888	0.016586847	Non	-0.01	0.407859979	0.4595749	Non	-0.0137	0.369931567	0.454270836	Non	-0.0095	0.648423411	0.686421866	Non	-0.01025	0.055161674	0.070287967	Non	-0.0315	0.00080684	0.001567241	Non	-0.015071429	0.010253092	0.011421393	Non	VPS13D	0
chr1	12654900	12656849	1949	cUMR	-0.085566667	0.0000898	0.000167955	Non	0.027833333	0.281563295	0.35442881	Non	0.057433333	0.120350818	0.157989541	Non	-0.012566667	0.28264377	0.367059149	Non	0.073683333	0.0000131	0.000054	Non	-0.021316667	0.198945486	0.22649017	Non	0.066233333	0.0000000144	0.0000000874	Non	0.012804762	0.0000013	0.00000239	Non	DHRS3	0
chr1	12674913	12680712	5799	Canyon	-0.047933333	0.001409799	0.002096214	Non	0.020166667	0.503303656	0.569474959	Non	0.3154	0.000000000000661	0.0000000000195	Hypermethylated	-0.026633333	0.173539727	0.248084496	Non	-0.048433333	0.006867265	0.012775001	Non	-0.044183333	0.000952897	0.00180271	Non	-0.028433333	0.202852878	0.236360878	Non	0.030280952	0.000015	0.0000233	Non	DHRS3	0
chr1	12718690	12719340	650	cUMR	-0.1538	0.00000000245	0.0000000136	Hypomethylated	-0.0806	0.001251331	0.003843637	Non	-0.0813	0.002179036	0.00466469	Non	-0.0856	0.003616304	0.011004634	Non	-0.1128	0.000000196	0.00000146	Hypomethylated	-0.16105	0.00000229	0.00000816	Hypomethylated	-0.0014	0.640366878	0.668382582	Non	-0.095542857	0.00000000343	0.0000000106	Non	AADACL4	0
chr1	13177381	13178230	849	cUMR	-0.5128	0.00000000000000022	0.00000000000000775	Hypomethylated	-0.2969	0.0000000231	0.000000301	Hypomethylated	-0.3433	0.0000000000369	0.000000000648	Hypomethylated	-0.2923	0.00000000134	0.0000000722	Hypomethylated	-0.3783	0.000000000303	0.00000000685	Hypomethylated	-0.36755	0.000000000237	0.0000000021	Hypomethylated	-0.3423	0.000000000026	0.000000000372	Hypomethylated	-0.365071429	0.00000000000000022	0.00000000000000546	Hypomethylated	HNRNPCL2	4730
chr1	13838817	13841276	2459	cUMR	0.107366667	0.00000578	0.000014	Hypermethylated	0.094166667	0.000230717	0.000896417	Non	-0.002633333	0.416852794	0.468291391	Non	0.014766667	0.273840748	0.357864118	Non	0.032116667	0.082799819	0.112812237	Non	0.364366667	0.00000000000181	0.0000000000308	Hypermethylated	0.228166667	0.00000000192	0.0000000149	Hypermethylated	0.11142381	0.000000000426	0.00000000162	Hypermethylated	LRRC38	0
chr1	13843701	13844450	749	cUMR	-0.194966667	0.0000000321	0.000000134	Hypomethylated	-0.125166667	0.000516582	0.001806687	Non	0.027866667	0.000014	0.0000549	Non	-0.166566667	0.000001	0.0000139	Hypomethylated	-0.055716667	0.010408791	0.018377246	Non	0.018283333	0.001859378	0.003297077	Non	-0.169166667	0.00000435	0.0000141	Hypomethylated	-0.098766667	0.00000000647	0.0000000188	Non	LRRC38	-3460
chr1	13909773	13911197	1424	cUMR	0.046333333	0.106021308	0.117884072	Non	0.325266667	0.00000000174	0.0000000339	Hypermethylated	0.279166667	0.0000000149	0.000000133	Hypermethylated	0.034066667	0.437030506	0.5175201	Non	0.192666667	0.0000181	0.0000717	Hypermethylated	0.415166667	0.000000000268	0.00000000234	Hypermethylated	0.377866667	0.000000000628	0.00000000566	Hypermethylated	0.230580952	0.00000000000868	0.0000000000507	Hypermethylated	PDPN	0
chr1	13991947	13992541	594	cUMR	-0.1267	0.000000166	0.00000058	Hypomethylated	-0.036133333	0.030466163	0.057202987	Non	-0.0437	0.044052268	0.065452343	Non	-0.048133333	0.075070602	0.127242437	Non	-0.094033333	0.000745622	0.001832162	Non	-0.096783333	0.0000923	0.000222504	Non	-0.050333333	0.00000016	0.000000739	Non	-0.070247619	0.0000000529	0.000000126	Non	PRDM2	34194
chr1	14026036	14027735	1699	cUMR	-0.043133333	0.005645905	0.007564495	Non	-0.032566667	0.09176471	0.141839048	Non	0.087866667	0.000780817	0.001890117	Non	-0.012166667	0.214095459	0.294160008	Non	-0.002966667	0.855837382	0.873822948	Non	0.321283333	0.0000000000378	0.00000000042	Hypermethylated	0.101833333	0.001575019	0.002872487	Non	0.052204762	0.000000115	0.000000255	Non	PRDM2	0
chr1	14075115	14077414	2299	cUMR	-0.0149	0.00000741	0.0000176	Non	-0.009666667	0.031891488	0.059388654	Non	-0.006066667	0.080757633	0.111288239	Non	-0.006266667	0.13243529	0.199857462	Non	-0.011066667	0.016298991	0.027292324	Non	-0.005066667	0.305820227	0.335977015	Non	-0.006443711	0.00019453	0.00043174	Non	-0.008635024	0.0000137	0.0000214	Non	PRDM2	0
chr1	14218777	14220226	1449	cUMR	0.022333333	0.00519664	0.007005797	Non	0.071333333	0.0000748	0.000337481	Non	0.003166667	0.589636903	0.632920717	Non	0.031733333	0.00136793	0.005063654	Non	0.272833333	0.000000000000105	0.0000000000143	Hypermethylated	0.275333333	0.000000000000767	0.0000000000147	Hypermethylated	0.161533333	0.000000000651	0.00000000583	Hypermethylated	0.104819048	0.000000000000697	0.0000000000056	Hypermethylated	PRDM2	-67204
chr1	14286847	14287230	383	cUMR	-0.109233333	0.0000000000137	0.000000000138	Hypomethylated	-0.053166667	0.000194296	0.000772983	Non	-0.032566667	0.000964342	0.002281661	Non	-0.063766667	0.0000946	0.00057159	Non	-0.055816667	0.0000561	0.000191232	Non	-0.036816667	0.032329057	0.043336798	Non	-0.069966667	0.000000372	0.00000156	Non	-0.061595238	0.0000000000598	0.000000000278	Non	PRDM2	-135274
chr1	14292231	14292604	373	cUMR	-0.0552	0.062068518	0.070900983	Non	-0.027666667	0.522989718	0.587336685	Non	-0.066533333	0.092324506	0.125110271	Non	0.045933333	0.476719066	0.555403986	Non	-0.065366667	0.226133507	0.272182471	Non	-0.064616667	0.212894718	0.241075933	Non	-0.011066667	0.05633413	0.074152038	Non	-0.034695238	0.062585669	0.065983701	Non	PRDM2	-140658
chr1	14924042	14927491	3449	cUMR	-0.015866667	0.541650242	0.554042511	Non	0.0354	0.001249481	0.003839421	Non	-0.0467	0.001378315	0.003125789	Non	0.0106	0.583156561	0.650206209	Non	0.05405	0.051884726	0.074860383	Non	0.5028	0.00000000000000755	0.000000000000291	Hypermethylated	0.0302	0.298947392	0.335777576	Non	0.0638	0.0000000229	0.0000000592	Non	KAZN	0
chr1	15250223	15252174	1951	cUMR	-0.012	0.0000354	0.000072	Non	0.0046	0.621175601	0.67574685	Non	0.000666667	0.247214791	0.297411077	Non	-0.0048	0.45576465	0.535286897	Non	0.0005	0.919288393	0.92931503	Non	0.11375	0.000000000000654	0.0000000000129	Hypermethylated	0.009433464	0.00000259	0.00000881	Non	0.012434029	0.00000000182	0.00000000597	Non	KAZN	0
chr1	15372972	15373321	349	cUMR	-0.080933333	0.013597551	0.017116265	Non	0.006	0.909092815	0.925727247	Non	-0.029266667	0.218292243	0.26676842	Non	-0.0476	0.032717372	0.064997793	Non	-0.0926	0.000278063	0.000772689	Non	-0.0241	0.754370897	0.76970234	Non	0.0582	0.015978325	0.023643682	Non	-0.029857143	0.001001289	0.001224391	Non	KAZN	0
chr1	15426381	15428080	1699	cUMR	-0.2335	0.00000000798	0.0000000389	Hypomethylated	-0.068133333	0.20233277	0.271319575	Non	0.077833333	0.121165736	0.158972717	Non	-0.035333333	0.443931975	0.52407156	Non	-0.139333333	0.0000119	0.0000496	Hypomethylated	-0.222333333	0.0000016	0.0000059	Hypomethylated	-0.135533333	0.000965549	0.001844741	Non	-0.102161905	0.000000157	0.000000341	Hypomethylated	KAZN	0
chr1	15477771	15481570	3799	Canyon	-0.027566667	0.000119202	0.000217303	Non	0.024033333	0.051739812	0.088461349	Non	0.031766667	0.000542563	0.001373681	Non	-0.009366667	0.337883386	0.423131208	Non	-0.004316667	0.002829608	0.005869665	Non	-0.018816667	0.022747649	0.031509619	Non	0.055633333	0.0000429	0.000110876	Non	0.008119048	0.0000221	0.0000335	Non	TMEM51	0
chr1	15573585	15574434	849	cUMR	-0.023409841	0.0000797	0.000150524	Non	-0.007133333	0.591395666	0.649348074	Non	-0.009933333	0.545258062	0.590943502	Non	-0.032733333	0.0000432	0.000304619	Non	0.013816667	0.197825053	0.242198249	Non	0.023566667	0.005081358	0.008130847	Non	-0.010133333	0.634424964	0.662812345	Non	-0.00858751	0.001764731	0.002104126	Non	FHAD1	0
chr1	15598811	15599610	799	cUMR	0.016166667	0.378645359	0.393505083	Non	0.042733333	0.040068037	0.071447013	Non	-0.093	0.0000378	0.000132652	Non	-0.065466667	0.008227021	0.021267404	Non	0.057083333	0.001254665	0.002874588	Non	-0.093916667	0.000997983	0.001880474	Non	0.034933333	0.00000362	0.0000119	Non	-0.015638095	0.00000165	0.00000298	Non	FHAD1	0
chr1	15631300	15631649	349	cUMR	-0.017178202	0.000357674	0.000592549	Non	-0.033666667	0.170541368	0.235328142	Non	0.039133333	0.159050959	0.202259104	Non	-0.018066667	0.531695203	0.604583546	Non	-0.028866667	0.360641158	0.410424115	Non	-0.021366667	0.156616921	0.181957119	Non	0.009133333	0.61205272	0.64116261	Non	-0.008062312	0.031627346	0.033946009	Non	FHAD1	0
chr1	15649948	15650547	599	cUMR	-0.023266667	0.232141861	0.247244523	Non	-0.025033333	0.10464693	0.157981735	Non	0.013566667	0.450292457	0.50051419	Non	-0.049033333	0.023854899	0.050412642	Non	-0.014433333	0.895119969	0.909039614	Non	-0.067183333	0.004699501	0.007578704	Non	0.000366667	0.44102914	0.476591539	Non	-0.021519048	0.3393418	0.344618765	Non	FHAD1	0
chr1	15735409	15739615	4206	Canyon	-0.098933333	0.000241035	0.000413208	Non	0.022733333	0.031084373	0.058132723	Non	-0.038433333	0.168839156	0.21290947	Non	0.004533333	0.219340094	0.299963543	Non	-0.040266667	0.284041469	0.333016995	Non	-0.111516667	0.000072	0.000178278	Hypomethylated	0.073933333	0.003898631	0.00657807	Non	-0.026438095	0.029707758	0.031938325	Non	EFHD2	0
chr1	15757636	15757935	299	cUMR	0.016466667	0.838900625	0.843943694	Non	-0.0174	0.269770738	0.342565461	Non	-0.0727	0.018683641	0.030876312	Non	-0.0382	0.358917402	0.443434584	Non	0.09855	0.032465332	0.049717181	Non	0.0138	0.661057118	0.681533089	Non	0.1356	0.0000718	0.00017629	Hypermethylated	0.014628571	0.005709024	0.00649894	Non	EFHD2	-798
chr1	15849941	15854290	4349	Canyon	-0.015733333	0.0000058	0.0000141	Non	-0.008533333	0.026224118	0.050517433	Non	-0.012733333	0.000529918	0.001345766	Non	-0.012333333	0.001388152	0.00512277	Non	-0.005983333	0.10455133	0.138379631	Non	-0.013733333	0.000789356	0.001523703	Non	-0.010533333	0.001549387	0.002832842	Non	-0.011619048	0.000000109	0.000000243	Non	CASP9	0
chr1	15910830	15911796	966	cUMR	-0.0373	0.0000259	0.0000543	Non	0.0000667	0.848676489	0.874892265	Non	0.173033333	0.000000000000302	0.00000000000996	Hypermethylated	-0.008533333	0.622590206	0.684545275	Non	0.220366667	0.00000000000000488	0.0000000000014	Hypermethylated	-0.038633333	0.0000572	0.000144893	Non	0.080266667	0.000000000103	0.00000000121	Non	0.054295238	0.0000000000000455	0.000000000000525	Non	AGMAT	0
chr1	15928881	15930580	1699	cUMR	-0.090333333	0.000000266	0.000000887	Non	-0.036066667	0.017943629	0.036786425	Non	-0.023333333	0.367144683	0.419469589	Non	-0.069866667	0.000199616	0.001052591	Non	-0.078916667	0.00014143	0.000427036	Non	-0.045916667	0.014232072	0.020589811	Non	-0.070266667	0.0000799	0.000194415	Non	-0.05892381	0.0000000621	0.000000146	Non	DDI2	13373
chr1	15943123	15945722	2599	cUMR	-0.0327	0.000159663	0.000283848	Non	-0.0291	0.001336747	0.004070717	Non	0.002966667	0.269175545	0.320357185	Non	-0.0289	0.001761719	0.006202176	Non	-0.0282	0.009897529	0.017580206	Non	-0.0227	0.002227057	0.003871843	Non	-0.0319	0.000724892	0.00142251	Non	-0.023757143	0.00000434	0.00000729	Non	DDI2	0
chr1	16010142	16012347	2205	cUMR	-0.028633333	0.027988301	0.033583731	Non	-0.014366667	0.2912886	0.364405638	Non	-0.023466667	0.03051777	0.047403456	Non	-0.016966667	0.152997118	0.22441564	Non	-0.013716667	0.44920551	0.498225911	Non	-0.023966667	0.160573268	0.18616765	Non	-0.004966667	0.13538859	0.164059516	Non	-0.01842381	0.005626428	0.006408548	Non	PLEKHM2	0
chr1	16084022	16086262	2240	cUMR	0.048433333	0.001863044	0.00271027	Non	0.031	0.379445093	0.451709285	Non	0.085933333	0.002872139	0.005951419	Non	-0.0286	0.199893642	0.278119918	Non	0.0286	0.012887215	0.022190504	Non	0.1486	0.000000941	0.00000368	Hypermethylated	0.0288	0.083336137	0.105637727	Non	0.047742857	0.0000199	0.0000303	Non	FBLIM1	0
chr1	16118403	16118887	484	cUMR	-0.060066667	0.00000000117	0.00000000701	Non	-0.024366667	0.071074729	0.115301451	Non	0.031266667	0.000773143	0.00187399	Non	-0.004766667	0.000932211	0.003706388	Non	-0.027816667	0.050430661	0.073029149	Non	-0.063316667	0.0000357	0.000095	Non	-0.054966667	0.0000228	0.0000627	Non	-0.027366667	0.0000000131	0.0000000357	Non	FBLIM1	-5320
chr1	16141866	16142315	449	cUMR	-0.010433653	0.003017914	0.004235133	Non	0.002801244	0.001593565	0.004741342	Non	0.058239415	0.000192651	0.000554593	Non	0.075802697	0.039699271	0.075992526	Non	0.043651559	0.004346968	0.008545705	Non	0.035398044	0.002190639	0.003817618	Non	0.13780773	0.089285751	0.112504808	Non	0.048143201	0.000000515	0.00000102	Non	UQCRHL	-7673
chr1	16159468	16163067	3599	Canyon	-0.0643	0.00000067	0.00000203	Non	-0.054733333	0.0000281	0.000142781	Non	-0.009966667	0.504219361	0.55195231	Non	-0.047533333	0.002632894	0.008547792	Non	-0.054633333	0.000936625	0.002230976	Non	-0.056383333	0.000144129	0.000331605	Non	-0.024333333	0.014429167	0.021571237	Non	-0.043504762	0.000000277	0.000000572	Non	FLJ37453	0
chr1	16173095	16177894	4799	Canyon	-0.010633333	0.009983008	0.012828375	Non	-0.0087	0.09569368	0.14664555	Non	-0.004633333	0.180964292	0.226174862	Non	-0.0113	0.004929749	0.014154305	Non	-0.01155	0.018397444	0.030324323	Non	-0.0053	0.371322806	0.401063914	Non	-0.0081	0.12273845	0.150134814	Non	-0.008557143	0.000128237	0.000174581	Non	FLJ37453	0
chr1	16301317	16302966	1649	cUMR	-0.0359	0.001492862	0.002208003	Non	-0.014466667	0.405885131	0.47798915	Non	-0.018566667	0.108130274	0.143621416	Non	-0.006866667	0.122369296	0.187526568	Non	-0.010566667	0.240534406	0.287260791	Non	-0.024066667	0.138221892	0.162394381	Non	0.008933333	0.680409477	0.70583549	Non	-0.015066667	0.026169895	0.028230544	Non	ZBTB17	0
chr1	16335504	16336028	524	cUMR	-0.140866667	0.000000586	0.0000018	Hypomethylated	-0.025366667	0.607308702	0.663412734	Non	-0.121866667	0.0000156	0.0000606	Hypomethylated	-0.096566667	0.002026273	0.006924152	Non	-0.032116667	0.359558606	0.40936612	Non	-0.113366667	0.000227101	0.000497382	Hypomethylated	0.074833333	0.019384485	0.028107386	Non	-0.068395238	0.000000668	0.00000129	Non	C1orf64	-2321
chr1	16339082	16340578	1496	cUMR	-0.0609	0.018690554	0.023039796	Non	-0.010433333	0.926303082	0.940549695	Non	0.1066	0.001444802	0.003260919	Non	-0.049033333	0.073788238	0.12548687	Non	0.013016667	0.049194978	0.071480288	Non	-0.036983333	0.291954681	0.321948708	Non	-0.044833333	0.017196906	0.025248394	Non	-0.009804762	0.002993995	0.003494589	Non	HSPB7	0
chr1	16353065	16354064	999	cUMR	-0.015733333	0.684929449	0.694351515	Non	0.089233333	0.023392199	0.045932352	Non	0.0671	0.065508149	0.09265806	Non	0.003633333	0.436020547	0.516579308	Non	0.126683333	0.0000407	0.000144287	Hypermethylated	0.081933333	0.046240711	0.059915509	Non	0.058833333	0.043296414	0.058400716	Non	0.054319048	0.000500176	0.000632816	Non	CLCNKA	0
chr1	16374871	16375720	849	cUMR	-0.0227	0.02024145	0.024796831	Non	0.152333333	0.0000307	0.000154383	Hypermethylated	0.051133333	0.024775811	0.039555286	Non	-0.033666667	0.347021305	0.4317699	Non	0.205633333	0.00000024	0.00000172	Hypermethylated	0.093883333	0.007649828	0.011753733	Non	0.008154519	0.0000000108	0.0000000678	Non	0.057223732	0.0000000513	0.000000123	Non	CLCNKB	0
chr1	16382350	16382988	638	cUMR	-0.0681	0.020588905	0.025199422	Non	0.104766667	0.005358394	0.013345861	Non	0.038733333	0.051545567	0.075158533	Non	-0.060833333	0.102177949	0.162559202	Non	-0.062433333	0.121989133	0.158345549	Non	-0.031183333	0.517305055	0.544177459	Non	-0.042033333	0.00000185	0.00000651	Non	-0.015461905	0.0000772	0.000108016	Non	CLCNKB	0
chr1	16399223	16400654	1431	cUMR	-0.034733333	0.000906633	0.001394793	Non	0.0254	0.181639157	0.248014436	Non	0.111266667	0.0000000298	0.000000245	Hypermethylated	-0.0088	0.393701461	0.476994359	Non	-0.0154	0.441703373	0.490899224	Non	0.02885	0.027301613	0.037176599	Non	0.0308	0.084573445	0.107025983	Non	0.021428571	0.00000816	0.0000132	Non	FAM131C	0
chr1	16405027	16405926	899	cUMR	-0.1223	0.00000229	0.00000612	Hypomethylated	-0.036066667	0.022091461	0.043745413	Non	-0.0983	0.000462699	0.001195852	Non	-0.051266667	0.026510097	0.054925657	Non	-0.088466667	0.010063069	0.017849244	Non	-0.092716667	0.006367718	0.009964238	Non	0.022133333	0.008440336	0.013233024	Non	-0.067838095	0.00000663	0.0000108	Non	FAM131C	-4901
chr1	16479312	16482904	3592	Canyon	-0.080766667	0.0000000179	0.0000000799	Non	0.049033333	0.009372252	0.021410993	Non	0.176733333	0.00000000217	0.0000000242	Hypermethylated	-0.028766667	0.144024456	0.213662059	Non	-0.038266667	0.00000194	0.0000103	Non	-0.076766667	0.0000378	0.0000999	Non	-0.051766667	0.001228649	0.002298387	Non	-0.001195238	0.00000000699	0.0000000201	Non	EPHA2	0
chr1	16493715	16494864	1149	cUMR	-0.093933333	0.00000379	0.00000957	Non	-0.0409	0.102889322	0.155844113	Non	0.1804	0.000000102	0.000000719	Hypermethylated	-0.0871	0.0000182	0.000149556	Non	0.0654	0.0000383	0.000137044	Non	-0.03385	0.384775718	0.414461168	Non	-0.0697	0.000216141	0.000474892	Non	-0.009814286	0.000000638	0.00000124	Non	EPHA2	-11134
chr1	16513927	16514885	958	cUMR	-0.1614	0.0000000104	0.0000000493	Hypomethylated	-0.069733333	0.030607075	0.057422819	Non	-0.033233333	0.089546058	0.121714756	Non	-0.129933333	0.0000172	0.000142865	Hypomethylated	-0.150233333	0.00000343	0.0000168	Hypomethylated	-0.148983333	0.000000959	0.00000374	Hypomethylated	-0.015333333	0.146836697	0.176590989	Non	-0.09827619	0.0000000136	0.0000000367	Non	ARHGEF19	9714
chr1	16532532	16534231	1699	cUMR	-0.061066667	0.001117719	0.001692204	Non	0.031766667	0.020268178	0.040710369	Non	0.1781	0.000000292	0.00000181	Hypermethylated	-0.017633333	0.66459664	0.721676722	Non	0.138266667	0.000000108	0.000000864	Hypermethylated	-0.015983333	0.671529243	0.691538495	Non	0.065366667	0.030122832	0.041970612	Non	0.045395238	0.00000206	0.00000365	Non	ARHGEF19	0
chr1	16542463	16544446	1983	cUMR	-0.0305	0.04987538	0.057750187	Non	0.1717	0.00000418	0.0000267	Hypermethylated	0.212833333	0.0000000085	0.0000000819	Hypermethylated	-0.0229	0.38616265	0.469868596	Non	0.0905	0.005938596	0.011267482	Non	-0.0075	0.302750983	0.332908656	Non	0.0119	0.055644185	0.073328967	Non	0.0637	0.000000766	0.00000146	Non	ARHGEF19	-3360
chr1	16562643	16564192	1549	cUMR	-0.026966667	0.000000132	0.000000474	Non	-0.019566667	0.000336483	0.001247612	Non	-0.005133333	0.111752822	0.147813794	Non	-0.016966667	0.000646263	0.002754445	Non	-0.021466667	0.00054432	0.001395824	Non	-0.016466667	0.001999546	0.003520756	Non	-0.015966667	0.00279555	0.004853839	Non	-0.017338095	0.0000000441	0.000000107	Non	RSG1	0
chr1	16570899	16571110	211	cUMR	-0.005150601	0.552604764	0.564816117	Non	-0.006056105	0.461289799	0.530730275	Non	-0.000216218	0.988079299	0.989751685	Non	-0.000258052	0.797235286	0.835149362	Non	0.003785852	0.915815835	0.926659808	Non	-0.004433644	0.757559051	0.772628154	Non	0.035312195	0.045025692	0.060499586	Non	0.00314659	0.373110706	0.378291055	Non	FBXO42	5449
chr1	16676905	16679539	2634	cUMR	-0.047866667	0.00000183	0.00000499	Non	-0.027533333	0.017046815	0.035238663	Non	-0.007366667	0.457185955	0.507217308	Non	-0.017533333	0.041829241	0.079337271	Non	-0.041283333	0.000851178	0.002055484	Non	-0.028533333	0.036031372	0.047831433	Non	-0.030533333	0.010997762	0.016814852	Non	-0.028247619	0.00000629	0.0000103	Non	FBXO42	0
chr1	16693202	16695323	2121	cUMR	-0.013866667	0.001065044	0.00161927	Non	-0.006433333	0.369022793	0.441482585	Non	0.0038	0.347169344	0.399736578	Non	-0.003233333	0.356379909	0.440955179	Non	-0.009283333	0.1289096	0.166245506	Non	-0.014783333	0.000181602	0.000407169	Non	-0.005033333	0.563817944	0.595344311	Non	-0.00657619	0.011235987	0.012481668	Non	SZRD1	0
chr1	16766618	16768267	1649	cUMR	-0.037166667	0.0000132	0.0000296	Non	-0.0129	0.293294864	0.366420902	Non	0.001333333	0.066684996	0.094123764	Non	-0.0149	0.067739977	0.11709332	Non	-0.02875	0.002820134	0.005852532	Non	-0.0385	0.0000562	0.000142607	Non	0.0005	0.134767978	0.163438986	Non	-0.017728571	0.0000325	0.000048	Non	NECAP2	0
chr1	16824714	16826520	1806	cUMR	-0.014566667	0.059248899	0.067869942	Non	-0.003733333	0.319269698	0.392102437	Non	-0.009233333	0.424084669	0.475153075	Non	0.007266667	0.61524466	0.677953533	Non	-0.005233333	0.731352337	0.762355248	Non	0.030766667	0.029976586	0.040428463	Non	0.011889021	0.00059113	0.001184028	Non	0.001042138	0.016259776	0.017837123	Non	CROCCP3	-5519
chr1	16839395	16841944	2549	cUMR	-0.095366667	0.000000000000905	0.0000000000123	Non	-0.043666667	0.000154759	0.000634748	Non	0.018133333	0.025584271	0.040660143	Non	-0.058266667	0.0000214	0.000170304	Non	-0.042616667	0.00000642	0.0000289	Non	-0.043366667	0.00000592	0.0000191	Non	-0.059666667	0.0000000605	0.000000311	Non	-0.046152381	0.000000000034	0.000000000168	Non	CROCCP3	-20200
chr1	16860768	16862067	1299	cUMR	0.2277	0.000331938	0.00055326	Hypermethylated	0.3106	0.0000217	0.000113353	Hypermethylated	0.2552	0.000115077	0.000353265	Hypermethylated	0.055	0.377834524	0.462021432	Non	0.33245	0.0000167	0.0000668	Hypermethylated	0.3532	0.0000128	0.0000378	Hypermethylated	0.2154	0.001126111	0.002122392	Non	0.244142857	0.00000000587	0.0000000172	Hypermethylated	MIR3675	13342
chr1	16918083	16918717	634	cUMR	-0.012566667	0.629977694	0.640746316	Non	0.003	0.767234256	0.805438872	Non	0.000933333	0.604002967	0.646354789	Non	-0.0188	0.367440931	0.451949344	Non	0.1346	0.002790789	0.005799623	Non	-0.0544	0.085353104	0.104615183	Non	0.019	0.173879762	0.205796294	Non	0.007628571	0.045806492	0.048700469	Non	NBPF1	0
chr1	16938833	16940673	1840	cUMR	-0.006866667	0.063812042	0.072730576	Non	0.0154	0.006685114	0.016056133	Non	-0.005033333	0.009741802	0.017381752	Non	0.004	0.522962626	0.596975385	Non	0.0038	0.561696016	0.605789052	Non	0.0223	0.000043	0.00011204	Non	0.0042	0.520559858	0.553646289	Non	0.004314286	0.00487664	0.005580117	Non	NBPF1	0
chr1	16970370	16971619	1249	cUMR	-0.0429	0.000000326	0.00000106	Non	-0.0282	0.000685431	0.002298153	Non	0.005266667	0.029098681	0.045536724	Non	-0.026	0.01896758	0.041925655	Non	-0.04215	0.0000239	0.0000912	Non	-0.03565	0.00117589	0.002178732	Non	-0.0266	0.019879802	0.02875465	Non	-0.026885714	0.00000171	0.00000308	Non	MST1P2	450
chr1	17025562	17028111	2549	cUMR	-0.005166667	0.282313818	0.297524639	Non	0.271566667	0.00000101	0.00000778	Hypermethylated	0.065833333	0.028808733	0.045138677	Non	0.147766667	0.001404201	0.005168716	Non	0.178916667	0.0000574	0.000194903	Hypermethylated	0.185166667	0.000440881	0.000900989	Hypermethylated	0.188566667	0.0000554	0.000139332	Hypermethylated	0.138852381	0.000000426	0.000000854	Hypermethylated	ESPNP	0
chr1	17033827	17034926	1099	cUMR	-0.0102	0.006175233	0.008218411	Non	0.0314	0.008949252	0.020589089	Non	0.0798	0.000000158	0.00000106	Non	-0.0112	0.004497639	0.013120257	Non	0.00605	0.851554203	0.870002679	Non	0.1098	0.000000000628	0.00000000501	Hypermethylated	0.0642	0.0000871	0.000209597	Non	0.037228571	0.00000000786	0.0000000224	Non	ESPNP	0
chr1	17199012	17200161	1149	cUMR	0.168666667	0.004243083	0.005808988	Non	0.2286	0.002096617	0.006007394	Non	0.2745	0.000119529	0.000364792	Hypermethylated	0.0288	0.806848231	0.843642498	Non	0.301	0.000134779	0.000409676	Hypermethylated	0.2945	0.000439389	0.000898236	Hypermethylated	0.2066	0.00483908	0.007991397	Non	0.210314286	0.00000034	0.000000694	Hypermethylated	MIR3675	-13497
chr1	17214788	17216387	1599	cUMR	-0.059233333	0.022170881	0.026999893	Non	0.158133333	0.000248118	0.000952898	Hypermethylated	0.2156	0.00000123	0.00000641	Hypermethylated	-0.028066667	0.484892855	0.562940117	Non	0.242433333	0.0000000168	0.000000181	Hypermethylated	0.272183333	0.000000787	0.00000313	Hypermethylated	0.213933333	0.0000167	0.0000472	Hypermethylated	0.134761905	0.00000000612	0.0000000178	Hypermethylated	MIR3675	-29273
chr1	17220956	17223605	2649	cUMR	-0.135966667	0.000000000000129	0.00000000000218	Hypomethylated	-0.079833333	0.00000395	0.0000255	Non	-0.0213	0.001947254	0.004233191	Non	-0.060833333	0.0000247	0.00019241	Non	-0.108633333	0.0000000198	0.000000206	Hypomethylated	-0.082633333	0.0000106	0.0000321	Non	-0.063433333	0.0000508	0.000128991	Non	-0.07797619	0.00000000000101	0.0000000000077	Non	CROCC	24840
chr1	17229847	17232396	2549	cUMR	-0.020266667	0.000206994	0.000359506	Non	0.004033333	0.529165723	0.592890986	Non	-0.020266667	0.000125593	0.000380938	Non	-0.012566667	0.064630018	0.112683097	Non	0.002483333	0.903990995	0.916465082	Non	0.081733333	0.00000000876	0.0000000538	Non	-0.011766667	0.139159281	0.16820577	Non	-0.00022381	0.00000388	0.00000657	Non	CROCC	16049
chr1	17239973	17241572	1599	cUMR	-0.0336	0.0000607	0.000117814	Non	-0.008733333	0.103115122	0.156086506	Non	-0.014433333	0.214407852	0.262634088	Non	-0.005333333	0.581625385	0.64897942	Non	-0.014933333	0.267005037	0.315051039	Non	-0.001433333	0.161845886	0.187462733	Non	0.021066667	0.044056949	0.05931368	Non	-0.009104762	0.004647585	0.005327126	Non	CROCC	6873
chr1	17286903	17287785	882	cUMR	-0.0547	0.024142596	0.029251563	Non	0.1511	0.000166174	0.000675261	Hypermethylated	0.3368	0.000000046	0.000000358	Hypermethylated	-0.0419	0.216066675	0.296294015	Non	0.13705	0.0000611	0.000205677	Hypermethylated	0.00855	0.041334564	0.054158347	Non	-0.0519	0.079474442	0.101184411	Non	0.073185714	0.00000127	0.00000233	Non	CROCC	0
chr1	17305803	17307652	1849	cUMR	-0.031333333	0.134082821	0.146969652	Non	0.1213	0.000156889	0.000641641	Hypermethylated	0.186833333	0.000000242	0.00000154	Hypermethylated	0.0077	0.32319478	0.408301327	Non	0.0095	0.011794548	0.020515737	Non	0.102	0.001581126	0.00284513	Non	0.0779	0.000168132	0.000379301	Non	0.068957143	0.000000509	0.00000101	Non	MFAP2	0
chr1	17337135	17338934	1799	cUMR	-0.025733333	0.0000535	0.00010489	Non	-0.015633333	0.016255455	0.033878887	Non	-0.0089	0.309313259	0.361783765	Non	-0.004233333	0.112025357	0.175115922	Non	-0.012483333	0.267572542	0.315705206	Non	-0.021733333	0.003472239	0.005770621	Non	0.004766667	0.519355896	0.552487735	Non	-0.012004762	0.003065965	0.00357461	Non	ATP13A2	0
chr1	17379690	17381139	1449	cUMR	-0.021633333	0.004739006	0.006430212	Non	-0.009866667	0.031208665	0.058306342	Non	0.0072	0.590095989	0.633328871	Non	-0.013066667	0.065042627	0.113251245	Non	-0.003466667	0.904814714	0.917261578	Non	-0.012966667	0.110971564	0.132991483	Non	-0.015866667	0.084867782	0.107370266	Non	-0.009895238	0.040067438	0.04274404	Non	SDHB	0
chr1	17444671	17446423	1752	cUMR	-0.035466667	0.063594934	0.072507131	Non	0.0444	0.11596794	0.171735463	Non	0.139866667	0.00000508	0.0000226	Hypermethylated	0.0372	0.161672907	0.234627746	Non	0.03245	0.006826416	0.012707839	Non	0.09645	0.003066345	0.005160471	Non	0.0478	0.006620668	0.010643246	Non	0.051114286	0.0000284	0.0000423	Non	PADI2	0
chr1	17452023	17452822	799	cUMR	-0.129566667	0.0000000394	0.00000016	Hypomethylated	-0.030733333	0.528988405	0.592747435	Non	-0.093066667	0.002540505	0.005347287	Non	-0.061733333	0.020707434	0.044967806	Non	-0.090233333	0.00704573	0.01305961	Non	-0.065233333	0.050371407	0.064731771	Non	-0.113933333	0.000025	0.000068	Hypomethylated	-0.085419048	0.000000162	0.000000351	Non	PADI2	-6076
chr1	17563473	17564072	599	cUMR	-0.0897	0.0000111	0.0000253	Non	-0.051366667	0.016725033	0.034656731	Non	0.003966667	0.0000706	0.000230267	Non	-0.031966667	0.002780116	0.008921086	Non	-0.009616667	0.563678372	0.607574315	Non	-0.038366667	0.034466499	0.045941351	Non	-0.015966667	0.155304737	0.18571826	Non	-0.034366667	0.000118127	0.000161593	Non	PADI1	0
chr1	17661916	17663115	1199	cUMR	-0.205533333	0.000000366	0.00000118	Hypomethylated	-0.0567	0.267738854	0.340441305	Non	-0.017366667	0.018689959	0.030882517	Non	-0.1337	0.007236478	0.019184351	Non	-0.12045	0.006131588	0.011588941	Non	-0.15945	0.00196569	0.003468827	Non	-0.2307	0.00000049	0.000002	Hypomethylated	-0.130357143	0.000000184	0.000000393	Hypomethylated	PADI4	0
chr1	17746378	17746655	277	cUMR	0.315066667	0.0000000681	0.000000262	Hypermethylated	0.128866667	0.008696792	0.02010424	Non	0.0629	0.013576816	0.023269851	Non	0.030266667	0.49871162	0.575104207	Non	0.340316667	0.000000124	0.000000974	Hypermethylated	-0.037433333	0.288998785	0.3189655	Non	0.495466667	0.000000000156	0.0000000017	Hypermethylated	0.19292381	0.00000000186	0.0000000061	Hypermethylated	RCC2	0
chr1	17763329	17768628	5299	Canyon	-0.0552	0.0000106	0.0000243	Non	-0.035033333	0.021038177	0.042036231	Non	-0.007033333	0.400215069	0.451918033	Non	-0.044033333	0.001969843	0.006766826	Non	-0.060033333	0.0000318	0.000116435	Non	-0.041533333	0.008724853	0.013233352	Non	-0.030233333	0.006670816	0.01071581	Non	-0.037890476	0.00000088	0.00000167	Non	RCC2	0
chr1	17803660	17804209	549	cUMR	-0.219333333	0.0000000106	0.0000000501	Hypomethylated	-0.060933333	0.153283977	0.215488244	Non	-0.066166667	0.000903917	0.002153282	Non	-0.126533333	0.0000777	0.000489694	Hypomethylated	-0.134583333	0.001700627	0.003748098	Non	-0.169333333	0.0000561	0.000142489	Hypomethylated	-0.110533333	0.00000524	0.0000166	Hypomethylated	-0.126247619	0.00000000488	0.0000000145	Hypomethylated	RCC2	-37411
chr1	17828638	17829469	831	cUMR	-0.0871	0.0000699	0.000133659	Non	-0.032133333	0.179966874	0.246136904	Non	-0.046433333	0.026040867	0.041325836	Non	-0.049933333	0.052004156	0.09493191	Non	0.082566667	0.013749533	0.023514034	Non	-0.051433333	0.064531682	0.081019302	Non	0.021466667	0.0000000129	0.0000000791	Non	-0.027990476	0.00000262	0.00000455	Non	ARHGEF10L	36861
chr1	17864611	17867610	2999	cUMR	0.009666667	0.24208471	0.257231683	Non	0.0127	0.78609198	0.821727331	Non	-0.015166667	0.522405952	0.569120191	Non	0.0307	0.206543697	0.285509666	Non	0.02725	0.425031069	0.474250993	Non	0.00275	0.549550058	0.575084996	Non	0.0671	0.010767232	0.01649798	Non	0.018271429	0.124658858	0.129150007	Non	ARHGEF10L	0
chr1	18152608	18152957	349	cUMR	-0.208566667	0.00000015	0.00000053	Hypomethylated	-0.024366667	0.040896821	0.072644161	Non	0.336266667	0.00000000512	0.0000000522	Hypermethylated	-0.084766667	0.035021582	0.068574802	Non	-0.072316667	0.093007438	0.12499544	Non	-0.035566667	0.703392449	0.721517398	Non	-0.039766667	0.003721126	0.006302844	Non	-0.011709524	0.000000651	0.00000126	Non	ACTL8	0
chr1	18249328	18250577	1249	cUMR	-0.251833333	0.00000000000563	0.0000000000628	Hypomethylated	-0.160466667	0.00000495	0.0000311	Hypomethylated	-0.112333333	0.0000000118	0.000000108	Hypomethylated	-0.154466667	0.00000703	0.0000679	Hypomethylated	-0.084416667	0.002621859	0.005490702	Non	-0.102166667	0.005516903	0.008761456	Non	-0.128466667	0.000000335	0.00000143	Hypomethylated	-0.147095238	0.0000000000045	0.0000000000286	Hypomethylated	ACTL8	-95771
chr1	18310855	18311504	649	cUMR	-0.2854	0.00000000000226	0.0000000000277	Hypomethylated	-0.205166667	0.000000327	0.00000293	Hypomethylated	0.0666	0.010899665	0.019172923	Non	-0.155166667	0.0000892	0.000544846	Hypomethylated	-0.106566667	0.000023	0.000088	Hypomethylated	-0.154316667	0.0000128	0.000038	Hypomethylated	-0.222366667	0.00000002	0.000000117	Hypomethylated	-0.150566667	0.000000000029	0.000000000146	Hypomethylated	IGSF21	122736
chr1	18426702	18428101	1399	cUMR	-0.258333333	0.0000000000000424	0.00000000000082	Hypomethylated	-0.102033333	0.00020471	0.000807346	Hypomethylated	0.010166667	0.098918553	0.132850654	Non	-0.218233333	0.000000000047	0.0000000059	Hypomethylated	-0.111583333	0.000396536	0.001054251	Non	-0.165833333	0.000000273	0.00000121	Hypomethylated	-0.237433333	0.00000000000498	0.0000000000911	Hypomethylated	-0.153919048	0.0000000000000329	0.000000000000397	Hypomethylated	IGSF21	6139
chr1	18432992	18438191	5199	Canyon	0.125233333	0.000000459	0.00000144	Hypermethylated	0.146533333	0.000000147	0.00000147	Hypermethylated	0.144233333	0.000000117	0.000000812	Hypermethylated	0.037133333	0.030212248	0.060991435	Non	0.063733333	0.001319767	0.003008016	Non	0.426483333	0.0000000000000213	0.000000000000704	Hypermethylated	0.370133333	0.000000000000236	0.00000000000721	Hypermethylated	0.181333333	0.0000000000000447	0.000000000000517	Hypermethylated	IGSF21	0
chr1	18444597	18445136	539	cUMR	-0.185266667	0.0000329	0.0000674	Hypomethylated	-0.046866667	0.296445546	0.369590662	Non	-0.272433333	0.0000000000188	0.000000000361	Hypomethylated	-0.183266667	0.000559949	0.002452497	Non	-0.136516667	0.012852947	0.02213562	Non	-0.076266667	0.090908475	0.110860025	Non	-0.088666667	0.039681082	0.053910428	Non	-0.14832381	0.000000194	0.000000413	Hypomethylated	IGSF21	0
chr1	18497209	18498295	1086	cUMR	-0.1879	0.0000000039	0.0000000206	Hypomethylated	-0.051566667	0.012540184	0.027235766	Non	0.184433333	0.0000304	0.000109849	Hypermethylated	-0.227966667	0.000000283	0.00000501	Hypomethylated	0.058433333	0.241899826	0.288622172	Non	-0.255316667	0.000000764	0.00000305	Hypomethylated	-0.326566667	0.000000000151	0.00000000166	Hypomethylated	-0.109680952	0.000000000138	0.000000000591	Hypomethylated	IGSF21	0
chr1	18556765	18558114	1349	cUMR	-0.130933333	0.0000283	0.0000589	Hypomethylated	0.102966667	0.034158073	0.062765615	Non	-0.298933333	0.0000000000513	0.000000000869	Hypomethylated	-0.145433333	0.000610472	0.002631221	Non	0.029566667	0.507634632	0.554268792	Non	-0.013683333	0.029272127	0.039575837	Non	0.023966667	0.021336932	0.030687282	Non	-0.074519048	0.000000126	0.000000278	Non	IGSF21	0
chr1	18582776	18583175	399	cUMR	-0.0038	0.010540583	0.013495216	Non	0.034166667	0.41715269	0.488679066	Non	0.1097	0.000269615	0.000744749	Hypermethylated	-0.009833333	0.11406661	0.177581057	Non	-0.056633333	0.079125975	0.10848141	Non	0.011866667	0.660584056	0.681132796	Non	-0.020833333	0.06001598	0.078513766	Non	0.013538095	0.000949112	0.001163185	Non	IGSF21	0
chr1	18883881	18885430	1549	cUMR	-0.168833333	0.00000000181	0.0000000104	Hypomethylated	0.089133333	0.0000289	0.000146331	Non	0.0575	0.178936193	0.2238723	Non	-0.069066667	0.083233757	0.138005166	Non	-0.036416667	0.0000117	0.0000492	Non	-0.093166667	0.002367629	0.004093201	Non	-0.101466667	0.0000113	0.0000334	Hypomethylated	-0.04552381	0.000000225	0.000000473	Non	KLHDC7A	-71402
chr1	18907558	18908257	699	cUMR	-0.117266667	0.0000146	0.0000325	Hypomethylated	0.0623	0.103811504	0.156974512	Non	0.086066667	0.00213489	0.004583214	Non	-0.0689	0.036640568	0.071132233	Non	0.03265	0.001154278	0.002676912	Non	-0.00885	0.051563341	0.066113879	Non	-0.0627	0.003208154	0.005506744	Non	-0.012528571	0.0000313	0.0000463	Non	PAX7	49243
chr1	18923564	18924400	836	cUMR	-0.084033333	0.0000101	0.0000233	Non	-0.039166667	0.087944613	0.137167516	Non	-0.0627	0.000000274	0.00000171	Non	-0.079166667	0.0000969	0.000584289	Non	-0.043866667	0.093108434	0.125124194	Non	-0.059616667	0.003225731	0.005398546	Non	0.009433333	0.000000966	0.00000366	Non	-0.052538095	0.0000000934	0.000000212	Non	PAX7	33100
chr1	18925473	18926372	899	cUMR	0.002266667	0.167255561	0.181220472	Non	-0.132333333	0.000334388	0.001240608	Non	-0.152566667	0.0000144	0.0000564	Hypomethylated	0.037866667	0.443534524	0.523730643	Non	0.201766667	0.0000937	0.000298934	Hypermethylated	0.064266667	0.30914005	0.339222118	Non	-0.027533333	0.365406118	0.402374349	Non	-0.012790476	0.00019284	0.000256686	Non	PAX7	31128
chr1	18956236	18973985	17749	Canyon	0.1643	0.0000000155	0.0000000706	Hypermethylated	0.233466667	0.000000000352	0.00000000926	Hypermethylated	0.0548	0.008577578	0.01553103	Non	0.016066667	0.572992597	0.641396117	Non	0.337966667	0.00000000000456	0.000000000246	Hypermethylated	0.294466667	0.0000000000466	0.000000000504	Hypermethylated	0.276866667	0.0000000000456	0.000000000594	Hypermethylated	0.185038095	0.0000000000000233	0.000000000000296	Hypermethylated	PAX7	0
chr1	18980170	18980934	764	cUMR	0.1417	0.00072277	0.001131582	Non	0.224733333	0.0000629	0.000289569	Hypermethylated	-0.064133333	0.035930186	0.054828117	Non	-0.056066667	0.165295121	0.23887747	Non	0.157783333	0.000268878	0.000749933	Hypermethylated	0.265033333	0.0000214	0.00006	Hypermethylated	-0.084243631	0.00000000172	0.0000000135	Non	0.0736802	0.0000000417	0.000000102	Non	PAX7	0
chr1	19147049	19147648	599	cUMR	-0.1887	0.00000000108	0.00000000654	Hypomethylated	-0.105666667	0.000557413	0.001926008	Non	-0.021866667	0.011559944	0.020186343	Non	-0.098266667	0.001096877	0.004235984	Non	-0.142116667	0.0000258	0.0000973	Hypomethylated	-0.010866667	0.315044353	0.345119503	Non	-0.118066667	0.00000359	0.0000118	Hypomethylated	-0.099580952	0.0000000043	0.000000013	Non	TAS1R2	18445
chr1	19227995	19230194	2199	cUMR	-0.0321	0.00000418	0.0000105	Non	-0.0158	0.096753249	0.147991814	Non	-0.010766667	0.320489486	0.373171417	Non	-0.0214	0.010399684	0.025693212	Non	-0.0201	0.00155952	0.003476531	Non	-0.0256	0.00437302	0.007104498	Non	-0.0194	0.004756545	0.007864258	Non	-0.020657143	0.00000339	0.00000579	Non	ALDH4A1	0
chr1	19239529	19239978	449	cUMR	0.043966667	0.01900806	0.023401258	Non	0.0595	0.097942516	0.14950721	Non	0.0358	0.065017302	0.092061047	Non	0.0147	0.065672635	0.114150256	Non	0.27255	0.0000000113	0.000000131	Hypermethylated	-0.0192	0.099160371	0.120072691	Non	0.1241	0.00000000289	0.0000000211	Hypermethylated	0.070957143	0.0000000659	0.000000154	Non	IFFO2	0
chr1	19280952	19284734	3782	Canyon	-0.035933333	0.001078017	0.001637341	Non	-0.009966667	0.660510753	0.710994712	Non	0.010066667	0.575191307	0.619290507	Non	-0.021166667	0.104498003	0.165550778	Non	0.023483333	0.165466392	0.206687181	Non	-0.008016667	0.156122309	0.181434964	Non	-0.003966667	0.0908228	0.114268285	Non	-0.007680952	0.015158342	0.016663734	Non	IFFO2	0
chr1	19535279	19537828	2549	cUMR	-0.010066667	0.005319704	0.007160089	Non	-0.0055	0.372011249	0.444373919	Non	-0.0049	0.26834165	0.319475178	Non	0.0011	0.487347359	0.565136261	Non	-0.0074	0.210554253	0.255613924	Non	-0.0089	0.048673943	0.062737734	Non	-0.0035	0.462246694	0.497134404	Non	-0.005557143	0.023646129	0.025593982	Non	UBR4	0
chr1	19577021	19579370	2349	cUMR	-0.041933333	0.000000000547	0.0000000036	Non	-0.0208	0.005378435	0.013393008	Non	-0.004766667	0.00000104	0.00000554	Non	-0.0162	0.001816134	0.006350145	Non	-0.01985	0.000221528	0.000634389	Non	-0.04535	0.0000000183	0.000000105	Non	-0.0528	0.0000000000105	0.000000000173	Non	-0.028285714	0.00000000000496	0.0000000000311	Non	EMC1	0
chr1	19600313	19601075	762	cUMR	0.1543	0.000255653	0.000435326	Hypermethylated	0.336866667	0.00000146	0.0000107	Hypermethylated	0.493966667	0.00000000000435	0.000000000101	Hypermethylated	0.159666667	0.000388966	0.001825376	Non	0.162216667	0.00000695	0.0000311	Hypermethylated	-0.192783333	0.00000821	0.0000255	Hypomethylated	0.334866667	0.0000000772	0.000000386	Hypermethylated	0.226409524	0.000000000308	0.00000000121	Hypermethylated	AKR7L	0
chr1	19614745	19615942	1197	cUMR	0.034333333	0.0000166	0.0000364	Non	0.0129	0.138351754	0.198255365	Non	0.288666667	0.0000000362	0.000000291	Hypermethylated	0.0571	0.307964651	0.393203993	Non	-0.04975	0.369468253	0.418976629	Non	-0.12775	0.000238934	0.000520646	Hypomethylated	0.1005	0.006198402	0.010023267	Non	0.059442857	0.000000743	0.00000142	Non	AKR7A3	0
chr1	19637370	19639619	2249	cUMR	-0.013466667	0.158157469	0.171834025	Non	-0.0015	0.064773632	0.106775907	Non	-0.010133333	0.225017591	0.274014549	Non	-0.0033	0.443388809	0.523635556	Non	-0.00355	0.921815683	0.931479132	Non	-0.01605	0.143851281	0.168384328	Non	-0.0045	0.265325994	0.301215319	Non	-0.007614286	0.148533216	0.153271676	Non	AKR7A2	0
chr1	19810358	19812864	2506	cUMR	0.000266667	0.124717445	0.137272357	Non	0.013133333	0.048395676	0.083729941	Non	0.028766667	0.010042619	0.01784581	Non	0.020933333	0.06721462	0.116327055	Non	-0.003566667	0.869995327	0.886738036	Non	-0.003066667	0.54684529	0.572577788	Non	0.046733333	0.000387162	0.000804299	Non	0.015761905	0.001241584	0.001504334	Non	CAPZB	0
chr1	19922782	19924781	1999	cUMR	-0.0202	0.0000279	0.0000581	Non	-0.012466667	0.03752485	0.06771784	Non	-0.006033333	0.028335805	0.044475753	Non	-0.004866667	0.442279868	0.522505161	Non	-0.008866667	0.275893374	0.324379319	Non	-0.020116667	0.000446915	0.000912305	Non	-0.013466667	0.004882927	0.00805718	Non	-0.012209524	0.0000341	0.0000501	Non	MINOS1	0
chr1	19969692	19973241	3549	Canyon	-0.06	0.013831465	0.017389818	Non	0.057166667	0.224682386	0.295436617	Non	0.0935	0.018298561	0.030308525	Non	-0.025033333	0.035848183	0.069871486	Non	-0.007583333	0.34749894	0.397136937	Non	-0.070833333	0.008095783	0.012367174	Non	0.033766667	0.320871151	0.358082218	Non	0.006195238	0.155653446	0.160406122	Non	NBL1	0
chr1	19980173	19980822	649	cUMR	-0.134433333	0.00000153	0.00000425	Hypomethylated	-0.046666667	0.043707413	0.076837931	Non	0.031233333	0.070478425	0.098726332	Non	-0.282466667	0.000000000000538	0.00000000022	Hypomethylated	0.067483333	0.093793689	0.125869595	Non	-0.184766667	0.0000000408	0.000000218	Hypomethylated	-0.251266667	0.0000000000155	0.00000000024	Hypomethylated	-0.114009524	0.00000000000394	0.0000000000254	Hypomethylated	NBL1	0
chr1	19985925	19987674	1749	cUMR	-0.241566667	0.00000000117	0.00000000703	Hypomethylated	-0.0613	0.177421898	0.243276173	Non	0.048766667	0.012498696	0.021631254	Non	-0.0545	0.263902769	0.347705244	Non	-0.1129	0.000833049	0.002016556	Non	-0.17365	0.0000719	0.000177929	Hypomethylated	-0.0597	0.183361925	0.215852701	Non	-0.090871429	0.000000941	0.00000177	Non	NBL1	-977
chr1	19988071	19989054	983	cUMR	-0.1506	0.00000065	0.00000198	Hypomethylated	-0.065933333	0.101033233	0.153524898	Non	-0.031266667	0.470931026	0.520096053	Non	-0.081933333	0.008738541	0.022328075	Non	-0.093933333	0.015420485	0.026001646	Non	-0.062433333	0.067604536	0.084533733	Non	-0.091933333	0.004467346	0.007430467	Non	-0.083304762	0.00000835	0.0000135	Non	HTR6	2726
chr1	19990778	19992927	2149	cUMR	0.030966667	0.000760998	0.001186192	Non	0.1595	0.0000000761	0.000000837	Hypermethylated	0.1988	0.000000000511	0.0000000066	Hypermethylated	0.0723	0.000148737	0.000827025	Non	0.0768	0.0000658	0.000219246	Non	0.21605	0.00000000146	0.0000000107	Hypermethylated	0.2511	0.0000000000243	0.000000000353	Hypermethylated	0.141842857	0.0000000000014	0.0000000000103	Hypermethylated	HTR6	0
chr1	20042031	20042377	346	cUMR	-0.077606563	0.025633475	0.030922881	Non	-0.020072799	0.070717117	0.114875985	Non	-0.0581046	0.238404899	0.288118418	Non	-0.041072135	0.06503557	0.113247139	Non	0.109978468	0.238335279	0.284876919	Non	-0.07952182	0.21426099	0.242452166	Non	-0.043870746	0.480933452	0.515597636	Non	-0.034784996	0.387663131	0.392797634	Non	TMCO4	0
chr1	20125846	20127145	1299	cUMR	-0.023466667	0.000273825	0.000463203	Non	-0.009933333	0.320633695	0.393416679	Non	0.0442	0.000204842	0.000585077	Non	-0.026733333	0.0000067	0.0000654	Non	-0.000883333	0.018506526	0.030468693	Non	-0.024883333	0.000910238	0.001729202	Non	0.010666667	0.22468687	0.259418055	Non	-0.003104762	0.0000263	0.0000393	Non	TMCO4	0
chr1	20151819	20152018	199	cUMR	0.039066667	0.578076902	0.589775495	Non	0.1666	0.008644313	0.020004034	Non	0.060919287	0.005430834	0.010404025	Non	-0.0028	0.54323648	0.615144624	Non	0.0719	0.15047382	0.190267505	Non	0.08015	0.364843021	0.394737445	Non	0.2672	0.000264329	0.000569243	Hypermethylated	0.096089247	0.003835476	0.004431264	Non	RNF186	-10049
chr1	20208252	20210101	1849	cUMR	-0.013833333	0.014107696	0.017715855	Non	0.002533333	0.209716546	0.279525394	Non	-0.0075	0.250719432	0.301158253	Non	-0.010066667	0.077394019	0.130276688	Non	0.003583333	0.107190557	0.141569552	Non	-0.025416667	0.00000781	0.0000244	Non	-0.009466667	0.008345296	0.013098505	Non	-0.008580952	0.000268611	0.000351087	Non	OTUD3	0
chr1	20427363	20427648	285	cUMR	-0.116976148	0.000000218	0.00000074	Hypomethylated	-0.0507	0.081715863	0.12912178	Non	0.045166667	0.367575164	0.419822092	Non	-0.0721	0.003389474	0.010424959	Non	-0.03375	0.110313214	0.145144844	Non	-0.0395	0.400792922	0.430348342	Non	0.0883	0.00143085	0.002638739	Non	-0.025610906	0.0000199	0.0000304	Non	PLA2G5	-8970
chr1	20478335	20479967	1632	cUMR	-0.0711	0.000108641	0.000199559	Non	-0.016733333	0.562313525	0.623708585	Non	-0.0851	0.001476453	0.003324029	Non	-0.075133333	0.021956936	0.047183721	Non	-0.000683333	0.007558892	0.013861515	Non	-0.035933333	0.144407894	0.168986618	Non	0.000866667	0.881835834	0.892990807	Non	-0.043961905	0.000368113	0.000473007	Non	PLA2G2F	-1457
chr1	20512230	20514079	1849	cUMR	-0.0023	0.00000245	0.00000649	Non	-0.004366667	0.043244697	0.076196801	Non	0.3097	0.00000000000026	0.00000000000872	Hypermethylated	-0.041966667	0.005815969	0.016128103	Non	0.102283333	0.0000000343	0.000000328	Hypermethylated	-0.029216667	0.000253088	0.000548019	Non	-0.061166667	0.00000279	0.0000094	Non	0.045690476	0.0000000000323	0.000000000161	Non	UBXN10-AS1	0
chr1	20521644	20521944	300	cUMR	-0.0796	0.009088919	0.011755917	Non	-0.1103	0.000521629	0.00182196	Non	0.038254527	0.00000628	0.0000271	Non	-0.0869	0.004040797	0.012029047	Non	0.0299	0.231929489	0.278244413	Non	-0.1286	0.001043152	0.001956407	Non	0.2093	0.000129295	0.000299856	Hypermethylated	-0.016639574	0.00000488	0.00000814	Non	UBXN10	-1703
chr1	20537645	20539894	2249	cUMR	-0.2069	0.00000000371	0.0000000197	Hypomethylated	-0.051866667	0.171183238	0.236011128	Non	-0.004733333	0.073829311	0.102864172	Non	-0.051466667	0.108478825	0.170456619	Non	-0.081316667	0.0000266	0.0000999	Non	-0.198816667	0.00000372	0.0000126	Hypomethylated	-0.082266667	0.028844519	0.040352852	Non	-0.094809524	0.0000000868	0.000000198	Non	UBXN10	-17704
chr1	20688333	20688807	474	cUMR	-0.0049	0.035623153	0.042053923	Non	0.065	0.076224069	0.121979771	Non	0.0231	0.051224536	0.074762817	Non	-0.077	0.004857484	0.013985143	Non	0.1506	0.000146837	0.000441648	Hypermethylated	-0.0189	0.676683345	0.696421749	Non	0.065637089	0.00000000101	0.00000000852	Non	0.025833507	0.000000873	0.00000165	Non	LINC01141	0
chr1	20692254	20694503	2249	cUMR	0.002066667	0.000163067	0.000289346	Non	0.1116	0.00000113	0.00000865	Hypermethylated	0.1174	0.0000000756	0.000000557	Hypermethylated	0.0078	0.004624103	0.01341903	Non	0.04815	0.0000738	0.000242084	Non	0.22865	0.000000000648	0.00000000515	Hypermethylated	0.0844	0.0000000809	0.000000403	Non	0.081228571	0.0000000000745	0.000000000339	Non	LINC01141	0
chr1	20703219	20703674	455	cUMR	-0.05	0.005774321	0.007719383	Non	-0.069566667	0.000787005	0.002590163	Non	-0.000146946	0.0000000332	0.000000269	Non	-0.060166667	0.004854217	0.013977407	Non	-0.021416667	0.089337244	0.120614539	Non	-0.023666667	0.284705968	0.314688763	Non	-0.067966667	0.000521144	0.001055341	Non	-0.041992223	0.000000363	0.000000736	Non	LINC01141	0
chr1	20726332	20727431	1099	cUMR	-0.0593	0.033878064	0.040141291	Non	0.030633333	0.105949769	0.159537291	Non	-0.065633333	0.00000265	0.0000127	Non	-0.107966667	0.000572206	0.002495298	Non	0.044783333	0.253026727	0.300410803	Non	-0.003716667	0.810579539	0.822803106	Non	0.001833333	0.049974572	0.06659223	Non	-0.027509524	0.0000355	0.0000521	Non	LINC01141	0
chr1	20756897	20757456	559	cUMR	0.061166667	0.237719208	0.252871788	Non	0.068366667	0.045632599	0.079678202	Non	0.1275	0.006364284	0.011965867	Non	-0.022233333	0.455848231	0.535332879	Non	0.123916667	0.020624839	0.033491736	Non	0.104416667	0.011071317	0.016418252	Non	0.095166667	0.07966079	0.101384166	Non	0.07862381	0.003277949	0.003809966	Non	LINC01141	-1611
chr1	20767302	20767601	299	cUMR	-0.035966667	0.000513848	0.000827278	Non	0.057133333	0.057093346	0.096107465	Non	-0.021946832	0.000000128	0.00000088	Non	-0.061466667	0.047977617	0.088737786	Non	0.002033333	0.280145058	0.328800682	Non	-0.07618537	0.0000000442	0.000000234	Non	-0.09564098	0.000000142	0.000000662	Non	-0.032685124	0.00000000491	0.0000000146	Non	LINC01141	-12016
chr1	20802630	20803779	1149	cUMR	-0.043433333	0.216914099	0.231691678	Non	-0.054033333	0.133771605	0.192885014	Non	0.1984	0.000000952	0.00000512	Hypermethylated	-0.057233333	0.102684905	0.163214882	Non	0.096566667	0.000706632	0.001747579	Non	-0.108683333	0.000189567	0.000423018	Hypomethylated	-0.063433333	0.001924356	0.003442923	Non	0.00022381	0.0000151	0.0000235	Non	CAMK2N1	5105
chr1	20809810	20814067	4257	Canyon	-0.001766667	0.002694525	0.003813512	Non	0.048866667	0.00000288	0.0000194	Non	-0.014933333	0.000742529	0.001807422	Non	0.030466667	0.000121884	0.0007052	Non	0.045566667	0.00000161	0.00000882	Non	0.011066667	0.004960153	0.007954447	Non	0.091666667	0.0000000000707	0.000000000876	Non	0.028038095	0.000000000386	0.00000000148	Non	CAMK2N1	0
chr1	20819923	20821848	1925	cUMR	-0.020533333	0.005035848	0.006803872	Non	0.0103	0.295589985	0.368772036	Non	-0.093533333	0.00000352	0.0000164	Non	-0.0937	0.0000148	0.000125951	Non	0.00005	0.984330634	0.986377229	Non	-0.0332	0.033953304	0.045314949	Non	-0.1143	0.000000991	0.00000375	Hypomethylated	-0.051585714	0.000000699	0.00000135	Non	MUL1	4093
chr1	20833704	20834995	1291	cUMR	-0.029666667	0.0000000368	0.000000151	Non	0.003766667	0.295325002	0.368546275	Non	-0.011636151	0.0000000151	0.000000135	Non	0.006366667	0.077110045	0.129853101	Non	-0.031083333	0.000038	0.000135956	Non	-0.017333333	0.000342658	0.00071922	Non	0.001566667	0.228918501	0.263851866	Non	-0.010943747	0.0000000195	0.0000000512	Non	MUL1	0
chr1	20848389	20849338	949	cUMR	-0.059233333	0.000323714	0.000540525	Non	-0.004433333	0.2796222	0.352371936	Non	-0.006233333	0.058650197	0.084138138	Non	-0.062033333	0.001313572	0.00490159	Non	0.018766667	0.557576172	0.601938472	Non	-0.044483333	0.038121292	0.05032682	Non	-0.010033333	0.0000453	0.000116381	Non	-0.025090476	0.0000256	0.0000384	Non	MUL1	-13716
chr1	20878451	20881150	2699	cUMR	0.039433333	0.000730337	0.001142324	Non	0.2077	0.00000000000654	0.000000000408	Hypermethylated	0.0056	0.625755765	0.666230556	Non	0.0309	0.002880317	0.009173919	Non	0.17185	0.00000000000653	0.000000000326	Hypermethylated	0.50335	0.00000000000000022	0.0000000000000155	Hypermethylated	0.1371	0.00000000166	0.0000000131	Hypermethylated	0.138557143	0.00000000000000289	0.0000000000000496	Hypermethylated	FAM43B	0
chr1	20901629	20902199	570	cUMR	-0.040666667	0.006997574	0.009225206	Non	-0.016333333	0.642445543	0.69502605	Non	-0.004166667	0.104451431	0.139388115	Non	-0.012533333	0.709757879	0.760335424	Non	0.089916667	0.001072985	0.002506718	Non	-0.022083333	0.507955024	0.535112576	Non	0.102666667	0.000000679	0.00000268	Hypermethylated	0.010609524	0.000143426	0.000194152	Non	CDA	13245
chr1	20959408	20961229	1821	cUMR	-0.022866667	0.0000142	0.0000316	Non	-0.005066667	0.265574473	0.338188875	Non	0.0198	0.001297719	0.002965898	Non	-0.011466667	0.101045002	0.161118405	Non	-0.001616667	0.923847716	0.933180293	Non	-0.026366667	0.0000192	0.0000545	Non	0.001733333	0.247981091	0.283443256	Non	-0.005838095	0.000278606	0.000363401	Non	MIR6084	0
chr1	20966687	20966968	281	cUMR	-0.0628	0.003330392	0.004639332	Non	-0.042566667	0.215312545	0.285656907	Non	-0.0338	0.028906397	0.045276981	Non	-0.010966667	0.065188358	0.11344761	Non	0.184283333	0.00000509	0.0000237	Hypermethylated	-0.088466667	0.002077616	0.003643866	Non	0.113233333	0.000000239	0.00000105	Hypermethylated	0.002919048	0.00000104	0.00000194	Non	PINK1	0
chr1	20986627	20988726	2099	cUMR	-0.015233333	0.011890819	0.015100388	Non	0.0304	0.000864268	0.002811322	Non	-0.0104	0.000117318	0.000359138	Non	-0.0068	0.492171092	0.569332708	Non	-0.0099	0.273299738	0.321753729	Non	-0.02465	0.000239344	0.000521493	Non	-0.0098	0.007692406	0.012175729	Non	-0.006371429	0.0000554	0.000079	Non	DDOST	0
chr1	21022630	21023979	1349	cUMR	-0.048	0.000160186	0.000284652	Non	-0.0095	0.252680522	0.324617078	Non	-0.031166667	0.015645581	0.026358166	Non	-0.0345	0.023904242	0.050494777	Non	-0.022	0.286257191	0.335304807	Non	-0.02675	0.130813314	0.154473428	Non	0.0347	0.0000252	0.0000684	Non	-0.020471429	0.000328005	0.00042414	Non	KIF17	0
chr1	21043786	21045021	1235	cUMR	-0.050366667	0.0000215	0.0000459	Non	0.026466667	0.072269309	0.11687498	Non	0.057466667	0.001104743	0.002568956	Non	-0.032533333	0.008655923	0.022177888	Non	0.018216667	0.520561394	0.566572805	Non	0.146216667	0.00000138	0.00000517	Hypermethylated	0.125466667	0.0000000238	0.000000136	Hypermethylated	0.037066667	0.0000000687	0.00000016	Non	KIF17	0
chr1	21055974	21057173	1199	cUMR	-0.116533333	0.002071404	0.002988129	Non	0.1514	0.00136829	0.004151559	Non	0.071966667	0.00000029	0.00000179	Non	-0.0382	0.48173726	0.560041669	Non	-0.0607	0.0000555	0.000189396	Non	0.1003	0.065444347	0.082058269	Non	-0.0466	0.0000000672	0.000000341	Non	0.0064	0.0000000478	0.000000116	Non	SH2D5	0
chr1	21057774	21060523	2749	cUMR	-0.0229	0.0000342	0.0000698	Non	0.085966667	0.00000000533	0.000000088	Non	-0.0034	0.024496118	0.039171032	Non	-0.011033333	0.021578558	0.046544942	Non	0.039266667	0.00000115	0.00000658	Non	0.164766667	0.0000000000000766	0.00000000000202	Hypermethylated	-0.000433333	0.741983115	0.763302102	Non	0.029452381	0.0000000000321	0.00000000016	Non	SH2D5	0
chr1	21111145	21114904	3759	Canyon	-0.036033333	0.00000862	0.0000202	Non	-0.0239	0.010603609	0.023693913	Non	-0.013533333	0.05544779	0.08007796	Non	-0.0245	0.008561452	0.021981022	Non	-0.01695	0.024412964	0.038752626	Non	-0.03145	0.001247906	0.002299614	Non	-0.0253	0.001783997	0.003212075	Non	-0.024557143	0.000000639	0.00000124	Non	HP1BP3	0
chr1	21501007	21503956	2949	cUMR	-0.0207	0.002005276	0.002900026	Non	-0.004533333	0.638696325	0.691590966	Non	-0.0177	0.012480459	0.021605552	Non	-0.010533333	0.268553458	0.352387761	Non	-0.017033333	0.055100881	0.078872647	Non	0.086216667	0.00000104	0.00000401	Non	-0.008133333	0.135999853	0.16473395	Non	-0.001990476	0.000235815	0.000310308	Non	EIF4G3	0
chr1	21521343	21522042	699	cUMR	-0.0988	0.00128878	0.001930086	Non	-0.029566667	0.012232509	0.026683596	Non	0.026866667	0.041007974	0.061528557	Non	-0.059366667	0.025048746	0.052459499	Non	-0.064966667	0.046333306	0.067817112	Non	-0.092716667	0.013700356	0.019888626	Non	0.013633333	0.871506941	0.883310813	Non	-0.041109524	0.0113286	0.012579936	Non	EIF4G3	-17963
chr1	21615963	21617512	1549	cUMR	-0.0277	0.030961889	0.036901904	Non	-0.021833333	0.006425953	0.015527678	Non	0.004133333	0.764699351	0.793375165	Non	-0.018833333	0.351211286	0.4358355	Non	-0.007283333	0.887712866	0.902393915	Non	0.158466667	0.00000344	0.0000117	Hypermethylated	0.009166667	0.444529526	0.480029733	Non	0.008738095	0.007849635	0.00882649	Non	ECE1	0
chr1	21670892	21672929	2037	cUMR	-0.023933333	0.024139889	0.029249952	Non	-0.0262	0.003598156	0.00953638	Non	-0.018766667	0.13481325	0.174540896	Non	-0.0102	0.557560534	0.627942877	Non	-0.02985	0.011778392	0.020492073	Non	-0.0201	0.161785614	0.187414072	Non	0.0062	0.833335104	0.847795764	Non	-0.017342857	0.004117653	0.004743628	Non	ECE1	0
chr1	21694721	21695409	688	cUMR	-0.0614	0.021329405	0.026043575	Non	-0.0131	0.700837936	0.747161516	Non	0.0561	0.213069692	0.261167519	Non	0.0045	0.292356508	0.376950622	Non	0.00585	0.317858982	0.367357237	Non	-0.0969	0.004311578	0.007014611	Non	-0.0335	0.067453193	0.087318799	Non	-0.017328571	0.008887079	0.009950324	Non	ECE1	-22688
chr1	21697063	21697535	472	cUMR	-0.3122	0.0000000000000109	0.000000000000243	Hypomethylated	-0.1751	0.000000235	0.00000219	Hypomethylated	-0.1202	0.00000000734	0.0000000722	Hypomethylated	-0.1161	0.000000426	0.00000695	Hypomethylated	-0.03395	0.163531118	0.20449194	Non	-0.14995	0.00000000992	0.0000000601	Hypomethylated	-0.056464079	0.000000000000002	0.000000000000146	Non	-0.144809985	0.00000000000000167	0.000000000000031	Hypomethylated	ECE1	-25030
chr1	21754478	21755288	810	cUMR	0.323966667	0.000000000758	0.0000000048	Hypermethylated	0.329733333	0.00000000538	0.0000000885	Hypermethylated	0.123966667	0.000637421	0.001582912	Non	0.018733333	0.705537326	0.756621356	Non	0.192133333	0.00000246	0.0000126	Hypermethylated	0.458383333	0.000000000043	0.00000000047	Hypermethylated	0.107558464	0.0000029	0.00000976	Hypermethylated	0.216281732	0.0000000000145	0.00000000008	Hypermethylated	NBPF3	11295
chr1	21762978	21764027	1049	cUMR	-0.064466667	0.000000253	0.000000847	Non	0.008533333	0.001436914	0.004331955	Non	0.1532	0.00000000355	0.0000000378	Hypermethylated	-0.095866667	0.000128929	0.000738689	Non	0.258033333	0.00000000016	0.00000000416	Hypermethylated	0.125533333	0.000000012	0.0000000711	Hypermethylated	0.013133333	0.00000000568	0.0000000383	Non	0.048447619	0.0000000000019	0.0000000000134	Non	NBPF3	2556
chr1	21766008	21767607	1599	cUMR	-0.0209	0.008382527	0.01090901	Non	0.008433333	0.517132578	0.582007301	Non	0.0081	0.337699631	0.390474819	Non	0.023633333	0.098712338	0.157946756	Non	0.017683333	0.046905537	0.068513131	Non	0.122183333	0.0000000286	0.000000157	Hypermethylated	0.047033333	0.001319039	0.002451111	Non	0.025090476	0.0000085	0.0000137	Non	NBPF3	0
chr1	21835217	21836766	1549	cUMR	0.128466667	0.00000602	0.0000146	Hypermethylated	0.157633333	0.0000771	0.000346371	Hypermethylated	0.0678	0.027735428	0.043651711	Non	-0.001566667	0.971959764	0.977680277	Non	0.156133333	0.000124153	0.000381711	Hypermethylated	0.299133333	0.0000000296	0.000000162	Hypermethylated	0.110033333	0.000884161	0.001701929	Non	0.123690476	0.0000000591	0.00000014	Hypermethylated	ALPL	0
chr1	21916745	21917544	799	cUMR	-0.010633333	0.185856188	0.200159207	Non	-0.0172	0.500941275	0.567468123	Non	0.149533333	0.000308121	0.000838047	Hypermethylated	-0.1154	0.008968192	0.022784975	Non	-0.01705	0.015028491	0.025421633	Non	-0.04255	0.44101068	0.469598134	Non	0.0092	0.098508868	0.122968952	Non	-0.000628571	0.001323112	0.001597893	Non	RAP1GAP	5164
chr1	21922165	21922214	49	cUMR	-0.016826324	0.474439538	0.488216072	Non	0.024424095	0.262699114	0.335076251	Non	0.068325295	0.176941987	0.221676528	Non	0.053023672	0.105087568	0.166244352	Non	0.06907448	0.211331954	0.256480531	Non	-0.043635012	0.1778225	0.204298766	Non	0.187822963	0.000424965	0.000875809	Hypermethylated	0.049637171	0.031598729	0.033919826	Non	RAP1GAP	494
chr1	21934297	21934696	399	cUMR	-0.102	0.000156932	0.000279384	Hypomethylated	0.1512	0.048686722	0.084148898	Non	0.463833333	0.0000000404	0.000000319	Hypermethylated	0.059	0.466313157	0.545548813	Non	0.1995	0.010363541	0.018306745	Non	-0.0795	0.019398918	0.02722786	Non	-0.0592	0.332344176	0.36944848	Non	0.097314286	0.000733447	0.000909843	Non	RAP1GAP	0
chr1	21948066	21949615	1549	cUMR	-0.1058	0.00000324	0.00000834	Hypomethylated	-0.062966667	0.016864662	0.034913037	Non	0.1552	0.0000191	0.0000726	Hypermethylated	-0.094766667	0.000156698	0.000862517	Non	-0.077716667	0.005495562	0.010516324	Non	-0.088466667	0.0000626	0.000157193	Non	-0.103366667	0.0000241	0.0000657	Hypomethylated	-0.04782381	0.00000196	0.00000348	Non	RAP1GAP	0
chr1	21977776	21978946	1170	cUMR	-0.027566667	0.17806411	0.192239995	Non	0.040133333	0.136784749	0.196418694	Non	0.003766667	0.292718059	0.34463161	Non	-0.028866667	0.176495026	0.251488714	Non	0.014683333	0.780903065	0.806920889	Non	0.038683333	0.282076772	0.312054632	Non	0.072333333	0.011737125	0.01784114	Non	0.013961905	0.080739546	0.084509472	Non	RAP1GAP	0
chr1	21985432	21986552	1120	cUMR	-0.0578	0.0000000677	0.00000026	Non	-0.0179	0.222924301	0.293573444	Non	0.005866667	0.002015341	0.004361142	Non	-0.0293	0.022443878	0.048044696	Non	-0.0423	0.000989899	0.002338225	Non	-0.01305	0.412788454	0.442046667	Non	-0.0183	0.234700715	0.269790499	Non	-0.024585714	0.0000088	0.0000141	Non	RAP1GAP	0
chr1	21994868	21996769	1901	cUMR	0.009433333	0.0000839	0.000157739	Non	0.054866667	0.000000928	0.00000722	Non	0.1271	0.000000000000217	0.00000000000745	Hypermethylated	0.019866667	0.000565806	0.002473208	Non	0.065266667	0.00000000776	0.0000000962	Non	0.084516667	0.0000000000994	0.000000000979	Non	0.072866667	0.0000000453	0.00000024	Non	0.061609524	0.000000000000141	0.0000000000014	Non	RAP1GAP	0
chr1	22107909	22110658	2749	cUMR	-0.0426	0.0000963	0.000178794	Non	-0.0424	0.000351226	0.001294507	Non	0.0109	0.646663739	0.685854618	Non	-0.0288	0.004379958	0.012836588	Non	-0.03785	0.006892761	0.012813527	Non	-0.0491	0.00010561	0.000251159	Non	-0.044	0.00021874	0.000479857	Non	-0.031828571	0.00000175	0.00000315	Non	USP48	0
chr1	22140769	22141288	519	cUMR	0.475	0.00000000000199	0.0000000000247	Hypermethylated	0.4477	0.00000000000132	0.000000000121	Hypermethylated	0.236	0.00000032	0.00000195	Hypermethylated	0.1407	0.00000602	0.0000599	Hypermethylated	0.55725	0.00000000000108	0.0000000000822	Hypermethylated	0.6505	0.00000000000000133	0.0000000000000679	Hypermethylated	0.4059	0.0000000000254	0.000000000367	Hypermethylated	0.401957143	0.000000000000000111	0.00000000000000546	Hypermethylated	LDLRAD2	0
chr1	22262093	22264486	2393	cUMR	-0.0302	0.189424239	0.203665074	Non	-0.0029	0.933106516	0.946221761	Non	0.046966667	0.051723278	0.075372048	Non	-0.0461	0.014769168	0.034077691	Non	-0.0302	0.308459243	0.35765049	Non	-0.0402	0.000944506	0.001788521	Non	-0.0071	0.576612088	0.607575865	Non	-0.013185714	0.274227139	0.279587308	Non	HSPG2	0
chr1	22290512	22290668	156	cUMR	-0.012772545	0.00000000996	0.0000000474	Non	-0.00366357	0.88743488	0.908317475	Non	0.006987294	0.695153289	0.730148802	Non	-0.006606956	0.468687126	0.547661463	Non	-0.003298637	0.686502118	0.721911679	Non	-0.012783041	0.00000227	0.0000081	Non	0.010570165	0.483111667	0.517656471	Non	-0.002785281	0.215362257	0.220589097	Non	CELA3B	12750
chr1	22349359	22349658	299	cUMR	-0.0511	0.109192739	0.121147634	Non	-0.037833333	0.114280367	0.169757983	Non	0.1234	0.001071905	0.002501062	Non	-0.039433333	0.319315333	0.404481755	Non	-0.015433333	0.88665553	0.90149983	Non	0.057816667	0.201965348	0.229638622	Non	0.065766667	0.008040115	0.012663221	Non	0.015595238	0.011781665	0.013063754	Non	LOC101928043	829
chr1	22350259	22353008	2749	cUMR	-0.047633333	0.0000000848	0.000000319	Non	-0.008966667	0.005423374	0.01348262	Non	-0.027466667	0.001072045	0.002501062	Non	-0.032766667	0.00000148	0.0000193	Non	0.053533333	0.00000374	0.0000181	Non	0.051783333	0.0000441	0.000114771	Non	0.013233333	0.003545993	0.006029514	Non	-0.004909524	0.0000000122	0.0000000334	Non	LOC101928043	0
chr1	22378618	22381117	2499	cUMR	-0.076933333	0.00000127	0.00000361	Non	-0.056666667	0.001216039	0.00375101	Non	-0.007266667	0.399770136	0.451479456	Non	-0.048066667	0.005739273	0.01597175	Non	-0.060516667	0.0000164	0.0000656	Non	-0.047766667	0.016900104	0.024024228	Non	-0.048666667	0.000689378	0.001360441	Non	-0.04872381	0.00000018	0.000000385	Non	CDC42	0
chr1	22458357	22459356	999	cUMR	-0.1588	0.00000451	0.0000112	Hypomethylated	0.0039	0.521438379	0.585867516	Non	-0.171466667	0.000000395	0.00000235	Hypomethylated	-0.0591	0.134644978	0.202475799	Non	-0.05805	0.264787305	0.312771162	Non	-0.1228	0.003013249	0.005081405	Non	-0.0589	0.005703832	0.00929965	Non	-0.093585714	0.00000153	0.00000278	Non	WNT4	0
chr1	22468077	22470576	2499	cUMR	-0.0054	0.619251892	0.630209557	Non	0.013766667	0.200463557	0.269307595	Non	-0.0024	0.921551581	0.931876548	Non	-0.000233333	0.046386548	0.086303194	Non	0.036266667	0.001720282	0.003785531	Non	0.054766667	0.0000357	0.0000951	Non	0.037766667	0.0000433	0.000111694	Non	0.016395238	0.0000903	0.000125288	Non	WNT4	0
chr1	22601559	22602708	1149	cUMR	-0.2532	0.000000000000000222	0.00000000000000775	Hypomethylated	-0.103066667	0.000000084	0.000000907	Hypomethylated	-0.033866667	0.000190928	0.000550292	Non	-0.148866667	0.0000000987	0.00000212	Hypomethylated	-0.140116667	0.00000202	0.0000107	Hypomethylated	-0.163616667	0.0000000282	0.000000155	Hypomethylated	-0.143866667	0.0000000000319	0.000000000441	Hypomethylated	-0.140466667	0.00000000000000289	0.0000000000000496	Hypomethylated	WNT4	-132041
chr1	22644856	22645455	599	cUMR	0.052866667	0.118066813	0.130363072	Non	0.3484	0.00000137	0.0000102	Hypermethylated	-0.144466667	0.000749107	0.001821959	Non	0.1852	0.000299712	0.001470371	Non	0.21395	0.000348386	0.000941802	Hypermethylated	0.2777	0.000153075	0.000349528	Hypermethylated	0.1146	0.029611077	0.041324482	Non	0.133085714	0.00000201	0.00000358	Hypermethylated	ZBTB40	132889
chr1	22668372	22669272	900	cUMR	-0.1038	0.0000461	0.0000916	Hypomethylated	0.1504	0.003516312	0.0093431	Non	-0.0268	0.670579042	0.707668656	Non	0.166	0.000720152	0.002994689	Non	0.0872	0.0000642	0.000214679	Non	0.04495	0.285101362	0.315024651	Non	0.0436	0.02566436	0.036276672	Non	0.044142857	0.000019	0.000029	Non	ZBTB40	109072
chr1	22743047	22743496	449	cUMR	-0.020633333	0.255504858	0.270679917	Non	0.012333333	0.10702861	0.160854391	Non	-0.016133333	0.563181499	0.607826396	Non	-0.029066667	0.025391945	0.053028434	Non	-0.005716667	0.311269016	0.360553775	Non	-0.049716667	0.075183251	0.093164292	Non	0.082333333	0.009293869	0.014444341	Non	-0.003266667	0.722812483	0.725310648	Non	ZBTB40	34848
chr1	22746647	22747446	799	cUMR	-0.207966667	0.0000000034	0.0000000183	Hypomethylated	-0.0821	0.037181135	0.06723995	Non	-0.074966667	0.004106333	0.008134354	Non	-0.1127	0.002609266	0.008489011	Non	-0.0728	0.0000513	0.000176957	Non	-0.09155	0.034683108	0.046196857	Non	-0.0257	0.000981511	0.001871823	Non	-0.098785714	0.0000000653	0.000000153	Non	ZBTB40	30898
chr1	22754675	22755524	849	cUMR	-0.3136	0.00000000707	0.0000000349	Hypomethylated	-0.1093	0.077466443	0.123614918	Non	-0.052433333	0.003544627	0.007158168	Non	-0.1201	0.006200403	0.016958258	Non	-0.1656	0.000171909	0.000507368	Hypomethylated	-0.1406	0.049347211	0.063515866	Non	-0.1633	0.001557038	0.002844888	Non	-0.153842857	0.000000126	0.000000279	Hypomethylated	ZBTB40	22820
chr1	22777876	22779925	2049	cUMR	-0.0514	0.00000000108	0.00000000652	Non	0.005433333	0.462449379	0.5317892	Non	0.0126	0.268037175	0.319163997	Non	-0.021166667	0.003194426	0.009943073	Non	-0.030066667	0.000130155	0.000397778	Non	-0.035066667	0.0000424	0.000110686	Non	-0.017566667	0.161667377	0.192571908	Non	-0.018852381	0.0000000843	0.000000193	Non	ZBTB40	0
chr1	22874701	22874950	249	cUMR	-0.0877	0.03888632	0.045693511	Non	0.287733333	0.0000162	0.000088	Hypermethylated	-0.153533333	0.0000162	0.0000626	Hypomethylated	0.135533333	0.009094591	0.023040523	Non	0.036383333	0.00183906	0.004017921	Non	0.053383333	0.050936099	0.065370299	Non	0.179333333	0.001609831	0.002928469	Non	0.054990476	0.0000115	0.0000181	Non	EPHA8	15054
chr1	22882637	22884536	1899	cUMR	-0.108033333	0.000249879	0.000426638	Hypomethylated	0.0283	0.784484408	0.820260383	Non	0.029966667	0.00000732	0.0000311	Non	-0.0963	0.000491408	0.002210556	Non	-0.07695	0.000936158	0.002230518	Non	-0.06795	0.259244807	0.288339677	Non	-0.2287	0.0000000016	0.0000000127	Hypomethylated	-0.072328571	0.0000000789	0.000000181	Non	EPHA8	5468
chr1	22889237	22891836	2599	cUMR	-0.062666667	0.0000167	0.0000366	Non	0.060566667	0.014985796	0.031601708	Non	0.015	0.00000237	0.0000115	Non	0.003766667	0.978560931	0.982925318	Non	-0.001083333	0.065068486	0.091371155	Non	0.312666667	0.00000000000161	0.0000000000278	Hypermethylated	0.072966667	0.0000097	0.000029	Non	0.047052381	0.000000000584	0.00000000216	Non	EPHA8	0
chr1	22918925	22920274	1349	cUMR	-0.318533333	0.000000000112	0.00000000089	Hypomethylated	0.0895	0.034043548	0.062588492	Non	-0.0512	0.000000247	0.00000156	Non	-0.1919	0.000218304	0.001136218	Non	-0.2222	0.000000351	0.00000237	Hypomethylated	-0.2147	0.000119864	0.000281036	Hypomethylated	-0.2837	0.0000000283	0.000000158	Hypomethylated	-0.168471429	0.0000000000462	0.000000000221	Hypomethylated	EPHA8	0
chr1	22932186	22936735	4549	Canyon	-0.248566667	0.00000000000159	0.0000000000202	Hypomethylated	-0.189166667	0.000000000819	0.000000018	Hypomethylated	-0.048733333	0.00000000000404	0.0000000000947	Non	-0.171566667	0.0000000604	0.00000141	Hypomethylated	-0.225316667	0.00000000414	0.000000057	Hypomethylated	-0.145566667	0.000043	0.00011214	Hypomethylated	-0.192366667	0.00000000207	0.0000000159	Hypomethylated	-0.172366667	0.00000000000000577	0.0000000000000891	Hypomethylated	EPHA8	-2100
chr1	22937786	22938335	549	cUMR	-0.1118	0.0000000137	0.0000000628	Hypomethylated	-0.135933333	0.0000019	0.0000135	Hypomethylated	0.169866667	0.0000000105	0.0000000985	Hypermethylated	-0.121933333	0.0000573	0.00038332	Hypomethylated	-0.135383333	0.00000025	0.00000178	Hypomethylated	-0.003633333	0.011595771	0.017123286	Non	-0.168933333	0.0000000145	0.0000000882	Hypomethylated	-0.066904762	0.0000000000711	0.000000000325	Non	EPHA8	-7700
chr1	22939086	22940835	1749	cUMR	-0.338033333	0.0000000000225	0.000000000214	Hypomethylated	-0.252733333	0.000000341	0.00000303	Hypomethylated	-0.1217	0.0000000121	0.000000111	Hypomethylated	-0.251733333	0.000000316	0.00000548	Hypomethylated	-0.313033333	0.00000000119	0.0000000206	Hypomethylated	-0.263283333	0.00000149	0.00000554	Hypomethylated	-0.314333333	0.00000000041	0.00000000393	Hypomethylated	-0.261647619	0.0000000000000148	0.000000000000199	Hypomethylated	EPHA8	-9000
chr1	22942858	22943757	899	cUMR	-0.3555	0.0000000000308	0.000000000281	Hypomethylated	-0.187266667	0.000208537	0.00082056	Hypomethylated	-0.103166667	0.0000000418	0.000000329	Hypomethylated	-0.200466667	0.00000562	0.0000566	Hypomethylated	-0.351916667	0.00000000021	0.00000000515	Hypomethylated	-0.371666667	0.000000000121	0.00000000116	Hypomethylated	-0.400666667	0.00000000000436	0.0000000000814	Hypomethylated	-0.273952381	0.0000000000000137	0.000000000000186	Hypomethylated	EPHA8	-12772
chr1	22944508	22945457	949	cUMR	-0.289466667	0.00000000111	0.0000000067	Hypomethylated	-0.0734	0.037698776	0.067955371	Non	-0.067966667	0.000000999	0.00000533	Non	-0.22	0.0000111	0.0000994	Hypomethylated	-0.2218	0.00000117	0.00000666	Hypomethylated	-0.08905	0.109405308	0.131303806	Non	-0.2198	0.00000367	0.0000121	Hypomethylated	-0.170114286	0.000000000323	0.00000000127	Hypomethylated	MIR6127	14294
chr1	22945908	22946757	849	cUMR	-0.357033333	0.00000000018	0.00000000135	Hypomethylated	-0.073233333	0.194883699	0.262999132	Non	-0.170033333	0.0000000125	0.000000114	Hypomethylated	-0.165833333	0.000692711	0.002904564	Non	-0.255283333	0.0000032	0.0000158	Hypomethylated	-0.256533333	0.00000387	0.0000131	Hypomethylated	-0.367433333	0.000000000239	0.00000000246	Hypomethylated	-0.235490476	0.00000000000254	0.0000000000173	Hypomethylated	MIR6127	12994
chr1	22952143	22953592	1449	cUMR	-0.081433333	0.000467696	0.000758397	Non	0.151333333	0.00000381	0.0000247	Hypermethylated	0.0804	0.000000000286	0.00000000398	Non	-0.017066667	0.561137335	0.63100503	Non	-0.057016667	0.00000162	0.00000886	Non	-0.003766667	0.109955967	0.131885863	Non	-0.123466667	0.000000158	0.000000729	Hypomethylated	-0.005580952	0.000000000499	0.00000000188	Non	MIR6127	6159
chr1	22955324	22956223	899	cUMR	-0.2157	0.00000000422	0.000000022	Hypomethylated	-0.182933333	0.00000163	0.0000118	Hypomethylated	0.105133333	0.0000165	0.0000636	Hypermethylated	-0.123933333	0.003044987	0.009574385	Non	-0.112283333	0.0000176	0.0000698	Hypomethylated	-0.156783333	0.00000726	0.0000228	Hypomethylated	-0.161733333	0.0000717	0.000176243	Hypomethylated	-0.116647619	0.00000000183	0.00000000603	Hypomethylated	MIR6127	3528
chr1	22967889	22969838	1949	cUMR	-0.1893	0.00000000115	0.00000000692	Hypomethylated	-0.0731	0.018488098	0.037681443	Non	-0.1228	0.0000000885	0.000000637	Hypomethylated	-0.1299	0.0000205	0.000164685	Hypomethylated	-0.11055	0.000291204	0.000805029	Hypomethylated	-0.18405	0.00000000828	0.0000000511	Hypomethylated	-0.1945	0.00000000162	0.0000000128	Hypomethylated	-0.143957143	0.000000000000834	0.00000000000656	Hypomethylated	C1QC	280
chr1	22999537	23001186	1649	cUMR	-0.176233333	0.00000914	0.0000213	Hypomethylated	0.051833333	0.124015915	0.181357659	Non	0.0196	0.341654943	0.394367046	Non	-0.171166667	0.0000125	0.00010933	Hypomethylated	-0.005566667	0.007830155	0.014307785	Non	-0.022316667	0.862776013	0.872259455	Non	-0.109366667	0.002247155	0.00397149	Non	-0.062709524	0.0000189	0.0000289	Non	C1QB	-11509
chr1	23036360	23039209	2849	cUMR	-0.0381	0.005738674	0.007677264	Non	0.047233333	0.030852753	0.057753343	Non	-0.016266667	0.046007079	0.067975388	Non	-0.010366667	0.052501738	0.095695248	Non	0.012233333	0.779516605	0.805730082	Non	-0.047516667	0.00178916	0.003182167	Non	0.000633333	0.954948797	0.95964827	Non	-0.007995238	0.047617294	0.050558757	Non	EPHB2	0
chr1	23256885	23257299	414	cUMR	-0.213766667	0.000000000594	0.00000000388	Hypomethylated	-0.048166667	0.003075397	0.008313775	Non	-0.042900366	0.0000000000512	0.000000000868	Non	-0.070766667	0.004007707	0.011943818	Non	0.046983333	0.000226684	0.000646618	Non	-0.122516667	0.002769924	0.004715288	Non	0.035033333	0.000000893	0.00000341	Non	-0.064620819	0.0000000000548	0.000000000257	Non	EPHB2	-15063
chr1	23279448	23280547	1099	cUMR	0.185866667	0.0000000195	0.0000000862	Hypermethylated	0.2796	0.000000615	0.00000506	Hypermethylated	0.098366667	0.0035722	0.007208415	Non	0.1076	0.014116689	0.032877547	Non	0.1437	0.00000042	0.00000276	Hypermethylated	0.5847	0.00000000000013	0.00000000000318	Hypermethylated	0.2436	0.0000000725	0.000000365	Hypermethylated	0.222085714	0.0000000000184	0.0000000000984	Hypermethylated	LACTBL1	0
chr1	23290654	23290959	305	cUMR	-0.082233333	0.00000279	0.00000729	Non	0.0243	0.056738063	0.095582852	Non	0.118266667	0.000112558	0.000346546	Hypermethylated	-0.0489	0.029934255	0.060531651	Non	-0.02865	0.08461163	0.115001188	Non	-0.02615	0.420859871	0.449810669	Non	-0.0379	0.024569131	0.034871755	Non	-0.009014286	0.001791717	0.002134823	Non	LACTBL1	0
chr1	23302551	23303450	899	cUMR	-0.304966667	0.0000000000000321	0.000000000000637	Hypomethylated	-0.133133333	0.000350163	0.001291382	Non	-0.099966667	0.0000011	0.00000582	Non	-0.137933333	0.000078	0.000491083	Hypomethylated	-0.135383333	0.0000000333	0.000000321	Hypomethylated	-0.219383333	0.000000156	0.000000729	Hypomethylated	-0.161333333	0.0000000199	0.000000117	Hypomethylated	-0.171733333	0.00000000000017	0.00000000000164	Hypomethylated	LACTBL1	-10629
chr1	23345000	23347849	2849	cUMR	-0.0322	0.0000000175	0.0000000782	Non	-0.019433333	0.000468782	0.001660967	Non	-0.0167	0.00000427	0.0000194	Non	-0.014033333	0.019110906	0.042181224	Non	-0.026033333	0.000347867	0.000940524	Non	-0.023533333	0.000942916	0.001786071	Non	-0.021033333	0.00016024	0.000363196	Non	-0.021833333	0.00000000297	0.00000000928	Non	KDM1A	0
chr1	23493302	23496648	3346	cUMR	0.020133333	0.287608289	0.302799797	Non	0.0203	0.352450643	0.424602763	Non	0.001966667	0.646364401	0.685597488	Non	0.0375	0.017783385	0.039746245	Non	0.0578	0.000128918	0.000394346	Non	0.0238	0.305961265	0.336101316	Non	0.0985	0.0000039	0.0000127	Non	0.035442857	0.000189576	0.000252509	Non	LUZP1	0
chr1	23503621	23504670	1049	cUMR	-0.0129	0.025286464	0.030530248	Non	0.0089	0.382105181	0.454314331	Non	0.0146	0.236981127	0.286598976	Non	-0.0147	0.142962584	0.21230741	Non	-0.00065	0.997528166	0.997776595	Non	0.03835	0.000263151	0.00056763	Non	0.0263	0.024595771	0.03490751	Non	0.007528571	0.008957489	0.010025898	Non	LUZP1	-8271
chr1	23510829	23511628	799	cUMR	-0.149066667	0.000000000645	0.00000000416	Hypomethylated	-0.065533333	0.007353756	0.01740531	Non	-0.025066667	0.00185217	0.004053914	Non	-0.048533333	0.047763479	0.088436752	Non	-0.097983333	0.00000216	0.0000113	Non	-0.094483333	0.000378058	0.000785653	Non	0.054866667	0.000000523	0.00000212	Non	-0.060304762	0.00000000431	0.000000013	Non	HTR1D	6760
chr1	23542689	23544995	2306	cUMR	-0.015	0.163546714	0.177393379	Non	0.016566667	0.278291774	0.3510258	Non	0.021666667	0.000163911	0.00048264	Non	-0.010833333	0.478207343	0.556734168	Non	0.012916667	0.519237367	0.565463759	Non	0.026666667	0.042523926	0.055538538	Non	0.003366667	0.166220336	0.197497298	Non	0.006966667	0.009649697	0.01077215	Non	HTR1D	-21468
chr1	23666659	23671958	5299	Canyon	-0.012666667	0.023818321	0.028887911	Non	-0.0021	0.570843534	0.631339115	Non	-0.005333333	0.617508307	0.658642729	Non	0.0221	0.033694959	0.066522059	Non	-0.011	0.11658143	0.152352456	Non	-0.01275	0.201982926	0.229647773	Non	-0.0257	0.000169732	0.000382588	Non	-0.006614286	0.013246707	0.014632341	Non	HNRNPR	0
chr1	23692814	23698143	5329	Canyon	-0.086633333	0.00000133	0.00000374	Non	-0.039466667	0.076080994	0.121799342	Non	-0.0118	0.810804242	0.834134248	Non	-0.008666667	0.034931521	0.068437382	Non	-0.083216667	0.0000593	0.000200385	Non	-0.034716667	0.214535714	0.242740241	Non	-0.033666667	0.005577965	0.009112315	Non	-0.042038095	0.0000287	0.0000428	Non	C1orf213	0
chr1	23729507	23730961	1454	cUMR	-0.161266667	0.000000328	0.00000107	Hypomethylated	0.039933333	0.027948939	0.053197913	Non	-0.041266667	0.318475157	0.371041111	Non	-0.089266667	0.01665213	0.037672084	Non	-0.169766667	0.0000041	0.0000196	Hypomethylated	-0.095016667	0.013249967	0.01928253	Non	-0.114666667	0.000518502	0.001050431	Non	-0.088409524	0.00000238	0.00000417	Non	TCEA3	0
chr1	23748342	23751941	3599	Canyon	-0.0329	0.252788699	0.267932005	Non	-0.0088	0.607837156	0.663786732	Non	0.3821	0.000000000398	0.00000000535	Hypermethylated	-0.0098	0.767599015	0.809841998	Non	0.02935	0.480503648	0.528174835	Non	-0.00315	0.608183761	0.630827369	Non	0.0638	0.026298311	0.037095532	Non	0.069314286	0.0000165	0.0000255	Non	TCEA3	0
chr1	23809401	23811550	2149	cUMR	-0.010066667	0.21881871	0.233618162	Non	-0.002166667	0.349753704	0.421888507	Non	0.024933333	0.070915756	0.099229352	Non	-0.015366667	0.30582199	0.39098035	Non	0.006933333	0.662831666	0.699860556	Non	0.054683333	0.00120511	0.002228302	Non	-0.011566667	0.432658749	0.468428523	Non	0.005433333	0.14042024	0.145086264	Non	ASAP3	0
chr1	23854533	23858332	3799	Canyon	-0.031466667	0.018449861	0.02277105	Non	-0.0346	0.003322788	0.008888678	Non	-0.009966667	0.398436399	0.45020492	Non	-0.0142	0.058434715	0.104015346	Non	-0.02605	0.277299933	0.325858184	Non	-0.0418	0.008388405	0.012770472	Non	-0.0198	0.16180455	0.192716249	Non	-0.024657143	0.003197173	0.003721105	Non	LOC101928163	0
chr1	23866090	23866886	796	cUMR	-0.054633333	0.032030431	0.038109476	Non	-0.0303	0.144748464	0.205454658	Non	0.0777	0.006796644	0.012664156	Non	-0.0519	0.04883229	0.089984547	Non	-0.0198	0.052308472	0.075404053	Non	-0.0138	0.871571498	0.880449799	Non	-0.0337	0.038437628	0.052377599	Non	-0.016442857	0.036182131	0.038710575	Non	E2F2	-8379
chr1	23871242	23871891	649	cUMR	-0.0438	0.448299305	0.462541387	Non	-0.0566	0.13220631	0.190982282	Non	-0.170110036	0.000000000368	0.00000000497	Hypomethylated	0.0244	0.553964717	0.624710644	Non	0.01445	0.032479333	0.049729142	Non	0.0407	0.037285383	0.049319527	Non	0.013238512	0.0000000213	0.000000123	Non	-0.033077526	0.0000000921	0.000000209	Non	ID3	12530
chr1	23880671	23883020	2349	cUMR	-0.030666667	0.012867171	0.016263143	Non	0.028666667	0.219632767	0.290078968	Non	0.0225	0.130247968	0.169394762	Non	0.035866667	0.269573723	0.353328243	Non	-0.017833333	0.684893738	0.720377517	Non	0.024416667	0.456989243	0.485325759	Non	-0.033533333	0.00541196	0.008860347	Non	0.003780952	0.052896792	0.05601876	Non	ID3	1401
chr1	23884321	23889746	5425	Canyon	-0.0315	0.075995798	0.085877924	Non	0.090733333	0.010526179	0.023555844	Non	0.1005	0.002209511	0.004720283	Non	-0.022066667	0.261409271	0.345155916	Non	-0.015416667	0.067330556	0.094136211	Non	0.006583333	0.063758578	0.080136349	Non	0.021733333	0.031438197	0.043620862	Non	0.023733333	0.06090093	0.064258095	Non	ID3	0
chr1	23894345	23895498	1153	cUMR	-0.037666667	0.000804436	0.001248891	Non	0.216566667	0.00000000729	0.000000115	Hypermethylated	-0.033333333	0.016809353	0.028090971	Non	0.006766667	0.262639027	0.346437969	Non	0.044416667	0.001812141	0.0039663	Non	0.034166667	0.021012643	0.029308484	Non	0.128366667	0.0000000419	0.000000224	Hypermethylated	0.047052381	0.0000000188	0.0000000495	Non	ID3	-8061
chr1	23945647	23946696	1049	cUMR	-0.069633333	0.000135285	0.000243818	Non	-0.002533333	0.009987946	0.022539758	Non	0.105533333	0.0000136	0.0000537	Hypermethylated	-0.014733333	0.034635018	0.067983601	Non	-0.018383333	0.155370826	0.195495309	Non	-0.077883333	0.000279617	0.000599539	Non	0.025866667	0.242867239	0.27835469	Non	-0.003619048	0.000161909	0.000217643	Non	MDS2	7128
chr1	23961472	23962121	649	cUMR	-0.1088	0.0000497	0.0000982	Hypomethylated	-0.014066667	0.166357843	0.23053223	Non	0.058866667	0.024901885	0.039727615	Non	-0.022666667	0.206137104	0.28504573	Non	-0.029966667	0.01700039	0.028304227	Non	-0.078216667	0.020887096	0.029153619	Non	0.040533333	0.504235723	0.538208814	Non	-0.020380952	0.001855733	0.002207061	Non	MDS2	0
chr1	23963809	23964308	499	cUMR	-0.0426	0.00000000306	0.0000000166	Non	0.0005	0.577628252	0.637059269	Non	0.008233333	0.587033402	0.630378812	Non	0.0033	0.612559347	0.675968573	Non	-0.0061	0.654287099	0.691683941	Non	-0.0176	0.105594044	0.12710316	Non	0.0107	0.17814149	0.210364976	Non	-0.006528571	0.0000222	0.0000336	Non	MDS2	0
chr1	23999735	24000884	1149	cUMR	-0.086933333	0.002406492	0.00343532	Non	-0.0392	0.042664214	0.075322703	Non	0.118758269	0.000000000602	0.00000000765	Hypermethylated	-0.1156	0.001349917	0.005013144	Non	-0.0746	0.054066123	0.077585031	Non	-0.11285	0.006808493	0.010592064	Non	-0.1208	0.000738885	0.001447846	Non	-0.055338216	0.000000284	0.000000586	Non	RPL11	17385
chr1	24017822	24019621	1799	cUMR	-0.067333333	0.000000000265	0.00000000189	Non	-0.067133333	0.00000081	0.00000644	Non	-0.001333333	0.224717923	0.273704433	Non	-0.034533333	0.004364026	0.01279838	Non	-0.074083333	0.00000225	0.0000117	Non	-0.072333333	0.00000196	0.00000707	Non	-0.070933333	0.00000133	0.00000486	Non	-0.053161905	0.00000000017	0.000000000711	Non	RPL11	0
chr1	24069057	24072137	3080	cUMR	-0.014866667	0.000429982	0.00070168	Non	-0.011666667	0.095422998	0.146319311	Non	0.004466667	0.729300124	0.760577937	Non	-0.008066667	0.343360321	0.428342755	Non	-0.010616667	0.152434062	0.192392816	Non	-0.019116667	0.000105168	0.000250255	Non	-0.006266667	0.226537601	0.261367012	Non	-0.008895238	0.001815797	0.002162232	Non	TCEB3	0
chr1	24098738	24098996	258	cUMR	0.0147	0.379722177	0.394505067	Non	0.021833333	0.442216176	0.512360958	Non	-0.002633333	0.350613338	0.403049453	Non	0.020233333	0.091029842	0.148118645	Non	0.040783333	0.101592506	0.1349601	Non	-0.038466667	0.006027498	0.009491025	Non	0.120467185	0.000000049	0.000000258	Hypermethylated	0.025551965	0.0000679	0.0000957	Non	TCEB3-AS1	0
chr1	24104108	24105707	1599	cUMR	-0.016233333	0.028216452	0.033833908	Non	-0.003233333	0.066291132	0.108823312	Non	-0.004566667	0.745168034	0.775381462	Non	-0.013233333	0.002398328	0.007933064	Non	0.011266667	0.122681439	0.159116109	Non	-0.012483333	0.035762226	0.047502928	Non	-0.011233333	0.241656026	0.277098297	Non	-0.007661905	0.09006468	0.094045353	Non	PITHD1	0
chr1	24116646	24119245	2599	cUMR	-0.100833333	0.000000000147	0.00000000112	Hypomethylated	-0.004133333	0.943552817	0.954187072	Non	-0.046166667	0.0000737	0.000239203	Non	-0.064533333	0.00000193	0.0000237	Non	-0.077333333	0.000000521	0.00000331	Non	-0.092083333	0.000000233	0.00000105	Non	0.002066667	0.977076014	0.979392805	Non	-0.05407619	0.000000000249	0.000000001	Non	LYPLA2	0
chr1	24125752	24128451	2699	cUMR	-0.0554	0.000000129	0.000000462	Non	-0.0305	0.00112732	0.003521068	Non	0.0176	0.330636369	0.383171839	Non	-0.0369	0.002857329	0.009116365	Non	-0.03065	0.00826755	0.014995592	Non	-0.0349	0.011924514	0.017558165	Non	-0.0315	0.000934624	0.001791481	Non	-0.0281	0.00000601	0.00000988	Non	GALE	0
chr1	24150956	24152786	1830	cUMR	-0.0728	0.00000624	0.0000151	Non	-0.038733333	0.06806361	0.111270612	Non	-0.0128	0.629495357	0.669768163	Non	-0.052733333	0.006719121	0.018075169	Non	-0.077883333	0.0000895	0.000287097	Non	-0.057883333	0.007557703	0.011628498	Non	-0.059733333	0.000278008	0.000595402	Non	-0.051790476	0.000000694	0.00000134	Non	HMGCL	0
chr1	24164408	24165707	1299	cUMR	-0.063333333	0.000221317	0.00038226	Non	-0.009333333	0.604533417	0.660834773	Non	0.0165	0.001419466	0.003208243	Non	-0.044133333	0.03444085	0.067668618	Non	-0.040083333	0.038668303	0.057967574	Non	-0.047833333	0.032402915	0.043428557	Non	-0.050533333	0.002602962	0.004549943	Non	-0.032933333	0.0000609	0.0000864	Non	FUCA1	5865
chr1	24193740	24195889	2149	cUMR	-0.038466667	0.00000753	0.0000179	Non	0.0048	0.591850758	0.649771184	Non	0.0132	0.020717887	0.033813725	Non	-0.0428	0.001765164	0.006211582	Non	-0.0278	0.007695052	0.014083343	Non	-0.04155	0.004645712	0.007502001	Non	-0.0416	0.001048915	0.001989191	Non	-0.023628571	0.00000847	0.0000136	Non	FUCA1	0
chr1	24396943	24397792	849	cUMR	-0.130266667	0.000000333	0.00000108	Hypomethylated	0.049766667	0.19264910699999999	0.260421044	Non	0.0064	0.001586238	0.003537075	Non	-0.037633333	0.033544853	0.066272647	Non	-0.018933333	0.002361263	0.005002788	Non	-0.115683333	0.000245629	0.000533561	Hypomethylated	0.026566667	0.038451552	0.05239361	Non	-0.031090476	0.0000051	0.00000847	Non	MYOM3	0
chr1	24438598	24439399	801	cUMR	-0.1236	0.002399381	0.003425779	Non	-0.0615	0.443992182	0.513899784	Non	-0.064933333	0.004235254	0.008359502	Non	-0.0955	0.140812324	0.20986427	Non	-0.06185	0.000233179	0.000662245	Non	0.0059	0.992953144	0.993777925	Non	-0.0127	0.001517098	0.002780709	Non	-0.062957143	0.0000974	0.000134489	Non	MYOM3	0
chr1	24476354	24478303	1949	cUMR	-0.146733333	0.00000000355	0.0000000189	Hypomethylated	0.060766667	0.13778221	0.197615494	Non	0.076433333	0.007170977	0.013271346	Non	-0.071833333	0.019210038	0.042345728	Non	-0.112983333	0.0000143	0.0000583	Hypomethylated	-0.128983333	0.00000513	0.0000168	Hypomethylated	-0.050633333	0.099092505	0.12363346	Non	-0.048519048	0.000000929	0.00000175	Non	IFNLR1	2344
chr1	24512839	24514638	1799	cUMR	-0.057733333	0.000180206	0.00031677	Non	-0.013766667	0.515560413	0.580644012	Non	0.139266667	0.00000608	0.0000264	Hypermethylated	-0.029966667	0.083910503	0.138888337	Non	-0.045816667	0.088582396	0.119729574	Non	-0.052316667	0.011079848	0.016427892	Non	-0.056366667	0.004380872	0.007306304	Non	-0.011538095	0.0000508	0.0000729	Non	IFNLR1	0
chr1	24525258	24526300	1042	cUMR	-0.153966667	0.00000000157	0.00000000915	Hypomethylated	-0.120766667	0.0000454	0.000217822	Hypomethylated	-0.139133333	0.00000172	0.00000864	Hypomethylated	-0.130566667	0.0000209	0.000167583	Hypomethylated	-0.058466667	0.003101068	0.006349692	Non	-0.108966667	0.000389286	0.00080654	Hypomethylated	-0.016566667	0.119517606	0.146559605	Non	-0.107652381	0.00000000132	0.00000000447	Hypomethylated	LOC284632	430
chr1	24542546	24543495	949	cUMR	-0.097266667	0.027134976	0.032615026	Non	-0.089333333	0.188540455	0.255727757	Non	0.0079	0.100734336	0.135010902	Non	-0.151333333	0.00406707	0.012096796	Non	-0.082933333	0.015431323	0.026013572	Non	0.024066667	0.842371092	0.85309765	Non	-0.072733333	0.020913682	0.030138557	Non	-0.066819048	0.00229668	0.002706905	Non	LOC284632	-4367
chr1	24647916	24649465	1549	cUMR	-0.1235	0.0000000776	0.000000294	Hypomethylated	-0.077566667	0.012613976	0.027371363	Non	0.085166667	0.032279632	0.049844583	Non	-0.023566667	0.035659509	0.069648472	Non	-0.121916667	0.0000274	0.000102492	Hypomethylated	-0.054416667	0.210183717	0.23827479	Non	0.122033333	0.0000299	0.00008	Hypermethylated	-0.023738095	0.000014	0.0000218	Non	GRHL3	0
chr1	24739412	24740664	1252	cUMR	-0.057533333	0.000000393	0.00000126	Non	-0.025766667	0.089063857	0.138534195	Non	0.2323	0.000000000000165	0.00000000000593	Hypermethylated	-0.044966667	0.000325933	0.001575044	Non	-0.039866667	0.000892539	0.002140352	Non	-0.040866667	0.000134972	0.000313017	Non	-0.031966667	0.008917789	0.013918198	Non	0.006061905	0.00000000022	0.000000000895	Non	STPG1	0
chr1	24741410	24743809	2399	cUMR	-0.116166667	0.0000000207	0.0000000906	Hypomethylated	-0.0361	0.208071104	0.277654745	Non	0.068666667	0.001919018	0.004179727	Non	-0.0611	0.003839412	0.011546505	Non	-0.0775	0.002004247	0.004328972	Non	-0.10925	0.00000239	0.00000848	Hypomethylated	-0.0687	0.000817825	0.001586149	Non	-0.053185714	0.000000716	0.00000138	Non	STPG1	0
chr1	24828341	24830422	2081	cUMR	-0.041133333	0.000000406	0.00000129	Non	-0.0292	0.00000648	0.0000396	Non	0.063366667	0.001456401	0.003284944	Non	-0.0346	0.000115075	0.000672301	Non	-0.03405	0.00203767	0.004391714	Non	-0.01505	0.332407293	0.362541342	Non	-0.0292	0.006282618	0.010148553	Non	-0.015085714	0.0000675	0.0000952	Non	RCAN3AS	0
chr1	24881834	24883083	1249	cUMR	-0.023266667	0.200932426	0.215365814	Non	0.053433333	0.026741747	0.051317662	Non	0.121066667	0.00000531	0.0000235	Hypermethylated	-0.042766667	0.002879122	0.009171325	Non	0.070483333	0.007609667	0.013944015	Non	-0.039016667	0.024628437	0.033873413	Non	0.003433333	0.047393602	0.063430139	Non	0.02237619	0.000191089	0.000254427	Non	LOC100506985	0
chr1	24913421	24914223	802	cUMR	-0.200233333	0.000000000757	0.00000000479	Hypomethylated	-0.1345	0.000134913	0.000562942	Hypomethylated	-0.0029	0.000719489	0.001758265	Non	-0.1453	0.0000601	0.000397448	Hypomethylated	0.02985	0.00026462	0.000739229	Non	-0.21715	0.0000000361	0.000000195	Hypomethylated	0.0255	0.000304763	0.000647581	Non	-0.092557143	0.00000000122	0.00000000419	Non	NCMAP	0
chr1	24968162	24971111	2949	cUMR	-0.0049	0.684088089	0.693556935	Non	0.011933333	0.221917847	0.292497982	Non	0.0191	0.010137098	0.017991146	Non	0.002933333	0.835845269	0.867674649	Non	0.006433333	0.654312686	0.691683941	Non	0.000683333	0.362547823	0.392483263	Non	0.034333333	0.000321915	0.000681085	Non	0.01027619	0.010597148	0.011798109	Non	SRRM1	0
chr1	25015019	25015418	399	cUMR	-0.0195	0.160630675	0.174356021	Non	0.012333333	0.90107104	0.918995592	Non	-0.017833333	0.426258196	0.47721115	Non	-0.014266667	0.748422316	0.793948255	Non	-0.013416667	0.140638446	0.179384178	Non	-0.019416667	0.811330047	0.823530241	Non	0.032333333	0.183161722	0.215690785	Non	-0.005809524	0.212045451	0.217256432	Non	SRRM1	-15248
chr1	25071079	25073578	2499	cUMR	-0.019333333	0.039506741	0.046377311	Non	-0.013666667	0.36439073	0.436808448	Non	-0.015333333	0.186947514	0.232687954	Non	-0.010066667	0.596367474	0.661873688	Non	-0.009166667	0.523645789	0.569595387	Non	0.048083333	0.000461799	0.00093998	Non	-0.018266667	0.104362216	0.129597607	Non	-0.007495238	0.01036135	0.011539853	Non	CLIC4	0
chr1	25174331	25175730	1399	cUMR	-0.0263	0.000000259	0.000000864	Non	0.0000667	0.916981698	0.932499493	Non	0.049866667	0.00000000466	0.000000048	Non	-0.015933333	0.00079625	0.003254417	Non	-0.002883333	0.002842114	0.005891549	Non	0.002366667	0.074211361	0.092049896	Non	0.023266667	0.003135032	0.005391601	Non	0.005038095	0.000000137	0.0000003	Non	CLIC4	-3517
chr1	25254389	25259475	5086	Canyon	0.2708	0.000000716	0.00000216	Hypermethylated	0.253566667	0.0000124	0.0000698	Hypermethylated	0.08196666699999999	0.078475956	0.108522529	Non	0.088566667	0.06482463	0.112947793	Non	0.188216667	0.000643271	0.001612393	Non	0.235716667	0.0000949	0.000227901	Hypermethylated	0.237766667	0.0000198	0.0000551	Hypermethylated	0.191766667	0.00000000867	0.0000000244	Hypermethylated	RUNX3	0
chr1	25427783	25428282	499	cUMR	-0.087333333	0.0000137	0.0000306	Non	-0.0607	0.08670953	0.135589848	Non	0.091833333	0.000322788	0.000873306	Non	-0.0725	0.009377559	0.023633175	Non	-0.027	0.014124855	0.024051777	Non	-0.13275	0.00000278	0.00000971	Hypomethylated	-0.089874396	0.000000407	0.00000169	Non	-0.049353037	0.0000000772	0.000000178	Non	SYF2	120485
chr1	25558310	25559743	1433	cUMR	-0.004304865	0.000148085	0.000265005	Non	0.012566667	0.000472621	0.001671867	Non	-0.00330656	0.000369979	0.000982345	Non	-0.001633333	0.350600251	0.435413567	Non	-0.001541316	0.000325796	0.000889146	Non	-0.004290698	0.000352801	0.000738388	Non	0.014366667	0.000145408	0.000332898	Non	0.001639834	0.000000497	0.000000985	Non	SYF2	0
chr1	25565907	25567130	1223	cUMR	-0.0372	0.008048409	0.010504255	Non	-0.0257	0.010030459	0.022616635	Non	0.002466667	0.055306108	0.079911656	Non	-0.0349	0.015381986	0.035218537	Non	-0.0187	0.341883094	0.391442904	Non	-0.0097	0.693406079	0.712233055	Non	0.0613	0.000419005	0.000864709	Non	-0.0091	0.015035831	0.016538618	Non	RSRP1	1610
chr1	25572039	25574734	2695	cUMR	-0.030566667	0.00000189	0.00000515	Non	-0.007033333	0.528611646	0.592407908	Non	0.0026	0.052513252	0.076333957	Non	-0.009033333	0.319936673	0.405077179	Non	-0.027233333	0.000361188	0.000971309	Non	-0.025733333	0.001154016	0.002142486	Non	-0.017433333	0.031489093	0.043682279	Non	-0.015633333	0.0000355	0.0000521	Non	RSRP1	0
chr1	25663114	25666113	2999	cUMR	-0.003208347	0.080208284	0.090366975	Non	-0.000210958	0.395545274	0.467646423	Non	-0.001031394	0.160810043	0.204097562	Non	0.000587525	0.735203216	0.78258435	Non	-0.001159415	0.882783773	0.89795742	Non	-0.00191007	0.17823083	0.204758131	Non	0.002589105	0.615191451	0.644170572	Non	-0.000649273	0.608038363	0.611949067	Non	TMEM50A	0
chr1	25755965	25759226	3261	cUMR	-0.0074	0.00194824	0.002823302	Non	-0.0011	0.66956902	0.719160355	Non	-0.006066667	0.002942829	0.006080972	Non	0.0007	0.932107502	0.94560607	Non	0.0006	0.852541657	0.870826927	Non	-0.0044	0.192241883	0.219359103	Non	0.0011	0.81646012	0.832455594	Non	-0.002642857	0.022765404	0.024682836	Non	TMEM57	0
chr1	25869580	25871429	1849	cUMR	-0.026433333	0.0000772	0.00014636	Non	-0.015566667	0.003033576	0.008221805	Non	0.1024	0.00000000499	0.000000051	Hypermethylated	-0.016166667	0.019466315	0.042793218	Non	-0.007266667	0.078588241	0.107836205	Non	-0.008516667	0.525108212	0.551711619	Non	-0.000366667	0.00209944	0.003728761	Non	0.006633333	0.00000994	0.0000158	Non	LDLRAP1	0
chr1	25899111	25899810	699	cUMR	-0.159333333	0.0000125	0.0000282	Hypomethylated	-0.1469	0.000186179	0.000744748	Hypomethylated	0.169833333	0.00017872	0.000520333	Hypermethylated	-0.1077	0.008660698	0.02218619	Non	-0.13275	0.000429418	0.001130889	Non	-0.148	0.00054673	0.00109464	Non	-0.0235	0.00040724	0.000842523	Non	-0.070014286	0.000000911	0.00000172	Non	LDLRAP1	-3735
chr1	25934778	25935627	849	cUMR	-0.2043	0.0000000000938	0.000000000761	Hypomethylated	-0.098733333	0.003165676	0.00852137	Non	-0.088133333	0.00042333	0.001105004	Non	-0.073533333	0.003770106	0.011366447	Non	-0.114883333	0.000692105	0.001717647	Non	-0.167633333	0.00000236	0.00000839	Hypomethylated	-0.159733333	0.000000098	0.000000478	Hypomethylated	-0.129847619	0.0000000000473	0.000000000225	Hypomethylated	MAN1C1	8332
chr1	25941535	25944834	3299	cUMR	-0.026166667	0.00094779	0.001452917	Non	0.042233333	0.027389309	0.052335278	Non	0.0885	0.0000297	0.000107524	Non	-0.006766667	0.822143718	0.856106616	Non	0.008583333	0.174520699	0.216805846	Non	0.089833333	0.000156519	0.000356137	Non	0.046433333	0.014494832	0.02165597	Non	0.033633333	0.0000349	0.0000513	Non	MAN1C1	0
chr1	25945485	25945886	401	cUMR	-0.053133333	0.132245808	0.145068258	Non	0.1037	0.068688582	0.112109778	Non	-0.0483	0.185691122	0.231314792	Non	-0.0111	0.905860986	0.924936996	Non	0.00145	0.282168425	0.331014249	Non	0.0117	0.179712415	0.206293039	Non	0.0359	0.207428157	0.241276615	Non	0.002471429	0.153253099	0.157986532	Non	MAN1C1	0
chr1	26122883	26123882	999	cUMR	-0.1602	0.00000202	0.00000546	Hypomethylated	-0.131	0.005440385	0.013517938	Non	0.112966667	0.010175603	0.018046194	Non	-0.1692	0.000378522	0.001783307	Non	-0.13695	0.0000808	0.000261857	Hypomethylated	-0.15745	0.003221166	0.005392404	Non	-0.0894	0.038592724	0.05255922	Non	-0.0974	0.000000863	0.00000164	Non	SEPN1	2785
chr1	26125948	26127447	1499	cUMR	0.003366667	0.942910263	0.944831449	Non	0.014066667	0.06800645	0.111192253	Non	-0.0163	0.013854791	0.023692362	Non	-0.000133333	0.643798763	0.703754257	Non	0.008866667	0.55522981	0.599700953	Non	-0.004133333	0.406583644	0.435985283	Non	0.019466667	0.258664398	0.294526856	Non	0.003095238	0.474201299	0.478971536	Non	SEPN1	0
chr1	26137205	26137704	499	cUMR	-0.025066667	0.118227176	0.130534156	Non	0.1495	0.00000000203	0.0000000389	Hypermethylated	-0.0224	0.164045066	0.207612941	Non	-0.0143	0.487797284	0.565467527	Non	-0.0189	0.038790198	0.058114155	Non	-0.04565	0.00737075	0.011374981	Non	0.1949	0.000000000000097	0.00000000000349	Hypermethylated	0.031642857	0.000000000358	0.00000000139	Non	SEPN1	0
chr1	26144581	26147880	3299	cUMR	-0.0321	0.0000743	0.000141343	Non	0.002366667	0.579093633	0.638412448	Non	-0.007433333	0.553221065	0.598497025	Non	-0.019033333	0.066601889	0.115540445	Non	-0.015183333	0.21415744	0.25950724	Non	-0.021183333	0.059443403	0.075167504	Non	0.004966667	0.347225256	0.384130195	Non	-0.012604762	0.004328291	0.004977483	Non	SEPN1	0
chr1	26184931	26186730	1799	cUMR	-0.0548	0.0000000019	0.0000000108	Non	-0.025466667	0.00447014	0.011476343	Non	0.026366667	0.015793409	0.026585773	Non	-0.036066667	0.00022308	0.001154071	Non	-0.030216667	0.007479636	0.01373499	Non	-0.050466667	0.00000138	0.00000519	Non	-0.008066667	0.57323328	0.604385627	Non	-0.024038095	0.000000563	0.0000011	Non	AUNIP	0
chr1	26201154	26202811	1657	cUMR	-0.023933333	0.00160057	0.002354877	Non	0.032533333	0.014762607	0.031196575	Non	0.0219	0.011717378	0.020424259	Non	-0.016466667	0.097608083	0.156671808	Non	-0.011266667	0.227450184	0.27352136	Non	-0.008266667	0.650505704	0.671603036	Non	0.009933333	0.413909376	0.450029795	Non	0.001133333	0.019008669	0.020745964	Non	PAQR7	-3411
chr1	26221848	26222430	582	cUMR	-0.024166667	0.667317241	0.677466018	Non	0.280766667	0.00000144	0.0000106	Hypermethylated	-0.065166667	0.017112341	0.028547815	Non	0.075166667	0.128050619	0.194440064	Non	0.079416667	0.157770836	0.198101376	Non	0.024416667	0.793601231	0.806860284	Non	0.270566667	0.000000624	0.00000249	Hypermethylated	0.086052381	0.0000357	0.0000524	Non	STMN1	0
chr1	26230526	26233925	3399	cUMR	-0.0316	0.000755563	0.00117856	Non	-0.0227	0.010985204	0.024399527	Non	-0.017933333	0.0000319	0.000114505	Non	-0.0145	0.193494462	0.270889998	Non	-0.0191	0.233201761	0.27961469	Non	-0.0056	0.526668389	0.553037381	Non	-0.0265	0.007176974	0.011444039	Non	-0.020414286	0.0000779	0.000108972	Non	MIR3917	0
chr1	26248554	26250553	1999	cUMR	-0.0485	0.0000428	0.0000855	Non	0.0111	0.688904599	0.736786389	Non	0.2225	0.0000000000000728	0.00000000000292	Hypermethylated	-0.0255	0.125048208	0.190818878	Non	0.116	0.0000000000523	0.00000000167	Hypermethylated	0.08425	0.000000648	0.00000263	Non	-0.0207	0.173935307	0.205841814	Non	0.0477	0.00000000000499	0.0000000000312	Non	STMN1	-15187
chr1	26323743	26325292	1549	cUMR	-0.008066667	0.003413917	0.004746081	Non	0.000966667	0.392530437	0.464714844	Non	0.005433333	0.000623151	0.001551152	Non	-0.004433333	0.59778325	0.662986964	Non	-0.000233333	0.11339235	0.148629228	Non	-0.007233333	0.220600364	0.248772875	Non	0.011566667	0.089973133	0.113288008	Non	-0.000147619	0.001970155	0.002337032	Non	PAFAH2	0
chr1	26361057	26363356	2299	cUMR	-0.048366667	0.000000004	0.0000000211	Non	0.023566667	0.037446489	0.067621015	Non	0.0033	0.026934793	0.042570646	Non	-0.039233333	0.0000358	0.000262205	Non	-0.015533333	0.10134903	0.134696059	Non	-0.009783333	0.617839393	0.640181179	Non	-0.011233333	0.036659753	0.050164882	Non	-0.014461905	0.0000722	0.000101413	Non	EXTL1	0
chr1	26371864	26373563	1699	cUMR	-0.0283	0.005105265	0.006891478	Non	0.108133333	0.0000000851	0.000000918	Hypermethylated	0.012385566	0.00000394	0.0000181	Non	0.019133333	0.09086249	0.147906261	Non	0.052033333	0.00000556	0.0000256	Non	0.138283333	0.0000000296	0.000000162	Hypermethylated	0.154733333	0.0000000000538	0.000000000686	Hypermethylated	0.059308135	0.000000000115	0.000000000501	Non	SLC30A2	0
chr1	26430987	26431786	799	cUMR	-0.0428	0.548590316	0.560831895	Non	0.006866667	0.251434568	0.323309433	Non	0.0537	0.149860427	0.191674357	Non	0.002266667	0.796871063	0.834804107	Non	-0.005133333	0.9912944	0.992447547	Non	-0.128133333	0.007224019	0.0111743	Non	-0.054533333	0.275260466	0.311480989	Non	-0.019847619	0.126833125	0.131357429	Non	PDIK1L	5870
chr1	26436615	26439464	2849	cUMR	-0.014866667	0.001201769	0.001808545	Non	-0.0112	0.046998713	0.081647177	Non	-0.0097	0.073680624	0.102674821	Non	-0.0112	0.038668147	0.074361584	Non	0.0013	0.208196699	0.253045042	Non	-0.02045	0.0000593	0.000149649	Non	-0.0056	0.264140165	0.300067013	Non	-0.0104	0.0000831	0.000115863	Non	PDIK1L	0
chr1	26471566	26472165	599	cUMR	-0.080933333	0.000568148	0.000907666	Non	-0.000466667	0.038501487	0.06914984	Non	0.083566667	0.013900812	0.023757572	Non	-0.034866667	0.333658917	0.418790251	Non	0.011483333	0.054395153	0.07799217	Non	-0.038016667	0.159550552	0.185079869	Non	0.073333333	0.00027505	0.000589906	Non	0.002847619	0.000906374	0.001113517	Non	FAM110D	13346
chr1	26483101	26483399	298	cUMR	-0.063961724	0.000501574	0.000809085	Non	0.102129804	0.033652658	0.06197387	Non	0.001129172	0.476830622	0.525816333	Non	0.117729123	0.040824664	0.077763072	Non	0.008173491	0.117328401	0.153162503	Non	-0.033620665	0.478165168	0.506119569	Non	0.048728209	0.554656508	0.5865753	Non	0.024689413	0.010884196	0.012106502	Non	FAM110D	2112
chr1	26487611	26489003	1392	cUMR	-0.037466667	0.000708787	0.001112075	Non	0.012366667	0.326935021	0.399541191	Non	0.011033333	0.09707686	0.130668501	Non	-0.023233333	0.162940128	0.236068731	Non	0.042616667	0.016912342	0.028175154	Non	0.062616667	0.000169283	0.000382073	Non	0.021366667	0.041379322	0.056037551	Non	0.008995238	0.000180331	0.000241008	Non	FAM110D	0
chr1	26490371	26491220	849	cUMR	-0.047933333	0.000639331	0.001012127	Non	-0.0186	0.417208252	0.488720395	Non	0.0734	0.001199169	0.002762146	Non	-0.0318	0.00835832	0.021565181	Non	-0.02885	0.114383569	0.149813319	Non	-0.0166	0.314659242	0.344744643	Non	-0.0228	0.129389455	0.157455093	Non	-0.011285714	0.00140501	0.00169205	Non	FAM110D	-1253
chr1	26495951	26497118	1167	cUMR	-0.0265	0.000000173	0.000000604	Non	-0.0012	0.108024608	0.162114279	Non	0.007833333	0.12249378	0.16041819	Non	-0.0114	0.099588125	0.159185104	Non	-0.01675	0.009329479	0.016683232	Non	-0.02725	0.00000975	0.0000298	Non	-0.0034	0.682741213	0.708101936	Non	-0.010514286	0.0000172	0.0000265	Non	ZNF593	0
chr1	26551489	26552588	1099	cUMR	-0.005	0.0000000108	0.0000000509	Non	0.380733333	0.00000000000000766	0.00000000000271	Hypermethylated	0.208666667	0.000000000201	0.00000000293	Hypermethylated	0.144933333	0.000000179	0.00000346	Hypermethylated	0.321333333	0.0000000000000113	0.00000000000268	Hypermethylated	0.096583333	0.000295614	0.000630362	Non	0.067733333	0.002831044	0.004911572	Non	0.167447619	0.0000000000000108	0.000000000000152	Hypermethylated	LOC101928303	0
chr1	26559934	26561983	2049	cUMR	-0.019633333	0.0000185	0.0000401	Non	-0.007866667	0.193435994	0.2613285	Non	0.001533333	0.002284873	0.004862315	Non	-0.017466667	0.000174746	0.000942695	Non	-0.012216667	0.023382107	0.037354947	Non	-0.030466667	0.0000000188	0.000000107	Non	-0.002866667	0.023600641	0.033629867	Non	-0.012009524	0.000000725	0.00000139	Non	CEP85	0
chr1	26604889	26607088	2199	cUMR	-0.060366667	0.0000303	0.0000625	Non	0.0209	0.21602205	0.286440507	Non	0.0203	0.055084396	0.079619949	Non	0.0077	0.172435815	0.246887188	Non	-0.0342	0.069501621	0.096884946	Non	-0.04595	0.005515282	0.008759459	Non	0.0919	0.001582855	0.002884404	Non	0.001185714	0.006625637	0.007502331	Non	CEP85	0
chr1	26632140	26634139	1999	cUMR	-0.0339	0.006476588	0.008590061	Non	0.004133333	0.431588269	0.502414615	Non	0.0256	0.147424749	0.188940148	Non	-0.026666667	0.011209789	0.027222466	Non	-0.020066667	0.244187597	0.291034805	Non	-0.045316667	0.000797871	0.001537921	Non	-0.028866667	0.018506739	0.026972623	Non	-0.016238095	0.005676355	0.006462829	Non	UBXN11	0
chr1	26647539	26648551	1012	cUMR	0.027766667	0.669234683	0.679269539	Non	0.014733333	0.491280831	0.558151541	Non	-0.0844	0.003532611	0.007135099	Non	0.106533333	0.010900726	0.026675586	Non	0.104183333	0.047006551	0.068627401	Non	-0.009316667	0.945268273	0.949128118	Non	0.086933333	0.048758658	0.06510521	Non	0.03087619	0.023044338	0.024970658	Non	AIM1L	0
chr1	26662186	26663465	1279	cUMR	0.0356	0.0000000458	0.000000183	Non	0.003366667	0.898193727	0.916642988	Non	0.1696	0.000000000000129	0.00000000000484	Hypermethylated	-0.022833333	0.006035868	0.016607941	Non	0.041016667	0.000000416	0.00000275	Non	-0.025983333	0.003487992	0.005794404	Non	-0.015433333	0.0768564	0.098172806	Non	0.031909524	0.0000000000325	0.000000000162	Non	AIM1L	0
chr1	26685188	26687387	2199	cUMR	-0.0234	0.006800819	0.008989262	Non	0.1995	0.0000528	0.000248637	Hypermethylated	0.120266667	0.000219292	0.000621122	Hypermethylated	0.0269	0.074556766	0.126561634	Non	0.1791	0.0000132	0.0000545	Hypermethylated	-0.0369	0.148827926	0.173779211	Non	0.1761	0.00006	0.000149743	Hypermethylated	0.090357143	0.00000084	0.00000159	Non	ZNF683	738
chr1	26690114	26690813	699	cUMR	-0.114766667	0.0000418	0.0000838	Hypomethylated	-0.050066667	0.16207339	0.225802642	Non	0.211733333	0.000000000124	0.00000000191	Hypermethylated	-0.098266667	0.000612629	0.002637685	Non	-0.049016667	0.230185426	0.276479509	Non	-0.124766667	0.000130166	0.000303008	Hypomethylated	-0.080866667	0.000539178	0.001088565	Non	-0.035980952	0.0000000456	0.000000111	Non	ZNF683	0
chr1	26734671	26740270	5599	Canyon	0.0427	0.024313452	0.02944397	Non	0.137766667	0.00000217	0.0000151	Hypermethylated	0.106366667	0.000000076	0.000000559	Hypermethylated	0.006566667	0.112053908	0.175149192	Non	0.112116667	0.00000543	0.000025	Hypermethylated	0.242866667	0.000000000534	0.00000000434	Hypermethylated	0.197766667	0.00000000459	0.0000000317	Hypermethylated	0.114995238	0.0000000000783	0.000000000354	Hypermethylated	LIN28A	0
chr1	26743343	26744242	899	cUMR	-0.146133333	0.000000967	0.00000282	Hypomethylated	-0.0192	0.050043901	0.086062509	Non	-0.035966667	0.022152614	0.035873509	Non	-0.0824	0.013122893	0.031009558	Non	-0.0938	0.008109348	0.014746384	Non	-0.0783	0.015435478	0.022159189	Non	-0.0728	0.034128927	0.046981869	Non	-0.0758	0.0000122	0.0000191	Non	LIN28A	0
chr1	26757952	26759551	1599	cUMR	-0.075833333	0.0000000000238	0.000000000224	Non	-0.0472	0.0000183	0.0000978	Non	0.078666667	0.000000486	0.00000283	Non	-0.0234	0.000290274	0.00143018	Non	0.01175	0.002968529	0.006117815	Non	-0.01275	0.022633114	0.031363006	Non	-0.0162	0.0000065	0.0000201	Non	-0.012028571	0.00000000196	0.00000000638	Non	DHDDS	0
chr1	26796835	26800247	3412	cUMR	-0.035566667	0.0000347	0.0000707	Non	-0.031166667	0.0000774	0.000347482	Non	-0.006233333	0.038730918	0.058520137	Non	-0.013166667	0.132202415	0.199549877	Non	-0.026316667	0.011521648	0.020106357	Non	-0.025566667	0.025492813	0.034942598	Non	-0.023366667	0.005378773	0.008810804	Non	-0.022766667	0.0000163	0.0000251	Non	HMGN2	0
chr1	26826489	26828402	1913	cUMR	-0.013566667	0.000174298	0.000307371	Non	-0.008033333	0.002752195	0.007569009	Non	0.030933333	0.000000424	0.00000251	Non	-0.015033333	0.0000673	0.000435226	Non	-0.005983333	0.33136171	0.380862085	Non	-0.009483333	0.033169674	0.044369848	Non	0.000366667	0.092755843	0.116463175	Non	-0.002033333	0.0000151	0.0000234	Non	HMGN2	-23357
chr1	26855529	26858728	3199	cUMR	-0.105	0.0000467	0.0000926	Hypomethylated	-0.079733333	0.003315328	0.008871677	Non	0.084666667	0.018323413	0.030341345	Non	-0.080533333	0.007096079	0.018880735	Non	-0.111333333	0.000156022	0.000465842	Hypomethylated	-0.104833333	0.000389831	0.000807334	Hypomethylated	-0.102333333	0.000257826	0.000556533	Hypomethylated	-0.065704762	0.00000697	0.0000113	Non	RPS6KA1	0
chr1	26945572	26948702	3130	cUMR	-0.015066667	0.009128654	0.011802892	Non	-0.0063	0.576252708	0.635891446	Non	0.0791	0.00000216	0.0000106	Non	-0.0077	0.718415501	0.768039427	Non	-0.00765	0.670288552	0.706712177	Non	-0.0149	0.329772259	0.359895471	Non	0.0169	0.145717137	0.175332075	Non	0.008814286	0.002253109	0.002657892	Non	RPS6KA1	-44053
chr1	27003464	27004697	1233	cUMR	-0.0525	0.021423493	0.026151833	Non	-0.000533333	0.55852634	0.620335971	Non	-0.043638841	0.0000000285	0.000000235	Non	-0.038133333	0.189978755	0.266913948	Non	0.038916667	0.163512718	0.204479994	Non	-0.048583333	0.25757835	0.286742878	Non	0.034666667	0.479535864	0.514282365	Non	-0.018156754	0.0000345	0.0000507	Non	ARID1A	17825
chr1	27018729	27025906	7177	Canyon	-0.009933333	0.107151879	0.119042274	Non	-0.004766667	0.404547503	0.476810036	Non	-0.011433333	0.022648017	0.036591575	Non	-0.003366667	0.408062638	0.490497478	Non	-0.006266667	0.149759322	0.189543074	Non	-0.012016667	0.018005075	0.025446384	Non	-0.002566667	0.538861985	0.571195601	Non	-0.007280952	0.003442891	0.003993972	Non	ARID1A	0
chr1	27049597	27051196	1599	cUMR	0.162766667	0.000106751	0.000196403	Hypermethylated	0.154933333	0.0000311	0.000156051	Hypermethylated	-0.0639	0.00630074	0.011863981	Non	0.078933333	0.029785696	0.060297018	Non	0.083433333	0.073089293	0.101241925	Non	0.033683333	0.131700214	0.155446339	Non	0.125533333	0.00015116	0.000344854	Hypermethylated	0.08167619	0.0000746	0.000104554	Non	ARID1A	0
chr1	27112675	27116139	3464	cUMR	0.0023	0.879635885	0.88364948	Non	0.0133	0.058451215	0.098002987	Non	0.010466667	0.070585984	0.098825299	Non	0.0001	0.984610399	0.987972243	Non	0.01055	0.222511668	0.268305539	Non	0.00105	0.968039903	0.970577308	Non	0.0457	0.00000443	0.0000143	Non	0.011957143	0.001953127	0.002317859	Non	PIGV	0
chr1	27136593	27136892	299	cUMR	-0.074949781	0.0000897	0.000167675	Non	-0.008466667	0.234720066	0.305781988	Non	0.027366667	0.296621176	0.348664802	Non	-0.035266667	0.269564219	0.353328243	Non	-0.028966667	0.501005674	0.547837133	Non	-0.035466667	0.328879046	0.359001959	Non	-0.00384662	0.00015346	0.000349507	Non	-0.02231706	0.001566995	0.001878492	Non	PIGV	-11700
chr1	27188479	27192728	4249	Canyon	-0.1683	0.00000558	0.0000136	Hypomethylated	-0.119033333	0.002487703	0.006939822	Non	0.064866667	0.015150961	0.025630598	Non	-0.152033333	0.000102523	0.000611686	Hypomethylated	-0.204633333	0.000000712	0.00000433	Hypomethylated	-0.179633333	0.0000191	0.0000544	Hypomethylated	-0.214033333	0.000000016	0.0000000962	Hypomethylated	-0.130947619	0.00000000897	0.0000000252	Hypomethylated	SFN	0
chr1	27215523	27217322	1799	cUMR	-0.0201	0.017217414	0.021347255	Non	-0.006433333	0.431760023	0.502541722	Non	-0.005933333	0.73863406	0.769280127	Non	-0.010233333	0.142544046	0.211894789	Non	-0.013183333	0.259479024	0.307087983	Non	-0.019183333	0.023195817	0.032069582	Non	-0.013233333	0.240113148	0.275525806	Non	-0.012433333	0.01002609	0.011179895	Non	GPATCH3	0
chr1	27225818	27227517	1699	cUMR	-0.029233333	0.0000000538	0.000000212	Non	0.0138	0.002564068	0.007121524	Non	0.003933333	0.013410339	0.023023821	Non	-0.0208	0.0000459	0.000319674	Non	-0.0004	0.001793808	0.003931173	Non	0.0006	0.0000187	0.0000531	Non	0.0182	0.000000346	0.00000146	Non	-0.002714286	0.00000000989	0.0000000275	Non	GPATCH3	0
chr1	27247745	27249144	1399	cUMR	-0.021466667	0.022032676	0.026854658	Non	-0.020733333	0.047351908	0.082165978	Non	0.003366667	0.431285298	0.481742519	Non	-0.025333333	0.001154825	0.004419401	Non	-0.019383333	0.116111669	0.151820889	Non	-0.020633333	0.025238827	0.034625975	Non	-0.008533333	0.55161298	0.58358212	Non	-0.01547619	0.004835464	0.005534578	Non	NUDC	0
chr1	27285692	27287564	1872	cUMR	-0.047833333	0.00000803	0.0000189	Non	-0.033266667	0.004498752	0.011531372	Non	0.084833333	0.0000295	0.000106766	Non	-0.024466667	0.091868714	0.14924176	Non	-0.049666667	0.000055	0.00018799	Non	-0.045666667	0.000509451	0.001027858	Non	-0.038466667	0.001123203	0.002117738	Non	-0.018295238	0.0000114	0.000018	Non	KDF1	0
chr1	27319013	27321462	2449	cUMR	-0.032066667	0.000000348	0.00000113	Non	0.019566667	0.014537852	0.030799948	Non	0.0261	0.00000952	0.0000391	Non	-0.009633333000000001	0.006690451	0.018006086	Non	0.028016667	0.002932785	0.00605295	Non	-0.012483333	0.002316445	0.004013061	Non	0.016766667	0.001472521	0.002708127	Non	0.004566667	0.000000133	0.000000293	Non	TRNP1	0
chr1	27323839	27324688	849	cUMR	-0.109766667	0.0000783	0.00014824	Hypomethylated	-0.0631	0.024087833	0.046988142	Non	0.1564	0.0000273	0.0000999	Hypermethylated	-0.0871	0.006203733	0.016958258	Non	-0.09285	0.000183447	0.000537538	Non	-0.1281	0.00000802	0.000025	Hypomethylated	-0.0281	0.12345081	0.150937219	Non	-0.042728571	0.0000336	0.0000495	Non	TRNP1	0
chr1	27338646	27340286	1640	cUMR	-0.0729	0.00000318	0.0000082	Non	0.012733333	0.0000817	0.00036396	Non	0.159266667	0.000000000375	0.00000000505	Hypermethylated	-0.039466667	0.027287855	0.056219783	Non	0.028683333	0.00000729	0.0000323	Non	-0.006816667	0.025480562	0.034927792	Non	0.118533333	0.00000000203	0.0000000156	Hypermethylated	0.030419048	0.000000000192	0.000000000793	Non	FAM46B	0
chr1	27370847	27371546	699	cUMR	-0.0905	0.0000343	0.0000699	Non	-0.003533333	0.933972907	0.946940932	Non	0.0065	0.453763458	0.503953904	Non	-0.058533333	0.026235368	0.054454772	Non	-0.033833333	0.353332678	0.402830903	Non	-0.090083333	0.000913097	0.001733949	Non	-0.001133333	0.074991689	0.095997661	Non	-0.037590476	0.000913537	0.001121754	Non	FAM46B	-31515
chr1	27372218	27373040	822	cUMR	-0.124966667	0.0000205	0.0000439	Hypomethylated	-0.036466667	0.255038156	0.327018275	Non	-0.0253	0.542185241	0.587983436	Non	-0.002466667	0.806308038	0.843224021	Non	-0.057716667	0.071004312	0.098739795	Non	-0.101716667	0.003968126	0.006515222	Non	-0.042066667	0.311387717	0.348505017	Non	-0.055552381	0.000662893	0.000826234	Non	FAM46B	-32886
chr1	27429041	27429439	398	cUMR	-0.022966667	0.126445328	0.139091592	Non	-0.006766667	0.491478145	0.558349387	Non	-0.1193	0.000000241	0.00000153	Hypomethylated	-0.012166667	0.028867301	0.05879346	Non	0.006033333	0.082004786	0.111887177	Non	-0.015716667	0.696928789	0.715576905	Non	0.155833333	0.0000000505	0.000000264	Hypermethylated	-0.005938095	0.00000107	0.000002	Non	SLC9A1	0
chr1	27480650	27482392	1742	cUMR	-0.1239	0.0000000000252	0.000000000236	Hypomethylated	-0.0701	0.00036083	0.001325243	Non	0.057933333	0.012783249	0.022079325	Non	-0.0309	0.201830925	0.280200578	Non	-0.06715	0.00021357	0.000615107	Non	-0.08765	0.0000311	0.000084	Non	-0.0055	0.941973995	0.947720885	Non	-0.044214286	0.0000000927	0.00000021	Non	SLC9A1	0
chr1	27560486	27562585	2099	cUMR	-0.016633333	0.0000862	0.000161832	Non	-0.001066667	0.621749537	0.676127018	Non	0.005866667	0.045311586	0.067116516	Non	-0.011066667	0.021318129	0.046073926	Non	-0.001216667	0.026183075	0.041217927	Non	-0.004716667	0.084330193	0.103440344	Non	0.019933333	0.006444462	0.010382147	Non	-0.001409524	0.001202101	0.001458475	Non	WDTC1	0
chr1	27611001	27611443	442	cUMR	-0.071466667	0.004240667	0.005806011	Non	-0.081633333	0.003034982	0.008223768	Non	-0.068466667	0.000621545	0.001547792	Non	-0.074033333	0.006937178	0.018543774	Non	-0.026133333	0.084652356	0.115050053	Non	-0.076633333	0.007575493	0.011652893	Non	0.078566667	0.00000173	0.00000614	Non	-0.046747619	0.0000069	0.0000113	Non	WDTC1	0
chr1	27623332	27623455	123	cUMR	-0.018189768	0.01477411	0.018492961	Non	0.000381086	0.26479786	0.337306868	Non	-0.009265663	0.591520122	0.634687738	Non	-0.015523784	0.070628199	0.121224953	Non	0.008708007	0.107621117	0.142052565	Non	-0.00727139	0.054325485	0.069310438	Non	0.096442424	0.0000732	0.000179507	Non	0.007076678	0.00117721	0.001429788	Non	WDTC1	0
chr1	27648045	27649844	1799	cUMR	-0.0626	0.0000000000002	0.00000000000321	Non	-0.022666667	0.006158203	0.014978723	Non	-0.0321	0.00000171	0.00000863	Non	-0.027066667	0.001245045	0.004695142	Non	-0.048766667	0.000000412	0.00000272	Non	-0.074516667	0.000000000000764	0.0000000000147	Non	-0.039866667	0.00000066	0.00000261	Non	-0.04312381	0.0000000000000356	0.000000000000426	Non	TMEM222	0
chr1	27691631	27693880	2249	cUMR	-0.026066667	0.000299049	0.000502229	Non	-0.0076	0.007126935	0.016941674	Non	-0.000233333	0.623066706	0.663842132	Non	-0.0242	0.002254954	0.007553499	Non	-0.03065	0.0000417	0.000147575	Non	-0.02365	0.00964443	0.014507583	Non	-0.0072	0.56553844	0.596971214	Non	-0.016285714	0.000233505	0.000307554	Non	MAP3K6	0
chr1	27709389	27709988	599	cUMR	-0.127	0.00000973	0.0000225	Hypomethylated	-0.0167	0.735974787	0.778490165	Non	0.017833333	0.770794358	0.79839254	Non	-0.0821	0.000666109	0.002819217	Non	-0.0915	0.031112354	0.047922372	Non	-0.038	0.199832387	0.227460245	Non	-0.1131	0.001008246	0.001918406	Non	-0.063785714	0.001649922	0.001972703	Non	CD164L2	0
chr1	27717900	27719614	1714	cUMR	-0.034166667	0.0000000282	0.000000119	Non	0.012966667	0.253946208	0.32587825	Non	0.0475	0.0000000132	0.00000012	Non	-0.023833333	0.001033806	0.004031825	Non	0.009166667	0.470051544	0.518205383	Non	-0.023583333	0.002544201	0.004371803	Non	0.000366667	0.000637435	0.00126782	Non	-0.000861905	0.000000135	0.000000297	Non	GPR3	0
chr1	27721624	27722626	1002	cUMR	-0.062766667	0.000399244	0.000655738	Non	-0.029533333	0.200217592	0.269067164	Non	0.018233333	0.459005528	0.508907993	Non	-0.044133333	0.098935796	0.15825787	Non	-0.058933333	0.008455792	0.015297873	Non	-0.054683333	0.036792391	0.048766461	Non	-0.012133333	0.628390534	0.65707769	Non	-0.03287619	0.004029576	0.004646159	Non	GPR3	0
chr1	27814652	27817410	2758	cUMR	-0.0249	0.000518024	0.000833446	Non	0.000433333	0.643402584	0.69581151	Non	0.038433333	0.0020774	0.004478546	Non	-0.012366667	0.022922732	0.048836708	Non	-0.010816667	0.054055295	0.077574113	Non	-0.025066667	0.000805584	0.001550928	Non	-0.013166667	0.202158571	0.235620245	Non	-0.005366667	0.012886292	0.014244019	Non	WASF2	0
chr1	27853397	27856020	2623	cUMR	0.001566667	0.498040095	0.511366434	Non	0.0274	0.183318406	0.249868411	Non	-0.059933333	0.0000796	0.000256075	Non	-0.0176	0.223359915	0.304131949	Non	-0.06035	0.021095144	0.034133539	Non	0.05665	0.043021419	0.056141868	Non	0.0676	0.021174366	0.030484407	Non	0.000628571	0.138952529	0.143612886	Non	AHDC1	4736
chr1	27894577	27895626	1049	cUMR	-0.045266667	0.028847559	0.034542533	Non	-0.019466667	0.007584703	0.01787293	Non	0.3819	0.0000000000106	0.00000000022	Hypermethylated	-0.036466667	0.12116805	0.18604063	Non	-0.043516667	0.143179809	0.182239847	Non	-0.068016667	0.011322873	0.01675302	Non	0.034133333	0.376232312	0.412921603	Non	0.041847619	0.000000957	0.0000018	Non	AHDC1	0
chr1	27901399	27902841	1442	cUMR	0.254833333	0.000000223	0.000000757	Hypermethylated	0.081133333	0.001178823	0.003652109	Non	0.161833333	0.0000301	0.000108761	Hypermethylated	-0.046866667	0.015787234	0.036006129	Non	0.171583333	0.00000172	0.00000931	Hypermethylated	-0.018916667	0.722507315	0.73960245	Non	-0.018066667	0.281338244	0.317731831	Non	0.091190476	0.00000044	0.000000878	Non	AHDC1	0
chr1	27928352	27931401	3049	cUMR	-0.014066667	0.008901649	0.0115329	Non	0.006233333	0.136053511	0.195613452	Non	-0.010233333	0.226583309	0.275753981	Non	-0.012166667	0.160453168	0.23308219	Non	-0.000816667	0.829057938	0.849901655	Non	0.001933333	0.718764111	0.73608336	Non	0.032233333	0.0000234	0.0000642	Non	-0.000280952	0.003528957	0.004089876	Non	AHDC1	0
chr1	27932502	27933559	1057	cUMR	-0.056033333	0.001179855	0.00177812	Non	0.040433333	0.050672479	0.086975905	Non	-0.0497	0.006527198	0.012216886	Non	-0.055166667	0.027241262	0.05615088	Non	-0.023866667	0.316010295	0.365483591	Non	-0.018116667	0.606627786	0.629484407	Non	0.197233333	0.000000302	0.0000013	Hypermethylated	0.003147619	0.000112494	0.000154361	Non	AHDC1	-2498
chr1	27960358	27961857	1499	cUMR	-0.044166667	0.000294607	0.000495356	Non	0.270033333	0.0000000218	0.000000286	Hypermethylated	0.263833333	0.0000000115	0.000000107	Hypermethylated	0.058033333	0.064363193	0.112360491	Non	0.105833333	0.0000000545	0.000000483	Hypermethylated	0.094083333	0.002723738	0.004646409	Non	0.260233333	0.0000000261	0.000000147	Hypermethylated	0.144547619	0.000000000353	0.00000000137	Hypermethylated	FGR	0
chr1	27985517	27987166	1649	cUMR	-0.109333333	0.00000000344	0.0000000184	Hypomethylated	-0.070966667	0.000540791	0.001879439	Non	0.2215	0.00000000139	0.0000000163	Hypermethylated	-0.048766667	0.02919334	0.05931245	Non	-0.056166667	0.019453261	0.031827326	Non	-0.080916667	0.000318552	0.00067361	Non	0.050433333	0.036921631	0.050477368	Non	-0.006338095	0.0000000645	0.000000151	Non	IFI6	5406
chr1	27989358	27990186	828	cUMR	0.041733333	0.119908898	0.132251573	Non	0.000566667	0.743234976	0.784379329	Non	-0.021766667	0.346352272	0.3990055	Non	0.030566667	0.518040403	0.592460615	Non	0.094316667	0.0080378	0.014635052	Non	0.010566667	0.72973395	0.746461021	Non	0.126566667	0.000338233	0.000712417	Hypermethylated	0.037938095	0.040373735	0.043061267	Non	IFI6	2386
chr1	27995517	27996402	885	cUMR	0.004633333	0.044091413	0.051433578	Non	-0.0039	0.721349943	0.765203802	Non	-0.037533333	0.018865788	0.031143154	Non	0.1069	0.000000126	0.00000258	Hypermethylated	0.0193	0.538840973	0.583857453	Non	0.01105	0.402848621	0.432305222	Non	0.0093	0.184251187	0.216774452	Non	0.013871429	0.000148329	0.000200383	Non	IFI6	0
chr1	27998045	27998988	943	cUMR	-0.163133333	0.0000000154	0.0000000701	Hypomethylated	-0.073533333	0.001132604	0.003534362	Non	-0.071133333	0.0000105	0.0000426	Non	-0.105933333	0.000108716	0.0006418	Hypomethylated	-0.066883333	0.010979961	0.019290277	Non	-0.061883333	0.128381523	0.151847351	Non	-0.004533333	0.186436549	0.219065445	Non	-0.081161905	0.0000000368	0.0000000908	Non	IFI6	0
chr1	28051445	28053278	1833	cUMR	-0.0444	0.000451885	0.000734736	Non	-0.021166667	0.23909838	0.310395091	Non	-0.009566667	0.344083589	0.396695355	Non	-0.008566667	0.341942015	0.427104063	Non	-0.018816667	0.320363349	0.369790487	Non	-0.015316667	0.113645099	0.135857704	Non	-0.011566667	0.31365818	0.350643156	Non	-0.019052381	0.019380006	0.021131143	Non	FAM76A	0
chr1	28098704	28100953	2249	cUMR	-0.026833333	0.001019627	0.00155483	Non	0.031533333	0.077600082	0.123795378	Non	0.038666667	0.014011459	0.023921606	Non	0.013933333	0.545874278	0.617494172	Non	0.037333333	0.038618692	0.057904009	Non	-0.016916667	0.307634627	0.337754761	Non	0.012133333	0.021499332	0.03089319	Non	0.012590476	0.002611765	0.00306284	Non	STX12	0
chr1	28156712	28158358	1646	cUMR	-0.0311	0.00000103	0.00000299	Non	-0.019433333	0.007015971	0.016715794	Non	0.003566667	0.019837155	0.032548224	Non	-0.018433333	0.01096002	0.026786468	Non	-0.014933333	0.026570825	0.041745258	Non	-0.017433333	0.029872024	0.040310003	Non	-0.022633333	0.00000909	0.0000273	Non	-0.017061905	0.000000893	0.00000169	Non	PPP1R8	0
chr1	28198631	28199926	1295	cUMR	-0.0281	0.014247027	0.017875917	Non	0.129166667	0.0000000992	0.00000105	Hypermethylated	0.1464	0.00000000123	0.0000000146	Hypermethylated	-0.025433333	0.024503043	0.051534879	Non	0.190316667	0.000000000152	0.00000000398	Hypermethylated	0.145816667	0.000000000439	0.00000000364	Hypermethylated	-0.004633333	0.535514912	0.567947506	Non	0.072852381	0.0000000000234	0.000000000121	Non	THEMIS2	0
chr1	28240293	28241642	1349	cUMR	-0.0005	0.0000000000029	0.0000000000346	Non	0.011366667	0.002557286	0.007108268	Non	-0.012166667	0.000541797	0.001372317	Non	-0.006833333	0.289526707	0.374182912	Non	0.008166667	0.0000000697	0.000000597	Non	-0.008333333	0.012273244	0.018011123	Non	0.003366667	0.056926256	0.074853755	Non	-0.001061905	0.000000000453	0.00000000172	Non	RPA2	0
chr1	28261118	28262517	1399	cUMR	-0.100333333	0.004344784	0.005938101	Non	-0.0515	0.356441445	0.428742616	Non	0.140666667	0.000544753	0.001378318	Non	-0.1411	0.000011	0.0000987	Hypomethylated	0.039	0.00335975	0.006812221	Non	-0.02575	0.75016773	0.765705534	Non	-0.1239	0.000184872	0.000412318	Hypomethylated	-0.036785714	0.000052	0.0000745	Non	SMPDL3B	0
chr1	28285736	28287135	1399	cUMR	-0.0837	0.0000000332	0.000000138	Non	-0.056933333	0.000187585	0.0007495	Non	0.356633333	0.000000000000000444	0.0000000000000396	Hypermethylated	-0.059933333	0.000320265	0.001552868	Non	-0.044283333	0.019102218	0.031335483	Non	-0.072283333	0.000028	0.0000764	Non	-0.077533333	0.00000138	0.00000504	Non	0.005695238	0.00000000000222	0.0000000000154	Non	XKR8	0
chr1	28413369	28415534	2165	cUMR	-0.024866667	0.000017	0.0000372	Non	-0.0094	0.423729863	0.49513231	Non	-0.004866667	0.1219116	0.159846876	Non	-0.016	0.045739821	0.085266358	Non	-0.02545	0.00270218	0.005639801	Non	-0.0362	0.0000095	0.0000291	Non	-0.0254	0.000270528	0.000581275	Non	-0.0194	0.00000346	0.00000591	Non	EYA3	0
chr1	28558521	28559920	1399	cUMR	-0.0556	0.0000000000179	0.000000000175	Non	-0.011066667	0.335223948	0.40772721	Non	-0.0206	0.0000000000018	0.000000000046	Non	-0.019266667	0.036849953	0.071463269	Non	-0.046516667	0.00000092	0.00000541	Non	-0.070516667	0.0000000000304	0.000000000347	Non	-0.086666667	0.000000000000000333	0.0000000000000362	Non	-0.043152381	0.00000000000000278	0.000000000000048	Non	DNAJC8	0
chr1	28561716	28563765	2049	cUMR	-0.063033333	0.0000172	0.0000376	Non	-0.0305	0.068846259	0.112336023	Non	-0.004033333	0.422532653	0.473810697	Non	-0.0395	0.017710152	0.039622992	Non	-0.0527	0.003891247	0.007749051	Non	-0.0617	0.0000485	0.000124958	Non	-0.0665	0.00000129	0.00000476	Non	-0.044071429	0.00000139	0.00000254	Non	ATPIF1	0
chr1	28584950	28587358	2408	cUMR	-0.004366667	0.504528964	0.517680968	Non	0.0036	0.50681763	0.572751697	Non	-0.007533333	0.027352198	0.043136994	Non	-0.0042	0.264770624	0.348505708	Non	0.0083	0.564766539	0.608528686	Non	0.01355	0.207312368	0.235285332	Non	0.0166	0.076807635	0.098115717	Non	0.002742857	0.123182254	0.127664113	Non	SESN2	0
chr1	28654975	28656324	1349	cUMR	-0.0894	0.00000000486	0.000000025	Non	0.0111	0.30923072	0.382267104	Non	0.014766667	0.0188369	0.031103989	Non	0.0183	0.378242147	0.462379103	Non	-0.0564	0.000022	0.0000849	Non	-0.01915	0.414549121	0.443715231	Non	-0.0157	0.000717057	0.00140897	Non	-0.019471429	0.000000595	0.00000116	Non	MED18	0
chr1	28695814	28697513	1699	cUMR	-0.048033333	0.00000000545	0.0000000277	Non	-0.030166667	0.001610148	0.004783005	Non	-0.006533333	0.328466687	0.381042229	Non	-0.016766667	0.039726302	0.076020363	Non	-0.025866667	0.000254825	0.000716041	Non	-0.051366667	0.000000111	0.000000535	Non	-0.075166667	0.000000000000808	0.0000000000199	Non	-0.03562381	0.0000000000246	0.000000000127	Non	PHACTR4	0
chr1	28831553	28835652	4099	Canyon	-0.0564	0.0000041	0.0000103	Non	-0.0519	0.001069602	0.003370497	Non	0.0196	0.221124956	0.269823022	Non	-0.0351	0.012078086	0.028897708	Non	-0.0454	0.009830269	0.01746975	Non	-0.0754	0.00000434	0.0000145	Non	-0.0639	0.0000336	0.0000889	Non	-0.041671429	0.000000317	0.00000065	Non	SNHG3	0
chr1	28843462	28845961	2499	cUMR	-0.053366667	0.0000000463	0.000000185	Non	-0.047766667	0.00000019	0.00000182	Non	-0.010366667	0.301234756	0.353536023	Non	-0.023166667	0.021093079	0.045648946	Non	-0.047116667	0.0000352	0.000127156	Non	-0.045866667	0.000015	0.0000437	Non	-0.029566667	0.006748755	0.010826764	Non	-0.035909524	0.00000000505	0.000000015	Non	RCC1	0
chr1	28878779	28880578	1799	cUMR	-0.023233333	0.00000131	0.00000371	Non	-0.012266667	0.019650933	0.039679037	Non	-0.007233333	0.061726001	0.087995337	Non	-0.0000667	0.435581345	0.516211234	Non	-0.010566667	0.002773514	0.005767702	Non	-0.023316667	0.0000672	0.000167438	Non	0.001533333	0.154598734	0.184947428	Non	-0.010638095	0.0000221	0.0000334	Non	TRNAU1AP	0
chr1	28906515	28909864	3349	cUMR	-0.0325	0.000130255	0.000235422	Non	-0.031166667	0.013070197	0.028160993	Non	0.041833333	0.00140375	0.003177491	Non	-0.011366667	0.489640717	0.567105129	Non	-0.007166667	0.688823908	0.724005693	Non	-0.020166667	0.171679956	0.197864249	Non	-0.020966667	0.143387552	0.172857036	Non	-0.010595238	0.003568219	0.004133988	Non	SNHG12	0
chr1	28918470	28919569	1099	cUMR	-0.014433333	0.105633231	0.117479273	Non	0.035633333	0.152960133	0.215171064	Non	0.085066667	0.000232844	0.000655832	Non	-0.034366667	0.002592957	0.008449638	Non	0.166983333	0.0000000212	0.000000218	Hypermethylated	0.169233333	0.000000242	0.00000108	Hypermethylated	0.169833333	0.000000000438	0.00000000416	Hypermethylated	0.07497619	0.00000000136	0.0000000046	Non	RAB42	0
chr1	28968151	28970549	2398	cUMR	-0.035566667	0.008616424	0.011191041	Non	-0.017133333	0.016386666	0.034102211	Non	-0.010733333	0.427471245	0.478324761	Non	-0.020933333	0.000654631	0.002780769	Non	-0.016233333	0.081304236	0.111050812	Non	-0.028983333	0.013154586	0.019164453	Non	-0.030333333	0.040629285	0.055096137	Non	-0.02287619	0.000894945	0.001100326	Non	TAF12	0
chr1	28974302	28976001	1699	cUMR	-0.091	0.0000000462	0.000000185	Non	-0.067766667	0.000191491	0.000763967	Non	0.044166667	0.003442637	0.00698087	Non	-0.058766667	0.000708908	0.002959361	Non	-0.058416667	0.00246087	0.005189188	Non	-0.061166667	0.002700125	0.004610473	Non	-0.061966667	0.000491909	0.001000342	Non	-0.04862381	0.000000341	0.000000695	Non	RNU11	0
chr1	28994529	28996561	2032	cUMR	-0.019766667	0.0000673	0.000129118	Non	-0.004566667	0.430079893	0.500997543	Non	0.0159	0.043546831	0.064821269	Non	-0.002166667	0.392211061	0.475571206	Non	-0.001516667	0.010290585	0.018199215	Non	-0.016766667	0.011821463	0.017419202	Non	0.007833333	0.219098262	0.253548733	Non	-0.002595238	0.001252189	0.001516649	Non	GMEB1	0
chr1	29062431	29064930	2499	cUMR	0.000166667	0.595703894	0.60706527	Non	0.002833333	0.848184979	0.874458283	Non	0.014	0.028285339	0.044402326	Non	-0.012166667	0.018676278	0.041400141	Non	-0.002666667	0.584286896	0.626815012	Non	-0.011416667	0.055398932	0.070538068	Non	-0.004966667	0.006414196	0.010343069	Non	-0.00122381	0.023668533	0.025615931	Non	YTHDF2	0
chr1	29101393	29102578	1185	cUMR	-0.1453	0.00000234	0.00000623	Hypomethylated	-0.008033333	0.009250519	0.021171028	Non	0.431033333	0.00000000000194	0.0000000000492	Hypermethylated	-0.107433333	0.001013291	0.003966584	Non	-0.111133333	0.002724075	0.005679104	Non	-0.043633333	0.267122448	0.296619019	Non	0.142366667	0.0000003	0.00000129	Hypermethylated	0.035138095	0.00000000219	0.00000000707	Non	YTHDF2	-5107
chr1	29111481	29112230	749	cUMR	0.0335	0.309587654	0.324691791	Non	0.044933333	0.099993871	0.152204682	Non	0.1105	0.000465319	0.001201849	Non	0.023333333	0.479309374	0.557774643	Non	0.089083333	0.017128194	0.028491421	Non	0.122833333	0.000948745	0.001795701	Non	0.029533333	0.482033184	0.516661696	Non	0.06287619	0.000155823	0.000209837	Non	YTHDF2	-15195
chr1	29137528	29140077	2549	cUMR	-0.0811	0.0000267	0.000056	Non	-0.029366667	0.082239887	0.129830619	Non	-0.086766667	0.00000377	0.0000174	Non	-0.026166667	0.316966441	0.401950076	Non	0.030983333	0.250349822	0.297555118	Non	0.163733333	0.00000000194	0.0000000138	Hypermethylated	0.050433333	0.0000407	0.000105724	Non	-0.007252381	0.0000000482	0.000000116	Non	OPRD1	0
chr1	29141832	29142681	849	cUMR	-0.214066667	0.0000000364	0.000000149	Hypomethylated	0.1607	0.000604381	0.002066074	Non	-0.035733333	0.000707563	0.001733867	Non	0.0741	0.010631946	0.026146291	Non	-0.1039	0.000691835	0.001717153	Non	0.0286	0.295576284	0.32561359	Non	-0.0131	0.0000203	0.0000564	Non	-0.019757143	0.000000128	0.000000282	Non	OPRD1	0
chr1	29202927	29204252	1325	cUMR	-0.280433333	0.000000000014	0.00000000014	Hypomethylated	-0.087066667	0.040091489	0.071471822	Non	-0.1221	0.000732982	0.001786889	Non	-0.005466667	0.146154044	0.216180957	Non	-0.201016667	0.00000269	0.0000136	Hypomethylated	-0.195766667	0.0000361	0.0000959	Hypomethylated	-0.174066667	0.0000402	0.000104453	Hypomethylated	-0.152438095	0.000000000339	0.00000000132	Hypomethylated	EPB41	9351
chr1	29208200	29209002	802	cUMR	-0.116566667	0.00000000984	0.0000000469	Hypomethylated	-0.024266667	0.341755636	0.414041507	Non	-0.074566667	0.00000779	0.0000328	Non	-0.075066667	0.000615437	0.002648832	Non	-0.078566667	0.001256806	0.002878672	Non	-0.075566667	0.000637083	0.001255514	Non	0.125333333	0.0000000482	0.000000254	Hypermethylated	-0.046666667	0.00000000693	0.00000002	Non	EPB41	4601
chr1	29211252	29211901	649	cUMR	-0.102181045	0.000145375	0.000260465	Hypomethylated	0.070266667	0.343996956	0.416087473	Non	0.1428	0.026565666	0.042064486	Non	-0.120933333	0.007625923	0.019990954	Non	-0.170996341	0.0000031	0.0000153	Hypomethylated	-0.142747443	0.0000742	0.000183038	Hypomethylated	0.027866667	0.211677568	0.245713476	Non	-0.032148018	0.0000178	0.0000274	Non	EPB41	1702
chr1	29213046	29215045	1999	cUMR	-0.054766667	0.000000902	0.00000265	Non	-0.004433333	0.678549649	0.727422853	Non	0.079566667	0.0000215	0.0000804	Non	-0.039833333	0.001861344	0.006470668	Non	-0.035933333	0.000988225	0.00233473	Non	-0.029683333	0.008675943	0.013169116	Non	-0.018433333	0.029404888	0.041067651	Non	-0.012204762	0.00000269	0.00000467	Non	EPB41	0
chr1	29240486	29242135	1649	cUMR	-0.013466667	0.021833327	0.026625272	Non	-0.000133333	0.29487627	0.36809125	Non	-0.007133333	0.347189921	0.399741171	Non	-0.006733333	0.16806195	0.241846224	Non	-0.006633333	0.432770801	0.482038862	Non	-0.004383333	0.188365341	0.215353829	Non	0.032666667	0.000702428	0.001383111	Non	-0.001104762	0.056696225	0.059944965	Non	EPB41	0
chr1	29447741	29451770	4029	Canyon	0.0028	0.001413605	0.002101354	Non	0.1002	0.00000353	0.0000231	Hypermethylated	0.177466667	0.000000000179	0.00000000264	Hypermethylated	-0.0166	0.188983761	0.265810464	Non	0.1053	0.000000325	0.00000222	Hypermethylated	0.2118	0.00000000000926	0.000000000125	Hypermethylated	0.0808	0.0000263	0.0000711	Non	0.090628571	0.0000000000177	0.0000000000951	Non	TMEM200B	0
chr1	29459956	29461405	1449	cUMR	-0.381033333	0.000000000009	0.0000000000954	Hypomethylated	0.1257	0.002104256	0.006027133	Non	0.015133333	0.766062381	0.794570827	Non	-0.3103	0.0000000605	0.00000141	Hypomethylated	-0.19245	0.00000257	0.0000131	Hypomethylated	-0.3607	0.0000000217	0.000000123	Hypomethylated	-0.4141	0.0000000000219	0.000000000324	Hypomethylated	-0.211471429	0.0000000000028	0.0000000000188	Hypomethylated	TMEM200B	-9536
chr1	29505667	29509216	3549	Canyon	-0.0391	0.0000461	0.0000915	Non	-0.0239	0.038991446	0.069893392	Non	-0.0041	0.085749082	0.117208246	Non	-0.0207	0.094984047	0.153166861	Non	-0.03485	0.002419577	0.005111955	Non	-0.0191	0.011097137	0.016450468	Non	-0.0339	0.000930477	0.001784099	Non	-0.024785714	0.0000175	0.000027	Non	SRSF4	0
chr1	29529079	29529713	634	cUMR	-0.156033333	0.00000182	0.00000498	Hypomethylated	0.001	0.836967198	0.865113823	Non	-0.041366667	0.38593091	0.437654732	Non	-0.1304	0.0000171	0.000141311	Hypomethylated	-0.08945	0.022579182	0.036245212	Non	-0.08095	0.039507036	0.051972953	Non	0.067	0.180664028	0.213061884	Non	-0.062228571	0.0000682	0.0000961	Non	MECR	0
chr1	29556224	29557820	1596	cUMR	-0.028733333	0.0000733	0.000139533	Non	-0.0142	0.097425481	0.148887736	Non	0.005266667	0.00026406	0.000731751	Non	-0.0158	0.101796408	0.162080598	Non	-0.0169	0.040908154	0.060885968	Non	-0.02915	0.001098201	0.00204754	Non	-0.0292	0.000126304	0.000293455	Non	-0.017742857	0.00000356	0.00000607	Non	MECR	0
chr1	29562324	29566273	3949	Canyon	-0.038933333	0.0000000757	0.000000287	Non	-0.015633333	0.109116937	0.163346639	Non	0.126566667	0.00000000000582	0.000000000131	Hypermethylated	-0.033433333	0.0000137	0.000118198	Non	-0.019183333	0.002107522	0.004521131	Non	0.049316667	0.00000000792	0.0000000491	Non	0.004166667	0.001214906	0.002273914	Non	0.012052381	0.0000000000396	0.000000000193	Non	PTPRU	0
chr1	29585747	29586996	1249	cUMR	0.613733333	0.00000000000000411	0.000000000000104	Hypermethylated	-0.014333333	0.240784547	0.312180416	Non	0.332566667	0.0000000221	0.000000188	Hypermethylated	0.525066667	0.0000000000376	0.00000000517	Hypermethylated	0.467066667	0.00000000379	0.0000000531	Hypermethylated	0.224566667	0.0000522	0.000133503	Hypermethylated	0.093666667	0.050473818	0.067198029	Non	0.327609524	0.000000000000617	0.00000000000502	Hypermethylated	PTPRU	0
chr1	29705738	29706287	549	cUMR	-0.115733333	0.0000135	0.0000302	Hypomethylated	-0.001533333	0.079642247	0.126508848	Non	-0.019733333	0.024939617	0.039772013	Non	-0.044133333	0.28086245	0.36517877	Non	0.037016667	0.028892783	0.044916992	Non	0.025266667	0.00000943	0.0000289	Non	0.096466667	0.020927765	0.030154604	Non	-0.008847619	0.00000841	0.0000136	Non	LOC101928460	-29113
chr1	29716302	29717751	1449	cUMR	-0.259433333	0.000000202	0.000000692	Hypomethylated	-0.101566667	0.091555864	0.141629538	Non	-0.0276	0.004204546	0.008305012	Non	-0.206766667	0.0000944	0.000570463	Hypomethylated	-0.056266667	0.350040171	0.399575926	Non	-0.243016667	0.0000234	0.000065	Hypomethylated	-0.219166667	0.0000321	0.0000852	Hypomethylated	-0.158766667	0.000000108	0.000000242	Hypomethylated	LOC101928460	-39677
chr1	30176230	30176855	625	cUMR	-0.1488	0.0000383	0.0000773	Hypomethylated	-0.1319	0.000856521	0.002789512	Non	-0.036966667	0.000212459	0.000604038	Non	-0.1417	0.000402699	0.00187594	Non	-0.0903	0.037789947	0.056781528	Non	-0.13055	0.003245314	0.005427927	Non	-0.1497	0.0000417	0.000108027	Hypomethylated	-0.117557143	0.000000251	0.000000525	Hypomethylated	LOC101929406	309944
chr1	30179572	30180671	1099	cUMR	-0.048533333	0.000690027	0.0010849	Non	0.052066667	0.028919085	0.054819319	Non	-0.0247	0.000115137	0.000353359	Non	-0.038733333	0.000252045	0.001276272	Non	-0.010033333	0.297232941	0.346542998	Non	0.097966667	0.000111464	0.000263417	Non	-0.010933333	0.2266625	0.261473554	Non	-0.002161905	0.00000599	0.00000985	Non	LOC101929406	306128
chr1	30265600	30266449	849	cUMR	-0.086033333	0.008440594	0.010978056	Non	-0.017633333	0.166919977	0.231231428	Non	-0.004366667	0.692731005	0.727953591	Non	-0.137633333	0.000028	0.000213869	Hypomethylated	-0.088033333	0.002417749	0.005108541	Non	-0.165283333	0.00000446	0.0000148	Hypomethylated	-0.089633333	0.007484123	0.01187626	Non	-0.079433333	0.00000726	0.0000118	Non	LOC101929406	220350
chr1	30558463	30559375	912	cUMR	0.128966667	0.0000000219	0.0000000947	Hypermethylated	0.198166667	0.0000000179	0.000000243	Hypermethylated	0.038466667	0.008590343	0.015548301	Non	-0.025833333	0.285737302	0.370189247	Non	0.164966667	0.00000369	0.0000179	Hypermethylated	0.437216667	0.000000000000004	0.000000000000171	Hypermethylated	0.167966667	0.0000000242	0.000000138	Hypermethylated	0.146138095	0.00000000000266	0.000000000018	Hypermethylated	LOC101929406	-48005
chr1	30852764	30853568	804	cUMR	-0.237266667	0.00000000528	0.0000000269	Hypomethylated	-0.073266667	0.138186684	0.198042377	Non	-0.117766667	0.004418675	0.008669698	Non	-0.225466667	0.00000026	0.00000469	Hypomethylated	-0.158766667	0.000508357	0.001313506	Non	-0.189266667	0.0000146	0.0000427	Hypomethylated	-0.106866667	0.003998076	0.00673125	Non	-0.158580952	0.000000000685	0.0000000025	Hypomethylated	MATN1	330556
chr1	31157773	31158861	1088	cUMR	0.023633333	0.00196981	0.002852501	Non	0.2977	0.00000000295	0.0000000538	Hypermethylated	0.1553	0.0000609	0.00020248	Hypermethylated	-0.0085	0.258042629	0.341421582	Non	0.15855	0.00000118	0.00000671	Hypermethylated	0.0353	0.086756062	0.106161965	Non	0.0623	0.100675758	0.125439944	Non	0.103042857	0.0000000448	0.000000109	Hypermethylated	MATN1	25263
chr1	31180577	31181246	669	cUMR	-0.058133333	0.000163081	0.000289348	Non	0.047733333	0.128239819	0.186347158	Non	-0.043633333	0.072300998	0.100968327	Non	-0.021666667	0.724957575	0.773729025	Non	-0.022716667	0.543119035	0.587911351	Non	0.003033333	0.964282552	0.967091766	Non	0.059733333	0.001704675	0.003081228	Non	-0.007438095	0.00180493	0.002150141	Non	MATN1	2878
chr1	31190311	31192717	2406	cUMR	-0.050133333	0.000163019	0.000289302	Non	0.0133	0.233101974	0.304183811	Non	-0.040133333	0.001065382	0.002488231	Non	-0.0163	0.454803789	0.534432279	Non	-0.0393	0.022808143	0.036566472	Non	-0.0173	0.169307165	0.195344444	Non	-0.0545	0.000119014	0.000278262	Non	-0.030157143	0.0000405	0.000059	Non	MATN1	0
chr1	31255012	31256095	1083	cUMR	-0.0738	0.001446751	0.002146125	Non	-0.0365	0.127944628	0.186064073	Non	-0.0888	0.000166086	0.000488328	Non	-0.0495	0.015064138	0.03465212	Non	-0.0393	0.110202599	0.145023058	Non	-0.0743	0.010484863	0.015630326	Non	-0.0031	0.001294256	0.002409332	Non	-0.053585714	0.0000235	0.0000354	Non	LAPTM5	-24330
chr1	31274358	31275157	799	cUMR	-0.040066667	0.00000272	0.00000712	Non	0.009766667	0.572452776	0.632712965	Non	-0.0089	0.252293839	0.302807896	Non	-0.028833333	0.000612206	0.002637279	Non	-0.010983333	0.342263113	0.391859393	Non	-0.047202049	0.00000000000037	0.00000000000786	Non	0.010366667	0.0000101	0.00003	Non	-0.016286454	0.000000000978	0.00000000343	Non	LAPTM5	-43676
chr1	31295912	31298911	2999	cUMR	-0.091166667	0.00000000355	0.0000000189	Non	-0.015	0.343279646	0.415407504	Non	-0.048	0.000247476	0.000690559	Non	-0.051	0.000666955	0.002821183	Non	-0.0385	0.0000131	0.0000542	Non	-0.036	0.053082122	0.067875039	Non	0.0098	0.043726602	0.0589282	Non	-0.0404	0.000000147	0.00000032	Non	SDC3	43402
chr1	31380257	31382173	1916	cUMR	-0.013966667	0.006973429	0.009195889	Non	0.006666667	0.769815768	0.807656329	Non	-0.0253	0.002569628	0.00540245	Non	-0.011733333	0.015876839	0.036189942	Non	0.021116667	0.26247537	0.310344511	Non	-0.001383333	0.766441146	0.781058854	Non	0.029266667	0.058511859	0.076708474	Non	-0.001019048	0.017110417	0.018739573	Non	SDC3	0
chr1	31535304	31539053	3749	Canyon	-0.036966667	0.000201015	0.000350235	Non	-0.026966667	0.031896148	0.059392743	Non	0.003533333	0.5079717	0.555479513	Non	-0.033166667	0.001215196	0.004605094	Non	-0.043216667	0.000335825	0.0009134	Non	-0.054466667	0.000000544	0.00000225	Non	-0.056766667	0.0000000000823	0.000000000994	Non	-0.033595238	0.000000057	0.000000135	Non	PUM1	0
chr1	31627140	31628739	1599	cUMR	-0.051966667	0.00000102	0.00000295	Non	-0.0337	0.006861286	0.016404049	Non	0.140366667	0.00000000252	0.0000000277	Hypermethylated	-0.0381	0.000670393	0.002832744	Non	-0.0338	0.019865409	0.032402641	Non	-0.0353	0.0000582	0.000147229	Non	-0.0501	0.0000134	0.0000388	Non	-0.010157143	0.000000151	0.000000328	Non	NKAIN1	23853
chr1	31635337	31636077	740	cUMR	-0.1642	0.0000000325	0.000000135	Hypomethylated	-0.1009	0.001187154	0.003673197	Non	0.0103	0.152416919	0.194613624	Non	-0.0633	0.057923699	0.103255687	Non	-0.0797	0.022926154	0.036716585	Non	-0.1057	0.00204053	0.00358506	Non	-0.1193	0.0000938	0.000224362	Hypomethylated	-0.088071429	0.000000227	0.000000477	Non	NKAIN1	16515
chr1	31654262	31656211	1949	cUMR	-0.071666667	0.000441863	0.000719574	Non	0.138133333	0.00037435	0.001369056	Non	0.390666667	0.00000000000551	0.000000000125	Hypermethylated	-0.072066667	0.000516012	0.002300196	Non	-0.030416667	0.311585876	0.360850973	Non	0.049583333	0.024074629	0.03319318	Non	-0.032666667	0.027353927	0.038434907	Non	0.061647619	0.0000000127	0.0000000346	Non	NKAIN1	0
chr1	31711552	31713201	1649	cUMR	-0.013666667	0.002846852	0.004010959	Non	0.0254	0.001792393	0.005243571	Non	-0.001833333	0.369476553	0.421753872	Non	0.0014	0.161617897	0.234576166	Non	0.01775	0.016986711	0.028287316	Non	0.15575	0.0000000000000643	0.00000000000175	Hypermethylated	0.0068	0.014574814	0.021761973	Non	0.021971429	0.00000000125	0.00000000425	Non	NKAIN1	0
chr1	31768709	31770944	2235	cUMR	-0.017533333	0.000058	0.000112875	Non	-0.006	0.248985544	0.320707968	Non	-0.014366667	0.0000109	0.0000442	Non	-0.0074	0.313839784	0.398891847	Non	-0.0172	0.000973775	0.002306698	Non	-0.0192	0.000262123	0.000565704	Non	-0.01	0.001716358	0.003100252	Non	-0.012971429	0.000000429	0.000000858	Non	SNRNP40	0
chr1	31845710	31846959	1249	cUMR	-0.035866667	0.155903439	0.169461483	Non	0.3435	0.000000237	0.00000221	Hypermethylated	0.028133333	0.520991101	0.567784169	Non	-0.0285	0.238069056	0.320026226	Non	0.0698	0.113147957	0.148412773	Non	0.03755	0.45467324	0.483057743	Non	0.2245	0.0000302	0.0000807	Hypermethylated	0.088014286	0.0000499	0.0000717	Non	FABP3	0
chr1	31867461	31868110	649	cUMR	-0.021667774	0.0000000187	0.0000000829	Non	-0.003133333	0.821223788	0.851761575	Non	0.021966667	0.012870942	0.02219841	Non	-0.022733333	0.00572102	0.015940008	Non	-0.012533333	0.03582329	0.054194843	Non	-0.019533333	0.018156101	0.025638777	Non	0.014266667	0.089383381	0.112604274	Non	-0.005272295	0.00000343	0.00000586	Non	SERINC2	14302
chr1	31885025	31888174	3149	cUMR	-0.0652	0.000000435	0.00000137	Non	-0.0454	0.000446563	0.001593016	Non	0.021133333	0.120175586	0.157819688	Non	-0.037	0.0000751	0.000476553	Non	-0.0387	0.006288503	0.011847648	Non	-0.03945	0.005926922	0.00934909	Non	-0.0336	0.01150439	0.017527599	Non	-0.033057143	0.0000225	0.000034	Non	SERINC2	0
chr1	31895249	31895970	721	cUMR	-0.0988	0.000782895	0.001217882	Non	-0.053233333	0.004158984	0.010799187	Non	-0.0258	0.220981893	0.269672439	Non	-0.097433333	0.001943877	0.006699592	Non	-0.119133333	0.001144686	0.002656969	Non	-0.067633333	0.025980285	0.03552993	Non	0.049766667	0.331697677	0.368780844	Non	-0.057119048	0.000252204	0.000330643	Non	SERINC2	0
chr1	32013984	32015722	1738	cUMR	-0.1532	0.000000161	0.000000567	Hypomethylated	0.0228	0.769621322	0.807550462	Non	0.0318	0.020246087	0.033135857	Non	-0.0984	0.003679594	0.011156795	Non	-0.07795	0.006558471	0.012272474	Non	-0.10695	0.004652092	0.007510793	Non	-0.0072	0.289807741	0.326333348	Non	-0.053771429	0.0000458	0.0000662	Non	LINC01226	-24139
chr1	32054311	32055287	976	cUMR	-0.173633333	0.00000762	0.000018	Hypomethylated	0.299566667	0.000039	0.00019068	Hypermethylated	0.351033333	0.000000565	0.00000324	Hypermethylated	-0.101433333	0.0385707	0.074209719	Non	0.092616667	0.001972231	0.004268621	Non	-0.162383333	0.000963972	0.001821759	Non	0.046566667	0.00066538	0.001317312	Non	0.057395238	0.00000206	0.00000364	Non	TINAGL1	-1025
chr1	32082934	32084183	1249	cUMR	-0.009666667	0.071712896	0.081247643	Non	0.1094	0.0000000126	0.000000182	Hypermethylated	0.0065	0.009858788	0.01756967	Non	-0.0048	0.658285005	0.716275434	Non	0.0125	0.301226713	0.350420533	Non	0.10825	0.0000000685	0.000000347	Hypermethylated	0.0712	0.00000195	0.00000684	Non	0.038371429	0.0000000197	0.0000000515	Non	HCRTR1	0
chr1	32109451	32111300	1849	cUMR	-0.017133333	0.0000841	0.000158142	Non	0.005933333	0.336539344	0.409017429	Non	-0.000966667	0.00020131	0.000576216	Non	0.008733333	0.105124768	0.166290548	Non	-0.006216667	0.022292579	0.035840054	Non	-0.016466667	0.003927602	0.006456164	Non	-0.011066667	0.0000123	0.0000357	Non	-0.005180952	0.00000295	0.0000051	Non	PEF1	0
chr1	32135034	32136233	1199	cUMR	-0.084533333	0.00050962	0.000821405	Non	-0.032633333	0.328394932	0.400857742	Non	-0.031533333	0.238060216	0.287773873	Non	-0.072433333	0.006650714	0.017925166	Non	-0.093033333	0.001018999	0.002400141	Non	-0.060783333	0.060422844	0.076317925	Non	-0.029433333	0.000461233	0.000944575	Non	-0.056690476	0.0000544	0.0000777	Non	COL16A1	0
chr1	32169311	32171173	1862	cUMR	0.033733333	0.025546231	0.030825351	Non	0.177066667	0.000750586	0.002484801	Non	0.0959	0.066701538	0.0941416	Non	-0.043533333	0.204200205	0.282884039	Non	0.138816667	0.010466977	0.018462957	Non	0.007816667	0.191805377	0.218902485	Non	0.001866667	0.110078643	0.136030309	Non	0.05832381	0.006168619	0.007002609	Non	COL16A1	0
chr1	32179851	32181850	1999	cUMR	-0.046366667	0.000852946	0.001318259	Non	0.2569	0.0000000513	0.000000595	Hypermethylated	0.3608	0.0000000000303	0.000000000545	Hypermethylated	-0.0375	0.002612777	0.008496991	Non	0.27205	0.0000000172	0.000000184	Hypermethylated	0.04505	0.041878013	0.054757264	Non	0.1089	0.000037	0.0000971	Hypermethylated	0.137042857	0.000000000204	0.000000000837	Hypermethylated	COL16A1	-10084
chr1	32225421	32227074	1653	cUMR	-0.029133333	0.033703157	0.039949762	Non	0.088266667	0.0000315	0.000157827	Non	-0.023633333	0.054735313	0.079186623	Non	-0.022133333	0.062131879	0.109216866	Non	0.093366667	0.00000483	0.0000227	Non	0.031366667	0.127078897	0.150469107	Non	0.037466667	0.00612048	0.009909231	Non	0.020009524	0.0000202	0.0000307	Non	BAI2	0
chr1	32227605	32230954	3349	cUMR	-0.023733333	0.002689326	0.003807272	Non	0.003833333	0.665136397	0.715143305	Non	-0.028733333	0.000138479	0.000415731	Non	-0.004166667	0.022567449	0.048239148	Non	0.037933333	0.002012172	0.004343364	Non	0.083183333	0.000000202	0.000000921	Non	0.012833333	0.361389296	0.398464193	Non	0.006633333	0.0000344	0.0000506	Non	BAI2	0
chr1	32237514	32238663	1149	cUMR	0.0602	0.0000917	0.000171118	Non	0.568266667	0.00000000000096	0.0000000000956	Hypermethylated	0.0787	0.013838804	0.023666702	Non	0.137066667	0.000103072	0.000614199	Hypermethylated	0.284366667	0.0000000568	0.000000501	Hypermethylated	0.376116667	0.0000000463	0.000000243	Hypermethylated	0.263066667	0.000000344	0.00000146	Hypermethylated	0.237638095	0.0000000000531	0.00000000025	Hypermethylated	BAI2	-7851
chr1	32253686	32255285	1599	cUMR	-0.0256	0.0000323	0.0000663	Non	-0.001966667	0.741462016	0.78298572	Non	-0.012433333	0.110141385	0.145970803	Non	-0.011366667	0.23162318	0.313217202	Non	-0.019766667	0.013207728	0.022690513	Non	-0.015266667	0.037617296	0.049709454	Non	0.048633333	0.00000356	0.0000117	Non	-0.005480952	0.0000689	0.0000971	Non	SPOCD1	738
chr1	32409714	32410813	1099	cUMR	-0.015833333	0.452797249	0.467002273	Non	0.5113	0.00000000162	0.0000000319	Hypermethylated	0.334666667	0.00000685	0.0000293	Hypermethylated	-0.0593	0.327447022	0.412483969	Non	0.23025	0.002815235	0.005842868	Non	-0.09525	0.013952338	0.02022275	Non	0.3659	0.0000025	0.00000855	Hypermethylated	0.186928571	0.00000102	0.00000191	Hypermethylated	PTP4A2	-5727
chr1	32429613	32430168	555	cUMR	-0.170566667	0.0000235	0.0000497	Hypomethylated	-0.059166667	0.326928281	0.399541191	Non	0.039266667	0.424582846	0.475622788	Non	-0.139766667	0.000109067	0.000643245	Hypomethylated	-0.072066667	0.187391544	0.230855346	Non	-0.260816667	0.00000000847	0.0000000522	Hypomethylated	-0.113766667	0.006089379	0.009863517	Non	-0.10522381	0.00000207	0.00000367	Hypomethylated	PTP4A2	-25626
chr1	32469270	32469421	151	cUMR	0.007330211	0.018010807	0.022258805	Non	0.021466667	0.657291701	0.708193969	Non	0.1838	0.0000816	0.000261527	Hypermethylated	0.006502644	0.015166542	0.034837796	Non	0.197216667	0.000347523	0.000939807	Hypermethylated	-0.033283333	0.206492328	0.234476115	Non	0.103266667	0.003921564	0.006611671	Non	0.070245566	0.0000353	0.0000518	Non	KHDRBS1	9874
chr1	32478681	32480925	2244	cUMR	-0.003833333	0.116131769	0.128350837	Non	-0.000533333	0.951942375	0.960914687	Non	0.001166667	0.512769445	0.559862175	Non	-0.004133333	0.161716153	0.234676374	Non	-0.0000833	0.674611385	0.710710202	Non	-0.005583333	0.021234169	0.029586644	Non	0.000266667	0.573801827	0.604932133	Non	-0.001733333	0.362724382	0.368039249	Non	KHDRBS1	0
chr1	32537882	32539431	1549	cUMR	0.004533333	0.31801363	0.333093948	Non	0.0106	0.045988502	0.080158043	Non	-0.024966667	0.000885243	0.002113184	Non	0.0108	0.347063262	0.431799809	Non	0.0092	0.062786938	0.088518085	Non	0.00895	0.081066239	0.099802505	Non	0.0118	0.340767922	0.377871322	Non	0.003314286	0.668734788	0.672025476	Non	TMEM39B	0
chr1	32573017	32575266	2249	cUMR	-0.060133333	0.0000869	0.000162896	Non	-0.020333333	0.217583035	0.287974642	Non	-0.0118	0.016035174	0.026942938	Non	-0.021733333	0.310487703	0.395712538	Non	-0.010133333	0.849580431	0.86828064	Non	-0.048133333	0.006804683	0.01058682	Non	-0.050133333	0.003464555	0.005903537	Non	-0.032161905	0.000332282	0.000429463	Non	KPNA6	0
chr1	32644760	32646809	2049	cUMR	-0.026166667	0.0000335	0.0000684	Non	-0.002233333	0.66354512	0.713687144	Non	0.007833333	0.194426421	0.24086407	Non	-0.010233333	0.286732841	0.37114938	Non	-0.012333333	0.213599076	0.258945091	Non	-0.009583333	0.285890646	0.315802348	Non	-0.005233333	0.152492984	0.182664095	Non	-0.00817619	0.006537924	0.00740684	Non	TXLNA	0
chr1	32665099	32667392	2293	cUMR	-0.022333333	0.033033398	0.039198287	Non	0.1224	0.000000437	0.00000377	Hypermethylated	-0.028666667	0.044138556	0.06556842	Non	0.0646	0.002645318	0.008580815	Non	0.08475	0.000126928	0.000389049	Non	0.02875	0.232099958	0.260606876	Non	0.1126	0.00000136	0.00000498	Hypermethylated	0.047028571	0.00000274	0.00000475	Non	CCDC28B	0
chr1	32670486	32671884	1398	cUMR	-0.071933333	0.0000389	0.0000784	Non	-0.039233333	0.029297649	0.055410521	Non	0.007066667	0.917477592	0.928224317	Non	-0.045633333	0.049014586	0.09026085	Non	-0.073933333	0.0000418	0.000147781	Non	-0.031433333	0.21878167	0.24690679	Non	-0.049233333	0.000703357	0.001384421	Non	-0.042319048	0.0000191	0.0000292	Non	IQCC	0
chr1	32686716	32688715	1999	cUMR	-0.032	0.0000576	0.000112163	Non	-0.0112	0.162707574	0.22638515	Non	-0.022166667	0.012116216	0.021040325	Non	-0.0174	0.025887581	0.053895371	Non	-0.03375	0.000374412	0.001001619	Non	-0.02375	0.006309317	0.009882927	Non	-0.0338	0.0000146	0.000042	Non	-0.024771429	0.000000434	0.000000867	Non	EIF3I	0
chr1	32704967	32707434	2467	cUMR	-0.0532	0.0000636	0.000122822	Non	0.0264	0.576472787	0.636046921	Non	-0.003533333	0.63717145	0.677098276	Non	0.0048	0.312275453	0.397528466	Non	0.02055	0.001737159	0.003819532	Non	0.00505	0.175445876	0.20183772	Non	-0.0008	0.736049122	0.757736896	Non	-0.002457143	0.001850578	0.002201364	Non	MTMR9LP	0
chr1	32713899	32714498	599	cUMR	-0.024466667	0.421928387	0.436378982	Non	0.038066667	0.568537328	0.629308405	Non	0.235033333	0.0000022	0.0000108	Hypermethylated	-0.021933333	0.654753145	0.713110218	Non	0.089366667	0.049037507	0.071286187	Non	-0.004633333	0.3478803	0.378082329	Non	0.171266667	0.00000493	0.0000157	Hypermethylated	0.072552381	0.0000232	0.000035	Non	FAM167B	0
chr1	32756880	32758529	1649	cUMR	-0.048566667	0.0000525	0.000103148	Non	0.073733333	0.0000685	0.000312486	Non	0.0731	0.000443449	0.001151906	Non	-0.022266667	0.123181403	0.18860318	Non	-0.038316667	0.00393282	0.007819562	Non	-0.055816667	0.000011	0.0000331	Non	-0.024066667	0.065430036	0.084918784	Non	-0.002638095	0.00000687	0.0000112	Non	HDAC1	0
chr1	32799431	32802430	2999	cUMR	-0.0109	0.011770185	0.014959805	Non	-0.014266667	0.164550421	0.228421654	Non	-0.017733333	0.15637276	0.199147594	Non	0.020933333	0.27007097	0.353858755	Non	-0.002316667	0.680027124	0.715914969	Non	-0.022316667	0.105493043	0.126987929	Non	0.006133333	0.52314544	0.556101665	Non	-0.005895238	0.059463143	0.062787785	Non	MARCKSL1	0
chr1	32816651	32817950	1299	cUMR	-0.019233333	0.061144396	0.069914954	Non	-0.0049	0.221925576	0.292497982	Non	-0.006233333	0.779080873	0.805731254	Non	0.0017	0.512200892	0.587538895	Non	-0.00715	0.048410233	0.070450498	Non	-0.02115	0.092274866	0.112361785	Non	-0.0203	0.004892737	0.008072263	Non	-0.010957143	0.055485244	0.058698073	Non	FAM229A	8921
chr1	32827145	32828230	1085	cUMR	-0.030566667	0.062761436	0.071638171	Non	0.003766667	0.006156903	0.01497813	Non	0.3431	0.000000000000116	0.00000000000438	Hypermethylated	0.067366667	0.000194469	0.001031774	Non	0.119266667	0.00000953	0.000041	Hypermethylated	0.040016667	0.009411424	0.014189857	Non	0.023766667	0.041452765	0.056114934	Non	0.085338095	0.000000000701	0.00000000255	Non	FAM229A	0
chr1	32858951	32861350	2399	cUMR	-0.0477	0.00000000374	0.0000000198	Non	-0.024166667	0.005785255	0.014228138	Non	-0.007366667	0.053368443	0.077418296	Non	-0.001166667	0.080198091	0.134041725	Non	-0.024866667	0.004147105	0.008190881	Non	-0.030116667	0.00392525	0.006453618	Non	-0.021766667	0.00020797	0.000458527	Non	-0.022452381	0.0000000825	0.000000189	Non	BSDC1	0
chr1	32866635	32866834	199	cUMR	-0.028574623	0.021915289	0.026719874	Non	0.042033333	0.467169347	0.536218908	Non	0.0109	0.638551621	0.678295732	Non	-0.036136098	0.010976584	0.026818789	Non	0.028983333	0.682872374	0.718439575	Non	-0.034434877	0.000334769	0.000704011	Non	-0.008566667	0.836013967	0.850305708	Non	-0.004034337	0.002334832	0.002749989	Non	BSDC1	-6574
chr1	32892020	32893569	1549	cUMR	-0.076566667	0.0000000042	0.000000022	Non	0.202133333	0.000000712	0.00000577	Hypermethylated	-0.007733333	0.14498474	0.186100045	Non	-0.021866667	0.061341646	0.108103772	Non	0.040183333	0.000000638	0.00000394	Non	0.149433333	0.000135185	0.000313421	Hypermethylated	-0.137666667	0.0000000000745	0.000000000916	Hypomethylated	0.013304762	0.0000000000492	0.000000000234	Non	BSDC1	-31959
chr1	32929562	32931468	1906	cUMR	-0.0588	0.0000000172	0.0000000771	Non	0.186	0.000000000498	0.0000000121	Hypermethylated	-0.021633333	0.000604253	0.001510664	Non	0.004	0.056622568	0.101501945	Non	0.02445	0.000483881	0.001255685	Non	0.4097	0.000000000000000999	0.0000000000000528	Hypermethylated	0.0638	0.000178762	0.000400319	Non	0.072085714	0.000000000000421	0.00000000000361	Non	ZBTB8B	0
chr1	32965473	32966772	1299	cUMR	-0.019133333	0.001553687	0.002290934	Non	-0.005733333	0.479368392	0.547223466	Non	0.0032	0.591513557	0.634687738	Non	-0.007133333	0.39223934	0.475581566	Non	-0.013383333	0.109220503	0.143872072	Non	-0.002383333	0.911829462	0.918000768	Non	-0.002933333	0.890119458	0.900584416	Non	-0.006790476	0.058764561	0.062074624	Non	ZBTB8B	-12015
chr1	32985817	32986666	849	cUMR	-0.173966667	0.0000000948	0.000000351	Hypomethylated	-0.097466667	0.011341714	0.025046398	Non	0.1137	0.000000324	0.00000198	Hypermethylated	-0.064266667	0.023549506	0.049898532	Non	-0.127466667	0.001188879	0.002744514	Non	-0.093966667	0.024393688	0.03358121	Non	-0.159866667	0.00000752	0.0000231	Hypomethylated	-0.081580952	0.00000026	0.00000054	Non	ZBTB8A	18080
chr1	33004413	33006162	1749	cUMR	-0.0236	0.000431537	0.000703931	Non	-0.012433333	0.141510939	0.201846164	Non	0.0259	0.020648923	0.033716057	Non	-0.006233333	0.58476311	0.651545285	Non	-0.008433333	0.208719171	0.253552179	Non	-0.026433333	0.001835796	0.003258619	Non	0.007566667	0.065148651	0.084590037	Non	-0.00517619	0.003354907	0.003896219	Non	ZBTB8A	0
chr1	33077373	33078622	1249	cUMR	-0.0927	0.00000000353	0.0000000188	Non	-0.049933333	0.00259723	0.007203666	Non	0.195633333	0.00000000000962	0.000000000202	Hypermethylated	-0.058333333	0.000244985	0.001247599	Non	-0.036533333	0.031266433	0.048119953	Non	-0.009783333	0.012909535	0.018850752	Non	-0.081333333	0.00000064	0.00000254	Non	-0.014733333	0.000000000855	0.00000000304	Non	ZBTB8A	-5823
chr1	33115158	33117860	2702	cUMR	-0.035266667	0.0000000904	0.000000337	Non	-0.0192	0.012651519	0.027438003	Non	-0.008266667	0.000132404	0.000399033	Non	-0.0236	0.001619477	0.005791948	Non	-0.01935	0.002885126	0.005968312	Non	-0.0446	0.000000000827	0.0000000064	Non	-0.0522	0.0000000000129	0.000000000203	Non	-0.028342857	0.0000000000414	0.000000000201	Non	ZBTB8OS	0
chr1	33167536	33169335	1799	cUMR	-0.026266667	0.036674743	0.043212789	Non	0.0071	0.500887914	0.56743435	Non	-0.008266667	0.5231288	0.569753133	Non	-0.0237	0.104585998	0.165636862	Non	-0.01935	0.290783119	0.339994741	Non	0.02415	0.35864164	0.388568754	Non	0.0175	0.504831729	0.538749517	Non	-0.005242857	0.312656044	0.317946801	Non	SYNC	0
chr1	33177613	33178262	649	cUMR	-0.085433333	0.00028147	0.000475138	Non	-0.0696	0.004443271	0.011413441	Non	0.290566667	0.000000000342	0.00000000466	Hypermethylated	-0.0398	0.251222702	0.334399286	Non	-0.0521	0.113651402	0.148935314	Non	-0.0786	0.006171463	0.00969175	Non	-0.0476	0.128506032	0.156514423	Non	-0.0022	0.000000939	0.00000177	Non	SYNC	-9253
chr1	33182332	33182943	611	cUMR	-0.111033333	0.001601406	0.002355963	Non	-0.0832	0.003753364	0.009885975	Non	0.138966667	0.01746629	0.029063849	Non	-0.126370111	0.000104535	0.00062077	Hypomethylated	-0.107170128	0.000808624	0.001968902	Non	-0.1027	0.004258196	0.006937597	Non	-0.1108	0.001316875	0.002447467	Non	-0.064963638	0.000247795	0.000325308	Non	SYNC	-13972
chr1	33206504	33209703	3199	cUMR	-0.068633333	0.0000935	0.000174124	Non	-0.052966667	0.004059267	0.010575863	Non	0.078866667	0.005296013	0.01017162	Non	-0.048966667	0.014113407	0.032873079	Non	-0.064966667	0.001053945	0.002468609	Non	-0.062216667	0.000816934	0.001570145	Non	-0.039566667	0.079154974	0.100805782	Non	-0.032995238	0.000250675	0.000328803	Non	KIAA1522	0
chr1	33218972	33221021	2049	cUMR	-0.072066667	0.000504457	0.00081341	Non	-0.054366667	0.003643993	0.009638782	Non	0.024766667	0.425216875	0.476266617	Non	-0.040766667	0.020437906	0.044478606	Non	-0.061816667	0.051432654	0.074321088	Non	-0.046816667	0.109335293	0.131226312	Non	-0.042366667	0.126661976	0.154494911	Non	-0.040166667	0.007950811	0.008936921	Non	KIAA1522	0
chr1	33281394	33284721	3327	cUMR	-0.034	0.000115275	0.000210719	Non	-0.017766667	0.104247697	0.157495722	Non	-0.007666667	0.004514109	0.008834646	Non	-0.020366667	0.073596701	0.12526722	Non	-0.038916667	0.0000377	0.000135088	Non	-0.060166667	0.000000135	0.000000642	Non	-0.028166667	0.010648506	0.016333719	Non	-0.027938095	0.000000343	0.0000007	Non	YARS	0
chr1	33335627	33338755	3128	cUMR	-0.0307	0.259046071	0.274190373	Non	0.054566667	0.015092778	0.031785586	Non	-0.008533333	0.827928053	0.849249147	Non	0.009766667	0.858547123	0.886087994	Non	0.037466667	0.212198568	0.257428569	Non	0.174466667	0.000000507	0.00000211	Hypermethylated	0.057366667	0.070473127	0.090857168	Non	0.034880952	0.000783221	0.000968596	Non	FNDC5	0
chr1	33350659	33353558	2899	cUMR	-0.118966667	0.000000000411	0.00000000279	Hypomethylated	0.032633333	0.03344605	0.061664075	Non	0.011366667	0.00000169	0.00000852	Non	-0.055566667	0.002259285	0.007563716	Non	-0.062216667	0.00000509	0.0000237	Non	-0.003466667	0.079258101	0.097791292	Non	-0.078966667	0.0000000111	0.0000000691	Non	-0.040509524	0.000000000413	0.00000000158	Non	HPCA	0
chr1	33358182	33359681	1499	cUMR	-0.035533333	0.009197502	0.011883621	Non	0.029366667	0.349836854	0.421965096	Non	0.1848	0.000000000229	0.00000000328	Hypermethylated	-0.015033333	0.569226819	0.637980926	Non	-0.020033333	0.377701178	0.42723634	Non	-0.008283333	0.546197728	0.571924604	Non	0.068366667	0.001085015	0.002051357	Non	0.034166667	0.000000397	0.000000798	Non	HPCA	0
chr1	33365904	33367753	1849	cUMR	-0.072466667	0.0000609	0.000118135	Non	-0.015133333	0.74410467	0.784919026	Non	0.057533333	0.010798554	0.019009683	Non	-0.001133333	0.962840131	0.970613741	Non	-0.011133333	0.005079068	0.009807323	Non	-0.074383333	0.0000458	0.00011858	Non	-0.003933333	0.731492243	0.753346031	Non	-0.015219048	0.000788324	0.000974407	Non	TMEM54	0
chr1	33427603	33431129	3526	Canyon	-0.075966667	0.0000000347	0.000000143	Non	-0.0415	0.011075731	0.024562899	Non	0.002366667	0.92472859	0.934618528	Non	-0.0523	0.000984459	0.003871327	Non	-0.0573	0.000557758	0.001424875	Non	-0.06205	0.0000773	0.000189722	Non	-0.0405	0.000841833	0.001628909	Non	-0.045442857	0.000000203	0.00000043	Non	RNF19B	0
chr1	33438158	33439695	1537	cUMR	-0.073566667	0.000484079	0.000783067	Non	-0.004733333	0.974424868	0.978811791	Non	-0.030066667	0.00651357	0.01219896	Non	-0.061333333	0.014619591	0.033818057	Non	0.089016667	0.0000000126	0.000000143	Non	-0.016733333	0.218795076	0.246910356	Non	-0.027733333	0.058923312	0.077201682	Non	-0.022904762	0.00000307	0.00000527	Non	RNF19B	-7745
chr1	33501233	33502979	1746	cUMR	-0.026766667	0.005371346	0.007224351	Non	-0.021066667	0.006664822	0.016012185	Non	-0.007766667	0.156069722	0.198814197	Non	-0.023866667	0.036270215	0.070515501	Non	-0.028766667	0.010659129	0.018775213	Non	-0.038766667	0.000419046	0.000860958	Non	-0.027666667	0.001050663	0.001991816	Non	-0.024009524	0.0000199	0.0000303	Non	AK2	0
chr1	33546082	33547950	1868	cUMR	-0.023833333	0.006790251	0.008976437	Non	0.050066667	0.063919344	0.105678521	Non	0.076	0.001542893	0.003453844	Non	-0.014533333	0.686833301	0.740871633	Non	0.013666667	0.066219281	0.092797874	Non	0.011166667	0.174696593	0.201071715	Non	-0.001333333	0.070004614	0.090320795	Non	0.016666667	0.001144269	0.001391546	Non	AZIN2	0
chr1	33552028	33552327	299	cUMR	-0.089666667	0.011164128	0.014243602	Non	-0.0247	0.590346124	0.648444277	Non	-0.142789138	0.000000267	0.00000167	Hypomethylated	-0.0431	0.543350786	0.61521718	Non	-0.00125	0.935501176	0.943314004	Non	-0.0845	0.131396556	0.155116554	Non	0.0707	0.000112699	0.000264833	Non	-0.049233361	0.0000275	0.000041	Non	AZIN2	0
chr1	33560594	33561268	674	cUMR	-0.180066667	0.0000000335	0.000000139	Hypomethylated	-0.072566667	0.079524108	0.126354505	Non	0.1506	0.000000458	0.00000268	Hypermethylated	-0.097366667	0.005470594	0.015374391	Non	-0.142316667	0.000297378	0.000819933	Hypomethylated	-0.196066667	0.000000466	0.00000196	Hypomethylated	-0.089166667	0.001924852	0.003443372	Non	-0.080738095	0.0000000244	0.0000000625	Non	AZIN2	0
chr1	33592103	33594102	1999	cUMR	-0.1637	0.00000000000128	0.0000000000167	Hypomethylated	0.021866667	0.299015458	0.372043966	Non	0.140466667	0.0000000234	0.000000198	Hypermethylated	-0.038133333	0.041787922	0.079294345	Non	-0.025783333	0.000000000317	0.00000000706	Non	-0.016783333	0.006356994	0.009949395	Non	0.058866667	0.000295096	0.000628925	Non	-0.002761905	0.0000000000706	0.000000000323	Non	AZIN2	-5972
chr1	33645640	33648539	2899	cUMR	-0.0333	0.0000872	0.000163524	Non	-0.009966667	0.055865885	0.094289883	Non	-0.012966667	0.296365611	0.348398384	Non	-0.008566667	0.15115068	0.222194174	Non	-0.013466667	0.066474311	0.093101125	Non	-0.000966667	0.719582355	0.736796074	Non	0.001633333	0.241498763	0.276931147	Non	-0.011995238	0.02284837	0.024767218	Non	TRIM62	0
chr1	33720810	33723859	3049	cUMR	-0.038333333	0.0000164	0.0000361	Non	-0.0155	0.296127017	0.369308184	Non	0.030166667	0.063323966	0.089948772	Non	-0.0305	0.004675271	0.013546314	Non	-0.0345	0.00126156	0.002887641	Non	-0.03725	0.000529354	0.001063119	Non	-0.0289	0.006400114	0.010321743	Non	-0.0203	0.0000542	0.0000775	Non	ZNF362	0
chr1	33811786	33816735	4949	Canyon	-0.018166667	0.065474222	0.074508774	Non	0.0085	0.174908834	0.240418583	Non	-0.0125	0.07036423	0.098589291	Non	0.0051	0.505398589	0.581563136	Non	-0.0015	0.908519923	0.920475641	Non	-0.0175	0.129651447	0.153229062	Non	0.0055	0.031357024	0.043524127	Non	-0.0047	0.440864446	0.445841262	Non	PHC2	0
chr1	33833171	33833762	591	cUMR	-0.045833333	0.073475936	0.083162985	Non	-0.0116	0.767013558	0.805242265	Non	-0.054333333	0.030700645	0.047659868	Non	-0.0224	0.387288633	0.470779965	Non	0.00475	0.87936668	0.894738146	Non	0.00025	0.889021647	0.896874604	Non	0.0576	0.115480359	0.142117532	Non	-0.013228571	0.486657829	0.491368013	Non	PHC2	0
chr1	33895380	33897280	1900	cUMR	-0.014333333	0.013909165	0.017480212	Non	0.013366667	0.04181639	0.074048321	Non	-0.025166667	0.000181421	0.000526923	Non	-0.015233333	0.013759693	0.032170474	Non	0.008416667	0.083193712	0.113306736	Non	0.046416667	0.00000596	0.0000192	Non	-0.016233333	0.009994612	0.015414118	Non	-0.003090476	0.0000514	0.0000737	Non	ZSCAN20	40952
chr1	33936022	33936772	750	cUMR	-0.036333333	0.032715119	0.038847391	Non	0.0079	0.830750397	0.85960977	Non	-0.011	0.399632507	0.451387118	Non	-0.0235	0.105465744	0.166689414	Non	-0.03625	0.001514884	0.003387997	Non	-0.01175	0.173206828	0.199509495	Non	0.0483	0.0000000213	0.000000123	Non	-0.008928571	0.0000379	0.0000555	Non	ZSCAN20	1460
chr1	33937533	33938832	1299	cUMR	-0.024266667	0.003172068	0.004436477	Non	0.000433333	0.715207681	0.759691417	Non	-0.0321	0.00000534	0.0000236	Non	-0.015766667	0.038374998	0.073909903	Non	-0.027766667	0.004271763	0.008412269	Non	0.050983333	0.000028	0.0000764	Non	-0.023166667	0.008561145	0.013404116	Non	-0.012509524	0.0000164	0.0000253	Non	ZSCAN20	0
chr1	34098048	34099342	1294	cUMR	-0.266466667	0.00000000000011	0.0000000000019	Hypomethylated	-0.141766667	0.00000182	0.000013	Hypomethylated	0.111033333	0.000474115	0.001221687	Non	-0.190766667	0.0000000205	0.000000592	Hypomethylated	-0.177966667	0.000000082	0.000000685	Hypomethylated	-0.141716667	0.00000808	0.0000252	Hypomethylated	-0.171166667	0.0000000266	0.000000149	Hypomethylated	-0.135138095	0.00000000000151	0.000000000011	Hypomethylated	CSMD2	0
chr1	34100494	34101093	599	cUMR	-0.236266667	0.000000000323	0.00000000225	Hypomethylated	-0.131866667	0.000484518	0.001707933	Non	0.1799	0.00000768	0.0000324	Hypermethylated	-0.190666667	0.000000977	0.0000136	Hypomethylated	-0.168766667	0.000000527	0.00000334	Hypomethylated	-0.202516667	0.00000115	0.0000044	Hypomethylated	-0.027266667	0.000000528	0.00000214	Non	-0.102066667	0.000000000187	0.000000000774	Hypomethylated	CSMD2	0
chr1	34201047	34201445	398	cUMR	-0.305833333	0.0000000000000157	0.000000000000339	Hypomethylated	-0.2007	0.000000117	0.00000121	Hypomethylated	0.0665	0.107298663	0.142679322	Non	-0.2249	0.000000000971	0.0000000564	Hypomethylated	-0.18225	0.00000575	0.0000263	Hypomethylated	-0.24725	0.000000000298	0.00000000257	Hypomethylated	-0.2005	0.00000000173	0.0000000136	Hypomethylated	-0.179557143	0.0000000000000669	0.000000000000736	Hypomethylated	CSMD2	0
chr1	34522981	34523850	869	cUMR	-0.1469	0.00000218	0.00000584	Hypomethylated	0.077233333	0.021768057	0.043190393	Non	-0.0994	0.001162439	0.00268653	Non	-0.140966667	0.000431374	0.001988001	Non	0.050183333	0.012439583	0.021487282	Non	-0.127816667	0.003175241	0.005323659	Non	0.081033333	0.020161108	0.029121332	Non	-0.04862381	0.00000296	0.00000511	Non	CSMD2	0
chr1	34628414	34633265	4851	Canyon	0.043333333	0.0000205	0.000044	Non	0.174533333	0.0000000000159	0.000000000789	Hypermethylated	-0.028166667	0.0000565	0.000189559	Non	0.004733333	0.822518111	0.856398324	Non	0.120083333	0.0000000374	0.000000353	Hypermethylated	0.455583333	0.00000000000000022	0.0000000000000155	Hypermethylated	0.276133333	0.00000000000000833	0.000000000000478	Hypermethylated	0.133447619	0.00000000000000111	0.0000000000000219	Hypermethylated	C1orf94	0
chr1	34730731	34732680	1949	cUMR	-0.244033333	0.0000000000099	0.000000000104	Hypomethylated	-0.098033333	0.007019291	0.016717123	Non	-0.004366667	0.258008041	0.308863013	Non	-0.144033333	0.000028	0.000213869	Hypomethylated	-0.174283333	0.00000374	0.0000181	Hypomethylated	-0.005783333	0.075658278	0.093704744	Non	-0.128633333	0.0000107	0.0000316	Hypomethylated	-0.116119048	0.00000000102	0.00000000356	Hypomethylated	C1orf94	-46001
chr1	34945480	34945577	97	cUMR	-0.069005285	0.000174547	0.000307765	Non	0.051827969	0.434762432	0.505255381	Non	-0.023715282	0.109890081	0.145701853	Non	0.08882864	0.100528767	0.160402427	Non	0.006477247	0.056046023	0.080037872	Non	0.359428992	0.00000674	0.0000214	Hypermethylated	0.095027114	0.090196264	0.113527433	Non	0.059595136	0.000158858	0.000213708	Non	C1orf94	-260750
chr1	34964413	34966512	2099	cUMR	-0.2704	0.000000000366	0.00000000252	Hypomethylated	-0.177966667	0.0000213	0.00011186	Hypomethylated	0.051433333	0.0000031	0.0000147	Non	-0.148966667	0.00013428	0.000761918	Hypomethylated	-0.269066667	0.000000028	0.000000277	Hypomethylated	-0.068066667	0.06217603	0.078328561	Non	-0.309966667	0.0000000000122	0.000000000195	Hypomethylated	-0.167052381	0.0000000000219	0.000000000114	Hypomethylated	GJB5	254136
chr1	35208066	35208747	681	cUMR	-0.0361	0.000804758	0.00124915	Non	-0.023566667	0.08973269	0.139439519	Non	-0.0091	0.471529824	0.520688477	Non	-0.012966667	0.284539274	0.369096546	Non	-0.030016667	0.05757709	0.081998269	Non	-0.091516667	0.00000000273	0.0000000187	Non	-0.018366667	0.323140019	0.360445326	Non	-0.029480952	0.0000222	0.0000336	Non	GJB5	11901
chr1	35227928	35229777	1849	cUMR	-0.4767	0.000000000000717	0.0000000000099	Hypomethylated	-0.115733333	0.039213147	0.070222889	Non	-0.0752	0.0000129	0.000051	Non	-0.246533333	0.0000268	0.000206358	Hypomethylated	-0.368033333	0.000000155	0.00000119	Hypomethylated	-0.284783333	0.0000196	0.0000556	Hypomethylated	-0.295133333	0.00000254	0.00000866	Hypomethylated	-0.263133333	0.0000000000242	0.000000000125	Hypomethylated	GJB4	0
chr1	35246227	35248226	1999	cUMR	-0.0697	0.00000028	0.000000928	Non	0.033266667	0.221756569	0.292366462	Non	0.057966667	0.018219421	0.030188843	Non	-0.047733333	0.001739466	0.006135486	Non	-0.039783333	0.000219716	0.000630399	Non	-0.050033333	0.004874671	0.007832889	Non	-0.009733333	0.798241384	0.816159222	Non	-0.015733333	0.0000942	0.000130267	Non	GJB3	0
chr1	35251908	35252589	681	cUMR	-0.0554	0.000393369	0.000647103	Non	-0.0731	0.002509029	0.006992837	Non	-0.0739	0.000492326	0.001262405	Non	-0.0363	0.010475158	0.025826719	Non	-0.0904	0.000784508	0.001916377	Non	-0.1524	0.00000253	0.00000894	Hypomethylated	-0.0323	0.199257318	0.232553731	Non	-0.070157143	0.000000456	0.000000909	Non	GJB3	0
chr1	35258188	35258887	699	cUMR	0.065033333	0.002262823	0.003243295	Non	0.316933333	0.0000000141	0.000000199	Hypermethylated	0.3062	0.0000000007	0.00000000877	Hypermethylated	-0.015066667	0.704900632	0.75614046	Non	0.214533333	0.000000486	0.00000312	Hypermethylated	0.252533333	0.000000793	0.00000315	Hypermethylated	0.059933333	0.000183698	0.000410041	Non	0.168704762	0.000000000236	0.000000000956	Hypermethylated	GJA4	0
chr1	35318367	35318616	249	cUMR	-0.0607	0.040514971	0.04747531	Non	0.055366667	0.361359348	0.433843666	Non	-0.043340002	0.002676728	0.005601719	Non	-0.030633333	0.128060711	0.194443137	Non	0.195466667	0.000485352	0.001258853	Non	-0.053783333	0.069868843	0.087129962	Non	0.011387843	0.000571731	0.001148706	Non	0.003516045	0.001065521	0.001299768	Non	SMIM12	0
chr1	35323983	35325832	1849	cUMR	-0.024566667	0.000407395	0.00066776	Non	-0.011233333	0.237307825	0.30861928	Non	-0.001066667	0.033092254	0.050945961	Non	-0.011233333	0.135063029	0.20292717	Non	-0.032483333	0.00000168	0.00000915	Non	-0.041983333	0.0000000805	0.000000402	Non	-0.016833333	0.0117572	0.017867148	Non	-0.018519048	0.00000113	0.0000021	Non	SMIM12	0
chr1	35331263	35332512	1249	cUMR	0.0045	0.000156183	0.000278257	Non	-0.009433333	0.287097636	0.3602644	Non	0.425166667	0.00000000000000022	0.0000000000000218	Hypermethylated	0.015166667	0.000250165	0.001268803	Non	-0.020083333	0.035444017	0.053715484	Non	0.007166667	0.001371323	0.002500844	Non	0.065366667	0.000000195	0.000000881	Non	0.082338095	0.000000000000000222	0.00000000000000546	Non	DLGAP3	0
chr1	35350541	35351940	1399	cUMR	-0.057466667	0.023698839	0.028753128	Non	0.4577	0.0000000000371	0.00000000151	Hypermethylated	0.491533333	0.0000000000011	0.0000000000299	Hypermethylated	0.0811	0.005860055	0.016214903	Non	0.11045	0.0000422	0.000149099	Hypermethylated	0.4567	0.000000000334	0.00000000285	Hypermethylated	0.3339	0.000000011	0.0000000688	Hypermethylated	0.2639	0.0000000000042	0.000000000027	Hypermethylated	DLGAP3	0
chr1	35384117	35385177	1060	cUMR	-0.0625	0.009336651	0.012055005	Non	0.094833333	0.014098512	0.030038182	Non	0.2985	0.000000000243	0.00000000345	Hypermethylated	-0.025366667	0.632093071	0.693348756	Non	0.011583333	0.015790651	0.026563567	Non	0.016083333	0.606889809	0.629702072	Non	0.153233333	0.0000216	0.0000596	Hypermethylated	0.075433333	0.000000141	0.000000308	Non	DLGAP3	-13134
chr1	35390174	35391373	1199	cUMR	0.001466667	0.002172572	0.003123337	Non	0.0356	0.002511559	0.006998268	Non	0.227633333	0.0000000000000131	0.000000000000675	Hypermethylated	-0.029	0.001701229	0.006022657	Non	-0.0172	0.532928492	0.578465421	Non	0.0918	0.000000392	0.00000168	Non	0.0438	0.025807102	0.036465603	Non	0.055	0.0000000000351	0.000000000173	Non	DLGAP3	-19191
chr1	35393624	35396535	2911	cUMR	0.012466667	0.024995765	0.030204992	Non	0.0971	0.000000566	0.00000472	Non	-0.016866667	0.116406834	0.153422221	Non	0.0205	0.092159152	0.149622802	Non	0.0518	0.000250104	0.000704832	Non	0.35105	0.000000000000000444	0.0000000000000277	Hypermethylated	0.1217	0.0000000213	0.000000124	Hypermethylated	0.079471429	0.00000000000259	0.0000000000176	Non	DLGAP3	-22641
chr1	35449414	35451222	1808	cUMR	-0.028	0.009758095	0.012562804	Non	-0.005633333	0.306691061	0.379672977	Non	-0.0025	0.216226657	0.264610228	Non	-0.024433333	0.054135843	0.097903606	Non	0.013916667	0.213919727	0.259268601	Non	-0.019333333	0.047115908	0.060944668	Non	0.021766667	0.131323459	0.159582938	Non	-0.007033333	0.269765499	0.275108294	Non	ZMYM6NB	0
chr1	35496068	35497817	1749	cUMR	-0.014	0.0000263	0.0000551	Non	0.0092	0.267984214	0.340735311	Non	0.026333333	0.000170556	0.000500006	Non	-0.0108	0.017870781	0.039904385	Non	0.005	0.00000613	0.0000278	Non	-0.02275	0.000056	0.000142257	Non	-0.001	0.007382358	0.011735138	Non	-0.000285714	0.000000755	0.00000144	Non	ZMYM6	0
chr1	35544272	35545771	1499	cUMR	-0.056833333	0.000000579	0.00000178	Non	-0.009733333	0.35890448	0.431390725	Non	0.001833333	0.330754881	0.383290749	Non	-0.037533333	0.023337029	0.049561623	Non	-0.012833333	0.254807599	0.302316652	Non	-0.049333333	0.000149023	0.000341366	Non	-0.019933333	0.324627336	0.361888517	Non	-0.026133333	0.0000257	0.0000386	Non	ZMYM1	0
chr1	35586185	35586884	699	cUMR	0.012933333	0.00000000497	0.0000000255	Non	0.302333333	0.000000000168	0.00000000515	Hypermethylated	0.3791	0.0000000000000693	0.00000000000282	Hypermethylated	0.013133333	0.00000108	0.0000149	Non	0.269683333	0.00000000000501	0.000000000265	Hypermethylated	0.141683333	0.00000000139	0.0000000103	Hypermethylated	0.208733333	0.00000000000775	0.000000000133	Hypermethylated	0.189104762	0.000000000000002	0.0000000000000361	Hypermethylated	ZMYM1	-4727
chr1	35654953	35659702	4749	Canyon	-0.0486	0.000000391	0.00000125	Non	-0.037833333	0.000110441	0.000474048	Non	-0.026433333	0.024832184	0.039634784	Non	-0.023233333	0.021247846	0.045944912	Non	-0.053433333	0.00000648	0.0000292	Non	-0.026433333	0.034540147	0.04602679	Non	-0.042633333	0.000192967	0.000428744	Non	-0.036804762	0.0000000215	0.0000000558	Non	SFPQ	0
chr1	35667645	35668230	585	cUMR	-0.0387	0.143463078	0.156574876	Non	0.0076	0.08606739	0.134813176	Non	-0.163866667	0.00000257	0.0000124	Hypomethylated	-0.0014	0.246165501	0.328848398	Non	0.0903	0.09595796	0.128330925	Non	0.08505	0.215069267	0.243252567	Non	0.0142	0.263079231	0.299002743	Non	-0.011771429	0.156590387	0.16135787	Non	SFPQ	-8903
chr1	35734123	35735577	1454	cUMR	-0.011566667	0.009037105	0.011693307	Non	-0.0034	0.396602478	0.468660745	Non	0.005433333	0.664028324	0.701646696	Non	-0.013373067	0.002398552	0.007933064	Non	-0.00565	0.3975944	0.447198593	Non	-0.0099	0.139924722	0.164158461	Non	-0.0116	0.022136502	0.031716087	Non	-0.006882523	0.012781125	0.014130365	Non	ZMYM4	0
chr1	36020933	36024432	3499	cUMR	-0.029133333	0.000763519	0.001189968	Non	-0.009933333	0.378628435	0.450959341	Non	-0.009966667	0.137450061	0.17759217	Non	-0.018133333	0.089796194	0.146477268	Non	-0.029133333	0.002950253	0.006085875	Non	-0.019883333	0.082519733	0.101431294	Non	-0.018133333	0.000985015	0.001877763	Non	-0.018904762	0.0000624	0.0000884	Non	NCDN	0
chr1	36105569	36108518	2949	cUMR	-0.070366667	0.000000452	0.00000142	Non	-0.0536	0.001283748	0.003933183	Non	0.0148	0.087782046	0.11960037	Non	-0.046	0.00380179	0.011449079	Non	-0.05595	0.0000713	0.000234945	Non	-0.0752	0.0000212	0.0000595	Non	-0.051	0.000318282	0.000673931	Non	-0.046028571	0.0000000479	0.000000116	Non	PSMB2	0
chr1	36111598	36112364	766	cUMR	-0.143245939	0.000000000000686	0.0000000000095	Hypomethylated	-0.0714	0.021159997	0.042220125	Non	-0.1046	0.000000516	0.00000299	Hypomethylated	-0.0716	0.0000978	0.000588058	Non	-0.01335	0.001494231	0.00334771	Non	-0.15735	0.00000201	0.00000726	Hypomethylated	-0.1244	0.00000764	0.0000234	Hypomethylated	-0.100195139	0.0000000000197	0.000000000105	Hypomethylated	PSMB2	-4154
chr1	36172771	36174450	1679	cUMR	-0.042766667	0.002593554	0.003678674	Non	0.111866667	0.0000307	0.000154234	Hypermethylated	0.1649	0.0000000694	0.000000517	Hypermethylated	-0.001733333	0.776291049	0.817330553	Non	0.000316667	0.082667606	0.112651208	Non	0.090316667	0.000154879	0.000352799	Non	0.138866667	0.00000000488	0.0000000335	Hypermethylated	0.066866667	0.00000000927	0.000000026	Non	C1orf216	5027
chr1	36181146	36181447	301	cUMR	-0.066533333	0.040637333	0.047614064	Non	-0.037633333	0.133324983	0.192379168	Non	-0.0012	0.991206997	0.992295995	Non	-0.032233333	0.031536273	0.063156412	Non	0.014966667	0.647820809	0.685904476	Non	-0.081783333	0.016209998	0.023171701	Non	0.084366667	0.041344211	0.055996297	Non	-0.01717619	0.11807416	0.122512427	Non	C1orf216	0
chr1	36183263	36184978	1715	cUMR	-0.019566667	0.020090701	0.024628431	Non	-0.0144	0.045323649	0.07925143	Non	0.013433333	0.014076648	0.02401721	Non	-0.0078	0.034734588	0.068140191	Non	-0.0114	0.046626762	0.068172049	Non	0.03585	0.000608034	0.001204077	Non	0.0074	0.392544839	0.42900764	Non	-0.000371429	0.020196554	0.021991621	Non	C1orf216	0
chr1	36234113	36236212	2099	cUMR	-0.056433333	0.000000151	0.000000533	Non	-0.019933333	0.001913782	0.005547762	Non	-0.0141	0.009463884	0.016959967	Non	-0.012333333	0.545368209	0.617066537	Non	-0.050683333	0.0000189	0.0000744	Non	-0.053433333	0.0000042	0.000014	Non	-0.040933333	0.00061286	0.001222776	Non	-0.034447619	0.000000207	0.000000439	Non	CLSPN	0
chr1	36240396	36240745	349	cUMR	-0.0952	0.000111615	0.000204586	Non	-0.000966667	0.241916494	0.313378225	Non	-0.1302	0.00000109	0.00000576	Hypomethylated	-0.073166667	0.012953836	0.030673239	Non	-0.007366667	0.044266692	0.065188142	Non	-0.069616667	0.00379583	0.00626134	Non	-0.006566667	0.000000811	0.00000314	Non	-0.058966667	0.000000366	0.000000741	Non	CLSPN	-4846
chr1	36273077	36274460	1383	cUMR	-0.022333333	0.0000285	0.0000592	Non	-0.0088	0.156931504	0.219859212	Non	-0.000166667	0.168082233	0.212054115	Non	-0.0108	0.173829175	0.24840986	Non	0.0075	0.111766758	0.146848881	Non	0.026	0.01462384	0.021090748	Non	-0.0134	0.057663792	0.075745025	Non	-0.004742857	0.003721589	0.004304432	Non	AGO4	0
chr1	36348337	36349936	1599	cUMR	-0.001766667	0.003676372	0.005082509	Non	0.003633333	0.652687771	0.704178071	Non	0.010233333	0.15943659	0.202674629	Non	0.001433333	0.437954582	0.518310063	Non	-0.005266667	0.810606776	0.833824309	Non	0.019233333	0.15342527	0.178584851	Non	0.012833333	0.064073406	0.083319633	Non	0.005604762	0.014485314	0.015958082	Non	AGO1	0
chr1	36395082	36397981	2899	cUMR	-0.007933333	0.032974695	0.039134411	Non	-0.003633333	0.496509319	0.563135887	Non	-0.007933333	0.012863158	0.022190306	Non	-0.000833333	0.923965527	0.939602254	Non	-0.005683333	0.002595984	0.005443142	Non	-0.009683333	0.010587889	0.015770254	Non	-0.003833333	0.525122273	0.557932285	Non	-0.005661905	0.005776752	0.006573555	Non	AGO3	0
chr1	36549244	36550543	1299	cUMR	-0.070966667	0.0000164	0.0000361	Non	0.119766667	0.0000323	0.000161314	Hypermethylated	-0.044966667	0.003496696	0.007072636	Non	-0.007033333	0.774624927	0.815897176	Non	0.112366667	0.000000000695	0.0000000132	Hypermethylated	0.185866667	0.00000045	0.0000019	Hypermethylated	0.051166667	0.00000633	0.0000197	Non	0.03762381	0.000000000993	0.00000000348	Non	TEKT2	0
chr1	36553956	36555672	1716	cUMR	-0.017166667	0.0000198	0.0000426	Non	-0.002466667	0.182959195	0.249449345	Non	0.0245	0.0000917	0.000289596	Non	-0.009266667	0.048154601	0.089009678	Non	-0.023416667	0.0000000508	0.000000458	Non	-0.009416667	0.076986085	0.095266031	Non	0.001333333	0.00162031	0.002944641	Non	-0.003980952	0.00000377	0.0000064	Non	ADPRHL2	0
chr1	36590350	36591449	1099	cUMR	-0.041333333	0.000212212	0.000367847	Non	0.120666667	0.000126341	0.000532246	Hypermethylated	0.068666667	0.004411508	0.008659161	Non	0.018466667	0.330124823	0.41537949	Non	0.031166667	0.0176073	0.029185633	Non	0.106416667	0.000884741	0.001686213	Non	0.126866667	0.0000000325	0.000000178	Hypermethylated	0.058409524	0.000000188	0.000000402	Non	COL8A2	0
chr1	36614088	36616845	2757	cUMR	-0.040533333	0.005712058	0.007645478	Non	-0.0111	0.438844187	0.509115034	Non	-0.019533333	0.341776808	0.394469923	Non	-0.0167	0.118790234	0.183312439	Non	-0.02295	0.349748454	0.399404713	Non	-0.0147	0.246671804	0.275530598	Non	-0.0141	0.042593935	0.057536913	Non	-0.020585714	0.017995231	0.019679119	Non	TRAPPC3	0
chr1	36620776	36623375	2599	cUMR	-0.009373821	0.00422532	0.005786314	Non	0.001466667	0.217685428	0.288043807	Non	-0.008733333	0.002488428	0.005249596	Non	-0.000733333	0.111433827	0.174315636	Non	-0.001483333	0.848938768	0.8677472	Non	-0.006692253	0.10708819	0.128736152	Non	0.001266667	0.961136714	0.965101899	Non	-0.003754123	0.096411383	0.100533168	Non	TRAPPC3	0
chr1	36653591	36653940	349	cUMR	-0.02228534	0.00244602	0.003487616	Non	-0.003466667	0.946619412	0.956503186	Non	0.011333333	0.80814738	0.832075863	Non	-0.017466667	0.594474938	0.660320675	Non	0.033630983	0.015013368	0.025403184	Non	-0.027378868	0.03709009	0.049109724	Non	0.181333333	0.00000222	0.00000769	Hypermethylated	0.0217516	0.000099	0.000136649	Non	MAP7D1	-7141
chr1	36688756	36690683	1927	cUMR	-0.010766667	0.037119343	0.04371741	Non	-0.002666667	0.637622633	0.690738743	Non	0.011233333	0.130447827	0.16959977	Non	-0.018466667	0.0000396	0.000284095	Non	-0.009516667	0.108248353	0.142786623	Non	-0.010516667	0.018124837	0.025599128	Non	-0.001266667	0.431207444	0.46696214	Non	-0.005409524	0.000645773	0.000806021	Non	THRAP3	0
chr1	36771575	36773218	1643	cUMR	-0.046133333	0.0000000634	0.000000246	Non	-0.0068	0.443924522	0.513890778	Non	0.235866667	0.000000000000000555	0.0000000000000485	Hypermethylated	-0.0394	0.000000307	0.00000536	Non	-0.0408	0.0000425	0.000150151	Non	-0.03155	0.002436613	0.004205824	Non	0.0418	0.00000192	0.00000676	Non	0.023628571	0.000000000000875	0.00000000000683	Non	SH3D21	0
chr1	36787911	36790010	2099	cUMR	0.023233333	0.830777649	0.836086021	Non	0.1073	0.123663654	0.180952336	Non	0.185233333	0.002090547	0.004503845	Non	0.0759	0.028330799	0.057945645	Non	0.0354	0.686276198	0.7217056	Non	0.0874	0.049161339	0.06330501	Non	0.1443	0.027747259	0.038946697	Non	0.096557143	0.003538584	0.004100639	Non	EVA1B	0
chr1	36849882	36852931	3049	cUMR	-0.015	0.004134194	0.005668287	Non	-0.014333333	0.011372493	0.025100107	Non	-0.002666667	0.706197865	0.739782479	Non	-0.003733333	0.757070072	0.801329758	Non	-0.008333333	0.311600461	0.360850973	Non	-0.029083333	0.0000000931	0.000000458	Non	-0.005133333	0.53764452	0.570105719	Non	-0.010619048	0.000144544	0.000195577	Non	STK40	0
chr1	36862621	36863770	1149	cUMR	-0.012267261	0.002057497	0.002970019	Non	-0.000666667	0.69286671	0.740136941	Non	-0.0118	0.006675394	0.012457496	Non	-0.003866667	0.152236435	0.223531173	Non	-0.006716667	0.383966155	0.433529326	Non	-0.006216667	0.347319687	0.37749007	Non	-0.008466667	0.058256029	0.076410499	Non	-0.007460985	0.005278383	0.006025223	Non	LSM10	0
chr1	36886056	36886955	899	cUMR	-0.0763	0.009879907	0.012706052	Non	-0.046566667	0.367060161	0.439549207	Non	-0.119133333	0.000867522	0.002075336	Non	0.001033333	0.437545397	0.517952988	Non	-0.057466667	0.136249431	0.17452006	Non	-0.012716667	0.87343434	0.882220669	Non	0.065433333	0.264809889	0.300705081	Non	-0.038680952	0.037207747	0.039784899	Non	OSCP1	0
chr1	36915402	36916851	1449	cUMR	-0.042466667	0.003738952	0.005161918	Non	-0.035866667	0.043758155	0.07689209	Non	-0.025633333	0.102003719	0.136446607	Non	-0.010266667	0.798918378	0.836727413	Non	0.012783333	0.406191634	0.456102045	Non	-0.000716667	0.82356849	0.835354553	Non	0.069933333	0.000000109	0.000000525	Non	-0.006895238	0.0000678	0.0000955	Non	OSCP1	0
chr1	36928907	36930606	1699	cUMR	-0.002666667	0.891583604	0.895149641	Non	0.0084	0.063768885	0.105473185	Non	0.007166667	0.465827891	0.515452315	Non	-0.0106	0.11397917	0.177513736	Non	0.00475	0.785807914	0.811293394	Non	-0.02	0.018462065	0.026035747	Non	-0.0038	0.708215026	0.731875853	Non	-0.001828571	0.636113874	0.639697539	Non	MRPS15	0
chr1	37019414	37020969	1555	cUMR	-0.2383	0.00000000000000644	0.000000000000155	Hypomethylated	-0.113866667	0.000011	0.0000629	Hypomethylated	-0.116466667	0.000000000857	0.0000000105	Hypomethylated	-0.111066667	0.00000433	0.0000456	Hypomethylated	-0.123716667	0.00000987	0.0000423	Hypomethylated	-0.183966667	0.00000000185	0.0000000132	Hypomethylated	-0.160066667	0.00000000436	0.0000000303	Hypomethylated	-0.150980952	0.00000000000000133	0.0000000000000257	Hypomethylated	CSF3R	-70500
chr1	37037741	37039090	1349	cUMR	-0.313366667	0.00000000141	0.00000000834	Hypomethylated	0.031566667	0.739884985	0.781699775	Non	0.070466667	0.000969687	0.002292732	Non	-0.147433333	0.008228436	0.021268782	Non	-0.160783333	0.0000766	0.000250126	Hypomethylated	-0.140033333	0.011846774	0.017453295	Non	-0.116433333	0.027760562	0.038963101	Non	-0.109204762	0.000000233	0.000000489	Hypomethylated	CSF3R	-88827
chr1	37041506	37042405	899	cUMR	-0.090233333	0.000248409	0.00042446	Non	-0.050666667	0.076483037	0.122296732	Non	-0.034233333	0.248571421	0.298786618	Non	-0.062266667	0.00023523	0.001207622	Non	-0.042316667	0.228450735	0.274601248	Non	-0.079316667	0.007299427	0.011279358	Non	-0.011866667	0.003323532	0.005686965	Non	-0.053066667	0.000206924	0.000274253	Non	CSF3R	-92592
chr1	37115941	37116540	599	cUMR	-0.1407	0.00000529	0.000013	Hypomethylated	-0.0716	0.025585396	0.049449093	Non	-0.029866667	0.000829135	0.001994857	Non	-0.0398	0.199638409	0.277845012	Non	-0.11895	0.001237237	0.002839517	Non	-0.0447	0.37877975	0.408513892	Non	0.0094	0.001575362	0.002872487	Non	-0.062514286	0.00000757	0.0000123	Non	GRIK3	144588
chr1	37169146	37169695	549	cUMR	-0.056833333	0.0000049100000000000004	0.0000121	Non	-0.0058	0.632545057	0.685947448	Non	-0.018666667	0.007697067	0.014113828	Non	-0.0776	0.0000000153	0.000000472	Non	-0.002	0.054798907	0.078487017	Non	0.037	0.034477391	0.045953328	Non	-0.0308	0.00000181	0.0000064	Non	-0.025257143	0.000000115	0.000000256	Non	GRIK3	91433
chr1	37202894	37204093	1199	cUMR	-0.219766667	0.00000293	0.00000761	Hypomethylated	0.050733333	0.42755274	0.498583932	Non	-0.1881	0.0000697	0.000227611	Hypomethylated	-0.208266667	0.0000415	0.000294418	Hypomethylated	-0.119516667	0.0000332	0.000121043	Hypomethylated	-0.015016667	0.001083336	0.002022484	Non	-0.153666667	0.000313192	0.000664086	Hypomethylated	-0.129752381	0.000000159	0.000000345	Hypomethylated	GRIK3	57035
chr1	37389340	37390439	1099	cUMR	-0.053666667	0.054899177	0.06321787	Non	0.048333333	0.086331991	0.135166134	Non	0.120333333	0.0000755	0.000244532	Hypermethylated	-0.065666667	0.004413789	0.01291446	Non	-0.014666667	0.607818514	0.648680715	Non	0.059083333	0.046526504	0.060259899	Non	-0.036866667	0.061352516	0.08008782	Non	0.008761905	0.000533434	0.000672493	Non	GRIK3	0
chr1	37496723	37504722	7999	Canyon	0.075	0.000153908	0.000274527	Non	0.078133333	0.004386398	0.011286601	Non	-0.033833333	0.046209777	0.068241403	Non	0.024933333	0.13863569	0.207337319	Non	0.130083333	0.0000427	0.000150674	Hypermethylated	0.427833333	0.000000000000144	0.00000000000349	Hypermethylated	0.363733333	0.00000000000205	0.0000000000435	Hypermethylated	0.137504762	0.0000000000181	0.0000000000972	Hypermethylated	GRIK3	0
chr1	37609421	37611220	1799	cUMR	-0.2845	0.000000000103	0.00000000083	Hypomethylated	-0.155466667	0.00008	0.000357554	Hypomethylated	0.126666667	0.0000187	0.000071	Hypermethylated	-0.239066667	0.0000000466	0.00000115	Hypomethylated	-0.219916667	0.00000457	0.0000216	Hypomethylated	-0.282916667	0.00000000911	0.0000000557	Hypomethylated	-0.220466667	0.000000349	0.00000148	Hypomethylated	-0.172380952	0.0000000000249	0.000000000128	Hypomethylated	MIR4255	15944
chr1	37720482	37722681	2199	cUMR	-0.1243	0.000000213	0.000000728	Hypomethylated	0.008133333	0.534475629	0.597673174	Non	0.0522	0.00000209	0.0000103	Non	-0.017666667	0.057738121	0.103011272	Non	0.005283333	0.000448057	0.001173892	Non	0.022783333	0.000812127	0.001562276	Non	0.012733333	0.009637384	0.014923145	Non	-0.008695238	0.000000174	0.000000374	Non	MIR4255	-93248
chr1	37755957	37757356	1399	cUMR	-0.434466667	0.000000000000668	0.00000000000927	Hypomethylated	-0.204166667	0.000185663	0.000743052	Hypomethylated	-0.295133333	0.0000000287	0.000000237	Hypomethylated	-0.239966667	0.00000826	0.0000779	Hypomethylated	-0.333466667	0.000000144	0.00000111	Hypomethylated	-0.367716667	0.0000000106	0.000000064	Hypomethylated	-0.299566667	0.000000133	0.000000624	Hypomethylated	-0.311452381	0.000000000000000333	0.0000000000000078	Hypomethylated	MIR4255	-128723
chr1	37783378	37784527	1149	cUMR	-0.101833333	0.00682608	0.009018854	Non	0.305566667	0.00001	0.0000578	Hypermethylated	0.384833333	0.0000000714	0.00000053	Hypermethylated	0.040166667	0.435775823	0.51636553	Non	0.096666667	0.06705189	0.093801024	Non	0.197916667	0.004545799	0.007357916	Non	0.146366667	0.009715324	0.015025702	Non	0.152480952	0.0000056	0.00000926	Hypermethylated	LINC01137	135953
chr1	37789531	37789780	249	cUMR	-0.0698	0.002891537	0.004069871	Non	0.1034	0.002237579	0.006353869	Non	0.2667	0.00000000738	0.0000000724	Hypermethylated	-0.0344	0.361763464	0.446317059	Non	0.07845	0.001581293	0.003520185	Non	-0.02405	0.03337022	0.044618297	Non	0.031	0.00161011	0.002928754	Non	0.054257143	0.00000146	0.00000266	Non	LINC01137	130700
chr1	37828372	37829021	649	cUMR	-0.0748	0.015618648	0.019488334	Non	0.162066667	0.000634243	0.00215228	Non	0.115033333	0.007661452	0.014057078	Non	-0.074533333	0.065872118	0.114439211	Non	0.033116667	0.007994197	0.014565564	Non	0.097616667	0.000148905	0.000341159	Non	0.076466667	0.062407992	0.081333927	Non	0.045266667	0.0000672	0.0000948	Non	LINC01137	91459
chr1	37899646	37900645	999	cUMR	-0.211066667	0.00000000445	0.0000000231	Hypomethylated	-0.046533333	0.070723614	0.114878794	Non	-0.0064	0.909977674	0.921294093	Non	-0.090333333	0.018891704	0.041789269	Non	-0.181233333	0.00000296	0.0000148	Hypomethylated	-0.205233333	0.00000042	0.00000178	Hypomethylated	-0.137333333	0.000157887	0.00035847	Hypomethylated	-0.120619048	0.00000000945	0.0000000264	Hypomethylated	LINC01137	19835
chr1	37939055	37943654	4599	Canyon	-0.115066667	0.000302529	0.000507507	Hypomethylated	-0.077533333	0.062962537	0.104397458	Non	0.139433333	0.000117851	0.00036031	Hypermethylated	-0.079133333	0.061823169	0.108769418	Non	-0.136233333	0.000147189	0.000442541	Hypomethylated	-0.141233333	0.00000914	0.0000281	Hypomethylated	-0.045733333	0.44570552	0.481191819	Non	-0.056447619	0.000391856	0.000502045	Non	LINC01137	0
chr1	37952978	37953159	181	cUMR	-0.000999137	0.102302542	0.113922674	Non	-0.014456591	0.488177346	0.555391763	Non	-0.017213099	0.09297658	0.125817027	Non	0.001979005	0.098170462	0.157315586	Non	0.064118883	0.153952754	0.194025075	Non	-0.038777771	0.00485318	0.007801477	Non	0.117719628	0.003814279	0.006447935	Non	0.01481228	0.007025676	0.007935168	Non	MEAF6	2402
chr1	37979017	37981066	2049	cUMR	-0.022566667	0.000663016	0.001045844	Non	-0.020866667	0.009738742	0.022096803	Non	-0.002733333	0.258921147	0.309755863	Non	-0.021266667	0.007791186	0.020352521	Non	-0.013316667	0.235429607	0.281893206	Non	-0.010566667	0.079325158	0.097869018	Non	-0.002066667	0.945700812	0.951227532	Non	-0.013380952	0.001150822	0.001399078	Non	MEAF6	0
chr1	38018764	38020113	1349	cUMR	-0.022633333	0.000000000227	0.00000000165	Non	-0.009066667	0.017343655	0.035740797	Non	-0.000133333	0.0000284	0.000103217	Non	-0.004466667	0.276398941	0.360561991	Non	-0.016966667	0.00000316	0.0000156	Non	-0.016966667	0.000033	0.0000885	Non	-0.021866667	0.00000000726	0.0000000475	Non	-0.012838095	0.000000000132	0.000000000567	Non	SNIP1	0
chr1	38060233	38062332	2099	cUMR	-0.072766667	0.000000000000741	0.0000000000102	Non	-0.0207	0.076757175	0.122658487	Non	-0.038933333	0.000000000938	0.0000000114	Non	-0.0157	0.193316014	0.270718812	Non	-0.0566	0.00000000117	0.0000000203	Non	-0.0831	0.000000000106	0.00000000103	Non	-0.0645	0.0000000034	0.0000000243	Non	-0.049528571	0.0000000000000364	0.000000000000433	Non	GNL2	0
chr1	38067859	38069408	1549	cUMR	-0.272466667	0.000000000101	0.000000000816	Hypomethylated	-0.100133333	0.011422547	0.025196733	Non	-0.185436318	0.000000000000155	0.0000000000056	Hypomethylated	-0.108933333	0.010003978	0.024931154	Non	-0.167133333	0.000127808	0.000391337	Hypomethylated	-0.123133333	0.003980258	0.006532219	Non	-0.155533333	0.0000153	0.0000437	Hypomethylated	-0.163758203	0.00000000000251	0.0000000000171	Hypomethylated	GNL2	-6274
chr1	38097542	38102191	4649	Canyon	-0.013466667	0.481530719	0.495217107	Non	0.069766667	0.00033609	0.001246347	Non	-0.003966667	0.00038299	0.001012428	Non	-0.003833333	0.615110974	0.677868214	Non	0.072116667	0.00000377	0.0000182	Non	0.194616667	0.000000000699	0.0000000055	Hypermethylated	0.135566667	0.0000000208	0.000000121	Hypermethylated	0.056280952	0.00000000478	0.0000000143	Non	RSPO1	0
chr1	38155124	38158923	3799	Canyon	-0.0409	0.00000000159	0.00000000927	Non	-0.032366667	0.00000465	0.0000294	Non	-0.0059	0.000104453	0.000324286	Non	-0.020766667	0.007788886	0.020348719	Non	-0.034816667	0.00000819	0.0000358	Non	-0.038316667	0.000000565	0.00000233	Non	-0.027366667	0.00000000931	0.0000000592	Non	-0.027880952	0.000000000025	0.000000000129	Non	C1orf109	0
chr1	38200625	38201474	849	cUMR	0.115533333	0.001285881	0.001926344	Non	0.465133333	0.000000000125	0.00000000404	Hypermethylated	0.3802	0.00000000321	0.0000000346	Hypermethylated	0.164333333	0.000482491	0.002178984	Non	0.097783333	0.000505948	0.001308331	Non	0.076033333	0.26727795	0.296759309	Non	0.291333333	0.00000151	0.00000545	Hypermethylated	0.236390476	0.000000000508	0.00000000191	Hypermethylated	EPHA10	0
chr1	38217705	38220254	2549	cUMR	-0.026866667	0.000000216	0.000000734	Non	0.082666667	0.0000000259	0.000000331	Non	0.183466667	0.000000000000000555	0.0000000000000485	Hypermethylated	-0.015133333	0.003846239	0.011555501	Non	0.053716667	0.00000000644	0.0000000823	Non	0.098716667	0.00000000628	0.0000000397	Non	0.160466667	0.0000000000000271	0.00000000000126	Hypermethylated	0.076838095	0.000000000000000777	0.0000000000000163	Non	EPHA10	0
chr1	38229735	38233188	3453	cUMR	0.046733333	0.016015199	0.019952141	Non	0.151233333	0.00000186	0.0000132	Hypermethylated	0.0369	0.16066393	0.203968633	Non	0.048833333	0.085716068	0.141236637	Non	0.134233333	0.000103639	0.000326171	Hypermethylated	0.235233333	0.000000043	0.000000228	Hypermethylated	0.215033333	0.0000000388	0.000000209	Hypermethylated	0.115861905	0.00000000563	0.0000000165	Hypermethylated	EPHA10	0
chr1	38258961	38261410	2449	cUMR	-0.042866667	0.002077004	0.002995132	Non	0.0288	0.219125411	0.289551713	Non	0.0238	0.215798094	0.264149056	Non	-0.0112	0.004511635	0.013154432	Non	0.0258	0.094611708	0.126804947	Non	0.0368	0.170307348	0.196404406	Non	0.0604	0.014171733	0.021224569	Non	0.015028571	0.017915075	0.019592352	Non	MANEAL	0
chr1	38272785	38275138	2353	cUMR	-0.0115	0.004663591	0.006335732	Non	0.000633333	0.105435828	0.158892254	Non	-0.022	0.0005626	0.001418596	Non	-0.004366667	0.466578804	0.545797559	Non	-0.012916667	0.023459823	0.037461719	Non	-0.014416667	0.108109456	0.129884419	Non	-0.003566667	0.847372944	0.860805163	Non	-0.009909524	0.010102598	0.011262081	Non	C1orf122	0
chr1	38323905	38326158	2253	cUMR	-0.015266667	0.003306535	0.004609028	Non	0.000266667	0.904342546	0.92174699	Non	-0.0221	0.0000441	0.000151734	Non	-0.002533333	0.160166299	0.232763688	Non	-0.020183333	0.002473111	0.005208619	Non	-0.014433333	0.045187039	0.058689084	Non	-0.009133333	0.244980189	0.280496205	Non	-0.011990476	0.000170105	0.000228151	Non	MTF1	0
chr1	38396216	38398715	2499	cUMR	-0.144233333	0.0000000000000119	0.000000000000263	Hypomethylated	-0.066733333	0.0000543	0.000254585	Non	0.0226	0.283442964	0.334938878	Non	-0.076133333	0.00000332	0.0000372	Non	-0.134733333	0.0000000000128	0.000000000547	Hypomethylated	-0.090733333	0.00000123	0.00000467	Non	-0.116933333	0.00000000000718	0.000000000125	Hypomethylated	-0.083733333	0.0000000000000774	0.000000000000831	Non	INPP5B	0
chr1	38454473	38456565	2092	cUMR	-0.050266667	0.000286898	0.000483338	Non	-0.048766667	0.001355043	0.004117589	Non	-0.013933333	0.340689949	0.393441626	Non	-0.033366667	0.062014675	0.109042659	Non	-0.059516667	0.000521833	0.001345223	Non	-0.037516667	0.037053939	0.049069947	Non	-0.054966667	0.000602362	0.001204224	Non	-0.041680952	0.00000239	0.00000418	Non	SF3A3	0
chr1	38469741	38471627	1886	cUMR	-0.027533333	0.027111778	0.032590393	Non	0.0165	0.021436781	0.042645266	Non	0.0398	0.060224612	0.086124015	Non	0.0109	0.134577103	0.202415666	Non	0.02305	0.36752827	0.417034732	Non	0.09805	0.000294108	0.000627371	Non	0.0219	0.096653621	0.120853389	Non	0.022985714	0.026685899	0.028771724	Non	FHL3	0
chr1	38477901	38479250	1349	cUMR	-0.024241437	0.00000000000149	0.0000000000191	Non	-0.0066	0.486098492	0.553542619	Non	0.0049	0.013791488	0.023600858	Non	-0.0078	0.00000434	0.0000457	Non	-0.00685	0.105433775	0.139470995	Non	0.0009	0.309066582	0.339172389	Non	0.022	0.000821587	0.001592933	Non	-0.002910986	0.00000000133	0.0000000045	Non	UTP11L	0
chr1	38509869	38513863	3994	Canyon	0.015766667	0.000828493	0.001283594	Non	0.136033333	0.0000000205	0.000000272	Hypermethylated	0.0361	0.001799311	0.003949699	Non	0.024433333	0.046205629	0.086021574	Non	0.051683333	0.000116226	0.000360048	Non	0.362683333	0.0000000000000111	0.000000000000402	Hypermethylated	0.191833333	0.000000000399	0.00000000384	Hypermethylated	0.10657619	0.00000000000199	0.000000000014	Hypermethylated	POU3F1	0
chr1	38605544	38605993	449	cUMR	0.051266667	0.09884168	0.11022661	Non	0.188066667	0.000971703	0.003107928	Non	-0.051733333	0.350212007	0.402835749	Non	0.003466667	0.620595976	0.682788743	Non	0.229766667	0.003297068	0.006702045	Non	0.051766667	0.323138325	0.353263502	Non	0.041866667	0.244740918	0.280275518	Non	0.065438095	0.0088015	0.009857713	Non	MIR3659	-50544
chr1	38941647	38942703	1056	cUMR	-0.0203	0.522496991	0.535200735	Non	0.359666667	0.000000236	0.0000022	Hypermethylated	-0.0553	0.175662894	0.220291654	Non	-0.076533333	0.000539434	0.002383878	Non	0.151616667	0.000327714	0.000893454	Hypermethylated	0.229616667	0.000246015	0.00053424	Hypermethylated	0.101466667	0.028266339	0.039592263	Non	0.085552381	0.0000245	0.0000368	Non	LINC01343	-261209
chr1	39043151	39044837	1686	cUMR	0.029	0.027808837	0.03338834	Non	0.239466667	0.00000000000552	0.00000000036	Hypermethylated	0.010333333	0.678567268	0.715065332	Non	-0.038933333	0.001908682	0.006603792	Non	0.189666667	0.000000000000929	0.0000000000727	Hypermethylated	0.263916667	0.000000000000889	0.0000000000167	Hypermethylated	0.256866667	0.000000000000824	0.0000000000202	Hypermethylated	0.12392381	0.0000000000000222	0.000000000000284	Hypermethylated	RRAGC	259032
chr1	39269150	39270549	1399	cUMR	0.069033333	0.000000000000049	0.000000000000928	Non	0.034166667	0.030351029	0.057035697	Non	0.039366667	0.00434245	0.008538911	Non	0.001166667	0.000813125	0.00331159	Non	0.022866667	0.198620871	0.24302441	Non	0.191866667	0.0000000000188	0.000000000228	Hypermethylated	0.056966667	0.000000000735	0.00000000647	Non	0.056309524	0.000000000000898	0.00000000000698	Non	RRAGC	33320
chr1	39281566	39282265	699	cUMR	-0.152166667	0.000340317	0.000566248	Hypomethylated	0.295633333	0.00000992	0.0000573	Hypermethylated	0.393833333	0.000000114	0.000000789	Hypermethylated	-0.013366667	0.121230552	0.186124735	Non	0.167833333	0.00000686	0.0000307	Hypermethylated	0.301083333	0.0000931	0.000224163	Hypermethylated	0.078433333	0.000813998	0.001579617	Non	0.146547619	0.0000000541	0.000000129	Hypermethylated	RRAGC	21604
chr1	39283610	39284991	1381	cUMR	-0.020133333	0.054287533	0.062540442	Non	0.140166667	0.0000025	0.0000171	Hypermethylated	0.027033333	0.192402387	0.238601931	Non	-0.007633333	0.551484138	0.622466733	Non	0.071866667	0.006926248	0.012866845	Non	0.144616667	0.0000168	0.0000482	Hypermethylated	0.124366667	0.0000188	0.0000527	Hypermethylated	0.06262381	0.00000405	0.00000684	Non	RRAGC	18878
chr1	39324267	39326775	2508	cUMR	-0.0494	0.000000166	0.000000582	Non	-0.026966667	0.005427248	0.01349086	Non	-0.0069	0.048985788	0.071891567	Non	-0.020366667	0.155785692	0.227577814	Non	-0.041316667	0.000407626	0.001080042	Non	-0.046816667	0.000155421	0.000353901	Non	-0.055966667	0.000000405	0.00000169	Non	-0.034480952	0.0000000382	0.000000094	Non	RRAGC	0
chr1	39337873	39340167	2294	cUMR	-0.030733333	0.00000347	0.00000886	Non	-0.016666667	0.043734726	0.076857912	Non	-0.011566667	0.014060331	0.023992768	Non	-0.018266667	0.019781509	0.043335892	Non	-0.026066667	0.001136268	0.002639208	Non	-0.035566667	0.0000024	0.00000853	Non	-0.024666667	0.000306921	0.000651476	Non	-0.022809524	0.0000000305	0.0000000764	Non	GJA9	0
chr1	39455928	39458666	2738	cUMR	-0.002433333	0.908477894	0.911655713	Non	0.0000667	0.999875409	0.999875409	Non	-0.006766667	0.192092843	0.238303904	Non	-0.007533333	0.191648636	0.268836883	Non	0.001066667	0.101294489	0.134630997	Non	-0.010183333	0.120655776	0.143547908	Non	0.005066667	0.64745057	0.675132808	Non	-0.002961905	0.103119215	0.107341664	Non	AKIRIN1	0
chr1	39491322	39493023	1701	cUMR	-0.040033333	0.00010386	0.00019158	Non	-0.0016	0.923391353	0.937983393	Non	-0.0107	0.039665421	0.059752286	Non	0.0008	0.238569815	0.320567381	Non	-0.03795	0.000829222	0.002008501	Non	-0.0187	0.256704033	0.285835588	Non	-0.0542	0.000011	0.0000325	Non	-0.023028571	0.0000352	0.0000517	Non	NDUFS5	0
chr1	39546385	39548034	1649	cUMR	-0.027033333	0.264679144	0.279820919	Non	0.0838	0.000403252	0.001460786	Non	-0.0367	0.045106335	0.066861879	Non	0.0126	0.424367891	0.50600779	Non	0.02305	0.633562276	0.672936518	Non	0.07955	0.016459507	0.02348657	Non	0.1734	0.00000466	0.000015	Hypermethylated	0.039342857	0.000707975	0.000879558	Non	MACF1	1805
chr1	39567797	39568867	1070	cUMR	-0.2584	0.0000000000000798	0.00000000000143	Hypomethylated	-0.0511	0.006461562	0.015605321	Non	-0.099733333	0.0001558	0.000461983	Non	-0.1077	0.0000133	0.000115816	Hypomethylated	-0.1284	0.0000343	0.000124397	Hypomethylated	-0.2244	0.000000000141	0.00000000132	Hypomethylated	-0.1053	0.00000183	0.00000647	Hypomethylated	-0.139442857	0.000000000000419	0.0000000000036	Hypomethylated	MACF1	0
chr1	39570325	39573324	2999	cUMR	-0.003666667	0.761159054	0.768558237	Non	0.030166667	0.278356994	0.351071312	Non	-0.039833333	0.000953085	0.002256576	Non	-0.017433333	0.283250195	0.367648562	Non	0.006666667	0.933993741	0.942007331	Non	0.014166667	0.586871608	0.610561327	Non	-0.017633333	0.252156642	0.287824086	Non	-0.00577619	0.242167059	0.247423971	Non	MACF1	0
chr1	39872953	39875787	2834	cUMR	-0.016333333	0.316915157	0.33201554	Non	0.038466667	0.078191518	0.124565716	Non	-0.0215	0.18514527	0.230694452	Non	-0.012733333	0.589813618	0.656141103	Non	-0.002083333	0.45534205	0.504173531	Non	0.003416667	0.174860491	0.201250745	Non	-0.032533333	0.024329906	0.034562754	Non	-0.007304762	0.172510482	0.17738341	Non	KIAA0754	0
chr1	39956294	39958393	2099	cUMR	0.0516	0.00000171	0.0000047	Non	0.180166667	0.000000000359	0.00000000939	Hypermethylated	0.197933333	0.000000000043	0.000000000743	Hypermethylated	0.021566667	0.006495545	0.017593168	Non	-0.000233333	0.036713829	0.055350966	Non	0.181266667	0.00000000193	0.0000000137	Hypermethylated	0.099966667	0.00000275	0.00000927	Non	0.106566667	0.0000000000159	0.0000000000869	Hypermethylated	BMP8A	0
chr1	39980105	39981554	1449	cUMR	-0.008166667	0.129343221	0.142052362	Non	0.0683	0.000818564	0.002681841	Non	0.285	0.0000000000684	0.00000000112	Hypermethylated	0.1641	0.0000000195	0.000000569	Hypermethylated	0.24125	0.000000000311	0.00000000696	Hypermethylated	0.40825	0.00000000000459	0.0000000000684	Hypermethylated	0.1555	0.00000112	0.00000417	Hypermethylated	0.1771	0.00000000000145	0.0000000000106	Hypermethylated	BMP8A	0
chr1	40039596	40042795	3199	cUMR	-0.0223	0.00000271	0.00000711	Non	-0.011633333	0.053023565	0.090214562	Non	-0.003466667	0.057314854	0.082423665	Non	-0.004033333	0.500051991	0.57637408	Non	-0.015133333	0.001110537	0.002585674	Non	-0.004133333	0.265928315	0.295369678	Non	-0.016833333	0.001102666	0.002082275	Non	-0.011261905	0.00001	0.000016	Non	PABPC4	0
chr1	40071467	40072371	904	cUMR	0.052366667	0.082756112	0.093050429	Non	0.1041	0.032622984	0.06041934	Non	0.144366667	0.00200246	0.004337164	Non	0.1225	0.001213558	0.004601056	Non	0.02645	0.812536397	0.835630851	Non	0.13545	0.001082694	0.0020216	Non	0.1185	0.019545485	0.028320385	Non	0.101528571	0.00000286	0.00000494	Non	HEYL	16732
chr1	40104932	40105764	832	cUMR	-0.043333333	0.016363155	0.020362474	Non	0.234633333	0.0000299	0.00015086	Hypermethylated	0.568166667	0.00000000000101	0.000000000028	Hypermethylated	-0.078166667	0.0000818	0.00050948	Non	-0.050416667	0.15909501	0.199576863	Non	0.156583333	0.000368529	0.000767837	Hypermethylated	0.055833333	0.004664778	0.007727381	Non	0.132433333	0.00000000373	0.0000000114	Hypermethylated	HEYL	0
chr1	40119091	40119881	790	cUMR	-0.1446	0.000000174	0.000000605	Hypomethylated	-0.086233333	0.009421218	0.021488224	Non	-0.049433333	0.144522357	0.185575732	Non	-0.068033333	0.059958646	0.106194579	Non	-0.013433333	0.025282328	0.039948432	Non	-0.073933333	0.012163504	0.017869665	Non	-0.071633333	0.019810437	0.028671529	Non	-0.075519048	0.00000918	0.0000147	Non	NT5C1A	4912
chr1	40123717	40124050	333	cUMR	-0.060944324	0.000145487	0.000260645	Non	0.076633333	0.260992732	0.333340852	Non	0.175033333	0.003593172	0.007243455	Non	-0.021766667	0.578473019	0.64626995	Non	0.148783333	0.010286146	0.018192701	Non	-0.075216667	0.113725949	0.135940869	Non	0.008233333	0.631483118	0.660110937	Non	0.03697944	0.002490747	0.002926048	Non	NT5C1A	743
chr1	40136494	40138543	2049	cUMR	-0.004666667	0.925058598	0.927676326	Non	0.1341	0.000000379	0.00000332	Hypermethylated	0.026333333	0.215376388	0.263726636	Non	0.0195	0.435423527	0.516100592	Non	0.1725	0.00000000661	0.000000084	Hypermethylated	0.24775	0.000000000223	0.00000000199	Hypermethylated	0.1441	0.000000719	0.00000281	Hypermethylated	0.094271429	0.000000000883	0.00000000313	Non	NT5C1A	0
chr1	40156554	40158558	2004	cUMR	-0.0374	0.00019068	0.000333813	Non	0.176	0.0000000000349	0.00000000145	Hypermethylated	0.076266667	0.0000264	0.0000967	Non	-0.019	0.132704493	0.20018234	Non	0.0611	0.000000754	0.00000457	Non	0.10585	0.00000314	0.0000108	Hypermethylated	0.0662	0.0000564	0.000141558	Non	0.057628571	0.0000000018	0.00000000591	Non	HPCAL4	0
chr1	40204139	40205338	1199	cUMR	-0.010333333	0.000000103	0.000000378	Non	0.031866667	0.017122238	0.03536727	Non	0.035666667	0.0000476	0.000162612	Non	0.004866667	0.714888228	0.764879302	Non	0.018166667	0.00000000849	0.000000103	Non	0.053416667	0.000000477	0.000002	Non	-0.021733333	0.009522974	0.014762324	Non	0.014666667	0.00000000305	0.0000000095	Non	PPIE	0
chr1	40253388	40255387	1999	cUMR	-0.033933333	0.000450132	0.000732083	Non	0.0976	0.0000977	0.000425699	Non	0.131066667	0.000000392	0.00000234	Hypermethylated	-0.0102	0.474002713	0.553014639	Non	0.0224	0.112999336	0.14825818	Non	0.02965	0.012128842	0.017825263	Non	0.0696	0.000416478	0.000859789	Non	0.045028571	0.000000573	0.00000112	Non	BMP8B	0
chr1	40347655	40349588	1933	cUMR	-0.0644	0.000000378	0.00000121	Non	-0.001533333	0.082809999	0.130568118	Non	-0.055233333	0.0000111	0.000045	Non	-0.033333333	0.0446852	0.08368231	Non	-0.051483333	0.0000692	0.000229083	Non	-0.061483333	0.0000838	0.000204076	Non	-0.054333333	0.000226595	0.000495556	Non	-0.046161905	0.0000000152	0.0000000409	Non	TRIT1	0
chr1	40364605	40368454	3849	Canyon	-0.0419	0.000720415	0.001128409	Non	0.003	0.249888039	0.321646924	Non	-0.013566667	0.346494373	0.399131042	Non	-0.0166	0.203720312	0.282400603	Non	-0.02365	0.002070521	0.004452562	Non	-0.0144	0.272180209	0.301924079	Non	-0.0184	0.065214107	0.084670463	Non	-0.018428571	0.004048511	0.004666874	Non	MYCL	0
chr1	40420478	40422177	1699	cUMR	-0.036933333	0.0000187	0.0000406	Non	0.001366667	0.974910821	0.97924481	Non	0.028566667	0.033890222	0.051995071	Non	-0.019633333	0.098315772	0.157485607	Non	-0.037183333	0.00020793	0.000600224	Non	-0.021183333	0.023983389	0.033078738	Non	-0.004233333	0.026768292	0.037704376	Non	-0.011319048	0.000431718	0.000550335	Non	MFSD2A	0
chr1	40504468	40507417	2949	cUMR	-0.004633333	0.380090824	0.394871041	Non	-0.006166667	0.459344651	0.528950973	Non	-0.002466667	0.801995345	0.826483229	Non	-0.004766667	0.615170011	0.677902274	Non	-0.004966667	0.276561721	0.325101676	Non	-0.013966667	0.013250038	0.01928253	Non	-0.009366667	0.125214314	0.152891665	Non	-0.006280952	0.041347942	0.044062874	Non	CAP1	0
chr1	40550534	40550733	199	cUMR	-0.116286499	0.000000528	0.00000164	Hypomethylated	-0.1055	0.000385839	0.001406548	Non	-0.100812673	0.0000976	0.000305754	Hypomethylated	-0.062477134	0.001712282	0.006053781	Non	-0.07825	0.062749949	0.088481466	Non	-0.081209378	0.000869032	0.001659029	Non	0.073131609	0.0000325	0.0000862	Non	-0.068988754	0.000000125	0.000000276	Non	PPT1	0
chr1	40562336	40563435	1099	cUMR	-0.009466667	0.000768315	0.001196712	Non	-0.0102	0.003677781	0.009717489	Non	-0.002133333	0.201494197	0.248584813	Non	-0.0078	0.036247805	0.070483202	Non	-0.015250099	0.000000792	0.00000477	Non	-0.0133	0.001047973	0.001964685	Non	0.0008	0.06936123	0.089567427	Non	-0.007708978	0.00000367	0.00000624	Non	PPT1	0
chr1	40609565	40610274	709	cUMR	-0.035033333	0.000517868	0.000833251	Non	-0.0204	0.303339686	0.376355495	Non	-0.072015392	0.0000000000399	0.000000000694	Non	-0.0046	0.279275966	0.363699688	Non	0.00105	0.000865691	0.002087185	Non	-0.06645	0.0005263	0.001057868	Non	-0.001	0.100891449	0.125669701	Non	-0.029539728	0.0000000199	0.0000000521	Non	RLF	16767
chr1	40626533	40628903	2370	cUMR	-0.017266667	0.0000000117	0.0000000545	Non	0.0026	0.190570807	0.257915458	Non	-0.0096	0.002205461	0.004714547	Non	-0.0046	0.225264063	0.306156646	Non	-0.0156	0.00000131	0.00000739	Non	-0.0086	0.015676337	0.022468826	Non	0.0006	0.323381225	0.360632851	Non	-0.007571429	0.000000216	0.000000456	Non	RLF	0
chr1	40722212	40724661	2449	cUMR	-0.065033333	0.000000000153	0.00000000117	Non	-0.036033333	0.000422863	0.001521989	Non	0.004633333	0.296668564	0.348686488	Non	-0.020233333	0.000662349	0.002806627	Non	-0.044783333	0.00000186	0.00000998	Non	-0.046033333	0.0000966	0.000231675	Non	-0.052033333	0.00000153	0.00000551	Non	-0.036204762	0.000000000473	0.00000000179	Non	ZMPSTE24	0
chr1	40780596	40783245	2649	cUMR	0.015133333	0.0000246	0.000052	Non	0.119733333	0.0000000536	0.000000617	Hypermethylated	0.125633333	0.000000000918	0.0000000112	Hypermethylated	-0.000466667	0.562115979	0.631774212	Non	0.117383333	0.000000000192	0.00000000479	Hypermethylated	0.105633333	0.000000223	0.00000101	Hypermethylated	0.065533333	0.000029	0.0000777	Non	0.076019048	0.0000000000119	0.000000000067	Non	COL9A2	0
chr1	40804342	40805251	909	cUMR	-0.209966667	0.00000000221	0.0000000124	Hypomethylated	-0.1315	0.00032897	0.001222199	Non	-0.0113	0.328852253	0.381379354	Non	-0.2159	0.00000000806	0.000000293	Hypomethylated	-0.13455	0.000549202	0.00140555	Non	-0.15205	0.0000862	0.00020926	Hypomethylated	-0.0937	0.002489912	0.004367223	Non	-0.1337	0.000000000743	0.00000000268	Hypomethylated	COL9A2	-21404
chr1	40837811	40840960	3149	cUMR	-0.046	0.0000398	0.0000801	Non	-0.023633333	0.007847957	0.018405925	Non	-0.0425	0.000232513	0.000655028	Non	-0.016233333	0.148807643	0.219472831	Non	-0.017083333	0.353838272	0.403387013	Non	-0.023833333	0.002459061	0.00424123	Non	-0.028033333	0.050094558	0.066733662	Non	-0.029547619	0.0000102	0.0000162	Non	SMAP2	0
chr1	40870502	40870740	238	cUMR	-0.063333333	0.023890063	0.028969091	Non	-0.010666667	0.7725869	0.810105153	Non	-0.0145	0.57614489	0.620234066	Non	-0.015866667	0.636477937	0.697301569	Non	0.020833333	0.217317675	0.262607629	Non	-0.008666667	0.00523958	0.008361261	Non	0.142933333	0.000297717	0.000633952	Hypermethylated	0.00467619	0.004834741	0.005534276	Non	SMAP2	0
chr1	40914783	40916874	2091	cUMR	-0.049233333	0.00000000451	0.0000000234	Non	-0.006	0.521179624	0.585658711	Non	-0.020733333	0.000000297	0.00000183	Non	-0.0282	0.008650452	0.022167014	Non	-0.0209	0.025513589	0.040274235	Non	0.0486	0.0000237	0.0000658	Non	0.027	0.002063655	0.003673056	Non	-0.009857143	0.00000000979	0.0000000273	Non	ZFP69B	0
chr1	40942543	40944150	1607	cUMR	-0.008033333	0.011097354	0.014166659	Non	0.000566667	0.34082575	0.413143423	Non	0.067133333	0.00000226	0.000011	Non	-0.007033333	0.691718081	0.744806622	Non	0.009966667	0.117036237	0.152837625	Non	0.191466667	0.000000000204	0.00000000183	Hypermethylated	0.018566667	0.023251403	0.033173415	Non	0.034880952	0.0000000622	0.000000146	Non	ZFP69	0
chr1	40973813	40975112	1299	cUMR	-0.006666667	0.002494998	0.003551566	Non	-0.0063	0.218767749	0.289221838	Non	0.109333333	0.000000000000000777	0.0000000000000646	Hypermethylated	0.0041	0.514920184	0.589787851	Non	-0.00025	0.976619449	0.979505368	Non	0.05375	0.000000000586	0.00000000471	Non	0.1785	0.000000000000000111	0.000000000000026	Hypermethylated	0.0489	0.00000000000000155	0.0000000000000293	Non	EXO5	0
chr1	40996376	40998225	1849	cUMR	-0.044066667	0.0000000566	0.000000222	Non	-0.0182	0.007542592	0.017791096	Non	-0.033233333	0.000000000691	0.00000000867	Non	-0.0132	0.269179193	0.35297819	Non	-0.0304	0.000036	0.000129786	Non	-0.01415	0.085434973	0.104699551	Non	-0.0008	0.641571008	0.669610387	Non	-0.022942857	0.0000000125	0.0000000342	Non	ZNF684	0
chr1	41130626	41131975	1349	cUMR	-0.0348	0.001698942	0.002488669	Non	0.0185	0.221248987	0.291877633	Non	-0.017633333	0.093663465	0.126632753	Non	-0.0211	0.003909666	0.0117183	Non	0.0087	0.513378775	0.559791934	Non	-0.00405	0.258046243	0.287223943	Non	-0.0023	0.910041122	0.918541904	Non	-0.009157143	0.077510921	0.081239482	Non	RIMS3	0
chr1	41134250	41135263	1013	cUMR	-0.0678	0.000000134	0.00000048	Non	-0.015466667	0.424606411	0.495933617	Non	-0.0388	0.0000000292	0.00000024	Non	-0.022266667	0.175521592	0.250353319	Non	-0.024466667	0.125196812	0.161994887	Non	-0.057966667	0.000019	0.0000541	Non	-0.040266667	0.003020747	0.005212168	Non	-0.038838095	0.0000000181	0.0000000478	Non	RIMS3	-2927
chr1	41156595	41158986	2391	cUMR	-0.010933333	0.000715316	0.001121223	Non	0.005266667	0.252044356	0.323946173	Non	0.0000667	0.001595507	0.003555111	Non	-0.010333333	0.015536089	0.035520746	Non	0.006066667	0.000452795	0.00118461	Non	-0.023183333	0.00000488	0.000016	Non	0.000666667	0.077035309	0.098370048	Non	-0.004447619	0.00000731	0.0000119	Non	NFYC	0
chr1	41174267	41176629	2362	cUMR	-0.0425	0.217850183	0.232598746	Non	0.221933333	0.000139925	0.000581041	Hypermethylated	-0.029166667	0.260273359	0.311160759	Non	0.012333333	0.938136187	0.95080144	Non	0.067833333	0.112526979	0.147726909	Non	0.016833333	0.883690099	0.89186933	Non	0.019333333	0.550682622	0.582700179	Non	0.033619048	0.019706195	0.02146932	Non	NFYC	0
chr1	41248116	41250265	2149	cUMR	-0.037933333	0.001171139	0.00176631	Non	0.004533333	0.251873423	0.323770078	Non	0.122566667	0.00000439	0.0000198	Hypermethylated	-0.015866667	0.468473976	0.547518594	Non	0.001983333	0.121403676	0.157713519	Non	0.018983333	0.076911968	0.095188979	Non	-0.020866667	0.267411734	0.303383075	Non	0.012304762	0.00033403	0.000431537	Non	KCNQ4	0
chr1	41267535	41268434	899	cUMR	-0.055466667	0.004020318	0.005524728	Non	0.098766667	0.002638623	0.007302504	Non	0.189533333	0.000000259	0.00000163	Hypermethylated	-0.024633333	0.45415925	0.533817657	Non	0.014366667	0.144541852	0.183740547	Non	-0.009133333	0.237713316	0.266326444	Non	0.015966667	0.687451143	0.712342941	Non	0.036452381	0.0000533	0.0000763	Non	KCNQ4	0
chr1	41315480	41316227	747	cUMR	-0.073	0.000212224	0.000367847	Non	-0.0197	0.688029693	0.736046682	Non	-0.089	0.0000147	0.0000576	Non	-0.0479	0.078961803	0.13235178	Non	-0.0225	0.578118202	0.620999039	Non	-0.0495	0.098156991	0.118947359	Non	0.0233	0.597391598	0.627222133	Non	-0.042328571	0.00019602	0.000260618	Non	KCNQ4	-9357
chr1	41326300	41328632	2332	cUMR	-0.022266667	0.087753615	0.098417025	Non	0.0573	0.001772828	0.005193376	Non	0.099066667	0.0000118	0.0000475	Non	0.0371	0.023562898	0.049913741	Non	0.0109	0.755275536	0.784104337	Non	0.0704	0.000279005	0.000598439	Non	0.0283	0.00000195	0.00000683	Non	0.039985714	0.00000137	0.00000251	Non	CITED4	0
chr1	41417826	41418825	999	cUMR	-0.132533333	0.000000000223	0.00000000162	Hypomethylated	-0.043533333	0.07125091	0.115486189	Non	-0.0832	0.0000825	0.00026416	Non	-0.040933333	0.124591018	0.190217585	Non	-0.102033333	0.0000000957	0.000000784	Hypomethylated	-0.087283333	0.000193436	0.000430377	Non	0.012466667	0.532692232	0.565127989	Non	-0.068904762	0.00000000359	0.000000011	Non	CTPS1	26146
chr1	41444751	41446941	2190	cUMR	-0.018466667	0.001916969	0.002781833	Non	0.003	0.813955701	0.845462758	Non	0.001866667	0.001855498	0.004059723	Non	0.0126	0.488179296	0.565828709	Non	0.01245	0.058526716	0.083144303	Non	0.0072	0.057482575	0.07293288	Non	0.0062	0.023049772	0.032907176	Non	0.002514286	0.000449735	0.000572061	Non	CTPS1	0
chr1	41706532	41708481	1949	cUMR	-0.012333333	0.069637259	0.07901119	Non	-0.006033333	0.056379047	0.095084399	Non	0.000166667	0.985007736	0.987302072	Non	0.004566667	0.81392268	0.849208615	Non	0.005166667	0.617491768	0.657577296	Non	0.105166667	0.000000000224	0.00000000199	Hypermethylated	0.014166667	0.193328922	0.226360639	Non	0.012338095	0.0000151	0.0000235	Non	SCMH1	0
chr1	41733800	41734160	360	cUMR	-0.081633333	0.000114344	0.000209127	Non	-0.023566667	0.037517067	0.067713936	Non	0.002866667	0.201929624	0.248994529	Non	-0.045366667	0.057481883	0.102690964	Non	-0.042466667	0.01851933	0.03048769	Non	-0.031216667	0.105648478	0.12716233	Non	-0.116366667	0.000000404	0.00000168	Hypomethylated	-0.048395238	0.0000818	0.00011411	Non	SCMH1	-25986
chr1	41825879	41828628	2749	cUMR	-0.040033333	0.000303748	0.000509376	Non	-0.020633333	0.182695359	0.249146014	Non	-0.004033333	0.0988906	0.132820515	Non	-0.018433333	0.242753354	0.325119232	Non	-0.019283333	0.044777555	0.065815769	Non	0.005716667	0.254061369	0.283115416	Non	0.003366667	0.096509786	0.120723657	Non	-0.014204762	0.009532564	0.010645433	Non	FOXO6	0
chr1	41831357	41833306	1949	cUMR	-0.031033333	0.096632451	0.107873902	Non	0.0249	0.124789329	0.182192881	Non	-0.053366667	0.002533726	0.005333951	Non	-0.0383	0.105551838	0.166792668	Non	-0.04795	0.008088617	0.014716459	Non	0.02305	0.666170446	0.686393681	Non	0.0709	0.000579144	0.00116263	Non	-0.0091	0.001555714	0.001865897	Non	FOXO6	0
chr1	41869195	41870551	1356	cUMR	-0.126466667	0.000000197	0.000000677	Hypomethylated	0.111133333	0.000906532	0.002930024	Non	-0.0843	0.000581936	0.001459608	Non	-0.034266667	0.330669015	0.415933914	Non	-0.032466667	0.0000493	0.000171121	Non	-0.080216667	0.004231092	0.00689717	Non	0.006733333	0.001740087	0.003139584	Non	-0.037066667	0.00000123	0.00000227	Non	FOXO6	-19933
chr1	41882695	41883126	431	cUMR	-0.015333333	0.058670423	0.067248852	Non	0.000166667	0.790119592	0.825007012	Non	0.083666667	0.052910501	0.076841876	Non	-0.039633333	0.055550787	0.099932575	Non	0.056166667	0.245777561	0.292756051	Non	-0.079083333	0.00871422	0.013218889	Non	0.016566667	0.065919335	0.085484758	Non	0.00582381	0.018212474	0.019906754	Non	FOXO6	-33433
chr1	41961234	41962683	1449	cUMR	-0.057033333	0.000000175	0.00000061	Non	0.000466667	0.114424835	0.169899269	Non	0.0993	0.00000000601	0.0000000602	Non	-0.063733333	0.000000605	0.00000928	Non	-0.014783333	0.0000403	0.000143156	Non	-0.049783333	0.002467229	0.00425349	Non	0.001466667	0.372397032	0.409101435	Non	-0.008961905	0.00000000287	0.000000009	Non	HIVEP3	9353
chr1	41980795	41982644	1849	cUMR	-0.0913	0.000064	0.000123483	Non	-0.055666667	0.025699274	0.049641315	Non	0.008533333	0.014859803	0.025182245	Non	-0.039466667	0.101387653	0.161545779	Non	-0.089716667	0.001306634	0.002981184	Non	-0.084966667	0.004567601	0.007389731	Non	-0.084266667	0.0000343	0.0000906	Non	-0.059780952	0.00000288	0.00000498	Non	HIVEP3	0
chr1	42039633	42040281	648	cUMR	-0.043638706	0.586651196	0.598118147	Non	0.018929249	0.084828773	0.13319339	Non	-0.10680422	0.030030419	0.046770053	Non	-0.09087148	0.07511631	0.127283833	Non	-0.046221856	0.500293829	0.547207729	Non	-0.059970721	0.242198041	0.270910155	Non	0.033928924	0.232479801	0.26753108	Non	-0.043357453	0.210842841	0.216061013	Non	HIVEP3	0
chr1	42126697	42128730	2033	cUMR	-0.030266667	0.00000782	0.0000185	Non	0.081633333	0.0000539	0.0002534	Non	0.0089	0.015886297	0.026718871	Non	-0.018566667	0.023542575	0.049888231	Non	-0.012266667	0.444048952	0.493142156	Non	0.088483333	0.0000266	0.0000729	Non	0.024633333	0.084231004	0.106654244	Non	0.01757619	0.0000283	0.0000422	Non	HIVEP3	0
chr1	42383051	42386766	3715	Canyon	-0.1026	0.0000000198	0.0000000871	Hypomethylated	0.0329	0.040598431	0.072209949	Non	-0.028266667	0.11545503	0.152284363	Non	0.0193	0.73142267	0.779283027	Non	0.10015	0.000000473	0.00000305	Hypermethylated	-0.07835	0.000138006	0.000319162	Non	-0.0433	0.050752104	0.067520025	Non	-0.018671429	0.000000269	0.000000558	Non	HIVEP3	0
chr1	42500086	42502185	2099	cUMR	-0.0122	0.053227003	0.061386184	Non	-0.013233333	0.120613696	0.177361826	Non	-0.0157	0.019859349	0.032577985	Non	-0.018833333	0.002154369	0.007275223	Non	0.018216667	0.007859554	0.014356063	Non	0.018966667	0.000391406	0.000810179	Non	-0.002833333	0.517146644	0.550425194	Non	-0.005519048	0.000616104	0.000770665	Non	HIVEP3	0
chr1	42612430	42613243	813	cUMR	-0.115833333	0.000061	0.000118286	Hypomethylated	-0.065333333	0.083683939	0.131713381	Non	0.056833333	0.058948852	0.084526325	Non	-0.064733333	0.107273034	0.168936451	Non	0.008416667	0.014793596	0.025066523	Non	0.029666667	0.404143176	0.433539987	Non	-0.002933333	0.455079085	0.490382683	Non	-0.024761905	0.001111221	0.001352844	Non	GUCA2B	5849
chr1	42631318	42632017	699	cUMR	-0.35687385	0.000426822	0.000696995	Hypomethylated	-0.245471838	0.000902109	0.002919043	Non	0.062595511	0.46326395	0.512944985	Non	-0.23187107	0.060577537	0.107024979	Non	-0.315283245	0.006735943	0.01255882	Non	-0.314322075	0.022939563	0.031746258	Non	-0.07227302	0.829128272	0.843979167	Non	-0.200920129	0.005094879	0.005822374	Non	GUCA2A	-924
chr1	42798721	42801975	3254	cUMR	-0.009166667	0.004428588	0.006042016	Non	-0.004533333	0.503177281	0.569358703	Non	0.002333333	0.008969635	0.016152314	Non	0.002666667	0.816817214	0.851795074	Non	-0.007083333	0.27490944	0.323332895	Non	-0.011583333	0.023253711	0.032144091	Non	0.002666667	0.82986026	0.844652903	Non	-0.003190476	0.009966508	0.011115514	Non	FOXJ3	0
chr1	42845659	42847129	1470	cUMR	-0.008733333	0.02304462	0.028000265	Non	0.009433333	0.168707919	0.233305867	Non	-0.006233333	0.039733993	0.059851841	Non	-0.004366667	0.18960756	0.266532286	Non	0.009683333	0.19539277	0.239536804	Non	0.047683333	0.00000018	0.000000833	Non	0.020833333	0.000222008	0.000486629	Non	0.007690476	0.0000223	0.0000337	Non	RIMKLA	0
chr1	42921183	42923032	1849	cUMR	-0.046866667	0.00000000000179	0.0000000000225	Non	-0.012966667	0.230108351	0.301010854	Non	0.1693	0.00000000000000022	0.0000000000000218	Hypermethylated	-0.045566667	0.00000018	0.00000346	Non	0.002883333	0.001357383	0.003081508	Non	-0.048366667	0.000000955	0.00000373	Non	-0.009766667	0.000848375	0.00164025	Non	0.006033333	0.00000000000000833	0.000000000000122	Non	ZMYND12	0
chr1	42927782	42929531	1749	cUMR	-0.026933333	0.00000865	0.0000202	Non	-0.010466667	0.203946947	0.27311935	Non	0.0004	0.084621496	0.115890476	Non	-0.032466667	0.0000106	0.0000961	Non	0.023983333	0.0000758	0.000247603	Non	0.008983333	0.004176812	0.006818373	Non	-0.022866667	0.002165637	0.003837281	Non	-0.010180952	0.0000013	0.00000238	Non	PPCS	0
chr1	43123066	43125184	2118	cUMR	-0.039233333	0.0000000189	0.0000000836	Non	-0.024533333	0.000136854	0.000570252	Non	-0.001566667	0.823199031	0.845117805	Non	-0.018333333	0.005944983	0.016402816	Non	-0.045983333	0.00000000245	0.000000037	Non	-0.032733333	0.0000331	0.0000887	Non	-0.032733333	0.000000588	0.00000236	Non	-0.026790476	0.000000000183	0.000000000761	Non	PPIH	0
chr1	43146493	43149992	3499	cUMR	-0.0192	0.0000363	0.0000737	Non	0.0088	0.379281675	0.451581132	Non	-0.0122	0.005803471	0.011029341	Non	0.005	0.013552339	0.031793639	Non	-0.0097	0.058079476	0.082625847	Non	-0.01795	0.006745367	0.010501314	Non	-0.0104	0.046683822	0.062577514	Non	-0.008057143	0.000684372	0.000851639	Non	YBX1	0
chr1	43230547	43233682	3135	cUMR	-0.004166667	0.760646509	0.768105078	Non	0.039966667	0.000181861	0.00073002	Non	-0.030333333	0.001842078	0.004033656	Non	-0.015633333	0.338627333	0.423776561	Non	0.022916667	0.33173854	0.381222477	Non	0.015666667	0.278593223	0.308455754	Non	0.002166667	0.585137097	0.615755458	Non	0.002280952	0.180699246	0.185573851	Non	LEPRE1	0
chr1	43250631	43251419	788	cUMR	0.0457	0.0000853	0.000160183	Non	0.389133333	0.000000000332	0.00000000894	Hypermethylated	0.1262	0.0000434	0.000149852	Hypermethylated	0.174933333	0.000000697	0.0000103	Hypermethylated	0.109283333	0.00000296	0.0000147	Hypermethylated	0.414533333	0.000000000538	0.00000000436	Hypermethylated	0.356533333	0.00000000083	0.00000000716	Hypermethylated	0.220847619	0.00000000000429	0.0000000000274	Hypermethylated	LOC100129924	2242
chr1	43281678	43283618	1940	cUMR	-0.006566667	0.754960966	0.762715355	Non	0.010933333	0.053272424	0.090561242	Non	0.011433333	0.407497849	0.459216385	Non	0.000133333	0.782259198	0.822787401	Non	-0.004066667	0.48836104	0.535907301	Non	-0.010816667	0.644052219	0.665481965	Non	-0.007866667	0.760512428	0.780464543	Non	-0.000409524	0.716663772	0.719320403	Non	CCDC23	0
chr1	43310745	43313094	2349	cUMR	-0.0139	0.014208879	0.017831766	Non	0.0263	0.019177165	0.038852543	Non	-0.0169	0.0000473	0.000161639	Non	-0.0139	0.019361265	0.042616712	Non	0.00485	0.20762802	0.252455725	Non	0.0041	0.681128842	0.700518238	Non	0.0355	0.001949942	0.003484817	Non	0.002585714	0.000102028	0.000140616	Non	ZNF691	0
chr1	43388292	43391391	3099	cUMR	-0.031866667	0.000613466	0.000974313	Non	-0.0079	0.086978929	0.135940541	Non	-0.0212	0.04726263	0.069668146	Non	-0.0183	0.020457115	0.044504428	Non	-0.01995	0.146002569	0.185392028	Non	-0.00845	0.585793533	0.609544996	Non	0.0049	0.838127406	0.852203476	Non	-0.015385714	0.008316369	0.009330844	Non	SLC2A1	0
chr1	43422228	43425477	3249	cUMR	-0.067833333	0.000000286	0.000000946	Non	-0.035966667	0.024448014	0.047586737	Non	-0.0015	0.447995724	0.498145117	Non	-0.006566667	0.89615595	0.916970238	Non	-0.063916667	0.0000296	0.000109452	Non	-0.059416667	0.0000987	0.000236214	Non	-0.050566667	0.000353617	0.000741258	Non	-0.039280952	0.00000104	0.00000194	Non	SLC2A1	0
chr1	43533584	43534533	949	cUMR	-0.0578	0.000247499	0.000422995	Non	0.091633333	0.001260028	0.003868376	Non	0.041866667	0.00181107	0.003973703	Non	-0.041366667	0.018927834	0.041861504	Non	0.142283333	0.000000000191	0.00000000477	Hypermethylated	0.098033333	0.000385833	0.000800154	Non	0.089233333	0.000000321	0.00000137	Non	0.044661905	0.00000000256	0.00000000815	Non	FAM183A	79061
chr1	43539213	43539612	399	cUMR	-0.041766667	0.016233963	0.020209011	Non	-0.0412	0.069434471	0.113158558	Non	0.006566667	0.936520344	0.945069818	Non	-0.0204	0.528066554	0.601590378	Non	-0.0316	0.187818505	0.231310387	Non	-0.0996	0.00000454	0.000015	Non	0.059	0.001042807	0.001979165	Non	-0.0214	0.000375365	0.00048189	Non	FAM183A	73982
chr1	43612809	43613958	1149	cUMR	0.001433333	0.745654196	0.75385026	Non	0.026233333	0.37578886	0.448108875	Non	-0.014566667	0.647317272	0.686457121	Non	0.028033333	0.143674833	0.213233733	Non	0.088933333	0.00012782	0.000391337	Non	0.231933333	0.0000000169	0.0000000972	Hypermethylated	-0.012566667	0.105893256	0.131302625	Non	0.04037619	0.0000162	0.0000251	Non	FAM183A	0
chr1	43636695	43638744	2049	cUMR	-0.049366667	0.0000000000954	0.000000000773	Non	-0.024966667	0.01956258	0.039530356	Non	-0.022366667	0.000249456	0.000695603	Non	-0.020166667	0.059467332	0.105502744	Non	-0.053866667	0.0000000135	0.000000151	Non	-0.042116667	0.000117484	0.000275938	Non	-0.043366667	0.000000451	0.00000186	Non	-0.035909524	0.0000000000507	0.000000000239	Non	MIR6733	0
chr1	43692669	43693473	804	cUMR	-0.094766667	0.00000361	0.00000918	Non	-0.0677	0.008979415	0.020635971	Non	-0.0631	0.009772103	0.017426785	Non	-0.1301	0.000000139	0.00000281	Hypomethylated	-0.0326	0.008705816	0.015699525	Non	-0.11185	0.0000128	0.000038	Hypomethylated	-0.0221	0.134575336	0.16324644	Non	-0.074985714	0.0000000518	0.000000124	Non	WDR65	0
chr1	43823702	43825753	2051	cUMR	-0.045833333	0.000000253	0.000000847	Non	-0.032366667	0.000109899	0.000472058	Non	-0.0055	0.026396474	0.041832319	Non	-0.006366667	0.731997802	0.779723425	Non	-0.040166667	0.0000697	0.000230389	Non	-0.040666667	0.0000358	0.0000953	Non	-0.034166667	0.0000453	0.00011622	Non	-0.028452381	0.00000016	0.000000346	Non	CDC20	0
chr1	43831658	43834457	2799	cUMR	-0.059333333	0.0000179	0.0000389	Non	-0.0505	0.000266899	0.001014106	Non	0.078333333	0.001235439	0.002835391	Non	-0.0547	0.000329882	0.001589898	Non	-0.06125	0.000135327	0.000410991	Non	-0.06625	0.0000203	0.0000572	Non	-0.0603	0.0000152	0.0000435	Non	-0.034957143	0.00000184	0.00000329	Non	ELOVL1	0
chr1	43853712	43856473	2761	cUMR	-0.0388	0.00000213	0.00000572	Non	-0.012766667	0.324225161	0.397075305	Non	-0.010966667	0.220274009	0.268958405	Non	-0.021766667	0.045258521	0.084526066	Non	-0.034716667	0.000231173	0.0006574	Non	-0.027966667	0.00742576	0.01144961	Non	-0.004366667	0.558420936	0.590007493	Non	-0.021252381	0.0000187	0.0000287	Non	MED8	0
chr1	43918485	43921034	2549	cUMR	-0.024566667	0.001659173	0.002434559	Non	0.001	0.673871466	0.722954486	Non	0.017933333	0.022039795	0.035717215	Non	-0.0214	0.011609501	0.028027024	Non	0.0311	0.000437117	0.001148583	Non	0.0076	0.01322579	0.019253057	Non	0.1034	0.00000000021	0.0000000022	Hypermethylated	0.015142857	0.000000084	0.000000192	Non	HYI	0
chr1	43994481	43999480	4999	Canyon	-0.0607	0.00110719	0.001677844	Non	-0.057966667	0.00135582	0.00411943	Non	0.008966667	0.529611957	0.575773208	Non	-0.033166667	0.126025772	0.192018782	Non	-0.052366667	0.019794596	0.032313384	Non	-0.059116667	0.007801526	0.011959499	Non	-0.018966667	0.490313054	0.524579671	Non	-0.037338095	0.000716084	0.000889128	Non	PTPRF	0
chr1	44114708	44117757	3049	cUMR	-0.046433333	0.0000575	0.000112042	Non	0.0162	0.181036198	0.247317267	Non	-0.0051	0.028249953	0.044361226	Non	-0.025	0.061163956	0.107869513	Non	-0.04885	0.000717068	0.001770302	Non	-0.0406	0.007431068	0.011455596	Non	-0.0344	0.000357805	0.000749124	Non	-0.025228571	0.0000104	0.0000165	Non	KDM4A	0
chr1	44171796	44174080	2284	cUMR	-0.014733333	0.001029982	0.001569825	Non	0.003733333	0.330419886	0.403023284	Non	0.015933333	0.006586542	0.012311613	Non	-0.004666667	0.350815994	0.435479515	Non	-0.010066667	0.120520917	0.156744188	Non	-0.009566667	0.005819675	0.009198042	Non	0.001733333	0.567489251	0.59880106	Non	-0.00192381	0.033281923	0.035688506	Non	KDM4A-AS1	0
chr1	44398868	44400717	1849	cUMR	-0.075866667	0.00000853	0.00002	Non	0.040833333	0.110926986	0.165671991	Non	0.354966667	0.000000000000397	0.0000000000126	Hypermethylated	-0.051766667	0.014366756	0.033343936	Non	-0.080366667	0.0000869	0.000279683	Non	-0.019116667	0.334311222	0.364386983	Non	0.020633333	0.00614167	0.009938863	Non	0.037861905	0.00000000234	0.00000000752	Non	ARTN	0
chr1	44401318	44402717	1399	cUMR	-0.0301	0.001100759	0.001669147	Non	0.111933333	0.000000202	0.00000192	Hypermethylated	0.0394	0.106730819	0.141989582	Non	-0.004466667	0.900216574	0.920265515	Non	-0.067016667	0.000493853	0.001279252	Non	-0.031016667	0.000271495	0.000584099	Non	-0.076666667	0.000000261	0.00000114	Non	-0.005209524	0.00000131	0.0000024	Non	ARTN	0
chr1	44411569	44414021	2452	cUMR	-0.013975891	0.001288335	0.00192966	Non	0.002566667	0.42214275	0.493732653	Non	-0.0155	0.016900945	0.028232288	Non	0.000966667	0.148864665	0.219543495	Non	-0.014833333	0.041646567	0.061862724	Non	-0.014083333	0.045364744	0.058885243	Non	-0.001433333	0.596523344	0.626496521	Non	-0.008058361	0.017247661	0.018885184	Non	IPO13	0
chr1	44435203	44436391	1188	cUMR	-0.022870132	0.0000000047	0.0000000242	Non	0.0022	0.161301028	0.224895612	Non	-0.027066667	0.000142211	0.000425819	Non	-0.0184	0.087013363	0.142914942	Non	-0.0234	0.012968914	0.022313643	Non	-0.01365	0.316796248	0.346859732	Non	0.0114	0.434310351	0.470068823	Non	-0.0134814	0.0000014	0.00000256	Non	DPH2	0
chr1	44439727	44441676	1949	cUMR	-0.0159	0.00000866	0.0000203	Non	-0.010233333	0.01586052	0.033182058	Non	0.004433333	0.174612469	0.2192096	Non	-0.006833333	0.076839254	0.12954193	Non	-0.016483333	0.000180953	0.000530876	Non	-0.017733333	0.0000105	0.0000318	Non	-0.014433333	0.000163845	0.000370427	Non	-0.01037619	0.000000557	0.00000109	Non	ATP6V0B	0
chr1	44444091	44446790	2699	cUMR	-0.052266667	0.000166595	0.000294911	Non	-0.009166667	0.571349128	0.631724325	Non	-0.0176	0.327273683	0.379920036	Non	-0.019966667	0.116574683	0.180691884	Non	-0.037516667	0.047186077	0.068856127	Non	-0.039016667	0.011450479	0.016923253	Non	0.016033333	0.376422639	0.413111674	Non	-0.022595238	0.01051101	0.011703831	Non	B4GALT2	0
chr1	44456351	44458050	1699	cUMR	-0.0362	0.0000022	0.0000059	Non	-0.0205	0.032226156	0.05986845	Non	0.002633333	0.004616419	0.00900927	Non	-0.0329	0.000196712	0.001040692	Non	-0.0317	0.000890235	0.002136146	Non	-0.04195	0.0000111	0.0000334	Non	-0.0175	0.022220115	0.031828313	Non	-0.0243	0.000000198	0.000000421	Non	CCDC24	0
chr1	44471316	44471865	549	cUMR	-0.063066667	0.00356282	0.00493799	Non	0.011833333	0.511419432	0.576897015	Non	-0.0609	0.0093723	0.016812095	Non	-0.030166667	0.100182739	0.159965786	Non	-0.002316667	0.093360222	0.125364684	Non	-0.041816667	0.016659133	0.023730673	Non	-0.009966667	0.527469468	0.56008039	Non	-0.030338095	0.002924033	0.00341657	Non	SLC6A9	0
chr1	44494853	44497452	2599	cUMR	-0.0299	0.0000000599	0.000000233	Non	-0.011333333	0.182080782	0.248518868	Non	-0.0019	0.698474494	0.732870569	Non	-0.014333333	0.001617358	0.00578523	Non	-0.018733333	0.0163732	0.027398193	Non	-0.015483333	0.021578183	0.030013914	Non	0.005666667	0.000690874	0.001362857	Non	-0.012219048	0.0000128	0.00002	Non	SLC6A9	0
chr1	44513332	44514631	1299	cUMR	-0.152466667	0.0000616	0.000119387	Hypomethylated	0.027366667	0.599141613	0.656012152	Non	0.000866667	0.921038449	0.931435836	Non	-0.130833333	0.002242422	0.007517793	Non	0.035616667	0.144667535	0.183883538	Non	-0.169133333	0.0000422	0.000110317	Hypomethylated	-0.079633333	0.124909314	0.152565634	Non	-0.067404762	0.000290044	0.000377524	Non	SLC6A9	-16169
chr1	44678191	44680090	1899	cUMR	-0.041133333	0.0000408	0.000082	Non	-0.012066667	0.34660818	0.418741467	Non	-0.002966667	0.010206241	0.01809254	Non	-0.028266667	0.010774347	0.026413044	Non	-0.050966667	0.000000417	0.00000275	Non	-0.021716667	0.003326251	0.005553662	Non	-0.010666667	0.351907558	0.388863823	Non	-0.023152381	0.00000769	0.0000125	Non	DMAP1	0
chr1	44820194	44821343	1149	cUMR	-0.035033333	0.00000376	0.00000952	Non	0.0103	0.051804824	0.088559934	Non	-0.0147	0.003084784	0.00634709	Non	-0.0151	0.135149528	0.203044471	Non	-0.03095	0.0000573	0.000194668	Non	-0.03045	0.00079107	0.001526522	Non	-0.0029	0.121506518	0.148771395	Non	-0.016642857	0.0000129	0.0000203	Non	ERI3	0
chr1	44870602	44875279	4677	Canyon	0.203633333	0.00000000437	0.0000000227	Hypermethylated	0.2358	0.000000000413	0.0000000104	Hypermethylated	0.086133333	0.000524109	0.001333121	Non	0.0556	0.012202756	0.029135245	Non	0.2003	0.000000532	0.00000336	Hypermethylated	0.3063	0.000000000442	0.00000000366	Hypermethylated	0.2548	0.00000000925	0.0000000589	Hypermethylated	0.1856	0.000000000000296	0.00000000000266	Hypermethylated	RNF220	0
chr1	44882834	44884633	1799	cUMR	0.3721	0.000000000146	0.00000000112	Hypermethylated	0.3748	0.000000000702	0.000000016	Hypermethylated	-0.021566667	0.395183106	0.447073963	Non	0.0836	0.004209961	0.012435848	Non	0.4506	0.0000000000419	0.00000000141	Hypermethylated	0.4216	0.000000000594	0.00000000477	Hypermethylated	0.0844	0.001304058	0.00242608	Non	0.237314286	0.00000000000408	0.0000000000263	Hypermethylated	RNF220	0
chr1	45097035	45098584	1549	cUMR	-0.0144	0.051755309	0.059786177	Non	0.0003	0.279032263	0.35179409	Non	0.004266667	0.190739664	0.236852829	Non	-0.0077	0.33557098	0.420803948	Non	-0.00565	0.531084001	0.576819832	Non	-0.01565	0.0813341	0.100091366	Non	-0.0045	0.729718487	0.751712054	Non	-0.005871429	0.155644373	0.160403631	Non	RNF220	0
chr1	45139292	45140891	1599	cUMR	-0.012844682	0.00000488	0.000012	Non	-0.008566667	0.034322645	0.063019871	Non	0.0428	0.0000000371	0.000000296	Non	-0.014966667	0.000674392	0.002846145	Non	-0.007116667	0.01135977	0.019862701	Non	-0.008366667	0.144859949	0.169466239	Non	-0.011966667	0.009397032	0.014586798	Non	-0.001704489	0.000000411	0.000000825	Non	TMEM53	0
chr1	45180520	45181324	804	cUMR	-0.101266667	0.00000198	0.00000537	Hypomethylated	-0.088833333	0.0000728	0.000329508	Non	-0.0496	0.000116108	0.000356067	Non	-0.048633333	0.010352275	0.025589202	Non	-0.001183333	0.003127642	0.00639704	Non	-0.059933333	0.026768798	0.036517096	Non	-0.046433333	0.012109761	0.018355464	Non	-0.059119048	0.000000442	0.000000882	Non	C1orf228	0
chr1	45195864	45197314	1450	cUMR	-0.115633333	0.0000000816	0.000000308	Hypomethylated	-0.062466667	0.000940402	0.003021045	Non	0.0417	0.122626772	0.16058363	Non	-0.090066667	0.0000756	0.00047904	Non	-0.018216667	0.001842176	0.004022905	Non	-0.084466667	0.000942245	0.001784941	Non	-0.063466667	0.003269006	0.005603612	Non	-0.055266667	0.000000287	0.000000592	Non	C1orf228	-4602
chr1	45204406	45206183	1777	cUMR	-0.038633333	0.00000000183	0.0000000105	Non	0.011966667	0.330816223	0.403384198	Non	-0.0003	0.002645998	0.005542604	Non	-0.051833333	0.0000000469	0.00000116	Non	-0.024383333	0.044326074	0.065251664	Non	-0.020633333	0.01541743	0.022135917	Non	-0.029433333	0.000157485	0.000357625	Non	-0.021719048	0.00000000824	0.0000000234	Non	KIF2C	0
chr1	45240407	45242606	2199	cUMR	-0.068166667	0.0000104	0.000024	Non	-0.053633333	0.002656171	0.007339267	Non	0.003833333	0.867802917	0.885139875	Non	-0.022433333	0.103730929	0.164660295	Non	-0.065583333	0.000438378	0.001151519	Non	-0.081833333	0.00000516	0.0000169	Non	-0.070633333	0.00000825	0.000025	Non	-0.048833333	0.00000015	0.000000327	Non	RPS8	0
chr1	45249389	45250638	1249	cUMR	0.0273	0.000000000323	0.00000000225	Non	0.1663	0.00000000507	0.0000000845	Hypermethylated	0.1283	0.000000203	0.00000132	Hypermethylated	-0.0375	0.117668549	0.182036124	Non	0.19255	0.000000000573	0.0000000114	Hypermethylated	0.0988	0.00000277	0.00000968	Non	0.1155	0.0000000126	0.0000000777	Hypermethylated	0.094871429	0.00000000000102	0.00000000000779	Non	BEST4	0
chr1	45251332	45252424	1092	cUMR	0.192333333	0.0000000376	0.000000154	Hypermethylated	0.295133333	0.0000000414	0.000000496	Hypermethylated	0.367	0.0000000000497	0.000000000848	Hypermethylated	-0.043266667	0.003006601	0.009479664	Non	0.278833333	0.0000000173	0.000000185	Hypermethylated	-0.002916667	0.875981919	0.884645557	Non	0.171533333	0.0000000348	0.00000019	Hypermethylated	0.187904762	0.0000000000129	0.0000000000718	Hypermethylated	BEST4	0
chr1	45265145	45266844	1699	cUMR	-0.034133333	0.0000622	0.000120419	Non	-0.0153	0.21488009	0.285177372	Non	0.0312	0.000495983	0.001270293	Non	-0.0109	0.481674245	0.560012831	Non	-0.01005	0.57678278	0.619702681	Non	-0.01355	0.168556353	0.194552687	Non	0.0423	0.001382324	0.002557274	Non	-0.0009	0.000412356	0.000526824	Non	PLK3	0
chr1	45284602	45286372	1770	cUMR	-0.062066667	0.00000285	0.00000742	Non	0.0215	0.084773566	0.13311538	Non	0.344766667	0.0000000000000823	0.00000000000323	Hypermethylated	-0.0627	0.000909128	0.003630184	Non	-0.01915	0.343338585	0.392922696	Non	-0.03515	0.033424144	0.044678002	Non	0.0149	0.001366497	0.002530857	Non	0.0385	0.00000000109	0.00000000378	Non	PTCH2	0
chr1	45301514	45301913	399	cUMR	-0.035133333	0.00053837	0.000863297	Non	0.0319	0.071857097	0.11631225	Non	0.051366667	0.107600326	0.143035782	Non	-0.0817	0.00000258	0.0000303	Non	0.00795	0.042301474	0.062715696	Non	0.0027	0.034415133	0.045883037	Non	-0.0139	0.0000000709	0.000000358	Non	-0.0051	0.000000203	0.000000431	Non	PTCH2	0
chr1	45308208	45309357	1149	cUMR	-0.0594	0.0000578	0.000112502	Non	0.0851	0.0000612	0.000283038	Non	0.012766667	0.436396047	0.486684188	Non	-0.0389	0.05894941	0.104738102	Non	0.12385	0.00000000147	0.0000000245	Hypermethylated	0.03335	0.003604025	0.005969879	Non	-0.0105	0.175881807	0.20793092	Non	0.015071429	0.000000142	0.000000311	Non	PTCH2	0
chr1	45451023	45452822	1799	cUMR	-0.064566667	0.00000000048	0.00000000321	Non	-0.022233333	0.112674579	0.167703479	Non	-0.0099	0.421675946	0.472960063	Non	-0.050233333	0.00018651	0.00099723	Non	-0.037733333	0.00000717	0.0000319	Non	-0.016483333	0.285553768	0.315466681	Non	-0.021233333	0.140569173	0.169756668	Non	-0.032347619	0.000000068	0.000000159	Non	EIF2B3	0
chr1	45475808	45478852	3044	cUMR	-0.012466667	0.008102908	0.010568514	Non	0.0221	0.021281067	0.042402006	Non	0.0037	0.181326605	0.226533699	Non	0.0103	0.032706837	0.064982222	Non	-0.01855	0.017941767	0.029664543	Non	-0.0088	0.462079295	0.490342456	Non	0.0153	0.007313979	0.011638225	Non	0.002185714	0.00532906	0.006081084	Non	HECTD3	0
chr1	45670420	45672884	2464	cUMR	0.003463808	0.046306386	0.053876559	Non	0.028733333	0.0000152	0.0000834	Non	-0.013376882	0.002091564	0.004505402	Non	-0.004066667	0.346233273	0.431034037	Non	0.024433333	0.017621714	0.029203497	Non	0.005683333	0.719011551	0.736305472	Non	0.007333333	0.479835893	0.514577923	Non	0.006312633	0.011677281	0.012950394	Non	ZSWIM5	0
chr1	45768445	45770644	2199	cUMR	-0.068366667	0.00000000102	0.00000000623	Non	-0.030633333	0.037671839	0.067916976	Non	-0.0332	0.00026698	0.000738654	Non	-0.036233333	0.007063796	0.01880921	Non	-0.046033333	0.001145028	0.002657171	Non	-0.046783333	0.00169337	0.003027887	Non	-0.054633333	0.0000111	0.0000327	Non	-0.04537619	0.00000000236	0.00000000758	Non	LINC01144	0
chr1	45791778	45793227	1449	cUMR	0.175733333	0.0000000000066	0.0000000000722	Hypermethylated	0.277966667	0.0000000000654	0.00000000239	Hypermethylated	0.393066667	0.000000000000000666	0.0000000000000569	Hypermethylated	0.051966667	0.0000581	0.000386525	Non	0.037816667	0.014258477	0.024243335	Non	0.030566667	0.002684234	0.00458724	Non	0.378966667	0.000000000000235	0.00000000000719	Hypermethylated	0.206595238	0.0000000000000134	0.000000000000184	Hypermethylated	HPDL	0
chr1	45804726	45807175	2449	cUMR	-0.043666667	0.000235647	0.000404951	Non	-0.017933333	0.350990318	0.423123402	Non	0.005	0.005342847	0.010249318	Non	-0.001333333	0.263585448	0.347382124	Non	-0.024833333	0.126663256	0.1636902	Non	-0.065083333	0.000000354	0.00000153	Non	-0.007133333	0.096412629	0.120614647	Non	-0.02067619	0.0000601	0.0000854	Non	MUTYH	0
chr1	45955251	45957620	2369	cUMR	-0.069533333	0.000000119	0.000000429	Non	-0.0502	0.0000421	0.000203694	Non	0.0503	0.000751265	0.001826448	Non	-0.0308	0.055558965	0.099937295	Non	0.0123	0.002180264	0.004658627	Non	0.00355	0.978225045	0.980136674	Non	-0.0742	0.000000186	0.000000842	Non	-0.023657143	0.00000345	0.00000589	Non	TESK2	0
chr1	45964604	45967353	2749	cUMR	-0.071866667	0.00000104	0.00000302	Non	-0.015633333	0.21095489	0.280927327	Non	0.114633333	0.00000094	0.00000506	Hypermethylated	-0.017033333	0.498795696	0.575168857	Non	-0.028283333	0.046686779	0.068251516	Non	-0.083283333	0.00000163	0.00000602	Non	-0.048033333	0.002586816	0.00452369	Non	-0.016947619	0.00000106	0.00000197	Non	CCDC163P	0
chr1	45986160	45988459	2299	cUMR	-0.023133333	0.111599509	0.123618706	Non	-0.0126	0.056588253	0.095390495	Non	0.149366667	0.00000000342	0.0000000365	Hypermethylated	-0.0078	0.387617974	0.471068533	Non	-0.0348	0.011483058	0.020054988	Non	-0.01055	0.14740674	0.172253597	Non	-0.0352	0.008886315	0.013875399	Non	0.008514286	0.0000625	0.0000886	Non	PRDX1	0
chr1	46015848	46018125	2277	cUMR	-0.040866667	0.0000877	0.000164408	Non	-0.057566667	0.00162224	0.004815422	Non	-0.0487	0.0000297	0.000107357	Non	-0.013966667	0.384707535	0.468500211	Non	-0.053366667	0.0000456	0.000159981	Non	-0.006366667	0.008333132	0.012695146	Non	-0.020366667	0.004593466	0.007622351	Non	-0.035309524	0.0000021	0.00000372	Non	AKR1A1	0
chr1	46035771	46036270	499	cUMR	-0.0025	0.984390456	0.984962681	Non	0.0062	0.824746095	0.854779512	Non	-0.007333333	0.831962366	0.852661468	Non	-0.000567903	0.0001322	0.000753042	Non	0.04475	0.282263107	0.331076971	Non	-0.057452829	0.000134256	0.000311565	Non	0.1176	0.000783294	0.001525561	Non	0.014465532	0.00021166	0.000280206	Non	AKR1A1	-49
chr1	46048692	46052491	3799	Canyon	-0.039333333	0.00000366	0.0000093	Non	-0.012833333	0.214411333	0.284570934	Non	-0.020166667	0.001528015	0.003424356	Non	-0.025033333	0.013857102	0.032354273	Non	-0.038333333	0.000395569	0.001052028	Non	-0.051333333	0.00000105	0.00000406	Non	-0.053233333	0.0000000636	0.000000325	Non	-0.033461905	0.00000000627	0.0000000182	Non	NASP	0
chr1	46149907	46154756	4849	Canyon	-0.021966667	0.0000779	0.000147612	Non	-0.015266667	0.018677801	0.037994172	Non	0.004533333	0.074176674	0.103306334	Non	-0.003466667	0.611252536	0.674819663	Non	-0.014216667	0.018837009	0.030943029	Non	-0.023716667	0.00000247	0.00000873	Non	-0.021466667	0.000285188	0.00060955	Non	-0.013066667	0.0000285	0.0000425	Non	TMEM69	0
chr1	46215531	46216877	1346	cUMR	-0.057066667	0.000000000801	0.00000000504	Non	-0.028166667	0.003768297	0.009918585	Non	0.001766667	0.491513251	0.539931911	Non	-0.026366667	0.028683177	0.058463531	Non	-0.032066667	0.000107137	0.000335427	Non	-0.037566667	0.0000268	0.0000735	Non	-0.028766667	0.006168581	0.009978392	Non	-0.029338095	0.0000000626	0.000000147	Non	IPP	0
chr1	46268624	46270773	2149	cUMR	-0.043266667	0.036352439	0.042858011	Non	-0.0121	0.461508112	0.53088414	Non	-0.027433333	0.052373887	0.076165127	Non	-0.0195	0.137801217	0.206231135	Non	-0.0441	0.097380734	0.130031636	Non	-0.0676	0.002196323	0.003826141	Non	0.0003	0.498799536	0.532878458	Non	-0.029357143	0.008602724	0.009641356	Non	MAST2	0
chr1	46502608	46503090	482	cUMR	-0.0552	0.002332896	0.003336189	Non	-0.037066667	0.305003095	0.378049461	Non	-0.076866667	0.005989837	0.011342863	Non	-0.030666667	0.502544452	0.578804005	Non	-0.020366667	0.349875638	0.39949316	Non	-0.045116667	0.161260217	0.1868652	Non	0.089733333	0.0000719	0.000176546	Non	-0.026980952	0.000833684	0.001027786	Non	MAST2	-813
chr1	46595209	46601558	6349	Canyon	-0.046066667	0.00000577	0.000014	Non	-0.034966667	0.000429198	0.001542027	Non	-0.0324	0.000485025	0.00124607	Non	-0.002566667	0.957293471	0.96622676	Non	-0.037566667	0.001986315	0.004294861	Non	-0.012316667	0.457229999	0.485560044	Non	-0.029166667	0.000000109	0.000000524	Non	-0.028680952	0.000000254	0.000000529	Non	PIK3R3	0
chr1	46631976	46633153	1177	cUMR	0.0633	0.000125584	0.000227645	Non	0.2669	0.000000000706	0.0000000161	Hypermethylated	0.260633333	0.000000000567	0.00000000725	Hypermethylated	-0.1537	0.0000000122	0.0000004	Hypomethylated	0.1728	0.00000000491	0.0000000657	Hypermethylated	0.0973	0.000349298	0.000731756	Non	-0.0063	0.00000000475	0.0000000327	Non	0.101671429	0.00000000000129	0.00000000000961	Hypermethylated	TSPAN1	7596
chr1	46663394	46664693	1299	cUMR	-0.051333333	0.0000557	0.000108935	Non	-0.028433333	0.035426538	0.064677118	Non	0.0205	0.385351725	0.437162502	Non	-0.020033333	0.313139149	0.398228255	Non	-0.041333333	0.002141439	0.004587466	Non	-0.043583333	0.0099754	0.014949452	Non	-0.020033333	0.066027315	0.085620179	Non	-0.02477619	0.000391554	0.0005017	Non	POMGNT1	0
chr1	46668323	46670222	1899	cUMR	-0.018966667	0.856844902	0.861133057	Non	0.2471	0.000654151	0.002208334	Non	-0.0598	0.329550544	0.382042667	Non	0.1753	0.012287542	0.029308495	Non	0.3532	0.0000179	0.000071	Hypermethylated	0.24545	0.001878469	0.003327166	Non	0.1873	0.002891018	0.00500697	Non	0.142014286	0.0000596	0.0000848	Hypermethylated	LURAP1	0
chr1	46689005	46689604	599	cUMR	-0.201566667	0.0000000000356	0.00000000032	Hypomethylated	-0.139966667	0.000029	0.000146704	Hypomethylated	-0.0694	0.004278495	0.008430863	Non	-0.137966667	0.00000233	0.0000278	Hypomethylated	-0.146066667	0.0000673	0.000223651	Hypomethylated	-0.112566667	0.003628356	0.006007704	Non	-0.043566667	0.0000859	0.000207244	Non	-0.121938095	0.000000000524	0.00000000196	Hypomethylated	LURAP1	-2078
chr1	46712122	46714055	1933	cUMR	-0.024933333	0.0000000102	0.0000000482	Non	0.0146	0.000486166	0.00171248	Non	-0.009766667	0.040213003	0.060463777	Non	-0.0158	0.000887266	0.003562961	Non	-0.00635	0.376307826	0.426000188	Non	-0.0231	0.00000472	0.0000156	Non	-0.0114	0.022597283	0.032314785	Non	-0.011114286	0.000000204	0.000000433	Non	RAD54L	0
chr1	46766431	46771184	4753	Canyon	-0.071666667	0.000000000344	0.00000000238	Non	-0.0061	0.841866946	0.869469732	Non	-0.018833333	0.021588949	0.035066762	Non	0.1019	0.0000000000224	0.0000000037	Hypermethylated	-0.05825	0.000000157	0.0000012	Non	-0.076	0.00000000582	0.0000000372	Non	-0.0513	0.000000000186	0.00000000198	Non	-0.0245	0.00000000000189	0.0000000000134	Non	LRRC41	0
chr1	46804954	46807302	2348	cUMR	-0.060633333	0.00099294	0.001517206	Non	-0.015166667	0.599665989	0.656496812	Non	0.026033333	0.031784648	0.049152696	Non	-0.024366667	0.411989496	0.494166421	Non	-0.056966667	0.022035095	0.035466318	Non	-0.016466667	0.571948127	0.596116343	Non	-0.056566667	0.009073634	0.014140363	Non	-0.028052381	0.003917142	0.004521064	Non	NSUN4	0
chr1	46859456	46860905	1449	cUMR	-0.0206	0.001861958	0.002708855	Non	0.010033333	0.742079799	0.783503348	Non	0.0609	0.010476579	0.018522715	Non	-0.056766667	0.123099209	0.188513265	Non	0.081733333	0.002317297	0.004920886	Non	-0.070516667	0.002136543	0.003733237	Non	-0.035766667	0.05433186	0.071767377	Non	-0.003595238	0.00067316	0.000838553	Non	FAAH	0
chr1	46907792	46908841	1049	cUMR	-0.056266667	0.0000396	0.0000798	Non	-0.032833333	0.089000981	0.138472148	Non	0.103733333	0.00000751	0.0000318	Hypermethylated	-0.011833333	0.767112553	0.809541419	Non	0.199816667	0.0000000014	0.0000000235	Hypermethylated	0.011066667	0.102157823	0.123367538	Non	0.071966667	0.000149486	0.000341424	Non	0.036138095	0.0000000388	0.0000000953	Non	FAAHP1	0
chr1	46912194	46912843	649	cUMR	-0.074466667	0.001443386	0.00214166	Non	0.1085	0.000511968	0.001793412	Non	-0.000633333	0.005226978	0.010050244	Non	-0.0371	0.114466285	0.178099724	Non	-0.0638	0.000878535	0.002112235	Non	-0.00105	0.957611557	0.960881217	Non	-0.0143	0.067876902	0.087824831	Non	-0.012128571	0.000219714	0.000290198	Non	LINC01398	0
chr1	46931910	46932909	999	cUMR	0.006733333	0.027031562	0.032500453	Non	0.1426	0.000000000516	0.0000000125	Hypermethylated	0.140733333	0.000000008	0.0000000777	Hypermethylated	-0.0114	0.293631265	0.378371542	Non	0.17965	0.0000000000018	0.00000000012	Hypermethylated	0.1904	0.000000000642	0.00000000511	Hypermethylated	0.082	0.00000344	0.0000114	Non	0.098028571	0.00000000000101	0.00000000000772	Non	LINC01398	-16535
chr1	46943102	46943568	466	cUMR	-0.039233333	0.000635539	0.001006653	Non	-0.025566667	0.534954418	0.598097555	Non	-0.018233333	0.356751846	0.409168464	Non	-0.047566667	0.069389357	0.119541373	Non	-0.003566667	0.808811189	0.832225963	Non	-0.033566667	0.10368915	0.125028828	Non	0.024433333	0.109153609	0.134984109	Non	-0.021052381	0.007517818	0.008468396	Non	LINC01398	-27727
chr1	46950944	46951843	899	cUMR	0.2372	0.00000364	0.00000924	Hypermethylated	0.389033333	0.00000000142	0.0000000286	Hypermethylated	0.230533333	0.000000969	0.0000052	Hypermethylated	0.315033333	0.000000404	0.00000667	Hypermethylated	0.239033333	0.0000159	0.000064	Hypermethylated	0.484033333	0.000000000355	0.00000000302	Hypermethylated	0.370833333	0.0000000187	0.00000011	Hypermethylated	0.31637619	0.000000000000529	0.00000000000441	Hypermethylated	DMBX1	20825
chr1	46953602	46959051	5449	Canyon	0.156066667	0.0000175	0.0000382	Hypermethylated	0.249566667	0.00000000597	0.0000000966	Hypermethylated	0.062233333	0.024699874	0.039441891	Non	0.121966667	0.000360346	0.001709709	Non	0.243816667	0.000000706	0.00000431	Hypermethylated	0.251316667	0.0000000124	0.0000000738	Hypermethylated	0.210166667	0.00000127	0.00000467	Hypermethylated	0.177109524	0.00000000000844	0.0000000000494	Hypermethylated	DMBX1	13617
chr1	46993869	46994168	299	cUMR	-0.129466667	0.0000561	0.000109658	Hypomethylated	-0.095366667	0.001203691	0.003716728	Non	0.0522	0.221127681	0.269823022	Non	-0.065366667	0.109539477	0.171887889	Non	-0.119686888	0.0000000000108	0.000000000483	Hypomethylated	-0.065466667	0.139115702	0.16332506	Non	0.015433333	0.000561661	0.001130642	Non	-0.055163099	0.0000000232	0.0000000598	Non	MKNK1-AS1	10200
chr1	46998014	46999413	1399	cUMR	-0.010933333	0.008392785	0.01092059	Non	0.057	0.0000000000874	0.00000000304	Non	-0.001766667	0.142030747	0.182775656	Non	-0.0092	0.02275756	0.048587905	Non	0.0044	0.002783992	0.005787495	Non	0.10915	0.000000000000233	0.00000000000533	Hypermethylated	0.1162	0.000000000000000222	0.000000000000026	Hypermethylated	0.034228571	0.0000000000000137	0.000000000000186	Non	MKNK1-AS1	4955
chr1	47009357	47010256	899	cUMR	0.032733333	0.458794362	0.472904167	Non	0.438066667	0.00000000262	0.0000000485	Hypermethylated	0.2979	0.000000755	0.00000418	Hypermethylated	0.043466667	0.495830147	0.572438791	Non	0.348816667	0.000000177	0.00000134	Hypermethylated	0.512566667	0.000000000927	0.00000000708	Hypermethylated	0.486466667	0.00000000182	0.0000000142	Hypermethylated	0.293409524	0.0000000000305	0.000000000153	Hypermethylated	MKNK1-AS1	0
chr1	47069482	47070296	814	cUMR	-0.008794058	0.00024431	0.000417959	Non	-0.005266667	0.179998528	0.246136904	Non	0.151466667	0.0000000000491	0.000000000839	Hypermethylated	-0.006239401	0.102422794	0.162884213	Non	-0.005325799	0.209244048	0.25411294	Non	-0.006841199	0.0789643	0.097473707	Non	-0.006774489	0.009533963	0.01477841	Non	0.020453384	0.0000000226	0.0000000583	Non	MKNK1	0
chr1	47081524	47082973	1449	cUMR	-0.027533333	0.019540878	0.023994704	Non	0.0065	0.329297141	0.401796202	Non	0.0658	0.002426314	0.005132489	Non	-0.0027	0.588558568	0.65498681	Non	0.0073	0.732753531	0.763716734	Non	-0.01495	0.285414109	0.315326848	Non	0.0895	0.0000404	0.000105037	Non	0.019014286	0.003817689	0.00441156	Non	MOB3C	0
chr1	47132520	47134419	1899	cUMR	-0.0034	0.340096111	0.355312412	Non	-0.005833333	0.683754837	0.73222115	Non	-0.009566667	0.338649915	0.391388092	Non	-0.004633333	0.513956628	0.588988005	Non	0.005266667	0.737590848	0.768293247	Non	-0.012233333	0.200221452	0.227870811	Non	0.020166667	0.117154059	0.143951524	Non	-0.001633333	0.27543181	0.280756134	Non	ATPAF1	0
chr1	47183409	47185858	2449	cUMR	-0.0442	0.00000715	0.000017	Non	-0.0046	0.816369499	0.847531594	Non	-0.0057	0.582755828	0.626343604	Non	-0.0312	0.000464122	0.002110897	Non	-0.0252	0.007835613	0.014316673	Non	-0.0477	0.0000488	0.000125597	Non	-0.0266	0.001613656	0.002934539	Non	-0.0258	0.00000156	0.00000283	Non	EFCAB14	0
chr1	47190655	47191654	999	cUMR	-0.122	0.0000631	0.000122044	Hypomethylated	-0.0731	0.008330886	0.019360532	Non	0.003	0.696143536	0.731093303	Non	-0.0541	0.079329808	0.132820796	Non	0.0075	0.698157412	0.732665476	Non	-0.11	0.002860881	0.00484889	Non	-0.0175	0.042417373	0.05731126	Non	-0.052785714	0.000686383	0.000854009	Non	EFCAB14	-5920
chr1	47640803	47641502	699	cUMR	-0.0886	0.0000039	0.00000983	Non	-0.0828	0.000076	0.00034213	Non	-0.104933333	0.0000000245	0.000000206	Hypomethylated	-0.0898	0.0000132	0.000114856	Non	-0.0746	0.00123778	0.002840493	Non	-0.0621	0.008670805	0.013163804	Non	-0.0664	0.001573614	0.00287078	Non	-0.082942857	0.00000000000532	0.0000000000331	Non	LINC00853	3420
chr1	47644401	47646450	2049	cUMR	-0.105433333	0.000000115	0.000000418	Hypomethylated	-0.028033333	0.423022478	0.494569534	Non	-0.058266667	0.0000909	0.00028753	Non	-0.092033333	0.000229914	0.001184369	Non	-0.050183333	0.106442316	0.140673847	Non	-0.058933333	0.029956696	0.040412578	Non	0.007166667	0.666498609	0.692926198	Non	-0.056661905	0.00000118	0.00000219	Non	LINC00853	0
chr1	47658370	47659022	652	cUMR	0.040333333	0.121660884	0.134079575	Non	0.0711	0.008006162	0.018705109	Non	-0.082166667	0.000000815	0.00000446	Non	-0.0105	0.580374624	0.647883804	Non	0.04825	0.119326415	0.155417141	Non	-0.04975	0.038569192	0.050861355	Non	0.0477	0.12389423	0.151440946	Non	0.008128571	0.006988032	0.007896351	Non	PDZK1IP1	-2600
chr1	47689756	47692410	2654	cUMR	0.005533333	0.828581061	0.834084398	Non	0.256233333	0.0000000148	0.000000207	Hypermethylated	0.0167	0.057866156	0.083151976	Non	0.027433333	0.292653331	0.377292959	Non	0.225283333	0.000000363	0.00000244	Hypermethylated	0.366783333	0.000000000317	0.00000000273	Hypermethylated	0.137433333	0.0000053	0.0000168	Hypermethylated	0.131633333	0.00000000316	0.00000000981	Hypermethylated	TAL1	0
chr1	47694920	47698519	3599	Canyon	0.274766667	0.00000000469	0.0000000242	Hypermethylated	0.367533333	0.000000000044	0.00000000171	Hypermethylated	0.158933333	0.0000127	0.0000504	Hypermethylated	0.144133333	0.000114582	0.000670192	Hypermethylated	0.404183333	0.000000000141	0.00000000374	Hypermethylated	0.347683333	0.0000000027	0.0000000185	Hypermethylated	0.356533333	0.000000000215	0.00000000224	Hypermethylated	0.284304762	0.000000000000000555	0.0000000000000122	Hypermethylated	TAL1	0
chr1	47778528	47780587	2059	cUMR	-0.035533333	0.00000953	0.0000221	Non	-0.0102	0.343513764	0.415628197	Non	-0.004033333	0.062202968	0.088573441	Non	-0.016	0.114854838	0.178612021	Non	-0.02395	0.020673294	0.033559114	Non	-0.0287	0.002856423	0.004841675	Non	-0.038	0.00000633	0.0000197	Non	-0.021971429	0.00000289	0.000005	Non	STIL	0
chr1	47798439	47800789	2350	cUMR	-0.0453	0.0000000000852	0.000000000702	Non	-0.036733333	0.00000262	0.0000178	Non	-0.0018	0.014222006	0.024227478	Non	-0.021533333	0.004580378	0.01331941	Non	-0.009383333	0.175579663	0.217919176	Non	-0.023883333	0.001886987	0.003341117	Non	-0.029533333	0.00000448	0.0000145	Non	-0.024419048	0.00000000179	0.0000000059	Non	CMPK1	0
chr1	47881817	47883257	1440	cUMR	0.064433333	0.003576225	0.004955429	Non	0.474466667	0.000000000000236	0.0000000000331	Hypermethylated	0.175266667	0.00000147	0.00000751	Hypermethylated	0.083066667	0.00178708	0.006268566	Non	0.123016667	0.000864284	0.002084002	Non	0.440266667	0.0000000000134	0.000000000171	Hypermethylated	0.307466667	0.000000000494	0.00000000461	Hypermethylated	0.229038095	0.000000000000634	0.00000000000516	Hypermethylated	FOXE3	0
chr1	47889343	47890242	899	cUMR	-0.1283	0.0000288	0.0000599	Hypomethylated	-0.067366667	0.047180867	0.081916374	Non	-0.019279414	0.00000425	0.0000193	Non	-0.069566667	0.070550544	0.121149995	Non	-0.059716667	0.234075581	0.280508894	Non	-0.066966667	0.149031784	0.174008815	Non	-0.048766667	0.093259016	0.11702178	Non	-0.066305982	0.0000283	0.0000421	Non	FOXE3	-5620
chr1	47897776	47905470	7694	Canyon	0.121533333	0.005713809	0.007646971	Non	0.147866667	0.000589529	0.002021612	Non	0.0077	0.960604711	0.966177734	Non	0.207666667	0.0000193	0.000156682	Hypermethylated	0.280116667	0.0000127	0.0000525	Hypermethylated	0.181366667	0.000990792	0.00186751	Non	0.183466667	0.001912924	0.003424503	Non	0.151895238	0.000000399	0.000000802	Hypermethylated	FOXD2-AS1	0
chr1	47973495	47975001	1506	cUMR	-0.0248	0.001010332	0.001541632	Non	0.036233333	0.02721255	0.052047053	Non	0.027533333	0.061166289	0.087305883	Non	0.004233333	0.720858592	0.770035686	Non	0.003783333	0.008191879	0.014877375	Non	-0.026716667	0.039330731	0.051774935	Non	0.089233333	0.0000000214	0.000000124	Non	0.01637619	0.00000326	0.00000558	Non	FOXD2	-67133
chr1	48173623	48176972	3349	cUMR	-0.035533333	0.04795129	0.055674786	Non	0.0533	0.008699023	0.020107468	Non	0.0858	0.000240054	0.000673437	Non	0.0021	0.995186307	0.996550992	Non	0.01805	0.290383306	0.339576693	Non	0.07105	0.019416041	0.027248719	Non	0.0629	0.016893132	0.024852366	Non	0.0357	0.006064458	0.006888259	Non	TRABD2B	49228
chr1	48190103	48192602	2499	cUMR	-0.128866667	0.000000179	0.000000619	Hypomethylated	0.0443	0.04605407	0.080254911	Non	-0.023866667	0.299959311	0.352194955	Non	-0.0685	0.021667028	0.046666014	Non	0.0403	0.00000367	0.0000178	Non	0.0918	0.013629414	0.019790409	Non	-0.0285	0.010674118	0.016368838	Non	-0.018614286	0.00000579	0.00000955	Non	TRABD2B	33598
chr1	48204302	48204801	499	cUMR	-0.166666667	0.000000315	0.00000103	Hypomethylated	0.004633333	0.980457683	0.983805488	Non	-0.1265	0.0000306	0.000110526	Hypomethylated	-0.118366667	0.001222678	0.004626171	Non	0.216333333	0.00000427	0.0000203	Hypermethylated	-0.021416667	0.508175114	0.53532103	Non	0.000633333	0.001681245	0.003043216	Non	-0.044138095	0.00000121	0.00000224	Non	TRABD2B	21399
chr1	48461787	48463686	1899	cUMR	-0.0156	0.165889588	0.179845569	Non	0.092066667	0.00000385	0.0000249	Non	0.0279	0.058943191	0.084523237	Non	-0.011333333	0.110737956	0.173407191	Non	0.010316667	0.562532948	0.6065288129999999	Non	0.035316667	0.028535955	0.038684713	Non	-0.009133333	0.609082505	0.638328637	Non	0.017552381	0.004833879	0.005533553	Non	TRABD2B	0
chr1	48574878	48575485	607	cUMR	-0.1609	0.00000103	0.00000297	Hypomethylated	-0.1308	0.000231031	0.000897493	Hypomethylated	-0.0994	0.004862173	0.009430072	Non	-0.102	0.002312616	0.007708079	Non	-0.0714	0.000369457	0.000990012	Non	-0.11415	0.005827554	0.009208681	Non	-0.0538	0.003508717	0.005972416	Non	-0.106771429	0.000000123	0.000000273	Hypomethylated	SKINTL	0
chr1	48935431	48938712	3281	cUMR	-0.0112	0.744680976	0.753024303	Non	0.0025	0.247078031	0.318728015	Non	0.0268	0.023370461	0.03761748	Non	0.0019	0.622023435	0.683984519	Non	0.0143	0.746447621	0.776243679	Non	-0.0247	0.162146374	0.187783704	Non	0.0413	0.157708011	0.188218535	Non	0.008014286	0.270711894	0.276050068	Non	SPATA6	0
chr1	48963832	48964577	745	cUMR	-0.200033333	0.000000000616	0.000000004	Hypomethylated	-0.136733333	0.000108135	0.000465726	Hypomethylated	0.228633333	0.00000123	0.00000642	Hypermethylated	-0.132133333	0.000178924	0.00096136	Hypomethylated	0.005216667	0.0000621	0.000208495	Non	-0.147783333	0.000079	0.000193373	Hypomethylated	-0.043133333	0.000137707	0.00031747	Non	-0.055961905	0.00000000742	0.0000000213	Non	SPATA6	-25953
chr1	49142954	49143753	799	cUMR	-0.2329	0.000000441	0.00000139	Hypomethylated	-0.052533333	0.314888984	0.387968087	Non	0.030266667	0.350571793	0.403037979	Non	-0.114533333	0.022966127	0.048898898	Non	-0.088983333	0.0000179	0.0000711	Non	-0.127733333	0.002790148	0.004744689	Non	-0.053733333	0.052019812	0.069012245	Non	-0.091047619	0.00000414	0.00000698	Non	AGBL4	0
chr1	49162877	49162983	106	cUMR	-0.046669674	0.004817225	0.00652936	Non	-0.037739249	0.116692559	0.172606997	Non	0.013610532	0.158061176	0.201095941	Non	-0.02833666	0.043009346	0.081168394	Non	0.033431508	0.352638293	0.402114115	Non	-0.02457224	0.574484523	0.598552814	Non	0.039645672	0.074202913	0.095126271	Non	-0.008434105	0.004877687	0.005581051	Non	AGBL4	0
chr1	49241255	49243615	2360	cUMR	-0.0132	0.266409029	0.281501568	Non	0.100066667	0.00000458	0.000029	Hypermethylated	-0.031033333	0.000188477	0.000544431	Non	0.038066667	0.026143635	0.054301786	Non	0.040466667	0.037072351	0.055793749	Non	0.316216667	0.0000000000014	0.0000000000248	Hypermethylated	0.075666667	0.0000152	0.0000434	Non	0.06372381	0.00000000272	0.00000000858	Non	BEND5	0
chr1	50488969	50490518	1549	cUMR	-0.067766667	0.000256163	0.000436133	Non	-0.008166667	0.763576447	0.802088128	Non	0.016566667	0.001958021	0.004252062	Non	-0.042166667	0.05448881	0.098409005	Non	-0.032766667	0.026154401	0.041177889	Non	0.429233333	0.000000000000915	0.0000000000171	Hypermethylated	0.128633333	0.000000536	0.00000217	Hypermethylated	0.047719048	0.000000000661	0.00000000241	Non	AGBL4	0
chr1	50513416	50514446	1030	cUMR	0.269833333	0.00000364	0.00000925	Hypermethylated	0.3189	0.000000627	0.00000514	Hypermethylated	0.293666667	0.0000000257	0.000000215	Hypermethylated	0.1791	0.000428059	0.001975232	Non	0.35175	0.0000000335	0.000000323	Hypermethylated	0.45575	0.00000000651	0.0000000411	Hypermethylated	0.6279	0.000000000000331	0.00000000000952	Hypermethylated	0.349728571	0.000000000000115	0.00000000000117	Hypermethylated	ELAVL4	0
chr1	50574470	50577019	2549	cUMR	-0.139833333	0.000103502	0.000191008	Hypomethylated	0.286966667	0.00000123	0.00000925	Hypermethylated	-0.146333333	0.00019221	0.000553588	Hypomethylated	0.061566667	0.153249657	0.224715329	Non	0.055666667	0.087500399	0.118419892	Non	0.097166667	0.042675733	0.055718688	Non	0.092366667	0.073958879	0.094861285	Non	0.031395238	0.0000322	0.0000476	Non	ELAVL4	0
chr1	50717035	50718434	1399	cUMR	-0.118333333	0.00000884	0.0000206	Hypomethylated	0.3212	0.000000000397	0.0000000101	Hypermethylated	-0.127166667	0.00000314	0.0000148	Hypomethylated	0.1486	0.00000616	0.0000611	Hypermethylated	0.0215	0.016406743	0.027446699	Non	0.21775	0.00000449	0.0000149	Hypermethylated	-0.0614	0.022089289	0.031657855	Non	0.0436	0.00000000349	0.0000000107	Non	ELAVL4	-47579
chr1	50744580	50745979	1399	cUMR	-0.159933333	0.000000903	0.00000265	Hypomethylated	-0.112133333	0.005476651	0.013588668	Non	0.144733333	0.00000315	0.0000148	Hypermethylated	-0.069933333	0.00020272	0.001065693	Non	-0.112683333	0.000963426	0.002285553	Non	-0.007683333	0.030074201	0.040546497	Non	-0.121333333	0.000267957	0.000576337	Hypomethylated	-0.059704762	0.000000078	0.00000018	Non	ELAVL4	-75124
chr1	50798510	50799676	1166	cUMR	-0.035666667	0.0000269	0.0000563	Non	0.002333333	0.14091155	0.201186456	Non	0.399166667	0.000000000000000222	0.0000000000000218	Hypermethylated	-0.021866667	0.163009682	0.236117879	Non	0.144583333	0.0000000000146	0.000000000608	Hypermethylated	0.324833333	0.000000000000127	0.00000000000313	Hypermethylated	0.217333333	0.0000000000133	0.000000000209	Hypermethylated	0.144219048	0.00000000000000244	0.0000000000000428	Hypermethylated	DMRTA2	83547
chr1	50813910	50815109	1199	cUMR	-0.134233333	0.000271757	0.000459985	Hypomethylated	-0.027233333	0.129512182	0.187799678	Non	-0.066566667	0.045291716	0.067091208	Non	-0.167833333	0.0000556	0.000374675	Hypomethylated	-0.011233333	0.947065668	0.953269621	Non	-0.204233333	0.0000072	0.0000227	Hypomethylated	-0.161433333	0.000163289	0.000369309	Hypomethylated	-0.10997619	0.00000227	0.000004	Hypomethylated	DMRTA2	68114
chr1	50833759	50834758	999	cUMR	-0.0284	0.00000000000000688	0.000000000000164	Non	-0.048033333	0.002910715	0.007946018	Non	-0.022733333	0.000000493	0.00000287	Non	-0.061233333	0.001153507	0.004415059	Non	-0.022483333	0.00000000211	0.0000000326	Non	-0.014233333	0.00000165	0.00000607	Non	-0.065833333	0.00000037	0.00000155	Non	-0.03797619	0.000000000000198	0.00000000000187	Non	DMRTA2	48465
chr1	50880759	50894221	13462	Canyon	0.384933333	0.000000000276	0.00000000196	Hypermethylated	0.31	0.00000015	0.0000015	Hypermethylated	0.210933333	0.0000273	0.0000997	Hypermethylated	0.1744	0.000254552	0.001287075	Non	0.5166	0.00000000019	0.00000000476	Hypermethylated	0.50035	0.0000000000764	0.000000000779	Hypermethylated	0.3564	0.0000000731	0.000000368	Hypermethylated	0.338485714	0.000000000000000222	0.00000000000000546	Hypermethylated	DMRTA2	0
chr1	51424346	51426745	2399	cUMR	-0.0243	0.0000297	0.0000614	Non	-0.021033333	0.001326096	0.004040836	Non	-0.012466667	0.095864602	0.129267819	Non	-0.023233333	0.000445489	0.002042116	Non	-0.023133333	0.001524565	0.003407436	Non	-0.024383333	0.000612149	0.001211428	Non	-0.017633333	0.016065776	0.023752666	Non	-0.02057619	0.0000000125	0.0000000341	Non	FAF1	0
chr1	51432949	51437948	4999	Canyon	-0.015833333	0.001068308	0.001623721	Non	-0.0057	0.551998904	0.614076801	Non	-0.0175	0.00000551	0.0000242	Non	0.0061	0.354367801	0.439187167	Non	-0.016	0.047309108	0.069018942	Non	-0.00625	0.223004896	0.251178872	Non	0.0307	0.002101127	0.003731208	Non	-0.003814286	0.000067	0.0000945	Non	CDKN2C	0
chr1	51442109	51444758	2649	cUMR	0.022133333	0.000756789	0.001180165	Non	0.106133333	0.000000106	0.00000111	Hypermethylated	0.051966667	0.000000168	0.00000111	Non	0.022133333	0.197207082	0.275033062	Non	0.084383333	0.000161238	0.000479057	Non	0.005883333	0.021185204	0.02953069	Non	0.109933333	0.000000793	0.00000308	Hypermethylated	0.057047619	0.0000000403	0.0000000988	Non	CDKN2C	-1801
chr1	51700488	51704164	3676	Canyon	-0.034733333	0.000306215	0.000513333	Non	-0.0184	0.152772779	0.214970257	Non	-0.0254	0.020361896	0.033298111	Non	-0.003	0.960363369	0.968724842	Non	-0.03865	0.000862523	0.00208038	Non	-0.01115	0.434789836	0.463650122	Non	-0.018	0.034109294	0.046957524	Non	-0.021628571	0.000143537	0.000194291	Non	RNF11	0
chr1	51762137	51763236	1099	cUMR	-0.1552	0.0000111	0.0000252	Hypomethylated	-0.179	0.000000897	0.00000702	Hypomethylated	-0.0357	0.056122699	0.080916884	Non	-0.1054	0.011164144	0.027156938	Non	-0.0937	0.015428034	0.026011668	Non	-0.05795	0.31094787	0.341093749	Non	-0.0022	0.965536925	0.969237533	Non	-0.091	0.0000106	0.0000168	Non	TTC39A	0
chr1	51794701	51797450	2749	cUMR	-0.083466667	0.000204274	0.000355268	Non	-0.0954	0.0000871	0.000385095	Non	0.053533333	0.121954375	0.159876628	Non	-0.0708	0.005236485	0.014840505	Non	-0.06555	0.029792735	0.046146377	Non	-0.08155	0.002323252	0.00402283	Non	-0.056	0.052071028	0.069068782	Non	-0.053685714	0.0000665	0.0000939	Non	TTC39A-AS1	0
chr1	51810053	51811052	999	cUMR	-0.0819	0.0000000121	0.0000000565	Non	-0.016366667	0.226980728	0.297906023	Non	0.2511	0.000000000000908	0.0000000000255	Hypermethylated	-0.038166667	0.006138274	0.016825464	Non	0.027933333	0.000662481	0.001654007	Non	0.010433333	0.019035028	0.026758648	Non	0.060233333	0.000206972	0.00045662	Non	0.034204762	0.000000000377	0.00000000145	Non	TTC39A	0
... (truncated at 1000 rows)
