# Supplementary material for: Homeobox oncogene activation by pan-cancer DNA hypermethylation
Source: Genome Biol. 2018 Aug 10;19:108. doi: 10.1186/s13059-018-1492-3 (PMC6085761; doi:10.1186/s13059-018-1492-3)
Supplement: Supplementary file 6 — Review history. (DOCX 2465 kb) [file 13059_2018_1492_MOESM6_ESM.docx]

**First round of review**

**Reviewer 1**

**Are the methods appropriate to the aims of the study, are they well described, and are necessary controls included?**

Yes

**Are the conclusions adequately supported by the data shown?**

Yes

**Are sufficient details provided to allow replication and comparison with related analyses that may have been performed?**

Yes

**Does the work represent a significant advance over previously published studies? Please explain.**

Yes

**Is the paper of broad interest to others in the field, or of outstanding interest to a broad audience of biologists?**

Yes: This paper reported an important observation of oncogene regulation

**Comments to authors**

In this paper, the authors revealed a very interesting phenomena - the hypermethylation of gene bodies is strongly associated with increased expression for homeobox oncogenes. This is fundamentally different from the well-known promoter hypermethylation leading to silence of tumor suppressor genes. This paper is the first that used large-scale unbiased analysis to point out this important association. The paper is well-written, and the analysis has been carefully designed, and the conclusion is well justified. In addition, they performed an experimental study to use dCas9-SunTag-DNMT3A system to methylate the gene body of DLX1, and validated that the expression of DLX1 was increased. Overall this is a very good paper. I have only a few 

(1) No deduplication was performed in the WGBS data processing. Doing so could reduce the number of reads covering each CpG, which could impact the results further on since a coverage threshold of 4 reads was applied.
(2) How do you determine the percentage of the reference UMRs that are covered by the CpG islands, etc. when the CpG island (e.g.) partially overlaps with the UMR? For each UMR, does it either overlap or not with a region, or can it overlap partly? Furthermore, the authors employed a Obs/Exp threshold (.1 or greater ,page 14 line 309) and then merged regions together to form UMRs. Since the Obs/Exp threshold of .1 is lower than the typical CpG island Obs/Exp threshold of .6, and since these regions were further merged together to be much longer, is it not surprising that there wasn't much overlap between CpG islands and the UMRs? Would this overlap change if the canyons were restricted to just hypermethylated canyons, since these have higher CpG density (page 7, line 144).
(3) The paper focused on the association between gene body hypermethylation and gene expression for homeobox genes. What about other genes? I believe that the association is not strong, but this should be explicitly explained in the paper, as most of readers would wonder. 

Minor critiques:
Figure 1c:
y-axis should say "Coverage proportion"

Page 16, line 359:
Some type of typo or missing word in the sentence "…we established two control groups of conserved canyons/cUMRs and conserved Canyons (including 1398 canyongs and 9597 and cUMRs)" ?
Was one group canyons/cUMRs and the other just canyons (so the first group includes the second)?

**Reviewer 2**

**Are the methods appropriate to the aims of the study, are they well described, and are necessary controls included?**

The methods are well-described but there are missing controls as outlined in my comments below.

**Are the conclusions adequately supported by the data shown?**

No. There are missing controls and currently the effect was only tested in one gene in one cell line. This needs to be improved as detailed in my comments to the authors.

**Are sufficient details provided to allow replication and comparison with related analyses that may have been performed?**

Yes.

**Does the work represent a significant advance over previously published studies? Please explain.**

Yes. As outlined below, the key finding is the experimental proof that DNA methylation of a gene body can increase expression. Other groups have observed that homeobox genes are hotspots for aberrant DNA methylation in cancer previously.

**Is the paper of broad interest to others in the field, or of outstanding interest to a broad audience of biologists?**

Yes: Positive correlations between gene body methylation and expression have been observed in many contexts. But whether methylation in this context had direct functional import has been an open question for the past decade.

**Comments to authors**

In this paper, the authors first use a pan-cancer analysis to describe blocks of DNA methylation changes (canyons) and find these canyons are enriched in m-homeobox genes. They then show that these events positively correlate with expression changes and then target methylation to the DLX1 gene to show that methylation of the gene body increases expression rather than decreases expression. There are quite a few studies suggesting that homeobox genes have a propensity to undergo hypermethylation in cancer, but the truly novel finding in this work is the manipulation of methylation in a gene body to activate expression. While this a potentially very interesting finding, as detailed below, I do not find the data from the targeting experiment convincing at this stage since there were missing controls for the targeted methylation experiment and only one gene in one cell line was tested. 

Major Concerns:
1) I did not understand why two different genes were chosen for in depth analysis (Fig. 4) and then for actual targeting with a dCas9-DNMT (Fig. 5). This suggests that HOX13B was targeted, but then did not show the desired effect. Does DLX1 show the same relationships between expression and methylation in tumor samples.
2) To demonstrate that hypermethylation of a canyon increases expression, I think the authors need to analyze more than one gene. Showing the effect in multiple cell lines would also make the result more robust.
3) For the expression analysis after dCas9-DNMT, there appeared to be essential controls missing, such as the recruitment of a dCas9-SunTag-DNMT with a mutant DNMT that has no methyltransferase activity. This is essential to control for any off-target effects produced by the system that occur independent of methylation.
4) The authors conclude from their CpG island analysis that their data suggest "that CpG density alone is not sufficient to capture the majority of epigenetic information in UMRs." CpG islands are a rather indirect measure of CpG density. CpG O/E measures in Fig. S1a would indicate that all canyons have high CpG density. 
5) I wish the authors would talk a little more about homeobox gene structure and how this relates to their results. Homeobox genes tend to have very high CpG content as illustrated by the multiple CpG islands found in HOXB13. I think the authors try to extrapolate their results (p. 12 line 258), but homeobox genes have a rather unique structure in terms of CpG density.
6) p.11, line 247. There are two sentences seem to say opposite things, or there is a missing subtlety that needs to be explained. I believe the authors are stating that methylation of the entire canyon or regions within the canyon (a) leads to no mechanistic difference but (b) determines the functional consequences. 
7) Do the authors have any thoughts as to why the canyon they detect at HOXB13 and DXL1 extend beyond the length of the gene? Is this something that effects transcription? Do they observe the same effect on expression if they target the canyon beyond the edge of the gene? This might facilitate the understanding of mechanism. 
8) The authors comments about DNMT3A overexpression should be set in greater context. Interpreting these results has been quite controversial since despite this observed increase in expression, overwhelmingly tumors lose much more methylation than they gain.
9) I wish the authors would bring more of the results from other tumor types into the main body of the paper. Upon my initial reading, I wrongly assumed the authors had only examined uterine cancer to extrapolate their canyon findings to additional tumors. Only after I delved into the supplement did I realize the tremendous amount of work buried there. I wish there were greater emphasis that the results observed in uterine cancer held true for other cancer-types as well. Possibly this could just be through mentions in the text such as these relationships held true for lung, breast, … cancers. Being more specific about the cancer types would also be more accurate than always saying "pan-cancer" and would also highlight the work involved better.


Minor Concerns:
1) There are many grammatical typos in the background section that should be fixed.
2) The authors state that ~90% of pan-cancer differential UMRs can be recurrently identified in at least 3 individuals. Is this due to the limitations to detect UMRs as defined or does this have some biological significance?
3) The sentence on p. 6, line 128-129 was very confusing to me as written (something about the combination of "ands", slashes, and parentheticals all intermixed). But understanding these 4 categories is crucial.
4) Minor issues with figures: Fig. 2a has no y-axis label, Fig. 3a is missing a scale bar, Fig. 3b has no y-axis label. In Fig. S5 it is unclear what n= means.
5) It is not clear what is plotted in Fig 3e. Is each gene averaged by individual, then the box plot consists of all genes, or is each individual averaged across all genes, and then the box plot consists of all individuals?
6) The authors should indicate the locations of the 450k probes used for the analyses in Fig. 4 in Fig. 4B.
7) In Fig. 2d and 5b I wish the authors would indicate the expression values across all the samples.
8) Fig. 5d is missing information about how many replicates were performed, and the meaning of error bars. No information was provided as to what type of replicates these were either.

**Authors’ response to reviewers**

Genome Biology GBIO-D-17-01564

We thank the editors and reviewers for carefully considering our manuscript. We have attached a point-by-point reply to the reviewers’ comments with our response in blue font. In addition, we have provided a version of the manuscript with any major sections of altered text indicated in red so the reviewers can easily identify the extensive changes we have made.

Reviewer #1:Remarks to the Author:

Reviewer #1: In this paper, the authors revealed a very interesting phenomena - the hypermethylation of gene bodies is strongly associated with increased expression for homeobox oncogenes. This is fundamentally different from the well-known promoter hypermethylation leading to silence of tumor suppressor genes. This paper is the first that used large-scale unbiased analysis to point out this important association. The paper is well-written, and the analysis has been carefully designed, and the conclusion is well justified. In addition, they performed an experimental study to use dCas9-SunTag-DNMT3A system to methylate the gene body of DLX1, and validated that the expression of DLX1 was increased. Overall this is a very good paper. I have only a few

Response: We appreciate the reviewer’s comment on the quality and novelty of our manuscript.

(1) No deduplication was performed in the WGBS data processing. Doing so could reduce the number of reads covering each CpG, which could impact the results further on since a coverage threshold of 4 reads was applied.

Response: WGBS PCR duplicated reads have been removed by BSMAP with default threshold. We have added corresponding description in the method section on page 15: “*we used BSMAP to trim adaptor, low-quality and duplicated sequence with default threshold*.”

We agree with the reviewer that read coverage could impact the accuracy of methylation detection. In previous study, Laurent et al. performed a saturation analysis of bisulfite sequencing and showed a high accuracy (79%) of CpG methylation detection with at least 4 covered reads [1]. Therefore, we used the coverage threshold of 4 reads to ensure the accuracy of CpG methylation detection in our study. We have added the corresponding description in the revised manuscript on page 15.

(2) How do you determine the percentage of the reference UMRs that are covered by the CpG islands, etc. when the CpG island (e.g.) partially overlaps with the UMR? For each UMR, does it either overlap or not with a region, or can it overlap partly?

Response: For each reference UMR, the percentage of UMR covered by the CpG islands is defined as the length of the UMR covered by CpG islands (either partially or entirely) divided by the total length of the UMR. We have added the definition to Fig 1c legend.

Furthermore, the authors employed a Obs/Exp threshold (.1 or greater ,page 14 line 309) and then merged regions together to form UMRs. Since the Obs/Exp threshold of .1 is lower than the typical CpG island Obs/Exp threshold of .6, and since these regions were further merged together to be much longer, is it not surprising that there wasn't much overlap between CpG islands and the UMRs?

Response: We agree with the reviewer that it is a little confusing to emphasize the difference between UMR and CpG island. Therefore, we have deleted the following sentence in the revised manuscript: “*suggesting that CpG islands alone are not sufficient to capture the majority of epigenetic information in UMRs*”

Would this overlap change if the canyons were restricted to just hypermethylated canyons, since these have higher CpG density (page 7, line 144).

Response: Hypermethylated canyons have similar overlap with CpG islands (Reviewer Fig 1) because Hypermethylated canyons have similar CpG density with conserved (majority) canyons. We have edited the following sentence in the revised manuscript: “*hypermethylated canyon/cUMRs have similar- or high- CpG density”.*

Reviewer Figure 1. Percentage of pan-cancer hypermethylated canyons covered by CpG islands (downloaded from UCSC).

(3) The paper focused on the association between gene body hypermethylation and gene expression for homeobox genes. What about other genes? I believe that the association is not strong, but this should be explicitly explained in the paper, as most of readers would wonder.

Response: We appreciate the reviewer’s comment and suggestion. In fact, in Figure 4e, we reported that these homeobox canyon genes exhibit much stronger positive correlation between gene-body methylation and expression than hypermethylated cUMR and differentially expressed genes, although the negative correlation in promoter is similar across all gene sets.

**Figure 4e** Locus-specific spearman’s rank correlation of gene expression change and DNA methylation change (see method) between a uterine tumor and its matched normal sample in Fig. 4a for pan-cancer hypermethylated canyon genes (left), pan-cancer hypermethylated cUMR genes (middle) and all differentially expressed genes (right).

Minor critiques:

Figure 1c:y-axis should say "Coverage proportion"

Response: We have corrected Figure 1c y-axis accordingly.

Page 16, line 359:Some type of typo or missing word in the sentence "…we established two control groups of conserved canyons/cUMRs and conserved Canyons (including 1398 canyongs and 9597 and cUMRs)" ?Was one group canyons/cUMRs and the other just canyons (so the first group includes the second)?

Response: We thank for reviewer for pointing this out. This sentence has been changed to the following: “*To compare these differentially methylated UMRs with conserved UMRs across tumor types, we established two control groups: (1) 1398 conserved canyons and (2) 9,596 conserved cUMRs, which are not differentially methylated in all of seven tumor types.”*

Reference:

1.	Varley KE, Gertz J, Bowling KM, Parker SL, Reddy TE, Pauli-Behn F, Cross MK, Williams BA, Stamatoyannopoulos JA, Crawford GE, et al: **Dynamic DNA methylation across diverse human cell lines and tissues.** *Genome Res* 2013, **23:**555-567.

2.	Deaton AM, Bird A: **CpG islands and the regulation of transcription.** *Genes Dev* 2011, **25:**1010-1022.

3.	Irizarry RA, Ladd-Acosta C, Wen B, Wu Z, Montano C, Onyango P, Cui H, Gabo K, Rongione M, Webster M, et al: **The human colon cancer methylome shows similar hypo- and hypermethylation at conserved tissue-specific CpG island shores.** *Nat Genet* 2009, **41:**178-186.

Reviewer #2: In this paper, the authors first use a pan-cancer analysis to describe blocks of DNA methylation changes (canyons) and find these canyons are enriched in m-homeobox genes.  They then show that these events positively correlate with expression changes and then target methylation to the DLX1 gene to show that methylation of the gene body increases expression rather than decreases expression.  There are quite a few studies suggesting that homeobox genes have a propensity to undergo hypermethylation in cancer, but the truly novel finding in this work is the manipulation of methylation in a gene body to activate expression. While this a potentially very interesting finding, as detailed below, I do not find the data from the targeting experiment convincing at this stage since there were missing controls for the targeted methylation experiment and only one gene in one cell line was tested.

Major Concerns:

1) I did not understand why two different genes were chosen for in depth analysis (Fig. 4) and then for actual targeting with a dCas9-DNMT (Fig. 5).  This suggests that HOX13B was targeted, but then did not show the desired effect. Does DLX1 show the same relationships between expression and methylation in tumor samples.

Response: We decided to perform experimental validation in HEK293T cells, since our dCas9-SunTag-DNMT3A DNA methylation editing tool has been established in this cell line. Due to difference between cell line and primary cells, *HOX13B* gene-body is already highly methylated in HEK293T cells (**Reviewer Figure 2)**, and thus is not a good candidate for the methylation editing experiment. Similar to *HOXB13,* *DLX1*’s gene-body canyon hypermethylation and its aberrant overexpression were widely observed in multiple tumor types (**Figure 5b** and **Figure S4b**). *DLX1* is an oncogene that has been shown to promote ovarian cancer cell growth, cell migration and invasion[2]. Importantly, *DLX1* gene-body methylation canyon was detected in HEK293T cells. Therefore, *DLX1* was chosen as an example for experimental validation. We have added the following in the revised manuscript to clarify this issue: “*Due to difference between cell line and primary cells, HOX13B gene-body is already highly methylated in HEK293T cells. We therefore decided to focus on two other m-homeobox genes DLX1 and POU3F3 in the dCas9-mediated methylation editing experiments*.”

Reviewer Figure 2. UCSC track of DNA methylation level on *HOXB13* locus.

2) To demonstrate that hypermethylation of a canyon increases expression, I think the authors need to analyze more than one gene. Showing the effect in multiple cell lines would also make the result more robust.

Response: We appreciate the reviewer’s suggestion. We added a new homeobox gene *POU3F3* to demonstrate that hypermethylation of gene body canyon increases gene expression **(New Figure S5).** *POU3F3* was selected as another example because its gene-body canyon was methylated, and its gene expression was increased **(New Figure S5a and S5b)** in multiple tumor types, such as urothelial bladder carcinomas (BLCA), lung squamous carcinomas (LUSC) and uterine corpus endometrial carcinoma (UCEC). We used the dCas9-SunTag-DNMT3A methylation editing tool to specifically methylated the gene-body canyon of *POU3F3* (average methylation of 23 CpGs increases from 5.8% to 34.2%), while keeping the promoter at low methylation level (average methylation of 71 CpGs changes only from 1.3% to 1.9%) (**New Figure S5c)**. Consistent with the result of *DLX1*, the expression of *POU3F3* also exhibits significant increase (~3-fold) due to gene body hypermethylation (**New Figure S5d**). Together, our methylation editing experiments on two homeobox genes (*DLX1* and *POU3F3*) provide a direct experimental evidence that gene-body canyon hypermethylation can directly increase oncogene expression. The corresponding description has been added in the revised manuscript on page 11-12.

.

**Figure S5. Gene-body canyon hypermethylation by dCas9-SunTag-DNMT3A can directly increase gene** ***POU3F3* expression.** (**a**) Genome browser tracks of gene-body hypermethylated canyons around homeobox gene *POU3F3* across 30 normal and 6 BLCA, 4 LUSC and 5 UCEC tumor samples. (**b**) Gene expression of *POU3F3* between tumor and normal samples for BLCA, LUSC and UCEC. * p-values were calculated by Wilcoxon signed-rank test. Box plots represent the interquartile range (25–75%), with the median; whiskers correspond to 1.5 times the interquartile range. (**c**)CpG DNA methylation level dramatically increased at the gene-body of *POU3F3* after adding guide RNA POU3F3 (gray bar) with dCas9^SunTag^ and ^scFv^DNMT3A, yet the methylation level in gene promoter was not affected. When adding dCas9^SunTag^ and ^scFv^DNMT3A constructs without guide RNAs, CpG methylation level remained unchanged compared to untreated control (Negative). CpG DNA methylation level was calculated based on two biological replicates. Error bars represent mean ± s.e.m. of two biological replicates. **(d**) Quantitative PCR shows significant increase of gene expression of *POU3F3* in dCas9+3A+sgPOU3F3. P-value was computed by two-sided student’s t test.

Due to the technical limitation of our newly developed methylation editing system, it remains time consuming (will take months to establish stable cell lines) to test methylation editing in other cells, which we believe is beyond the scope of the current manuscript.

3) For the expression analysis after dCas9-DNMT, there appeared to be essential controls missing, such as the recruitment of a dCas9-SunTag-DNMT with a mutant DNMT that has no methyltransferase activity.  This is essential to control for any off-target effects produced by the system that occur independent of methylation**.**

Response: We completely agree with the reviewer that control for off-target effects is critical for any methylation editing experiments. Multiple lines of evidence indicate that our current dCas9-SunTag-DNMT3A experiments have minimal off-target effects:

(1) Our dCas9-SunTag-DNMT3A system has already been shown to offer the highest methylation efficiency with minimal off-target effects. In the methylation editing methodology paper recently published in Genome Biology (2017) [3], we already systematically compared the efficiency and specificity of our dCas9-SunTag-DNMT3A system with two other methylation tools: dCas9-DNMT3A (Liu et al. Cell, 2016) and dCas9-DNMT3ACD (Vojta et al, Nucleic Acid Research, 2016). Genome-wide reduced representation bisulfite sequencing and RNA-seq show that dCas9-SunTag-DNMT3A methylates regions of interest with minimal impact on the global DNA methylome and transcriptome (Fig 4 in [3]).
(2) Two experimental evidences suggest that the increased gene expression we observed in this study is unlikely due to any methylation-independent off-target effects produced by dCas9-SunTag-DNMT3A binding. First, dCas9-SunTag-DNMT3A+sgHOXA5 targeting *HoxA5* gene promoter represses *HoxA5* gene expression (Fig 1 in [5]). Second, dCas9-SunTag binding alone on *HoxA5* with no DNMT3A also slightly represses *HoxA5* gene expression (Fig S3 in [5]). If the off-target existed, the above two experiments would increase gene expression.
(3) We hope the robust and similar results from two independent genes are sufficient to rule out any off-target effects. Besides *DLX1*, we added a new homeobox gene *POU3F3* to demonstrate that hypermethylation of gene body canyon increases gene expression **(New Figure S5).**
(4) Our current off-target control, i.e. dCas9-SunTag-DNMT3A without sgRNA, is among the best practice in many published high-profile methylation editing papers, including dCas9-DNMT3A (Liu et al. Cell, 2016), dCas9-SunTag-DNMT3A (Huang et al. Genome Biology 2017), and dCas9-MQ1 (Let et al. Nature Communications, 2017).

Together, we would like to reiterate that the current manuscript is focused on biological discovery using large-scale patient DNA methylation data and established experimental techniques, rather than methodology development. Therefore, we hope the reviewer would agree that the development of more sophisticated controls (e.g. mutant DNMT) that have not been widely used is beyond the scope of the current manuscript, also beyond the technical expertise of our bioinformatics lab.

4) The authors conclude from their CpG island analysis that their data suggest "that CpG density alone is not sufficient to capture the majority of epigenetic information in UMRs." CpG islands are a rather indirect measure of CpG density.  CpG O/E measures in Fig. S1a would indicate that all canyons have high CpG density.

Response: We agree with the reviewer and we have removed the following sentence in the revised manuscript: “*suggesting that CpG islands alone are not sufficient to capture the majority of epigenetic information in UMRs*”

5) I wish the authors would talk a little more about homeobox gene structure and how this relates to their results.  Homeobox genes tend to have very high CpG content as illustrated by the multiple CpG islands found in HOXB13. I think the authors try to extrapolate their results (p. 12 line 258), but homeobox genes have a rather unique structure in terms of CpG density.

Response: We thank the reviewer for this great suggestion. We have added the following discussion on Page 13: “*Homeobox genes comprise a superfamily of transcription factors that are critical for cellular growth and differentiation. The homeodomain (the evolutionary conserved helix–loop–helix DNA-binding motif) is usually present in the second exon. Homeobox genes in general have high CpG density, which might facilitate the establishment of DNA methylation canyons. In fact, about 67% (157 out of 234) of homeobox genes are associated with reference canyons (Additional file 3: Table S3).”*

6) p.11, line 247.  There are two sentences seem to say opposite things, or there is a missing subtlety that needs to be explained.  I believe the authors are stating that methylation of the entire canyon or regions within the canyon (a) leads to no mechanistic difference but (b) determines the functional consequences.

Response: We apologize for the confusion. We have removed these two sentences in the revised manuscript.

7) Do the authors have any thoughts as to why the canyon they detect at HOXB13 and DXL1 extend beyond the length of the gene? Is this something that effects transcription?  Do they observe the same effect on expression if they target the canyon beyond the edge of the gene?  This might facilitate the understanding of mechanism.

Response: We thank the reviewer for this great suggestion. UMR beyond the length of the gene might indicate additional regulatory elements outside of gene-body. It would be very interesting to check the effect of canyon hypermethylation beyond the edge of the gene towards gene expression in future studies.

8) The authors comments about DNMT3A overexpression should be set in greater context. Interpreting these results has been quite controversial since despite this observed increase in expression, overwhelmingly tumors lose much more methylation than they gain.

Response: We apologize for the confusion. We have deleted the comments about overexpression of DNMT3A in tumors on Page 10.

9) I wish the authors would bring more of the results from other tumor types into the main body of the paper. Upon my initial reading, I wrongly assumed the authors had only examined uterine cancer to extrapolate their canyon findings to additional tumors.  Only after I delved into the supplement did I realize the tremendous amount of work buried there.  I wish there were greater emphasis that the results observed in uterine cancer held true for other cancer-types as well.  Possibly this could just be through mentions in the text such as these relationships held true for lung, breast, … cancers.  Being more specific about the cancer types would also be more accurate than always saying "pan-cancer" and would also highlight the work involved better.

Response: We thank the reviewers for this great suggestion. We have added more specific about the cancer types in our revised manuscript. For example, “*Furthermore, the unique association between increased expression and canyon hypermethylation was also observed in 4 other tumor types including BLCA, BRCA, LUAD and LUSC. (****Additional file 1: Figure S2)”****.*

Minor Concerns:

1) There are many grammatical typos in the background section that should be fixed.

Response: We have asked a native speaker to correct all grammatical typos.

2) The authors state that ~90% of pan-cancer differential UMRs can be recurrently identified in at least 3 individuals.  Is this due to the limitations to detect UMRs as defined or does this have some biological significance?

Response: It is due to the limitations to detect UMRs.

3) The sentence on p. 6, line 128-129 was very confusing to me as written (something about the combination of "ands", slashes, and parentheticals all intermixed). But understanding these 4 categories is crucial.

Response: We thank for reviewer for pointing this out. This text has been revised on Page 6. “*These pan-cancer differential UMRs can be further divided into 4 categories: i) tumor hypermethylated canyons, ii) tumor hypemethylated cUMRs, iii) tumor hypomethylated canyons and iv) tumor hypomethylated cUMRs (****Fig. 2a and Additional file 4: Table S3****).”*

4) Minor issues with figures: Fig. 2a has no y-axis label, Fig. 3a is missing a scale bar, Fig. 3b has no y-axis label. In Fig. S5 it is unclear what n= means.

Response: We thank for reviewer for pointing these out. We have revised these figures accordingly. N in Fig. S5 means the number of pan-cancer oncogenic signatures identified from 12 cancer types [4] or the number of pan-cancer hypermethylated canyon genes identified by current manuscript.

5) It is not clear what is plotted in Fig 3e.  Is each gene averaged by individual, then the box plot consists of all genes, or is each individual averaged across all genes, and then the box plot consists of all individuals?

Response: We apologize the confusion. The average expression of each gene was first computed across 22 normal samples or 427 tumor samples. The box plot shows the average expression of all genes. The Figure 3 legend has been revised accordingly.

6) The authors should indicate the locations of the 450k probes used for the analyses in Fig. 4 in Fig. 4B.

Response: We thank for reviewer for this great suggestion. We have added the probe locations to Fig 4B.

7) In Fig. 2d and 5b I wish the authors would indicate the expression values across all the samples.

Response: It is difficulty to indicate the expression values across all the samples, due to the large number of samples involved. Furthermore, DNA methylation and gene expression are not completely one-by-one matched for each sample in Fig.2d and 5b. For example, some samples have DNA methylation only but no expression. We instead report the expression upregulation of *HOXB13* and *DLX1* in tumors in Figure S4a and S4b.

8) Fig. 5d is missing information about how many replicates were performed, and the meaning of error bars. No information was provided as to what type of replicates these were either.

Response: We have two biological replicates for figures 5c and 5d. Error bars represent mean ± s.e.m. of two biological replicates. We have revised Figure 5 legend accordingly.

**Reference**

1.	Varley KE, Gertz J, Bowling KM, Parker SL, Reddy TE, Pauli-Behn F, Cross MK, Williams BA, Stamatoyannopoulos JA, Crawford GE, et al: **Dynamic DNA methylation across diverse human cell lines and tissues.** *Genome Res* 2013, **23:**555-567.

2.	Chan DW, Hui WW, Wang JJ, Yung MM, Hui LM, Qin Y, Liang RR, Leung TH, Xu D, Chan KK, et al: **DLX1 acts as a crucial target of FOXM1 to promote ovarian cancer aggressiveness by enhancing TGF-beta/SMAD4 signaling.** *Oncogene* 2016.

3.	Huang Y, Su J, Lei Y, Brunetti L, Gundry MC, Zhang X, Jeong M, Li W, Goodell MA: **DNA epigenome editing using CRISPR-Cas SunTag-directed DNMT3A.** *Genome Biol* 2017, **18:**176.

4.	Ciriello G, Miller ML, Aksoy BA, Senbabaoglu Y, Schultz N, Sander C: **Emerging landscape of oncogenic signatures across human cancers.** *Nat Genet* 2013, **45:**1127-1133.

**Second round of review**

**Reviewer 1**

**Please provide your comments for the author(s) on the revised manuscript.**

Most of my concerns have been addressed. However, I still do not feel that the conclusion concerning that methylation of the gene body of DLX1 causes increased expression is supported by the included experiments (details below). Also, the grammar still needs a lot of work. As is, the manuscript is still often difficult to read. I have made a lengthy, but likely incomplete list below. 

Major Comment 
As the authors indicate in their response letter, the previous characterization of the dCas9-SunTag-DNMT system in Huang et al. showed that repression of HoxA5 was achieved by both dCas9-SunTag + DNMT3A + sgRNA and by dCas9-SunTag + sgRNA in the absence of DNMT3A. This indicates that localization of the dCas9-SunTag affects expression of HoxA5 independent of DNA methylation. Interestingly, as far as I can tell the Huang et al manuscript never directly claims that the induced methylation is responsible for the silencing, only that the localization of the complex silences expression. 

It is unclear whether the SunTag system will induce different expression effects depending on where it is localized. The point of the current manuscript is that there is a difference in the effect on expression between targeting methylation to promoters versus targeting gene bodies. I see no reason why localization of the SunTag-DNMT3A system could not also produce different effects based on targeting different parts of the gene. 

Clearly the results from Huang et al. demonstrate that in order to conclude that the methylation of DLX1 causes expression changes, controls are required to rule out that the effect on DLX1 expression is not due to the recruitment of the dCas9-SunTag-DNMT complex independent of DNA methylation. Despite the authors claims, most (if not all) the papers on DNMT targeting have used some control for this purpose. This includes all the ones cited by the authors in their response. Personally I feel the two best ways are to either use a mutant DNMT as a control is one way (Votja et al, McDonald et al, Stepper et al, and Galonska et al use mutant DNMT3A, Let et al uses a “non-functional dCas9-dMQ1”) or to use an inducible system to induce methylation and then turn-off/remove the dCas9-SunTag-DNMT3A complex (similar experiments were used in Amabile et al, Liu et al, and McDonald et al). 

I think the POUF3A data, points out another issue with presentation of the data. From the methylation data it appears that the negative sample is about 0% methylated and then the methylation increased for the SunTag+DNMT3A, which then increases again for SunTag+DNMT3A+sgRNA. But the expression data is all normalized to the SunTag+DNMT3A control. The expression experiments for both POUF3A and DLX1 should be normalized to the negative control, which has no methylation, while showing the expression of SunTag+DNMT3A and SunTag+DNMT3A+sgRNA (and some type of control for the localization of the complex as above). 

Given the prior published results from the dCas9-SunTag-DNMT system, I do not feel the authors conclusions are justified without the additional controls. If the authors feel that such controls are beyond the scope of the work, then the conclusions and claims about “functional validation” and that “…locus-specific hypermethylation … can direct (sic.) increase its gene expression.” should be removed as they are not supported by the experiments as performed. 

Additional Minor Point: 
1) I think reference 3 is incorrect. As far as I am aware, that paper does not talk about DNA methylation and cell fate. I would either delete this sentence as it has nothing to do with the paper, or better, replace with a sentence that is cancer focused and more relevant to the current work (for which the reference would likely then be the correct one). 

Grammar 
1) Abstract: “direct” should be “directly” 
2) P. 3, line 70: “causing” is not the right verb/tense here. Something like “induced” would be better. Or restructure the sentence. 
3) P. 3, line 72: “has been well worked out” -- unclear what this means, be specific. 
4) P. 4, line 82: “Thus, it remained unconvinced..” This clause is awkward and not sure what “it” refers to. 
5) P. 4, line 85: “Recently, the broad …” should be “Recently, broad …” (The meaning behind this sentence is also unclear. Are the authors saying that UMRs, canyons and valleys are synonymous? If so, they should state more directly. Or if they are different then they should describe how so.) 
6) P.4 line 88: “specific” should be “specifically” 
7) P. 4, Line 92: “canyon” should be “canyons” 
8) P. 5, line 102: “define the” should be “define” 
9) P.5, line 110: “as control cUMRs (Fig. 1b)” should be “as controls (cUMRs, Fig. 1b)” 
10) P. 5 line 113: in “transcription initiation with low methylation”. Something is wrong here. Either the authors mean TSSs with low methylation, or something different. But the sentence makes no sense as is. 
11) P. 6, line 124: “inspired by recent advantages in “ The authors point is unclear here. I think the authors mean new advances in the analysis of GWAS data? 
12) P. 7, line 150: thereafter should be hereafter 
13) P. 7, line 156: omit the word “those” 
14) P. 8, line 172: “on tumor” should be “of tumor” – but this sentence as a whole is very awkward and should be re-written. 
15) P.9, line 206: “performed the” should be “performed” 
16) P. 10, line 221: “in the regions” should be “to regions” 
17) P. 10, line 229: “cell line” should be “cell lines” 
18) P. 11, line 237: “using CRISPR” should be “using the CRISPR” 
19) P. 11, line 239: “methylate gene-body” should be “methylate the gene-body” 
20) P. 11, line 241: “canyon” should be “the canyon” 
21) P. 11, line 254: “provide a” should be “provide”

**Reviewer 2**

**Please provide your comments for the author(s) on the revised manuscript.**

My concerns have been all addressed.

**Authors’ response to reviewers**

Genome Biology GBIO-D-17-01564

We thank the editors and reviewers for carefully considering our manuscript. We attach a point-by-point reply to the reviewers’ comments indicating our response in blue font. In addition, we have provided a version of the manuscript with any major sections of altered text indicated in red so the reviewers can easily identify the extensive changes we made.

Reviewer #2: Most of my concerns have been addressed. However, I still do not feel that the conclusion concerning that methylation of the gene body of DLX1 causes increased expression is supported by the included experiments (details below). Also, the grammar still needs a lot of work. As is, the manuscript is still often difficult to read. I have made a lengthy, but likely incomplete list below.

Major Comment

As the authors indicate in their response letter, the previous characterization of the dCas9-SunTag-DNMT system in Huang et al. showed that repression of HoxA5 was achieved by both dCas9-SunTag + DNMT3A + sgRNA and by dCas9-SunTag + sgRNA in the absence of DNMT3A. This indicates that localization of the dCas9-SunTag affects expression of HoxA5 independent of DNA methylation. Interestingly, as far as I can tell the Huang et al manuscript never directly claims that the induced methylation is responsible for the silencing, only that the localization of the complex silences expression.

It is unclear whether the SunTag system will induce different expression effects depending on where it is localized. The point of the current manuscript is that there is a difference in the effect on expression between targeting methylation to promoters versus targeting gene bodies. I see no reason why localization of the SunTag-DNMT3A system could not also produce different effects based on targeting different parts of the gene.

Clearly the results from Huang et al. demonstrate that in order to conclude that the methylation of DLX1 causes expression changes, controls are required to rule out that the effect on DLX1 expression is not due to the recruitment of the dCas9-SunTag-DNMT complex independent of DNA methylation. Despite the authors claims, most (if not all) the papers on DNMT targeting have used some control for this purpose. This includes all the ones cited by the authors in their response. Personally, I feel the two best ways are to either use a mutant DNMT as a control is one way (Votja et al, McDonald et al, Stepper et al, and Galonska et al use mutant DNMT3A, Lei et al uses a “non-functional dCas9-dMQ1”) or to use an inducible system to induce methylation and then turn-off/remove the dCas9-SunTag-DNMT3A complex (similar experiments were used in Amabile et al, Liu et al, and McDonald et al).

I think the POUF3A data, points out another issue with presentation of the data. From the methylation data it appears that the negative sample is about 0% methylated and then the methylation increased for the SunTag+DNMT3A, which then increases again for SunTag+DNMT3A+sgRNA.  But the expression data is all normalized to the SunTag+DNMT3A control.  The expression experiments for both POUF3A and DLX1 should be normalized to the negative control, which has no methylation, while showing the expression of SunTag+DNMT3A and SunTag+DNMT3A+sgRNA (and some type of control for the localization of the complex as above).

Given the prior published results from the dCas9-SunTag-DNMT system, I do not feel the authors conclusions are justified without the additional controls. If the authors feel that such controls are beyond the scope of the work, then the conclusions and claims about “functional validation” and that “…locus-specific hypermethylation … can direct (sic.) increase its gene expression.” should be removed as they are not supported by the experiments as performed. I think reference 3 is incorrect. As far as I am aware, that paper does not talk about DNA methylation and cell fate.  I would either delete this sentence as it has nothing to do with the paper, or better, replace with a sentence that is cancer focused and more relevant to the current work (for which the reference would likely then be the correct one).

Response: We thank the reviewer for these insightful comments. In order to fully address questions raised by reviewer, we decided to revise our dCas9-SunTag-DNMT3A system by 1) moving original constitutively-expressing system to doxycycline-inducuble system and 2) generating catalytic inactive DNMT3A mutation ^scFv^DNMT3A^E756A^ (Please see revised method: vector construction in the manuscript for further details.)

After establishing inducible dCas9-SunTag-DNMT3A system, we repeated our targeted methylation experiments in *DLX1* and *POU3F3* genes. Induction of dCas9-SunTag-DNMT3A expression can methylate gene-body portion of the canyon (average methylation of 25 CpGs increases from 5.1% to 46.2%), while keeping the remaining part (mostly promoter) of the canyon largely unchanged (average methylation of 27 CpGs changes from 1.0% to 1.1%) (**Fig. 5c**). qPCR showed that the dCas9-SunTag-DNMT3A together with sgRNAs can directly increase *DLX1* gene expression by about 2-fold, comparing to the same cells without induction of dCas9^SunTag^ and ^scFv^DNMT3A. Furthermore, induction of dCas9^SunTag^ and inactive DNMT3A mutation ^scFv^DNMT3A^E756A^ with sgRNAs didn’t change *DLX1* expression, suggesting DNA methylation, rather than dCas9^SunTag^-DNMT3A complex, is responsible for increased *DLX1* expression (**Fig. 5d**). Consistently, the expression of *POU3F3* also exhibits significant increase (~2-fold) due to targeted gene-body canyon hypermethylation but induction of dCas9^SunTag^ and ^scFv^DNMT3A^E756A^ didn’t change *POU3F3* expression (**Additional file 1: Figure S5c,d**). Together, our methylation editing experiments on two independent homeobox genes (*DLX1* and *POU3F3*) provide robust experimental evidence that gene-body canyon hypermethylation can directly increase oncogene expression.

In summary, using catalytic inactive mutation of DNMT3A, we demonstrated that DNA hypermethylation, not dCas9^SunTag^-DNMT3A complex, of gene-body canyon led to increased oncogene expression. Using doxycycline inducible system, we compared the gene expression with or without induction of dCas9-SunTag-DNMT3A system, providing a reliable control for experimental results.

Grammar

1)Abstract: “direct” should be “directly”

Response: Thank you for pointing it out. We have changed “direct” to “directly”.

2) P. 3, line 70: “causing” is not the right verb/tense here.  Something like “induced” would be better. Or restructure the sentence.

Response: Thank you for pointing it out. We have changed the text to “induced.

3) P. 3, line 72: “has been well worked out”  -- unclear what this means, be specific.

Response: It means that DNA methylation in hematologic tumors has been well studied. This text has been modified accordingly.

4)    P. 4, line 82: “Thus, it remained unconvinced..”  This clause is awkward and not sure what “it” refers to.

Response: We apologize for the confusion. The text has been modified as “*Thus, whether the repression of a specific gene was caused by gene-body hypomethylation of the same gene remained unclear. “*

5) P. 4, line 85: “Recently, the broad …” should be “Recently, broad …” (The meaning behind this sentence is also unclear.  Are the authors saying that UMRs, canyons and valleys are synonymous?  If so, they should state more directly. Or if they are different then they should describe how so.)

Response: We apologize for the confusion. The sentence has been restructured as “*Recently, broad (i.e. >3.5 kb) UMRs were reported as DNA methylation canyons[**20**] or DNA methylation valleys [**21**]. Canyons usually span promoters and gene-bodies and are very conserved across almost all normal cells. “*

6)  P.4 line 88: “specific” should be “specifically”

Response: We have changed the text to “specifically”.

7)    P. 4, Line 92: “canyon” shold be “canyons”

Response: We have changed the text to “canyons”.

8)    P. 5, line 102: “define the” should be “define”

Response: We have changed the text according to reviewer’s suggestion.

9)    P.5, line 110: “as control cUMRs (Fig. 1b)” should be “as controls (cUMRs, Fig. 1b)”

Response: We have changed the text according to reviewer’s suggestion.

10)    P. 5 line 113: in “transcription initiation with low methylation”.  Something is wrong here. Either the authors mean TSSs with low methylation, or something different.  But the sentence makes no sense as is.

Response: We have changed the text to “transcription start sites with low methylation”.

11)    P. 6, line 124: “inspired by recent advantages in “ The authors point is unclear here.  I think the authors mean new advances in the analysis of GWAS data?

Response: We have changed the text to “This is inspired by recent advances in the analysis of GWAS data”

12)     P. 7, line 150: thereafter should be hereafter

Response: We have changed the text according to reviewer’s suggestion.

13)    P. 7, line 156: omit the word “those”

Response: We have changed the text according to reviewer’s suggestion.

14)    P. 8, line 172: “on tumor” should be “of tumor” – but this sentence as a whole is very awkward and should be re-written.

Response: We have changed the text to “Together, our analysis suggested a previously unrecognized link between canyon hypermethylation and increased expression of homeobox oncogenes.”

15)    P.9, line 206: “performed the” should be “performed”

Response: We have changed the text according to reviewer’s suggestion.

16)    P. 10, line 221: “in the regions” should be “to regions”

Response: We have changed the text according to reviewer’s suggestion.

17)    P. 10, line 229: “cell line” should be “cell lines”

Response: We have changed the text according to reviewer’s suggestion.

18)    P. 11, line 237:  “using CRISPR” should be “using the CRISPR”

Response: We have changed the text according to reviewer’s suggestion.

19)    P. 11, line 239: “methylate gene-body” should be “methylate the gene-body”

Response: We have changed the text according to reviewer’s suggestion.

20)    P. 11, line 241: “canyon” should be “the canyon”

Response: We have changed the text according to reviewer’s suggestion.

21)    P. 11, line 254: “provide a” should be “provide”

Response: We have changed the text according to reviewer’s suggestion.
